# High-Valent Late Transition Metal-Oxo Complexes: Breaking Boundaries at the Oxo Wall and Beyond

**DOI:** 10.1021/acs.chemrev.5c00744

**Published:** 2026-04-17

**Authors:** Nabhendu Pal, Nicole A. Fritsch, Jason Shearer, Nicolai Lehnert

**Affiliations:** † Department of Chemistry and Department of Biophysics, 1259 University of Michigan, Ann Arbor, Michigan 48109-1055, United States; ‡ Department of Chemistry, Trinity University, San Antonio, Texas 78212, United States

## Abstract

High-valent first-row transition metal–oxo species are active intermediates in oxidation reactions crucial to many biological processes, oxidative chemical transformations in industry, and water splitting for renewable energy generation. The direct activation and hydroxylation of unactivated aliphatic C–H bonds remain a challenging yet important transformation in efficiently synthesizing complex organic molecules and pharmaceuticals with oxygenated functionalities. Despite documenting over 100 synthetic Fe–O species, terminal metal–oxo complexes involving the late transition metals Co, Ni, and Cu are much rarer, primarily due to the “oxo wall” phenomenon. The additional electrons in the metal–oxo unit weaken the M–O bond and destabilize these species. At the same time, as one traverses past the oxo wall, the M–O unit gains increasing amounts of metal–oxyl character, which holds promise for the generation of highly active catalysts for C–H bond activation, as exemplified by copper monooxygenase enzymes in nature. While forming late transition metal–oxo complexes is challenging, a few have been successfully prepared by targeting specific symmetries or unusual spin states. This review explores nonheme iron oxo complexes, the “oxo wall” concept, and recent advances in overcoming this limitation to prepare metal–oxo complexes of cobalt, nickel, and copper beyond the oxo wall.

## Graphical abstract

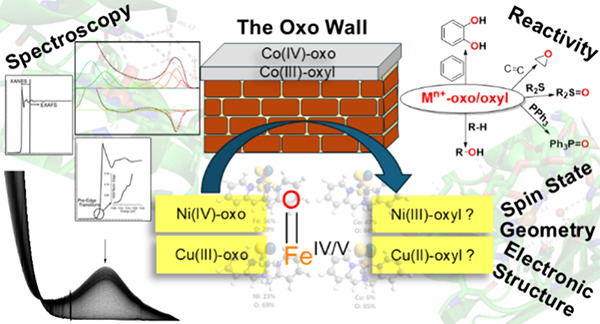

## 1 Introduction

The selective oxidation of organic molecules plays a crucial role in biological systems and has significant industrial applications. Metalloenzymes that catalyze oxidation reactions often exhibit remarkable substrate specificity, regioselectivity, and stereoselectivity. These enzymatic processes operate under mild conditions and often use O_2_ directly, making them environmentally friendly and inherently sustainable.^1–4^ High-valent metal–oxo complexes frequently serve as key reactive intermediates in many oxidation reactions (see Scheme 1), enabling efficient transformations. Nature predominantly employs iron, manganese, and copper for these reactions due to their natural abundance, ability to effectively bind oxygen species, and versatile redox chemistry. Our understanding of these enzymatic processes has advanced considerably, thanks to geometric and electronic structural insights derived from high-resolution X-ray crystallography and in-depth spectroscopic investigations with a plethora of different methods. The characterization of both the resting states and the reactive intermediates of metalloenzymes has been instrumental in elucidating their mechanisms. Additionally, kinetic studies, synthetic modeling, and theoretical investigations have provided further depth to our knowledge of the oxidation processes catalyzed by these enzymes.

In biological systems, the formation of the O–O bond, which leads to dioxygen evolution in the Oxygen-Evolving Complex (OEC) of Photosystem II (PS II), is believed to proceed via a transient manganese–oxo intermediate. Although this intermediate has not been identified, its role in the reaction mechanism is significant.^5–7^ A key intermediate in numerous heme-based oxidation reactions is “Compound I“ (Cpd I), which consists of an iron­(IV)–oxo (O^2–^) complex bound to a porphyrin radical.^8–18^ Similar to heme enzymes, non-heme iron enzymes often follow analogous pathways. Here, direct evidence for iron­(III)–peroxo and high-valent iron­(IV)–oxo intermediates has been obtained in some of these systems.^19–23^ For instance, the diiron enzyme soluble Methane Monooxygenase (sMMO), responsible for converting methane to methanol, activates O_2_ through di-iron­(III)–peroxo and di-iron­(IV)–oxo intermediates. Unlike sMMO, Rieske Dioxygenases activate molecular oxygen at a mononuclear iron center coordinated by a 2-His-1-carboxylate facial triad motif. These enzymes play a crucial role in environmental bioremediation by catalyzing the *cis*-dihydroxylation of arene double bonds, a key step in the biodegradation of aromatic compounds in the soil. Several studies have successfully trapped and characterized iron­(IV)–oxo intermediates in non-heme iron enzymes that utilize organic cofactors such as α-ketoglutarate (α-KG) or tetrahydrobiopterin. These intermediates play essential roles in oxidative transformations. A notable example is the mononuclear Fe­(IV)–oxo Intermediate *J*, identified in the catalytic cycle of Taurine Dioxygenase (TauD).^24^

Research in the field of transition metal-catalyzed oxidation reactions has expanded beyond the metals traditionally employed by nature, such as manganese, iron, and copper. This exploration has led to the synthesis of an extensive range of bioinspired and biomimetic complexes incorporating various first-row transition metals, including chromium, manganese, iron, cobalt, nickel and copper. Some of these synthetic complexes have served as valuable spectroscopic models for understanding the active sites of enzymes, offering insights into their catalytic mechanisms. Additionally, researchers have synthesized and investigated several heavy late-transition metal–oxo complexes, broadening the scope of oxidation chemistry beyond nature’s conventional choices.^3^ These studies have provided deeper mechanistic understanding and contributed to the development of novel catalytic systems with potential applications in industrial and pharmaceutical chemistry. In this review, we highlight key advancements made over the past five to ten years in the study of mononuclear nonheme high-valent transition metal–oxo complexes. We begin by focusing on the well-characterized iron-based species that are widely utilized in natural enzymatic systems (see Scheme 1). Subsequently, we explore the chemistry of late transition metals such as cobalt, nickel, and copper, which have gained increasing attention for their potential roles in oxidation catalysis. An overview of oxidative reactions in nature is shown in Figure 1.

**Figure 1:**
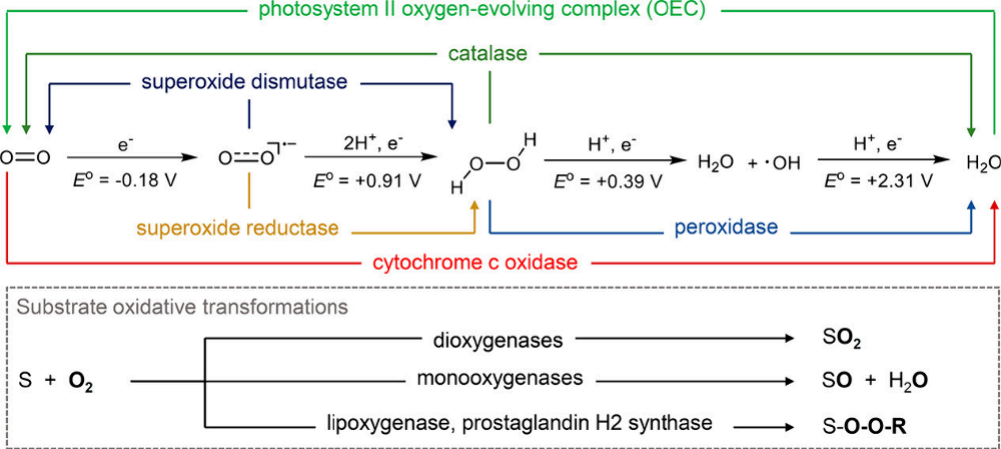
Reduction sequence of O_2_ and representative metalloenzymes related to O_2_ activation. Reduction potentials shown are versus the normal hydrogen electrode (NHE). Reproduced with permission from ref 14. Copyright 2018 American Chemical Society.

**Scheme 1:**
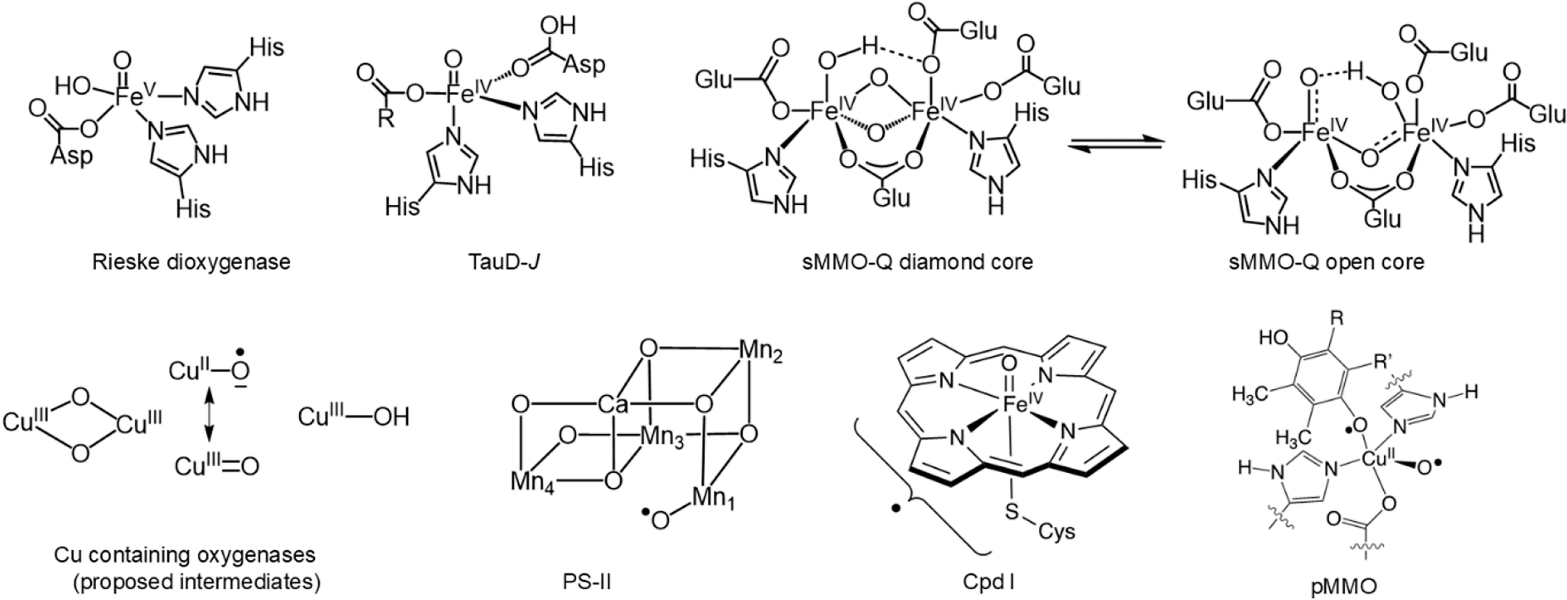
Structures of Proposed High-Valent Metal–Oxo Intermediates in Nature

### 1.1 High-Valent Metal–Oxo Intermediates in Nature

Heme enzymes, like Cytochrome (Cyt) P450s, peroxidases, and catalases, are especially interesting to scientists because they can be adapted to mediate many kinds of chemical reactions. In all of these enzymes, the key to their powerful activity is a special form of high-valent iron–oxo intermediate, called Cpd I, a highly reactive species responsible for oxidation and oxygenation reactions. Cyt P450 enzymes are present in many living organisms, including mammals (found in the liver, kidney, lung, intestine, and adrenal cortex), insects, plants, yeasts and bacteria. These enzymes play a crucial role in activating dioxygen. Here, the name “P450” stems from the fact that the ferrous form of these enzymes with a bound carbon monoxide (CO) ligand exhibits a strong absorption band, the Soret band (a π–π* transition of the porphyrin ring), at 450 nm.^25^ This characteristic absorption band in the UV–vis spectrum distinguishes it from other cytochromes and is used as a signature for the identification of the heme-thiolate active site of Cyt P450s. Due to the important physiological functions of Cyt P450 enzymes, the reaction intermediates of these enzymes have been the subject of intense investigation for many years, leading to numerous articles, books and reports. In nature, Cyt P450s act as key catalysts for oxidizing organic molecules. The hallmark of Cyt P450s is their active site that consists of a heme *b* cofactor with axial cysteinate (a deprotonated cysteine) ligation, as shown in Figure 2. These enzymes typically use dioxygen or sometimes hydrogen peroxide to drive various oxidation reactions, such as hydroxylation, epoxidation, dealkylation, desaturation, and heteroatom oxidation.^10^ This versatility makes them valuable for industrial applications. In humans, Cyt P450s participate in important biosynthetic pathways and aid in metabolizing small molecules. After one-electron reduction of the heme to the ferrous state, O_2_ is bound first, forming an Fe­(II)–O_2_/Fe­(III)–O_2_^–^ adduct (Figure 2), which is subsequently reduced and protonated, generating an Fe­(III)–OOH complex (Cpd 0). Subsequent protonation of this intermediate triggers heterolytic cleavage of the O–O bond, yielding the key oxidizing species Cpd I and a molecule of water. A critical intermediate in many heme-based oxidation reactions is Cpd I, a Fe­(IV)–oxo (O^2–^) complex with a bound porphyrin monoradical. Reaction with a substrate C–H bond then generates “Compound II” (Cpd II), the one-electron reduced and in this case also protonated form of Cpd I, and a substrate radical, as shown in Scheme 2. The most extensively investigated mammalian Cyt P450 has been the major phenobarbital-inducible form from rabbit liver, Cyt P450-LM2, first purified by Coon and co-workers.^25^ Another well-studied Cyt P450 is the bacterial camphor-hydroxylating enzyme from *Pseudomonas putida*, known as P450cam, which is soluble and was the first Cyt P450 to be purified.^10^ In 1971, Peterson et al. first reported the dioxygen-bound ferrous heme intermediate of Cyt P450cam.^26^ Later, a crystal structure of this species was obtained by Sligar and co-workers, as shown in Figure 2.^27^

**Figure 2:**
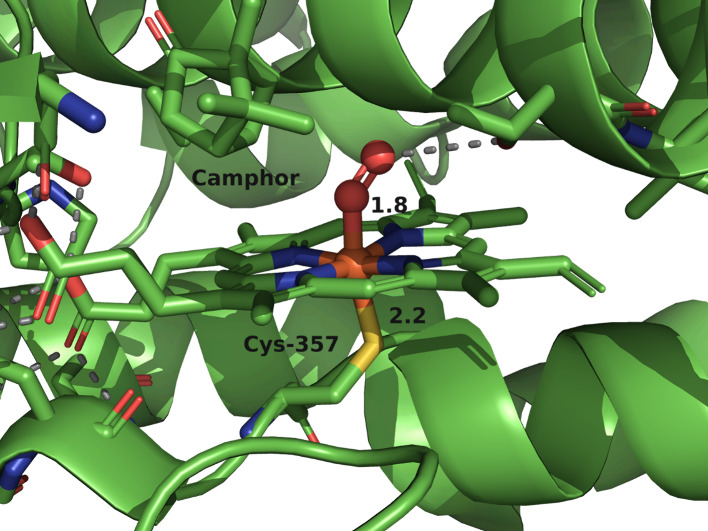
Crystal structure of the ferrous oxy complex in the active site of Cyt P450cam from *P. putida* (PDB: 1DZ8).^27^

Although Cyt P450 enzymes have been studied for the last few decades, an understanding of the precise nature of Cpd I has remained elusive due to its usually short lifetime and the fact that it is generated only in low yields.^16,28^ In 2010, Rittle and Green achieved a major milestone by generating Cpd I in yields of approximately 75% by reacting the thermophilic Cyt P450 enzyme CYP119 in its ferric oxidation state with *meta*-chloroperbenzoic acid (*m*-CPBA), allowing for the detailed spectroscopic characterization of Cpd I.^15^

Peroxidases are another class of important heme-containing enzymes that can oxidize a variety of substrates. The first step of the peroxidase mechanism is the reaction of the ferric heme with hydrogen peroxide, leading to the formation of the two-electron oxidized enzyme intermediate, Cpd I, via the heterolytic cleavage of the O–O bond of the bound peroxide.^29^ In 1937, using an ocular spectroscope, Keilin and Mann observed a red intermediate with a Soret band near 420 nm upon the addition of H_2_O_2_ to Horseradish Peroxidase (HRP).^30^ In the 1940s, Theorell used rapid kinetic methods, employing HRP, to identify a more short-lived green intermediate, followed by the red intermediate that had been previously observed.^31^ Compared to Cyt P450s, the famous Cpd I (the green species) and Cpd II (the red species) intermediates formed by HRP are much more straightforward to detect, allowing for their detailed spectroscopic characterization.^32^ Similar green and red intermediates were also seen by Stern and Chance in catalase during its reaction with hydrogen peroxide.^33–35^ Based on exact kinetic measurements of this reaction, the formation of a ferric-peroxide precursor complex, termed “Compound 0” (Cpd 0), was later shown.^36–40^ Hajdu and co-workers implemented a carefully designed strategy to capture crystal structures of high-valent redox intermediates in HRP.^41^ They also constructed a novel 3D “movie” showing the electron-driven (X-ray-induced) catalytic reduction of a bound dioxygen ligand within HRP. Additionally, through independent experiments, they acquired high-resolution crystal structures for all five defined oxidation states of HRP, preserving each enzyme’s distinct redox state (see Figure 3).^41^

**Figure 3:**
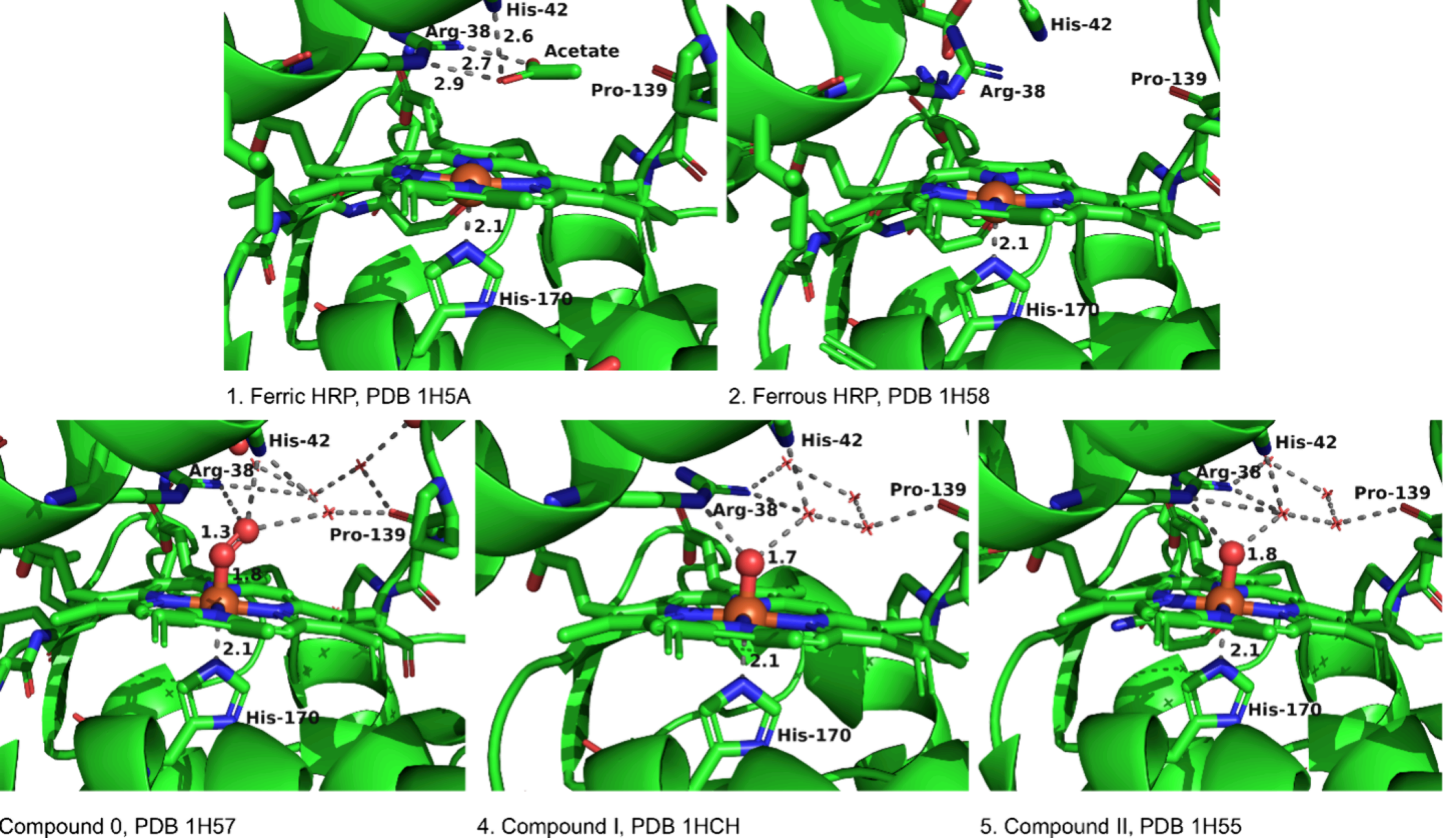
Structures of the five defined oxidation states of HRP (PDB: 1H5A (ferric), 1H58 (ferrous), 1H57 (Cpd 0), 1HCH (Cpd I), and 1H55 (Cpd II)).^41^

Most Cyt P450s generate their Cpd 0 through the reduction of iron-bound dioxygen, initially generating a ferric−peroxo anion species, unlike peroxidases. This step is followed by the transfer of two protons—one to convert the peroxo complex into Cpd 0 (a ferric–hydroperoxo complex), and a second to cleave the O–O bond to form water and the reactive Cpd I (see Scheme 2). Furthermore, it emerged that both peroxidases and catalases produce porphyrin cation radicals during Cpd I formation, just like Cyt P450s. Here, the cysteine versus histidine axial ligand switch in Cyt P450s versus peroxidases likely plays a pivotal role in modulating Cpd I formation. This difference in ligation aligns with the phenomenon commonly referred to as the “push–pull” mechanism, which describes two distinct ways to accelerate O–O bond heterolysis in Cpd 0 for Cpd I formation: the thiolate “push” and/or the arginine “pull”.^40^ Intriguingly, the axial ligand variation profoundly influences the reactivity and stability of Cpd I, as further discussed below. The mechanism of O_2_ activation by Cyt P450s, shown in Scheme 2, is considered the paradigm for O_2_ activation by transition-metal centers.

**Scheme 2:**
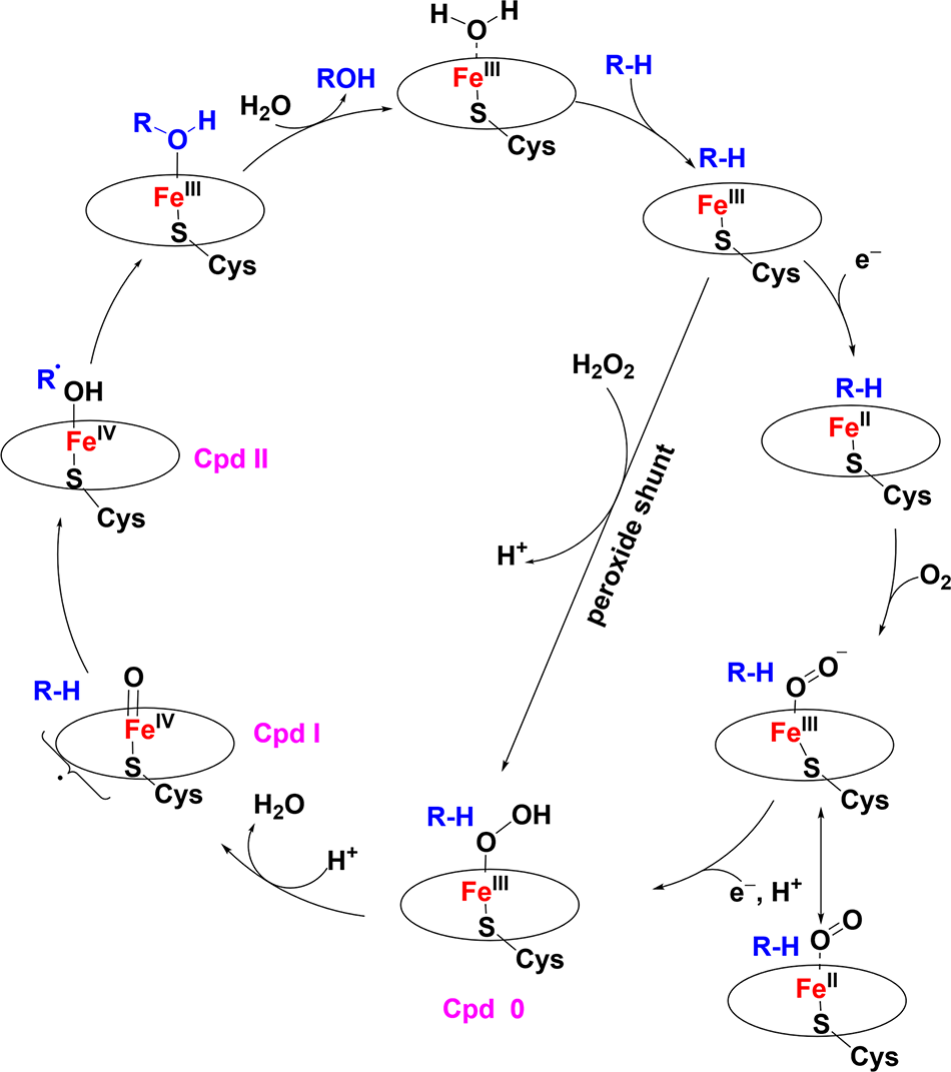
Catalytic Cycle of Cyt P450 Monooxygenases (Where R–H is the Substrate)

Similarly, many nonheme iron enzymes are used in biosynthetic pathways, and again, high-valent Fe­(IV)–oxo complexes are proposed as active intermediates in many of these reactions. For sMMO, catalysis depends on a nonheme diiron center instead of the more familiar Fe-porphyrin cofactor. The hydroxylase component of the sMMO enzyme, sMMOH, belongs to a family of diiron enzymes in which two His and four Asp/Glu residues are bound to the diiron active site (see Figure 4, left).^21,42,43^ The high-valent diiron oxidant for methane hydroxylation generated at the sMMOH active site is formed in two key steps (see Figure 4, right).^44–48^ In step 1, O_2_ binding to the ferrous enzyme generates an O_2_-adduct called Intermediate P or Hperoxo, which is best described as a (μ-1,2−peroxo) diferric intermediate commonly observed in model complexes. In step 2, the O–O single bond of Intermediate P is cleaved with the help of a proton to generate the diiron­(IV) oxidant “Q” that is capable of cleaving the 104 kcal/mol C–H bond of methane. Early X-ray absorption studies of Intermediate Q in sMMOH suggested an unusually short Fe······Fe separation of 2.46 Å, supporting the proposal of a bis­(μ-oxo) diamond core with an Fe­(IV)_2_(μ-O)_2_ arrangement for Q.^49^ Subsequent high-energy resolution fluorescence-detected extended X-ray absorption fine structure (HRFD-EXAFS) studies by DeBeer and co-workers found no evidence for such a short Fe······Fe distance. Instead, they observed a longer Fe······Fe separation around 3.4 Å—more consistent with an open-core geometry featuring a terminal Fe=O unit (see Scheme 1, Figure 4 and section 1.3.2.4).^50^

**Figure 4:**
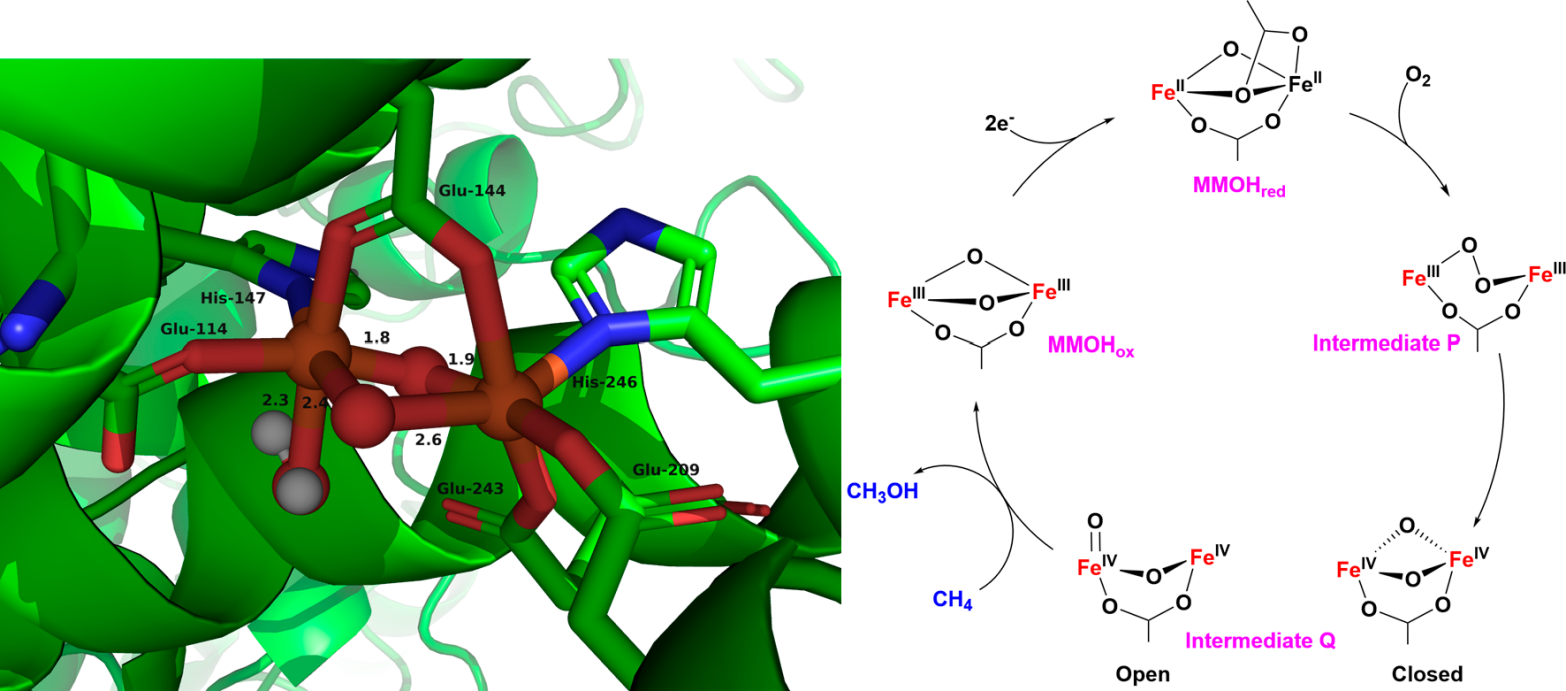
Left: The oxidized active site of soluble methane monooxygenase (sMMO) from *Methylococcus capsulatus* (PDB: 1MTY).^51^ Right: Proposed catalytic cycle for sMMO.

On the other hand, resonance rRaman (rRaman) work by Lipscomb, Proshlyakov, and co-workers identified a vibrational band at 690 cm^–1^ (shifted by 36 cm^–1^ upon ^18^O substitution), a feature characteristic of M_2_(μ-O)_2_ cores in synthetic models (see section 1.3.2.2).^52,53^ This result has fueled the ongoing debate about the exact geometric structure of Intermediate Q, between the EXAFS-derived open-core assignment and the rRaman-supported closed-core interpretation. Most recently, Solomon and co-workers reported a nuclear resonance vibrational spectroscopy (NRVS) study on Intermediate Q (see section 1.3.2.2).^54^ The vibrational fingerprint obtained from these studies was interpreted as supporting a closed, diamond-core description of the intermediate. Density functional theory (DFT) analysis of these data favors an Fe­(IV)_2_(μ-O)_2_ core with an Fe······Fe distance more in line with synthetic models (∼2.7 Å).^52,55^ Further investigations are required to resolve the exact geometric structure of Intermediate Q.

Rieske oxygenases (ROs) are a very large family of nonheme iron-containing enzymes that catalyze a remarkably range of reactions, including hydroxylation, halogenation, desaturation, epoxidation, *cis*-dihydroxylation, and aromatic ring cleavage reactions. Given this versatility, ROs are the nonheme equivalent of Cyt P450s in the domain of heme enzymes.^57–61^ In particular, Rieske-type iron-dependent dioxygenases (RDOs) catalyze the unique incorporation of both atoms of molecular oxygen into arenes, producing *cis*-dihydrodiol compounds using NADH and O_2_ as cosubstrates. Notable examples include Benzene Dioxygenase, Phthalate Dioxygenase, Toluene Dioxygenase, and Naphthalene 1,2-Dioxygenase (see Figure 5, left, for a picture of the O_2_-bound active site).^57,62,63^ These enzymes initiate the breakdown of aromatic compounds in soil bacteria, subsequently forming catechols that are further cleaved by intra- or extradiol dioxygenases.^64^ Furthermore, Rieske dioxygenases demonstrate a high degree of stereo- and enantioselectivity, capabilities which are difficult to replicate by synthetic catalysts. ROs were first identified as enzymes involved in the degradation of aromatic compounds by *Pseudomonas putida*.^64,65^ ROs are also involved in the biosynthesis of a large variety of natural products.^61^ For example, Challis and co-workers found that the enzymes RedG and McpG catalyze the regio and stereodivergent oxidative carbocyclization reactions in the biosynthesis of streptorubin B and metacycloprodigiosin, respectively.^66^

**Figure 5:**
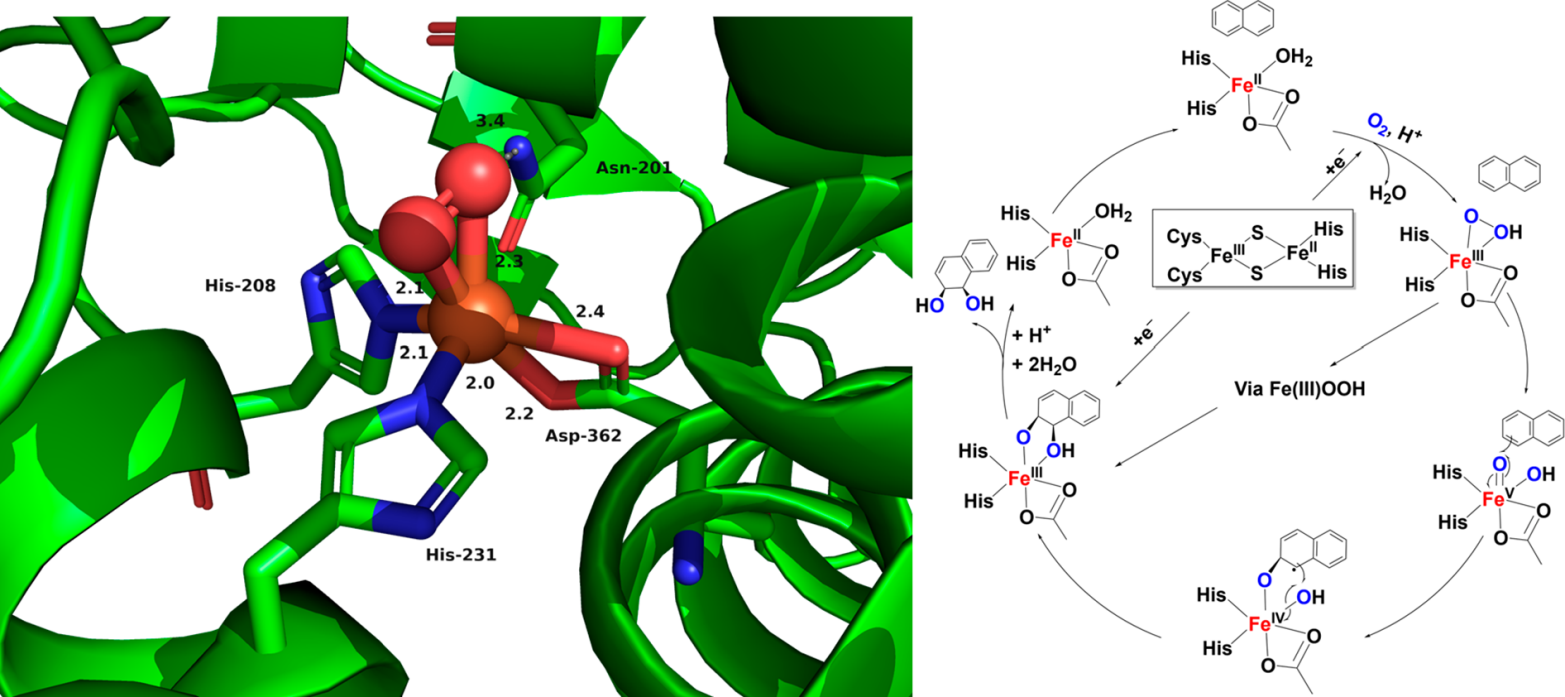
Left: O_2_-bound active site of naphthalene 1,2-dioxygenase (NDO) from *Pseudomonas putida* (PDB: 1O7M).^56^ Right: Proposed catalytic mechanism for NDO. Adapted from ref 57. Copyright 2013 American Chemical Society.

Compared to the extensively studied Cyt P450 enzymes, our understanding of the catalytic mechanism of Rieske dioxygenases has remained more limited.^67–70^ Available mechanistic evidence suggests that, following formation of an Fe­(III)–(hydro)­peroxo intermediate, the reaction may proceed via one of two pathways (see Figure 5, right). One possible mechanism involves direct attack of this intermediate on the substrate, thought to occur via nucleophilic attack on the arene by the coordinated hydroperoxo unit, followed by homolytic cleavage of the O–O bond to generate an Fe­(IV)–oxo species that then further oxidizes the substrate. The other possibility occurs via heterolytic cleavage of the O–O bond, producing a high-valent Fe­(V)­(O)­(OH) complex that then performs the substrate oxidation. In early 2000, Que and co-workers reported the involvement of a Fe­(III)–OOH intermediate in *cis*-dihydroxylation reactions with H_2_O_2_, with products characterized by mass spectroscopy (MS). Isotope-labeling experiments revealed that ^18^O incorporation in both the epoxide and the *cis*-diol products from H_2_^18^O and H_2_^18^O_2_ implicate a *cis*-H^18^O–Fe­(V)–oxo species derived from O–O bond heterolysis in the reaction.^71–73^ Only partial incorporation of ^18^O from water was observed for alcohol and epoxide products, but *cis*-diol products showed nearly full incorporation of one ^18^O atom. The extent of ^18^O-labeling increased with the concentration of H_2_^18^O and displayed saturation behavior, suggesting a water-binding pre-equilibrium that incorporates the ^18^O label from water into the metal-based intermediate prior to substrate oxidation. Based on these observations, initial formation of an Fe­(III)–OOH intermediate was proposed which then, in a water-assisted decay, affords a *cis*-H^18^O–Fe^V^=O species, followed by reversible oxo-hydroxo tautomerization.^73^ Costas and co-workers were the first to directly observe a Fe­(V)­(O)­(OH) species via variable-temperature mass spectrometry (VT-MS), and the reactions of this complex with C–H and C=C bonds were studied.^74^ The Fe­(V)­(O)­(OH) species was found to be a reactive oxidant that is capable of *cis*-dihydroxylation of alkenes. Studies on TMC- and N4Py-based model complexes indicate that a high-spin Fe­(III)–OOH species is sufficiently electrophilic to react directly with naphthalene.^74,75^ Later, Lipscomb, Solomon, and co-workers studied the peroxide shunt pathway for Benzoate 1,2-Dioxygenase using NRVS. They found separate mechanisms for the native O_2_ and peroxide shunt reactions in the RDOs and demonstrated the high reactivity of Fe(III)−superoxo relative to Fe­(III)–OOH species in catalysis.^76^ Very recently, Wang, Lehnert, Nam and co-workers have studied a series of TMC ligand frameworks to explore the effect of ring size on the reactivity of synthetic mononuclear nonheme side-on iron­(III)−peroxo complexes; i.e., [Fe^III^(12-TMC)­(O_2_)]^+^, [Fe^III^(13-TMC)­(O_2_)]^+^, and [Fe^III^(14-TMC)­(O_2_)]^+^, which model the corresponding Fe­(III)−peroxo intermediate in RDOs.^77^ These studies show that [Fe^III^(12-TMC)­(O_2_)]^+^ is a suitable catalyst for arene *cis*-dihydroxylation. Taken together, these results suggest that formation of an Fe­(V)­(O)­(OH) intermediate in the catalytic cycle of RDOs is not necessary for alkene *cis*-dihydroxylation. The question of whether a Fe­(II)/Fe­(III)−superoxo, Fe­(III)–OOH, side-on Fe­(III)−peroxo, or Fe­(V)­(O)­(OH) species in these enzymes is the critical intermediate that attacks the substrate as the active oxidant remains open. For Rieske monooxygenases, the Fe­(II)−superoxo adduct has also been suggested as a suitable intermediate in C–H bond activation reactions by Lipscomb and co-workers.^78^

Fe­(II)- and α-KG-dependent dioxygenases facilitate the hydroxylation of strong C–H bonds across various substrates, by coupling the reductive activation of dioxygen with the oxidative decarboxylation of α-KG. The diverse range of oxidative transformations that these enzymes catalyze is enabled by the fine-tuning of a highly conserved mononuclear nonheme iron cofactor, which is coordinated by as few as two protein ligands, leaving up to four coordination sites available for substrate and α-KG binding in the binary and tertiary complexes (see Scheme 3).^79^ In the most common arrangement, three protein ligands—two histidines and either an aspartate or glutamate—form what is known as the “facial triad,” occupying one face of an octahedral coordination site and leaving the three positions on the opposite face available for cofactor, substrate, and oxygen binding.^79^ The binding of α-KG to the iron­(II) center by replacing two water molecules in the binary complex is followed by binding of the substrate in the vicinity of the Fe­(II) center. The presence of the substrate leads to the dissociation of the third water molecule to generate the five-coordinate Fe­(II) center in the quaternary complex, which is then ready for the next step of catalysis, dioxygen binding to the Fe­(II) center (see Scheme 3). Various intermediates following O_2_ binding have been proposed in the reaction mechanisms of these enzymes,^79^ in particular Fe-coordinated superoxo complexes with an intact O–O bond ([Fe–O_2_]^2+/3+^) and high-valent Fe­(IV)–oxo intermediates. The latter intermediate was first captured through pioneering work by Krebs and Bollinger in Taurine Dioxygenase, and termed Intermediate *J*.^24^ The Fe­(IV)–oxo intermediate is particularly significant, as it is thought to be the catalytically competent species that initiates substrate oxidation (see Scheme 3). In most cases, substrate activation occurs when the Fe­(IV)–oxo intermediate abstracts a hydrogen atom from the target C–H bond, generating a substrate radical and an Fe­(III)–OH complex. A process known as “oxygen rebound” (see section 2.4.3), originally proposed for heme enzymes,^14,80^ then follows. This involves the recombination of the coordinated hydroxyl radical equivalent with the substrate radical, yielding a hydroxylated product and a coordinatively unsaturated Fe­(II) center. Quantum mechanics/molecular mechanics (QM/MM) studies have provided additional support for this mechanism.^81–97^ Beyond hydroxylation, various alternative outcomes have been observed following hydrogen atom abstraction (HAA) by the Fe­(IV)–oxo intermediate. These include the formal transfer of a ligand from the Fe center to the substrate radical, such as the transfer of a halogen atom in α-KG-dependent halogenases,^98^ and the transfer of a thiyl group in Isopenicillin N Synthase (IPNS).^99^ Other important processes that Fe­(II)- and α-KG-dependent dioxygenases are involved in^100^ include fatty acid metabolism,^101,102^ oxygen sensing,^103,104^ histone demethylation,^99,105^ DNA and RNA repair,^106,107^ and the formation of ethylene from α-KG in the ethylene-forming enzymes from plants.^98,108–110^

**Scheme 3:**
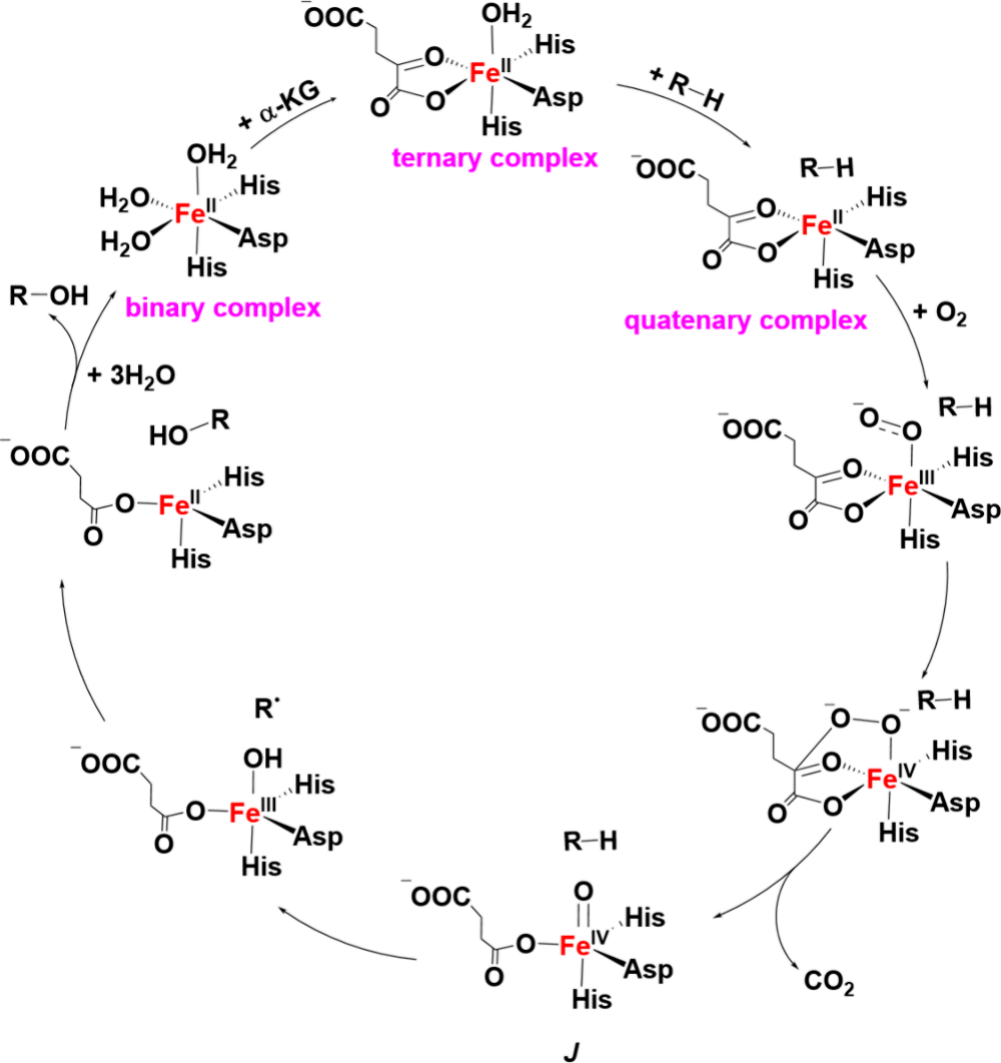
Catalytic Cycle of the Fe­(II)- and α-KG-Dependent Non-Heme Iron Dioxygenases

Pterin-dependent nonheme iron enzymes help make neurotransmitters by adding an −OH group to aromatic amino acids (like phenylalanine, tyrosine, or tryptophan).^112^ In the first step of the mechanism, the amino acid substrate binds in the active site, and initial studies concluded that the pterin cofactor binds nearby in the active site as well (see Scheme 4, top left), but not directly to the Fe­(II).^113–115^ In a recent study, Solomon and co-workers challenged this notion: here, magnetic circular dichroism (MCD) studies revealed that the 330 nm band, which appears after pterin and tryptophan are added to the ferrous form of Tryptophan Hydroxylase (TPH), belongs to the paramagnetic iron complex. This 330 nm absorption feature must therefore belong to a charge transfer (CT) transition between Fe­(II) and either the pterin or tryptophan. Both rRaman data and DFT calculations provide strong evidence for direct coordination of the pterin carbonyl to the iron center in the ternary complex. Upon reacting this ternary complex with O_2_, a new absorption band emerges at 442 nm, within the first 175 ms of the reaction, which decays within 2s and which was attributed to an Fe­(II)-peroxy-pterin intermediate. Correspondingly, this 442 nm absorption band was assigned to a metal-to-pterin charge-transfer transition, supported by the absence of a resonance-enhanced O–O stretching mode in rRaman spectroscopy and the detection of intrapterin vibrational modes in the rRaman spectrum. Based on DFT calculations, the most intense vibration at 489 cm^–1^ observed in the rRaman spectrum was assigned to the Fe–O_carbonyl_ stretch, while a band at 451 cm^–1^ was assigned to the Fe–O_peroxy_ stretch. These vibrational features indicate the simultaneous coordination of the pterin carbonyl group and the peroxide to the iron center, which, together, define the geometric structure of the peroxy–Fe­(II) intermediate (see Scheme 4, top right). Computationally, the authors found a 14 kcal/mol lower barrier for the reaction of O_2_ with the iron center in the presence of the pterin compared to the same reaction without the pterin, which corresponds to a 10^10^-fold increase in reaction rate. From Mössbauer spectroscopy, the authors found a new doublet with the isomer shift of 1.25 mm/s after the reaction with O_2_, which is similar to that of the initial Fe­(II) complex (δ = 1.29 mm/s). The smaller quadrupole splitting (Δ*E*_Q_ = 2.80 mm/s) for the former species, however, suggests an oxygenated complex in the Fe­(II) oxidation state, which further indicates that pterin oxidation happens first. The direct coordination of the pterin’s carbonyl to the iron center facilitates O–O bond cleavage and the generation of a highly reactive Fe­(IV)–oxo species in the next step of catalysis. This intermediate then attacks the aromatic ring of the substrate to abstract a hydrogen atom and initiates its hydroxylation. In the process, the pterin is oxidized and later recycled. After completion of the reaction, the iron returns to the Fe^2+^ state, completing the cycle.^111^

**Scheme 4:**
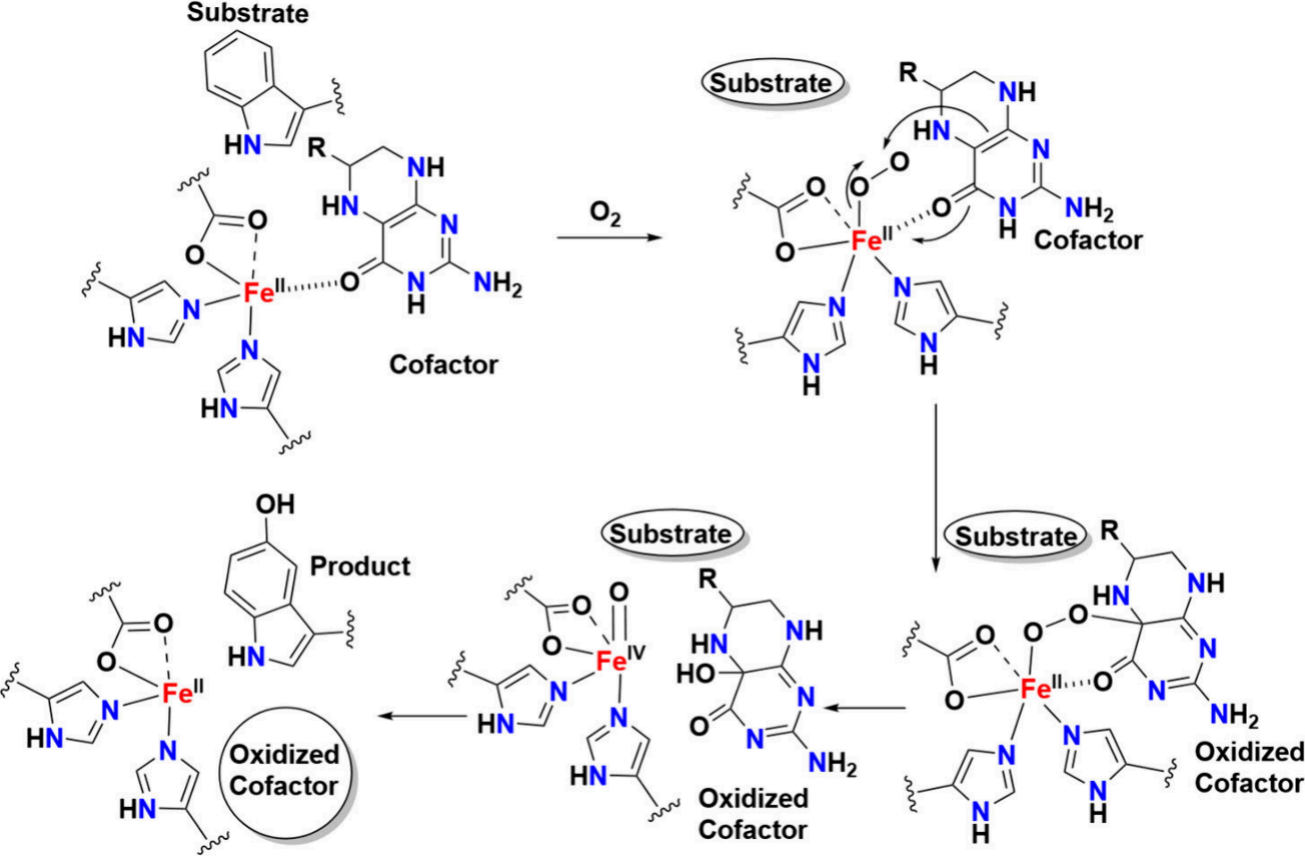
Proposed Mechanism of Pterin-Dependent Non-Heme Iron Enzymes (In This Example, The Substrate Is Tryptophan)^a^

a Adapted from ref 111. Copyright 2021 National Academy of Science.

The enzyme NOV1 from *Novosphingobium aromaticivorans* is a stilbene cleaving oxygenase (SCO) responsible for the oxidative cleavage of the central double bond of stilbenes, forming two phenolic aldehydes. In the active site, the iron center is unusual, as it is coordinated by four histidines (see Scheme 5). SCOs belong to the carotenoid cleavage oxygenase (CCO) family, nonheme, Fe­(II)-dependent enzymes that catalyze oxidative cleavage of β-carotene and apocarotenoids.^117–119^ In 2005, Schulz and co-workers first reported the crystal structure of the apocarotenoid-15,15′-oxygenase (ACO) from *Synechocystis* at 2.4 resolution.^117^ NOV1 and ACO superimpose in the β-sheet domain, substantial structural variations occur in the cap region. Very recently, McAndrew, Adams and co-workers reported crystal structures of NOV1 with O_2_ bound to the iron center, and of the Fe–O_2_ adduct in the presence of the substrate, resveratrol, or the product, vanillin.^116^ In all three of these structures, dioxygen is bound in a side-on fashion. Based on EPR studies, the authors propose that the Fe–O_2_ species corresponds to a ferric-superoxide intermediate, which subsequently reacts with the substrate.

**Scheme 5:**
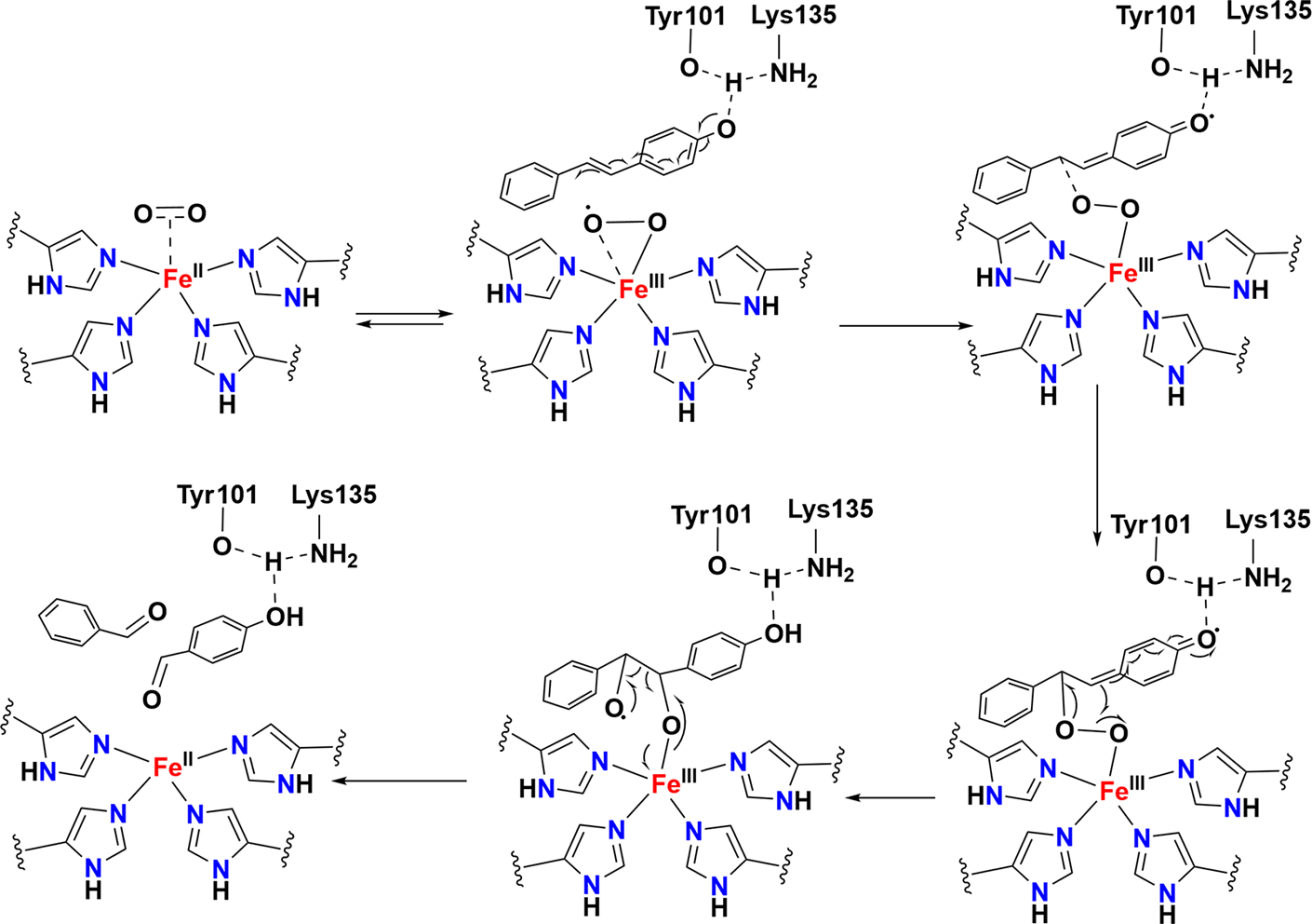
Proposed Reaction Mechanism for NOV1 ^116^

In addition to these early- to mid-transition metal–oxo complexes, evidence is mounting that several monocopper oxygenases function via corresponding, formally Cu­(III)–oxo intermediates.^121,122^ For example, methane hydroxylation by particulate methane monooxygenase (pMMO), which converts methane into methanol in methanotroph metabolism, is proposed to be mediated by a monocopper site as reported by Rosenzweig, Hoffman and co-workers.^123^ Whereas the relevant oxygen intermediates in Cyt P450s and many nonheme iron enzymes are quite well understood (see above), the Cu–O intermediate(s) of pMMO remain unresolved owing to the complexity of the enzymatic system. What is known is that the active site corresponds to one of the multiple conserved monocopper sites of these enzymes, labeled Cu_B_, Cu_C_, and Cu_D_, with most researchers favoring either Cu_C_ or Cu_D_, which was recently discovered by Rosenzweig and co-workers, as the site of methane hydroxylation.^123–128^ DFT calculations suggest that the critical intermediate for C–H bond activation of methane in pMMO is a formally Cu­(III)–oxo species.^129^ A recent QM/MM study has suggested that a quinol cosubstrate may directly provide an additional oxidizing equivalent at the Cu_C_ site (see Scheme 1). Thus, the active-site may be best formulated as a physical Cu­(II)–oxyl/quinyl species (i.e., a formally Cu­(IV)–oxo species for the purpose of electron counting).^129,130^

Lytic polysaccharide monooxygenases (LPMOs) are copper-dependent enzymes that belong to the auxiliary activity (AA) superfamily.^131–134^ These powerful monocopper enzymes are capable of activating strong C–H bonds, although their exact mechanism remains largely unclear. In LPMOs, the copper center is coordinated by an N-terminal histidine residue and a further histidine side chain in an arrangement known as the “histidine brace”, as shown in Figure 6. LPMOs enhance the breakdown of polysaccharides by oxidizing the glycosidic bonds that connect the individual sugar units. Mechanistically, LPMOs activate O_2_ to cleave polysaccharide glycosidic bonds by hydroxylating either the C1–H or C4–H positions.^133,135–142^ Although the exact mechanism of LPMOs is still unknown, it is often proposed to resemble that of other monooxygenases, such as Cyt P450 enzymes. Here, the reduced Cu­(I) center is thought to react with O_2_ to yield rapid oxidation to Cu­(II) with the presumed formation of a Cu­(II)–superoxide complex.^141^ After this step, multiple mechanistic pathways are possible. Depending on its redox and protonation state, the reactive oxygen species could be a superoxide, a hydroperoxyl, or an oxyl species, each of which has been proposed as the key agent responsible for abstracting a hydrogen atom from the glycosidic carbon of the polysaccharide substrate.^142^ In 2012, Cate and co-workers reported the crystal structures of Cu­(II)–O_2_ intermediates in *Neurospora crassa* PMO-2 and PMO-3.^140^ In 2017, Meilleur and co-workers reported a similar X-ray crystal structure for the Cu­(II)–O_2_ intermediate formed in the absence of substrate for *Neurospora crassa* PMO-2; additionally, they also obtained the structure of dioxygen in the active site pocket, prior to coordination to the copper center.^143^ Marletta and co-workers reported on the role of the second coordination sphere (SCS) for the stabilization of a copper dioxygen intermediate in *Mt* PMO3*, a C1-oxidizing PMO from the ascomycete fungus *Myceliophthora thermophila*.^144^ The authors concluded that histidine and glutamine residues play an important role in proton transfer, and that hydrogen bonds assist in O–O bond cleavage to generate a highly reactive Cu­(II)–oxyl intermediate. In 2022, Meilleur, Agarwal, and co-workers trapped both Cu­(II)–O_2_ and Cu­(II)–OOH intermediates at the copper active site of *Nc* LPMO9D, and characterized these species using neutron diffraction protein crystallography.^145^

**Figure 6:**
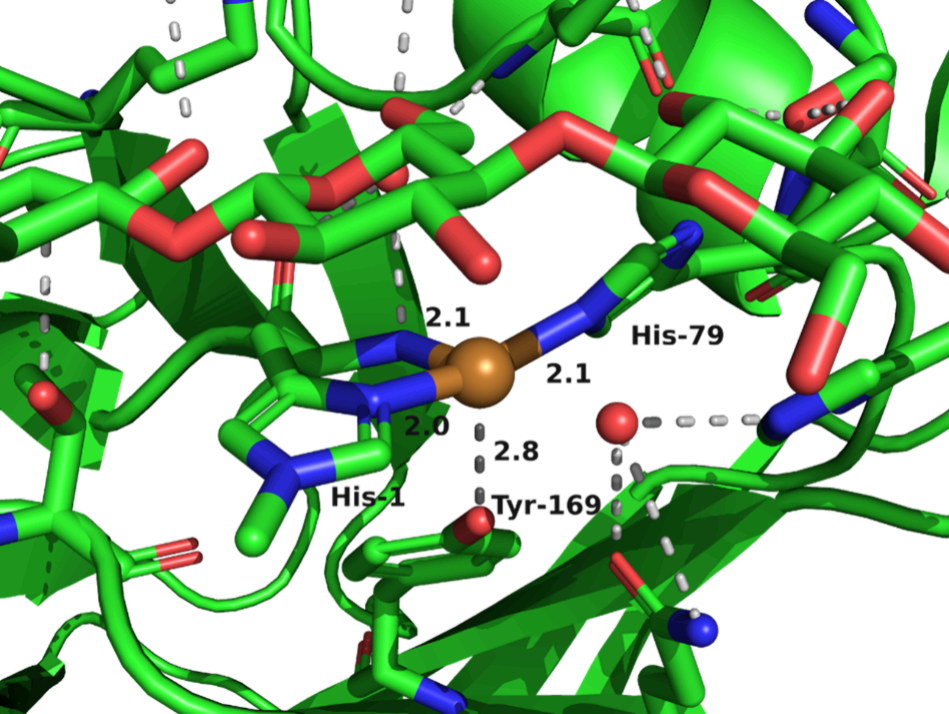
Active site structure of LPMO from *Achaetomiella virescens* with bound β-cellohexaose substrate (PDB: 6YDE).^120^

In 2017, Bissaro et al. demonstrated that besides using O_2_ as an oxidant, LPMOs can also employ H_2_O_2_ as a cosubstrate to perform the oxygenation of polysaccharides, producing the same hydroxylated products as in the O_2_-driven reaction.^146^ This initial oxidation reaction subsequently results in cleavage of the polysaccharide substrate. Davies, Walton and co-workers reported that the reaction of an AA9 LPMO with H_2_O_2_ at higher pH in the absence of substrate yields a highly stable, purple Cu­(II)–tyrosyl radical species.^147^ Spectroscopic characterization (UV–vis, circular dichroism (CD), variable-temperature/variable-field MCD (VTVH MCD), rRaman and electron paramagnetic resonance (EPR) spectroscopy), supported by X-ray absorption spectroscopy (XAS), MS, and DFT calculations, revealed the formation of a ferromagnetically coupled (*S* = 1) Cu­(II)–tyrosyl radical species, which has been proposed to play a role in the catalytic cycle of LPMOs.^148^ In a recent study, Walton, Heyes, Green and co-workers investigated transient intermediates in LPMOs using H_2_O_2_ and *meta*-chloroperbenzoic acid (*m*-CPBA) oxidants. Stopped-flow UV–Vis studies of three Cu­(I)–LPMOs with *m*-CPBA revealed a previously uncharacterized intermediate, called Int 1, formed in the absence of substrate. They suggest that Int 1 is a (*S* = 0) Cu­(II)-(histidyl radical) complex based on EPR and high-energy resolution fluorescence detection X-ray absorption spectroscopy (HRFD-XAS), and DFT calculations. This result aligns with a recently proposed mechanism that explains the oxidative damage of histidine 1 during uncoupled turnover of LPMOs.^146,149^ This species is quenched via a net hydrogen atom transfer from a nearby tyrosine, resulting in the formation of the previously obtained *S* = 1 ferromagnetically coupled Cu­(II)−tyrosyl radical complex, effectively returning the enzyme to its Cu­(II) resting state.^147^

A key question about LPMO is still whether the enzyme uses O_2_ or H_2_O_2_ as the natural cosubstrate (see Scheme 6), with theoretical studies supporting both possibilities.^150^ Experimentally, it was found that substrate oxidation is much faster when H_2_O_2_ is used compared to O_2_.^151,152^ In recent work, DeBeer, Eijsink, Sørlie and co-workers explored the role of a conserved glutamine or glutamate residue located in the SCS of LPMOs. Specifically, the type and proximity of these residues to the copper center influence LPMO activity and copper reactivity. It was further determined that the presence of glutamate or aspartate near the copper center lowers its reduction potential and decreases the ratio of reduction to reoxidation rates by 500-fold.^153^ Very recently, Hedegård and co-workers investigated the mechanism of LPMOs (specifically for *Ls* AA9) using QM/MM calculations, and proposed that the H_2_O_2_-driven pathway is more feasible than the O_2_-driven pathway,^150^ in agreement with previous investigations.^154,155^ Forming a reactive Cu–oxyl species through a caged hydroxyl radical and subsequent substrate oxidation is energetically downhill and kinetically feasible compared to the O_2_-driven pathway. Although not experimentally characterized, this intermediate may be either a triplet (*S* = 1) Cu­(II)–oxyl, [Cu–O]^+^, or a singlet (*S* = 0) Cu­(III)–hydroxide, [Cu–OH]^2+.^ Another mechanistic possibility is that the Cu center mediates Fenton-type chemistry with H_2_O_2_, and that the hydroxyl radicals generated via enzyme-catalyzed homolytic cleavage of the O–O bond of H_2_O_2_ play a major role in substrate oxidation. This possibility is supported by model complex studies.^156,157^

**Scheme 6:**
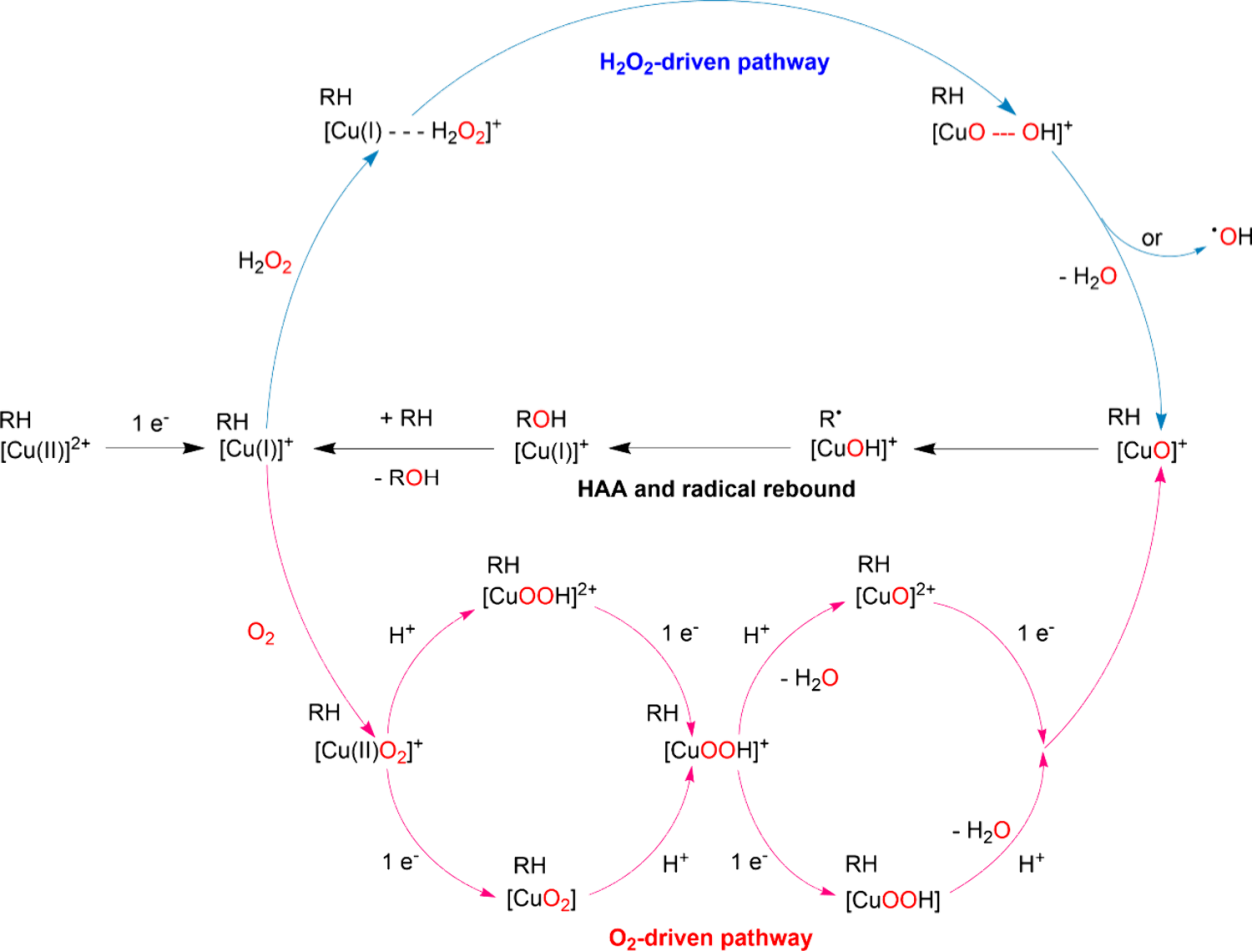
Proposed Reaction Mechanisms for LPMO with O_2_ or H_2_O_2_ as Co-substrates^a^

a Oxidation states are shown only for the Cu ions in moieties where they are unambiguous. Adapted from reference 120. Copyright 2024 American Chemical Society.

### 1.2 Chemical Transformations Catalyzed by Metal–Oxo Intermediates in Nature

Metal–oxo species play a crucial role in nature by facilitating various chemical transformations, particularly in enzymatic oxidation reactions. These highly reactive intermediates are commonly found in metalloenzymes such as Cyt P450s and nonheme iron (di)­oxygenases. In nature, these enzymes often activate molecular oxygen to perform the selective oxidation of organic substrates via high-valent metal–oxo intermediates that are able to mediate many reactions, in particular HAA and O atom transfer (OAT) chemistries, leading to substrate hydroxylation, epoxidation, and oxidative cleavage reactions.^10,40,158^ Figure 7 provides a chart that summarizes key enzymatic reactions mediated by metal–oxo intermediates in nature.

**Figure 7:**
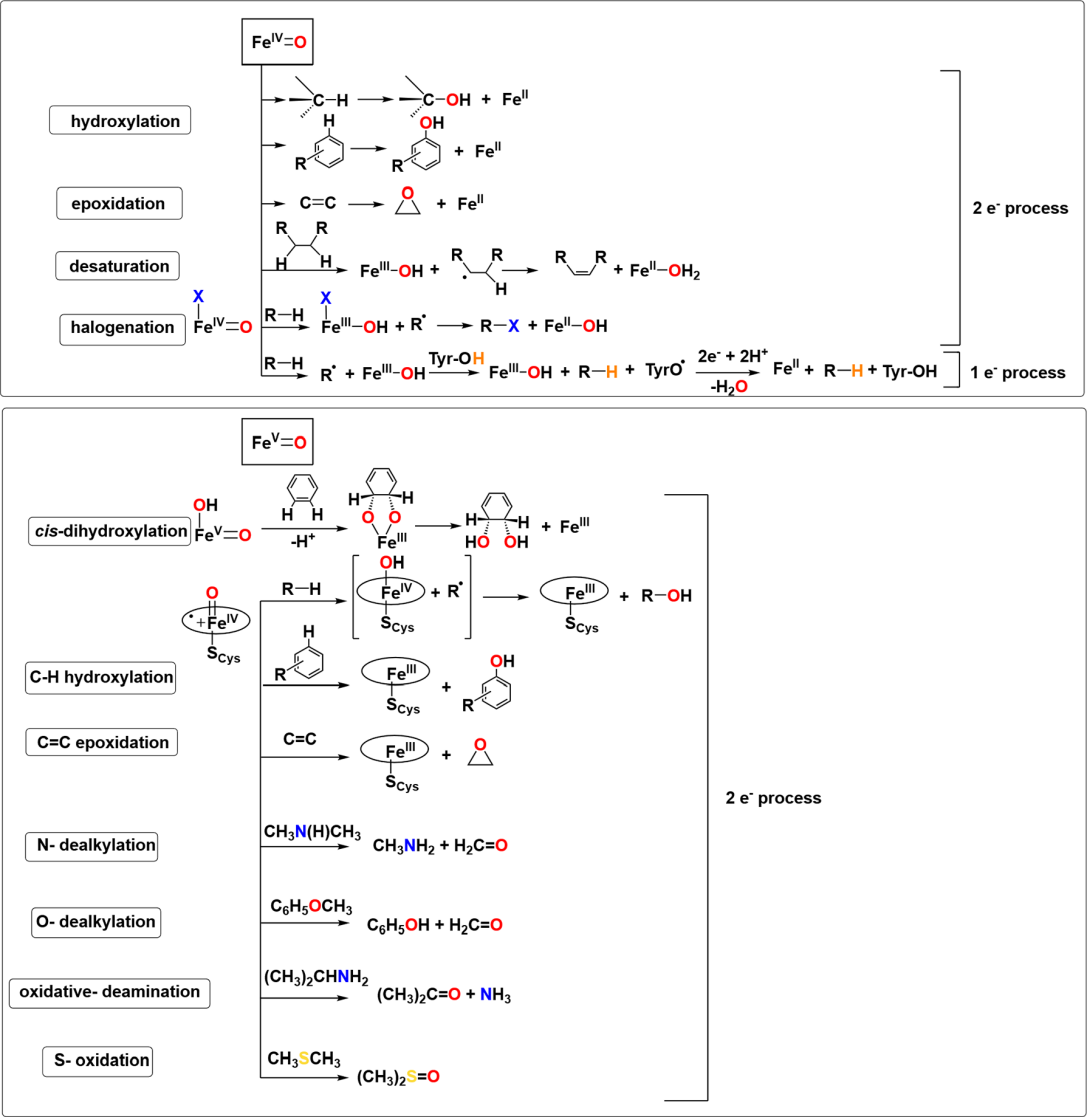
List of select reactions in biology that involve reactive high-valent metal–oxo intermediates.

In addition to HAA, considerable effort has been directed toward elucidating the role of high-valent metal–oxo species in both enzymatic and bioinspired OAT reactions.^68,159^ While epoxidation is commonly catalyzed by Cyt. P450s, examples among nonheme iron enzymes are far fewer. The enzymes AsqJ, DdaC and PenD, all α-KG-dependent oxygenases, perform C=C bond epoxidation on their respective native substrates.^160–162^ Beyond its hydroxylase activity, Thymine Hydroxylase was shown to catalyze epoxidation when provided with a substrate analogue bearing a vinyl group in place of the native methyl substituent.^163^ Beyond these transformations, metal–oxo intermediates are also involved in a number of other processes, such as electron-transfer reactions,^164^ water-exchange at the metal–oxo site,^165^ isomerization of the metal–oxo moiety,^166^ and potentially the “ferryl flip”. The latter process is proposed for several α-KG-dependent dioxygenases, such as Clavaminate Synthase^167^ and DNA-repairing AlkB oxygenases,^168^ and also for synthetic models.^169^ However, more recent computational studies suggest that the ferryl flip may not (always) be energetically favorable, whereas the alternative α-KG flip has a much lower energy barrier.^81^

### 1.3 General Comments about Model Complexes and Spectroscopic Methods to Study High-Valent Intermediates

#### 1.3.1 Timeline of Discovery of Metal–Oxo Intermediates from Enzymes to Model Complexes

Figure 8 shows a rough timeline of the discovery of metal–oxo and related intermediates in enzymes and model complexes.

**Figure 8:**
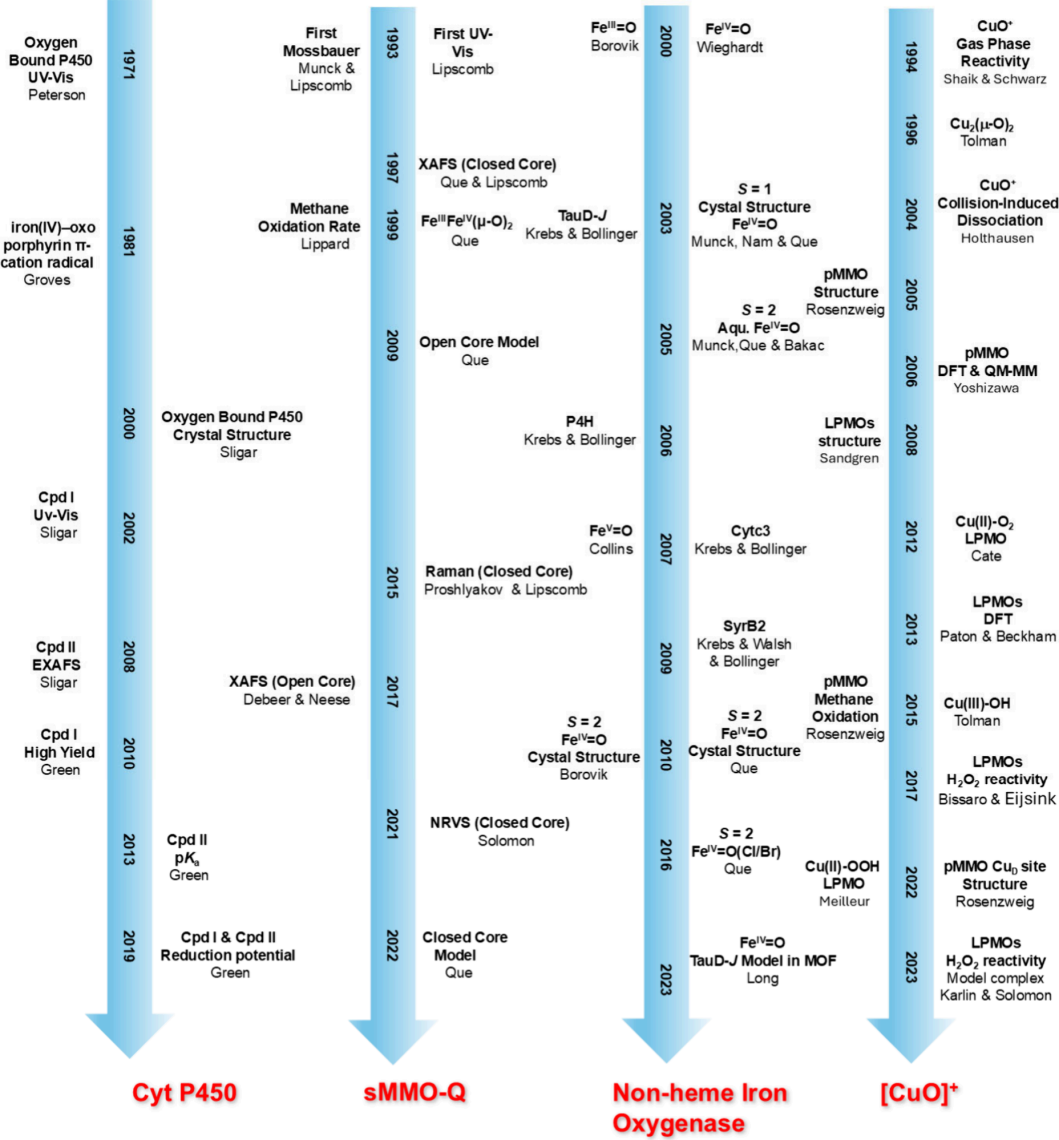
Timeline of the discovery of high-valent metal–oxo intermediates in metalloenzymes, key spectroscopic evidence, and the report of corresponding model complexes.

#### 1.3.2 Spectroscopic Methods for the Study of Metal–Oxo Intermediates and How These Results Relate to Their Electronic Structure

##### 1.3.2.1 UV–vis Absorption Spectroscopy and Kinetic Studies

UV–vis absorption spectroscopy^170^ is a powerful method for identifying and characterizing high-valent enzymatic intermediates. First of all, UV–vis spectroscopy is considered an initial tool for following the generation of reaction intermediates and quantifying their stability before performing other spectroscopy measurements. For this purpose, UV–vis spectroscopy is used in combination with the stopped-flow technique, which allows researchers to follow fast reactions down to about 1 ms (the limit of mixing two solutions), and to perform further kinetic analyses.^171,172^ This is exemplified by early efforts to follow formation of Cpd I in Cyt P450s. However, when this intermediate is generated from O_2_, electron transfer is rate limiting, and this intermediate, due to its instability, never builds up to any significant concentration. In the laboratory, an alternative approach for the generation of iron–oxo intermediates is the use of a “shunt reaction” (see Scheme 2), where the ferric resting state of a Cyt P450, for example, is reacted with a two-electron oxidant like hydrogen peroxide, O atom transfer agents, like iodosylbenzene (PhIO), or peracids.^173,174^ Green and co-workers successfully obtained Cpd I in high yield for the first time,^15^ by reacting a 20 mM ferric CYP119 (a thermophilic Cyt P450 from *Sulfolobus acidocaldarius*) enzyme solution at pH 7 with 40 mM *m*-CPBA. This method converts about 70% of the enzyme into Cpd I within 35 ms. The corresponding stopped-flow data show only two absorbing species—the ferric enzyme and Cpd I. The UV–vis absorption spectrum of Cpd I of CYP119 obtained in this way matches well with the previously reported spectrum by Kellner et al. for this species.^28^ The UV–vis spectrum of Cpd I shows the Soret band at 367 nm and Q bands at 610 and 690 nm, as shown in Figure 9.

**Figure 9:**
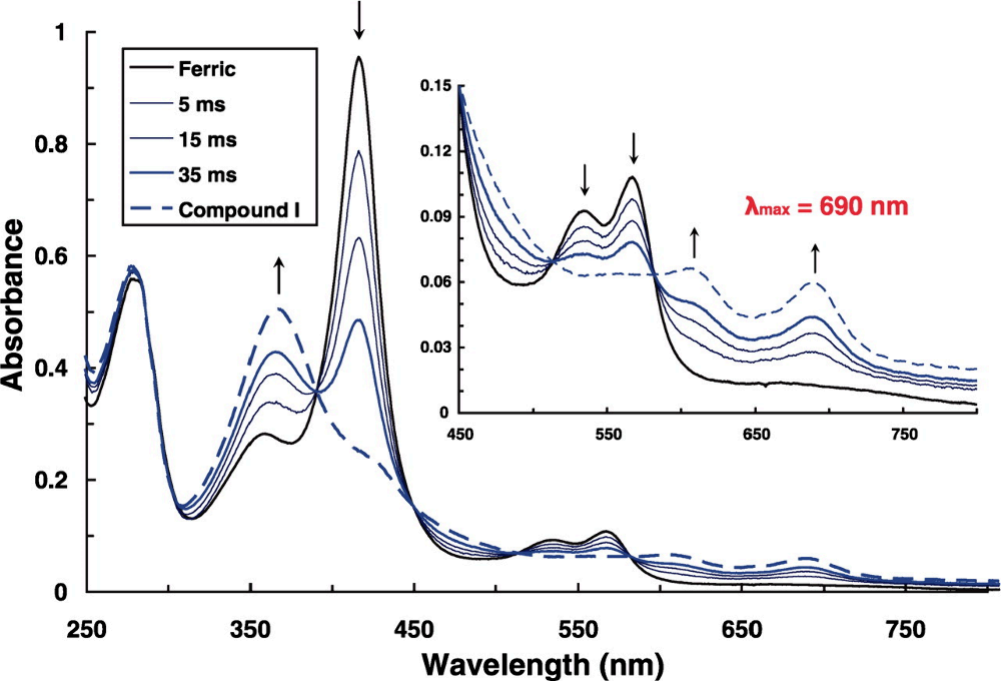
UV–vis absorption spectrum of ferric CYP119 (black) and spectral changes upon the addition of *m*-CPBA (blue, dashed) at different time points. Reproduced with permission from ref 15. Copyright 2010 AAAS.

The powerful Intermediate Q of the enzyme sMMO was first trapped by Lipscomb and co-workers.^46^ The rates of formation and decay of Q at 4 °C were measured at 430 nm using single wavelength stopped-flow spectroscopy. Among the intermediates detected in this investigation was a yellow species (λ_max_ = 330 and 430 nm), termed Intermediate Q. Substrates were found to have little effect on the rate of Intermediate Q formation, suggesting that the substrate first enters the reaction cycle after the formation of Q. On the other hand, the decay rate depends on both the concentration and the type of substrate present and was found to match the formation rate of the product bound in the active site. These observations suggest that Intermediate Q may be either the activated form of the enzyme that leads directly to substrate hydroxylation or an immediate precursor.^46^ Stopped-flow optical spectrophotometry at 4 °C was also used to analyze the kinetics of formation and decay of Intermediate Q. The reaction between Q and the natural substrate methane was first investigated directly with double-mixing stopped-flow spectroscopy by Lippard’s group. Table 1 shows data for methane and other hydrocarbon oxidation by Intermediate Q of sMMO.^175,176^ Interestingly, it was observed that propane reacts about ten times slower than ethane. Also, methane, which has a C–H bond strength of 104 kcal/mol, reacts more slowly than ethane, but faster than propane. Clearly, reactions with alkanes depend on more than just C–H bond strength.

**Table 1:** Reaction Order in Substrate and Second-Order Rate Constants for Substrate Reactions with Intermediate *Q* at 20 °C (Data Are Reported in ref 176)

substrate	reaction order in substrate	*k*(× 10^4^ M^–1^ s^–1^)
methane	1.10	2.9
ethane	0.73	3.2
propane	0.70	0.31
ethylene	0.90	6.1
propylene	0.83	1.2
acetylene	0.90	8.8

##### 1.3.2.2 Vibrational Spectroscopy (IR, rRaman, NRVS)

Vibrational spectroscopy techniques, including Infrared (IR), resonance Raman (rRaman), and Nuclear Resonance Vibrational Spectroscopy (NRVS), are powerful tools for detecting and characterizing high-valent enzymatic intermediates, providing detailed structural and electronic insights by probing vibrations within the active site.^177–181^ In particular, the determination of the M–O stretching vibration, confirmed by isotope labeling, provides a direct proof for the presence of a M–O unit. The M–O vibrational frequency provides a further measure for the strength of a metal–O bond (although one has to be careful here with regards to possible mode mixing).^180^ IR spectroscopy is generally not ideal for studying high-valent metal–oxo intermediates in enzymatic systems due to weak or broad M–O stretching bands, which typically appear in the 700–950 cm^–1^ range with low intensity. M–O stretching bands therefore overlap with other vibrational modes of the protein matrix, buffer components, solvents (in the case of model complexes), etc., making their assignment more challenging. In contrast, for synthetic, stable metal–oxo complexes, the M–O stretch has been successfully characterized using solid-state IR spectroscopy.^182^ Hence, given these limitations, alternative techniques such as rRaman spectroscopy and NRVS are better suited for studying the M–O stretching vibrations in high-valent metal–oxo intermediates.

rRaman spectroscopy is a highly effective technique for identifying M–O stretching vibrations in high-valent metal–oxo enzymatic intermediates. Unlike traditional IR spectroscopy, rRaman spectroscopy selectively enhances vibrational modes associated with electronic transitions, allowing for the detection of weak or overlapping vibrational signals.^183,184^ Here, excitation of a metal–oxo charge-transfer transition enhances the intensity of the M–O stretching mode, allowing for its detection in proteins and model complexes in solution. In heme systems, excitation of the Soret band has also been shown to enhance M–O stretching modes. Initially, rRaman studies focused on the oxy complexes of heme proteins. However, rRaman studies on the oxy complexes of Cyt P450s were hindered by their instability at ambient temperature (with a half-life of approximately 90 s at 298 K for CYP101 (Cyt P450cam)) and sensitivity to laser light. Using rRaman spectroscopy at cryogenic temperatures within a spinning optical cell, Bangcharoenpaurpong and colleagues successfully measured the O–O stretching vibration of the oxygenated Cyt P450cam enzyme, confirmed by isotope labeling, as reported in 1986.^185^ In the following, rRaman measurements revealed that the O–O and Fe–O bonds are slightly weaker in oxy-P450 (with corresponding O–O and Fe–O stretching vibrations of 1128–1140 and 537–541 cm^–1^, respectively)^185^ as compared to those seen in oxygenated myoglobin and hemoglobin (1148 and 572 cm^–1^, and 1136 and 554 cm^–1^, respectively).^186,187^ Unfortunately, to the best of our knowledge, the Fe–O stretch in Cpd I in Cyt P450s has not been reported yet. However, rRaman studies of both native and mesoheme-reconstituted HRP by Kincaid and co-workers revealed that authentic Cpd I spectra are accessible using ∼ 350 nm UV excitation, whereas 406.7 nm visible light excitation produces a Cpd II–like photoproduct that contaminates the signal. Isotopic labeling with H_2_^18^O_2_ unambiguously allowed for the assignment of the Fe–O stretch of HRP Cpd I at ∼790 cm^–1^ (shifting by ∼25 cm^–1^ to lower frequency upon ^18^O labeling), confirming the presence of an Fe­(IV)–oxo group in the native and the mesoheme-reconstituted proteins and a ^2^A_1u_-like ground state for the porphyrin radical in Cpd I, unaffected by the porphyrin substitution.^188,189^ Green and co-workers studied chloroperoxidase (CPO) Cpd II using rRaman spectroscopy; they found that the Fe–O stretch of this species occurs around 565 cm^–1^, which shifts 22 cm^–1^ to lower frequency upon ^18^O labeling, and down by 12 cm^–1^ in the presence of D_2_O. These results demonstrate that CPO Cpd II is an Fe­(IV)–OH species.^190^

In 2004, a rRaman study by Proshlyakov, Hausinger and co-workers on the Intermediate *J* of TauD using a continuous flow apparatus was reported. In these experiments, two reactants are mixed continuously and passed in a capillary through the excitation laser beam. The authors observed vibrations corresponding to the oxygenated intermediates of TauD, using transient rRaman spectroscopy. The most prominent vibration was observed at 821 cm^–1^ for ^16^O, which shifts to 787 cm^–1^ when prepared with the ^18^O isotope, corresponding to the Fe–O stretch of Intermediate *J*. Another oxygen-sensitive feature was observed around 1157/1095 cm^–1^, probably the O–O stretch of the Fe­(III)−superoxo intermediate, but further analysis was not possible due to solvent interference (with a solvent band at 1089 cm^–1^).^191^

In 2015, a key rRaman study on sMMO Intermediate Q was reported by Lipscomb, Proshlyakov and co-workers.^53^ Here, time-resolved rRaman spectroscopy was used, which allows for the identification of reaction intermediates by analyzing their distinct vibrational signatures through extensive signal averaging, facilitating the detection of short-lived species. Two distinct vibrational features were observed at Δ*t* ≈ 3 s in the absence of substrate: a major feature at 690 cm^–1^ and a weaker feature at 556 cm^–1^, which disappeared at a longer delay time (Δ*t* ≈ 30 s). Upon addition of the substrate, the major feature at 690 cm^–1^ disappeared. The correlation between Intermediate Q and the 690 cm^–1^ feature was observed when CD_4_ was used as the substrate, which, due to the large kinetic isotope effect (KIE) of the reaction of Intermediate Q with CH_4_, slows down the reaction considerably. The authors found that the loss of intensity of the 690 cm^–1^ vibration and the increase of the 556 cm^–1^ feature is smaller in the presence of CD_4_. The observed frequency at 690 cm^–1^ (see Figure 10) is much smaller than that of the Fe–O stretch of terminal Fe­(IV)–oxo model complexes,^191^ but similar to that of corresponding closed core model complexes.^192,193^ These data provide support for the formulation of Intermediate Q as a Fe_2_(μ-O)_2_ diamond core structure (see Figure 10).

**Figure 10:**
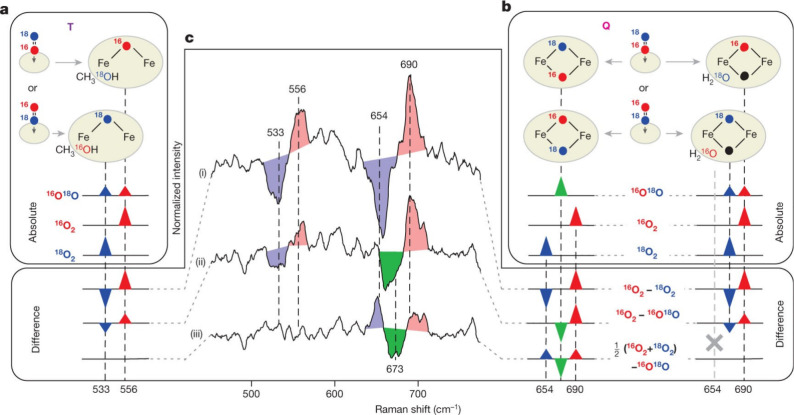
rRaman data for sMMO Intermediate Q using the ^16^O^18^O mixed oxygen isotope. (a,b) Asymmetrically labeled ^16^O^18^O can initially bind in two equiprobable orientations, to yield an even mixture of two isotopomers in T or Q, exhibiting characteristic vibrations (a,b, bottom). Since the two isotopomers of T (a) and those of singly labeled Q (b, right) have identical composition as corresponding ^16^O_2_ and ^18^O_2_ derivatives, they will display both vibrations simultaneously with half the intensity. Doubly labeled Q (b, left) will be different from both symmetrically labeled derivatives and thus, will exhibit a new vibration (green), as illustrated by isotope difference spectra. (c) The ^16^O_2_–^16^O^18^O difference (ii) in the singly labeled cluster (a,b, right) will appear as the ^16^O_2_–^18^O_2_ difference with reduced intensity. The ^16^O^18^O derivative will be identical to the average of symmetrical isotopomers, yielding no signal in the bottom traces in (c), as observed experimentally for T. The new frequency in doubly labeled Q should appear in both difference spectra, and can, indeed, be seen in experimental data at 673 cm^–1^ (see (ii) and (iii) in the middle). Reproduced with permission from ref 53. Copyright 2015 Nature.

For high-valent iron–oxo intermediates, another technique that can be used for the detection of Fe–O stretching vibrations is NRVS. NRVS is a synchrotron-based technique that is based on the resonant excitation of the Mössbauer nuclear transition of ^57^Fe, and the detection of the nuclear inelastic scattering. Here, inelastic scattering is derived for vibrations that contain motion of the iron nucleus, originating from quantized recoil.^194,195^ In the vibrational density of states (VDOS) representation of the NRVS intensity, the integral intensity of a vibrational band scales with the square of the amount of iron motion (amplitude) in the corresponding normal mode. Hence, this technique is very well suited for the detection of iron-ligand stretching and bending vibrations that contain a lot of iron motion. Using single crystals, directional information about the iron displacement can be obtained as well, further assisting in vibrational assignments.^196^

Solomon, Krebs, Bollinger and co-workers used NRVS to study the structure of the reactive Fe­(IV)–oxo intermediate in a nonheme iron enzyme called SyrB2,^197^ which comes from the bacterium *Pseudomonas syringae pv syringae*, and which catalyzes the halogenation of the native substrate l-threonine (l-Thr), and the hydroxylation of non-native substrates.^198,199^ This Fe­(IV)–oxo intermediate first reacts by HAA from the methyl group of l-Thr. It then either halogenates the natural substrate or hydroxylates non-natural ones. Previously, it was predicted, based on computational studies, that the intermediate [Fe^IV^(SyrB2)­(O)­Cl] has a six-coordinate structure and succinate acts as a bidentate ligand.^200–202^ However, DFT-calculated NRVS spectra of six-coordinate structures do not reproduce the experimental NRVS data, shown in Figure 11. Instead, the DFT analysis of the NRVS data showed that the intermediates with chloride or bromide bound have five-coordinate trigonal bipyramidal (TBP) geometries. The authors further assigned the NRVS data: vibrations in region 1 (340–400 cm^–1^, see Figure 11) originate from Fe-succinate stretching vibrations; in region 2 (285–340 cm^–1^), the signals originate from a pair of *trans*-axial bending modes, and in region 3 (200–285 cm^–1^), the *trans*-axial stretch and the Fe–X stretch are observed. The Fe–Cl/Br stretching modes are observed at 260 and 230 cm^–1^, respectively. Although the Fe–O stretch could not be identified in the data, due to the relatively low sample concentration (∼1.8 mmol), the data allowed for the assignment of the structure of the two intermediates. The DFT analysis further showed that the O_2_ reaction coordinate with the iron center produces an intermediate in which the Fe–oxo vector is oriented perpendicular to the substrate C–H bond. This arrangement facilitates HAA via the π* frontier molecular orbitals (FMOs), positioning the resulting substrate radical for efficient Cl^•^ rebound. In contrast, with a non-native substrate, alterations in the O_2_ reaction coordinate can yield an Fe–oxo vector parallel to the C–H bond. This orientation promotes HAA through a σ-pathway, positioning the radical for HO^•^ rebound and ultimately favoring hydroxylation over halogenation (see also section 1.3.2.3).

**Figure 11:**
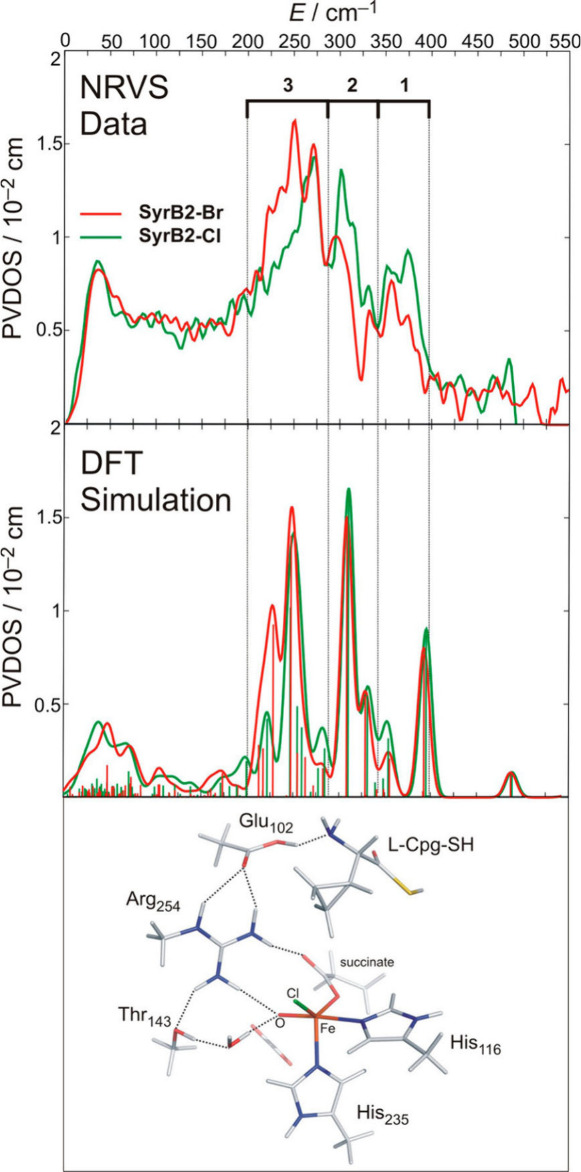
Top: Experimental NRVS data of the Fe­(IV)–oxo intermediate of SyrB2, with the Fe­(IV)–oxo intermediate being further ligated by either Cl^–^ (green line) or Br^–^ (red line). Middle: DFT-predicted NRVS data based on the TBP structure of the Fe­(IV)–oxo intermediate with inert substrate L-Cpg bound. The DFT-optimized structure is shown on the bottom. Reproduced with permission from ref 22. Copyright 2013 American Chemical Society.

Similarly, NRVS data for iron–oxygen intermediates in other nonheme iron enzymes were reported by Solomon and co-workers, including data for sMMO Intermediate Q (see below)^54^ and the peroxo intermediate of 4-Aminobenzoate *N*-Oxygenase (AurF).^203^

NRVS data for mononuclear Fe­(IV)–oxo model complexes were used to determine the Fe–O stretching frequencies of these compounds. In 2008, Solomon and co-workers studied^204^ three previously reported *S* = 1 Fe­(IV)–oxo complexes with the TMC,^205^ N4Py,^206^ and BnTpen^207^ ligand frameworks. NRVS data for [Fe^IV^(TMC)­(O_anti_)­(MeCN)]^2+^, [Fe^IV^(N4Py)­(O)]^2+^ and [Fe^IV^(BnTpen)­(O)]^2+^ show the Fe–O stretch at 831 (796 with ^18^O), 820 (788) and 824 (786) cm^–1^, respectively (see Figure 12), which is in good agreement with vibrational data for [Fe^IV^(TMC)­(O_anti_)­(MeCN)]^2+^ from IR (834 cm^–1^) and rRaman (839 cm^–1^) spectroscopy.^205,208^ From DFT analysis, the NRVS feature around 653 cm^–1^ for [Fe^IV^(N4Py)­(O)]^2+^ and [Fe^IV^(BnTpen)­(O)]^2+^ is assigned to an Fe–N_eq_ stretching mode, which is shifted to 526 cm^–1^ in [Fe^IV^(TMC)­(O_anti_)­(MeCN)]^2+^, due to the weaker tertiary amine σ-donation compared to the stronger pyridine σ-donation. The next intense features at 391, 381, and 389 cm^–1^ for the three complexes are assigned as Fe–N_ax_ stretching modes. In 2011, Solomon and co-workers reported NRVS data for the previously studied^209^ *S* = 2 [Fe^IV^(TMG_3_tren)­(O)]^2+^ complex. Here, the Fe–O stretching mode was identified at 821 cm^–1^, followed by an Fe–N_eq_ stretch at 515 cm^–1^, the latter being lower in frequency compared to the equatorial Fe–N stretching modes for the *S* = 1 complexes discussed above. The Fe–N_ax_ stretch is observed at 363 cm^–1^ in the NRVS data of [Fe^IV^(TMG_3_tren)­(O)]^2+^.^210^

**Figure 12:**
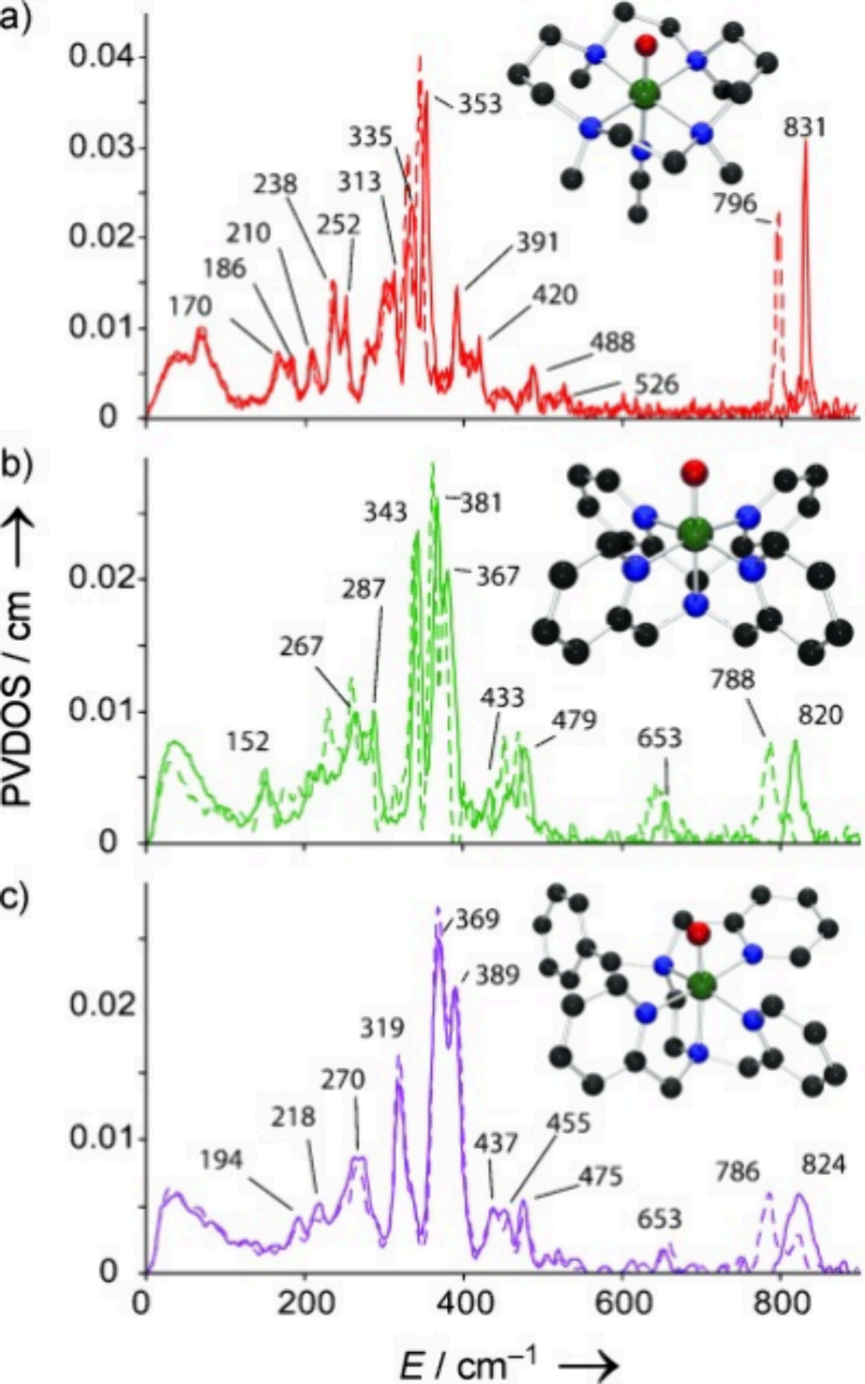
NRVS VDOS for (a) [Fe^IV^(TMC)­(O_anti_)­(MeCN)]^2+^, (b) [Fe^IV^(N4Py)­(O)]^2+^, and (c) [Fe^IV^(BnTpen)­(O)]^2+^ with ^16^O (solid lines) and ^18^O (dotted lines). Insets show the structures of the complexes. Reproduced with permission from ref 204. Copyright 2008 Wiley.

The exact structure of the Fe­(IV)_2_ Intermediate Q in sMMO, which is responsible for breaking the strong C–H bond in methane, is still under debate (see Scheme 1 and discussion above). Recently, Solomon and co-workers used NRVS at cryogenic temperatures to investigate the geometric and electronic structure of Intermediate Q.^54^ By combining NRVS data collected over several years (including multiple samples), they identified key vibrational features of this species. For further analysis, a set of 90 open- and closed-core structural models were then generated using DFT calculations to interpret the NRVS and other available spectroscopic data. Closed core Q models of the bis­(μ-O), bis­(μ-O)–OH, and bis­(μ-O)–H_2_O subclasses are predicted to show three modes above 600 cm^–1^: a symmetric (a_1g_) breathing mode at 700 cm^–1^ as well as two nonsymmetric (b_3u_ and b_2u_) modes between 600 and 650 cm^–1^ (using effective D_2h_ symmetry for the Fe_2_O_2_ core; Figure 13). The breathing mode is predicted to shift by 30–40 cm^–1^ and 15–20 cm^–1^ to lower frequency with ^18^O_2_ and ^16^O^18^O isotope substitution, respectively. No proton involvement in the breathing mode is predicted, reproducing previously obtained rRaman data, which show the corresponding mode at 690 cm^–1^ (see above).^53^ All bis­(μ-O) models, as well as 12 out of 16 bis­(μ-O)–H_2_O models, show this vibrational feature. For the open core model, the presence of the Fe=O bond weakens the Fe–(μ-O) bond, resulting in a shorter Fe–(μ-O) bond for the other iron, which allows ν­(Fe–OH) + ν_as_(Fe–O–Fe) coupling, resulting in a symmetric mode that would agree with the 690 cm^–1^ rRaman feature.^211^ On the other hand, ^18^O labeling provides evidence against this possibility, and supports the closed-core model.^53,192,193^ A notable challenge with the NRVS data analysis, however, is that all data contain mixtures of different species, including a paramagnetic Fe­(III)­Fe­(IV) form, a diamagnetic diferric species, and then Intermediate Q. More work is necessary to ultimately determine the exact structure of Intermediate Q.

**Figure 13:**
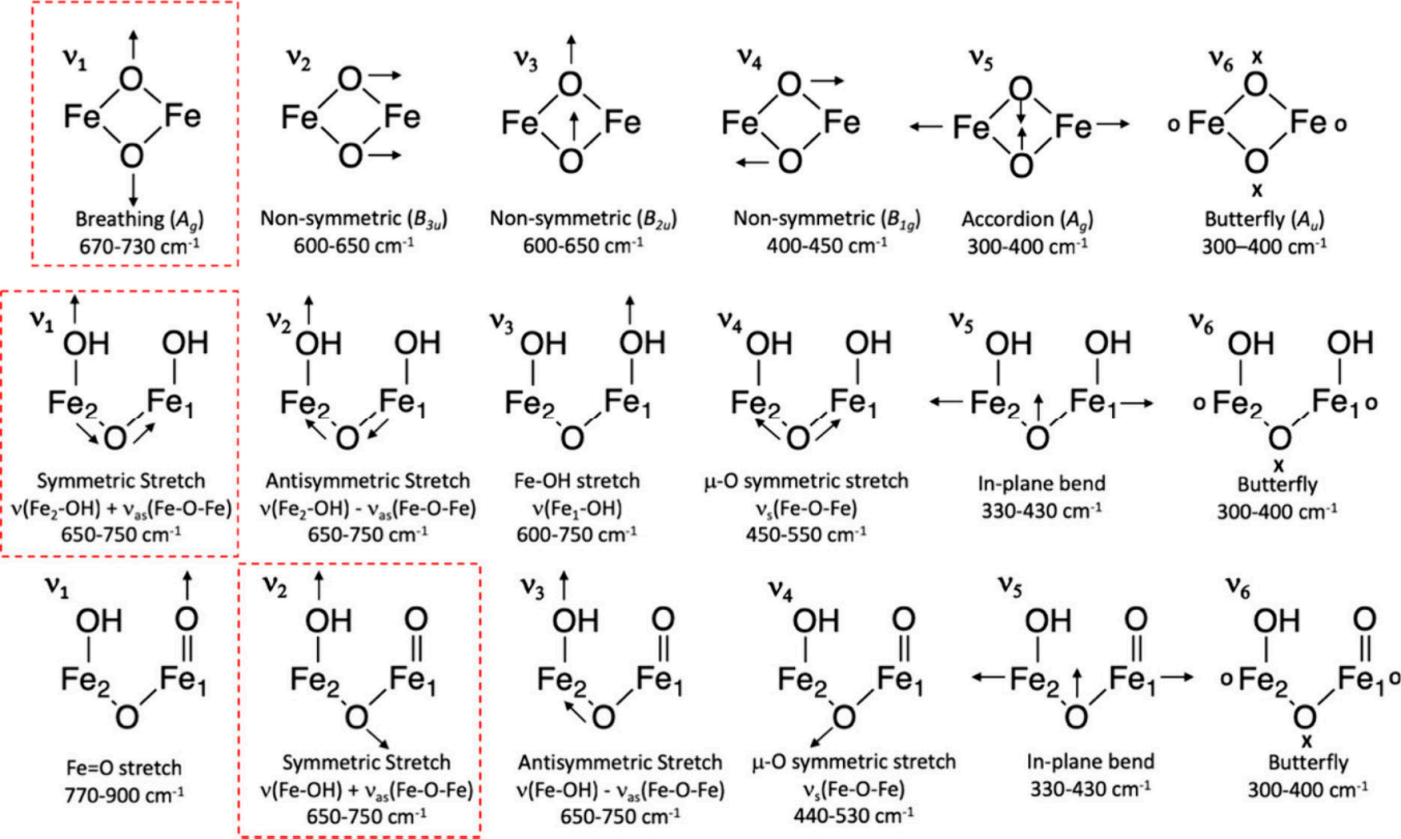
Normal modes for the Q closed-core bis­(μ-O), bis­(μ-O)–OH, and bis­(μ-O)–H_2_O subclasses (top) and Q open-core OC–OH/–OH (middle) and OC–OH/=O subclasses (bottom). Closed core modes are classified in effective *D*_2*h*_ symmetry. The calculated vibrations in each of these models that correspond to the reported rRaman 690 cm^–1^ vibration are highlighted in red. Arrows denote in-plane motions, and “x” and “o” characters denote out-of-plane motions. Reproduced with permission from ref 54. Copyright 2021 American Chemical Society.

##### 1.3.2.3 EPR and MCD Spectroscopy

Fe­(IV)–oxo centers (either *S* = 1 or 2) generally lack any detectable signals in standard (perpendicular mode) electron paramagnetic resonance (EPR) spectroscopy.^212–216^ In cases of small zero-field splitting, parallel mode EPR has in some cases allowed for the detection of these species.^217^ In contrast, Cyt P450 Cpd I is EPR-active because it consists of an *S* = 1 Fe­(IV)–oxo center that is antiferromagnetically coupled to an *S* = 1/2 porphyrin radical (*S*_tot_ = 1/2). In general, this magnetic interaction (represented by exchange coupling constant *J*) gives rise to nominal doublet (*S*_tot_ = 1/2) and quartet (*S*_tot_ = 3/2) manifolds, but the large zero-field splitting (represented by axial ZFS parameter *D*) of the ferryl center mixes and separates those spin states into three Kramers doublets. At ambient temperatures, only the lowest-energy Kramers doublet is populated, so the system behaves effectively as *S*_tot_ = 1/2 and gives corresponding signals in the EPR spectrum.^15^ For CYP119 Cpd I, Rittle and Green have found *g*_eff_ = 2.00, 1.96, 1.86, which are indicative of antiferromagnetic coupling and |*J*|/*D* = 1.3 (see Figure 14). For CPO Cpd I, the authors found quite similar *g*_eff_ = 2.00, 1.72, 1.61 and |*J*|/*D* = 1.02, values in good agreement with those previously reported by Rutter et al.^218^ It was previously suggested that there is a relationship between the nature of the axial ligand in Cpd I and the value of the exchange coupling constant *J* between the Fe­(IV)–oxo unit and the porphyrin radical.^219–221^ However, recent work by Hendrich and co-workers on Cpd I in different Cyt *c*s has shown that this is not the case, as these proteins do not follow the rule. The magnitude and sign of *J* likely depend on heme distortion and peripheral substitution of the cofactor.^222,223^ Using electron–nuclear double resonance spectra (ENDOR) of HRP Cpd I, Hoffman and Hager measured the weak hyperfine interaction of the unpaired electrons of Cpd I with the isotopically labeled ^17^O nucleus, to show that an oxygen atom is bound to the iron center in this species.^224^ Additionally, ENDOR findings were crucial to associate the broad EPR signal of HRP Cpd I with a porphyrin π-cation radical.^225^

**Figure 14:**
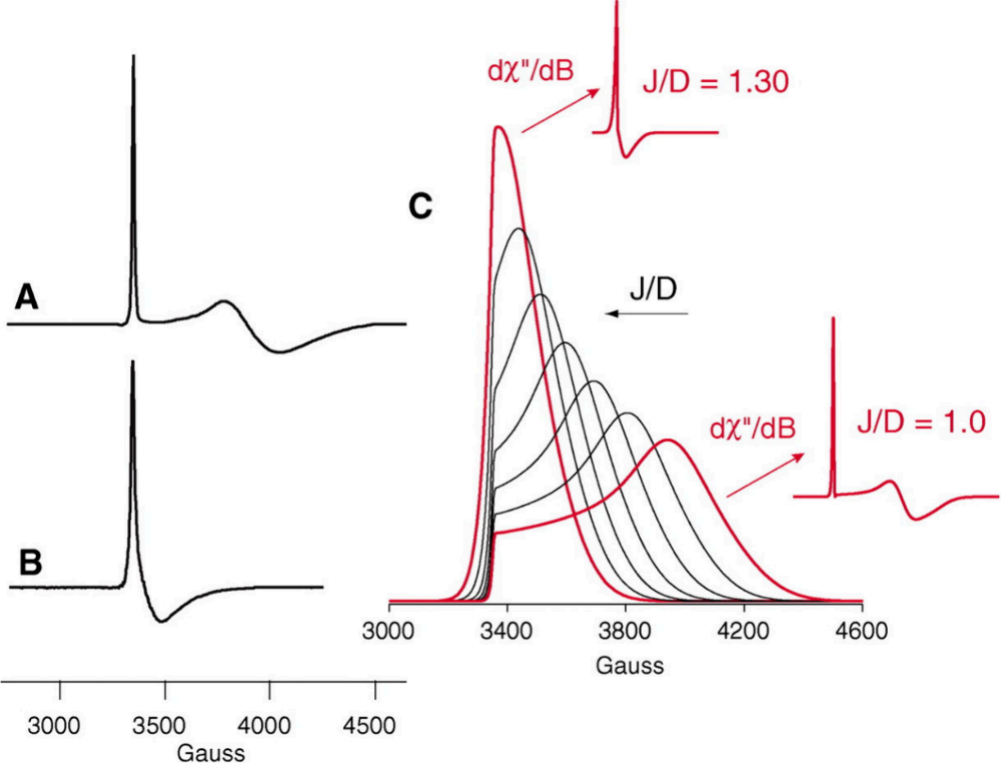
(A) EPR spectrum of CPO Cpd I at 8 K taken with 203 mW microwave power. (B) EPR spectrum of CYP119 Cpd I at 7.5 K. The CYP119 Cpd I spectrum was obtained from the difference of spectra taken with 203 mW and 128 mW microwave power. (C) The effect of |*J*|/*D* on the EPR spectrum is best illustrated through a comparison of the simulated absorption spectra. Reproduced with permission from ref 15. Copyright 2010 AAAS.

Magnetic circular dichroism (MCD) spectroscopy is an optical technique that measures the difference in absorption of left- and right-circular polarized light by a sample in a magnetic field that is oriented parallel to the direction of light propagation.^226^ MCD, especially the **C**-term component at low temperature and high field, is exceptionally sensitive to ground-state properties such as spin state, oxidation state, coordination geometry, and ligand-field environment of a paramagnetic species.^227,228^ In addition, the variable-temperature variable-field (VTVH) **C**-term signals are sensitive to the polarizations of the optical transitions, and can therefore be used to solidify the assignments of optical spectra.^47,229–231^

Solomon, Que and co-workers used variable-temperature (VT) MCD spectroscopy^232^ to study the Fe–O bonding in three *S* = 1 Fe­(IV)–oxo complexes, [Fe^IV^(TMC)­(O)­(MeCN)]^2+^,[Fe^IV^(TMC)­(O)­(OC­(O)­CF_3_)]^+^, and [Fe^IV^(N4Py)­(O)]^2+.^ This work allowed for an assignment of the near-infrared (NIR) absorption bands typically observed for these types of species. These NIR optical features are relatively broad and involve several electronic transitions, one of them being the d_*xy*_ to d_*xz*/*yz*_-O­(π*) CT transition. The energy of this transition relates to the strength of the Fe–O bond and the π-overlap between the oxo­(π) and the d_*xz*/*yz*_ orbitals. Another electronic transition that contributes to the NIR optical features is the d_*xy*_ to d_*x* ^2^__–*y* ^2^_ d–d transition, the energy of which reflects the strength of the equatorial ligand field. The authors further revealed that stronger Fe–O π-bonding correlates with higher HAA reactivity: they found that [Fe^IV^(N4Py)­(O)]^2+^ shows the strongest π-bonds and the highest reactivity, attributed to significant oxo character in the π* frontier molecular orbitals of the complex.

Solomon and co-workers also studied the Fe­(IV)–oxo intermediate of the nonheme iron enzyme SyrB2 using low-temperature MCD spectroscopy, to understand the electronic structure of its *S* = 2 ground state.^233^ VT MCD spectra of the *S* = 2 Fe­(IV)–oxo intermediate of SyrB2 closely parallel those of the Fe­(IV)–oxo model complex with the TMG_3_tren coligand (also *S* = 2), revealing key low-lying excited states and the FMOs that govern reactivity.^234^ A comparison between these intermediates shows that halide binding imposes π-anisotropy on the Fe­(IV)–oxo unit and increases oxo donor strength (see Figure 15), perturbing the FMOs and enhancing H-atom abstraction capability along the substrate direction via the π-channel (see section 2.4.2.1). This generates the TBP halide–Fe­(III)–OH (*S* = 5/2) intermediate and the substrate radical, which can then rebound with the halide or hydroxyl ligand.

**Figure 15:**
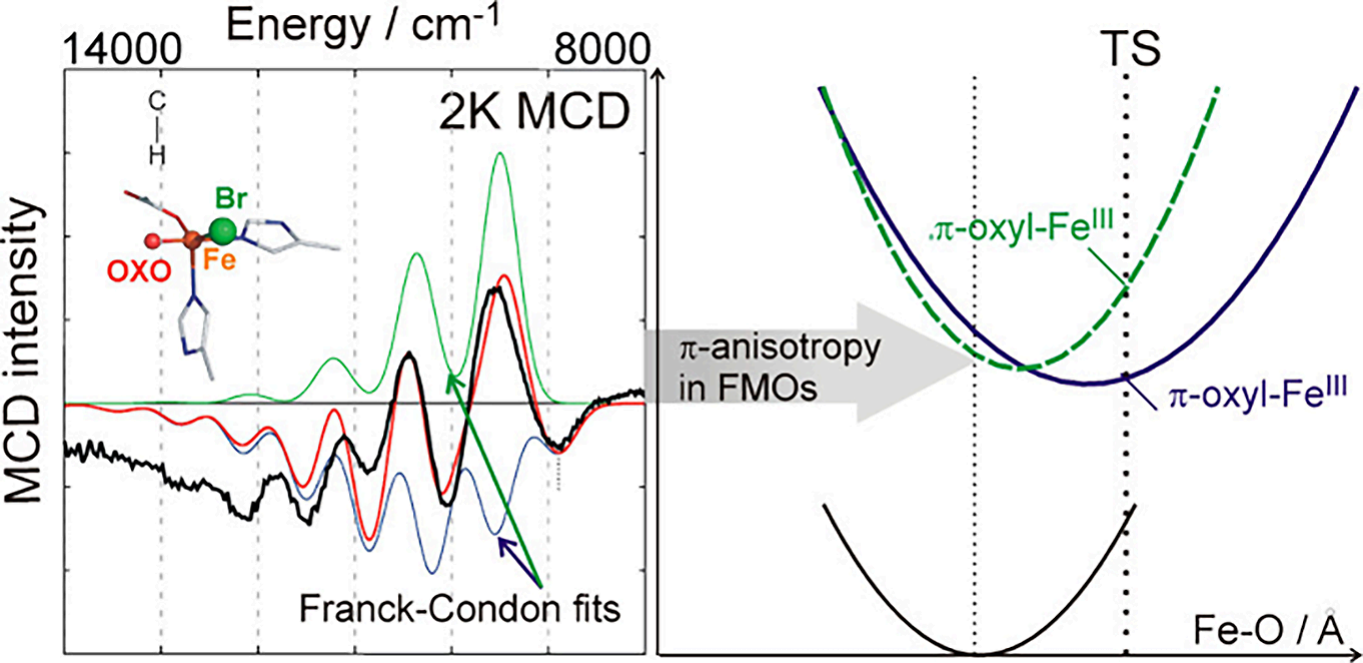
Left: MCD spectra of the SyrB2 Br–Fe­(IV)–oxo intermediate, indicating anisotropy in the two Fe–O π bonds, due to the presence of the halide, which disturbs one of these bonds and allows for the identification of two distinct d_*xy*_ to d_*xz*/*yz*_-O­(π*) CT transitions. Right: Potential energy surface for C–H bond activation. The π-anisotropy leads to a longer Fe–O distance and increased oxyl character in the transition state when the substrate attacks in the direction of the substrate cavity (through a π-channel, dark blue energy surface), in perpendicular orientation of the substrate C–H bond relative to the halide–Fe­(IV)–oxo plane. The Fe–O π bond that is perturbed by the halide gives the lower transition state energy for HAA (dark purple vs dashed green excited state energy surfaces). Reproduced with permission from ref 233. Copyright 2016 American Chemical Society.

##### 1.3.2.4 X-ray Spectroscopy and EXAFS

Many high-valent metal–oxo complexes are quasi-stable at cryogenic temperatures and/or trapped using methods such as rapid freeze-quench techniques. Such species are therefore not amenable to X-ray crystallographic techniques which require the generation of well-formed crystals. X-ray spectroscopies have proven highly valuable as a means of characterizing coordination environments about the metal-center in such compounds.^235,236^ Furthermore, these techniques can offer a means of obtaining detailed atom-specific electronic structural information concerning the complex. An advantage of X-ray spectroscopies in comparison to X-ray crystallography as a means of structurally characterizing metal-centers is that X-ray spectroscopies are atom-specific bulk techniques. Therefore, minor reaction products cannot be mistaken as representing the bulk material as can be the case with X-ray crystallography.

Broadly speaking, X-ray spectroscopies can be divided into absorption and emission techniques (Figure 16).^237^ X-ray absorption spectroscopies (XAS) examine transitions of core-level electrons into so-called bound and continuum states while X-ray emission spectroscopies (XES) examine the fluorescence decay of a higher-energy electron into a core-level hole following ionization. The following discussion will be limited only to electronic transitions involving first row transition metal (1s) core-orbitals (i.e., first row transition metal K-edge spectroscopies).

**Figure 16:**
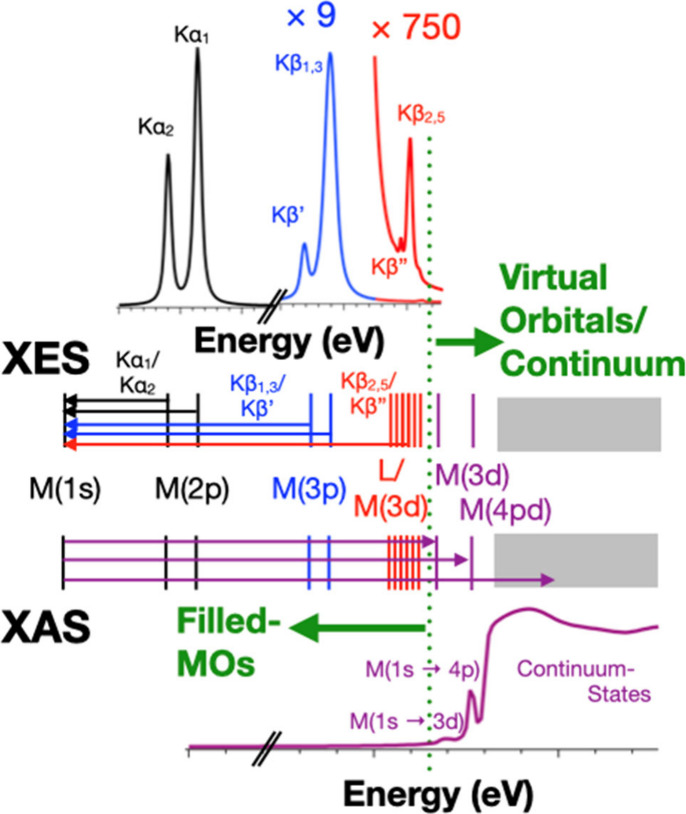
Schematic representation of the physical processes involved in X-ray emission (top) and X-ray absorption (bottom) spectroscopies probing metal K-edge transitions.

###### 1.3.2.4.1 X-ray Absorption Spectroscopies

The most widely employed X-ray spectroscopic techniques applied to high-valent metal–oxo complexes are metal K-edge XAS. The metal K-edge X-ray absorption spectrum can be divided into two different regions, the X-ray absorption near edge structure (XANES) and extended X-ray absorption fine-structure (EXAFS) regions (Figure 17a).^238^ The XANES region, which results from bound and low-energy continuum transitions, can be further broken down into pre-edge, rising edge and postedge regions, and contains a wealth of information concerning the geometry and electronic structure about the metal center. Two key pieces of information contained in the XANES pertinent to characterizing high-valent metal–oxo species are the edge position and the pre-edge peak (Figure 17b).

**Figure 17:**
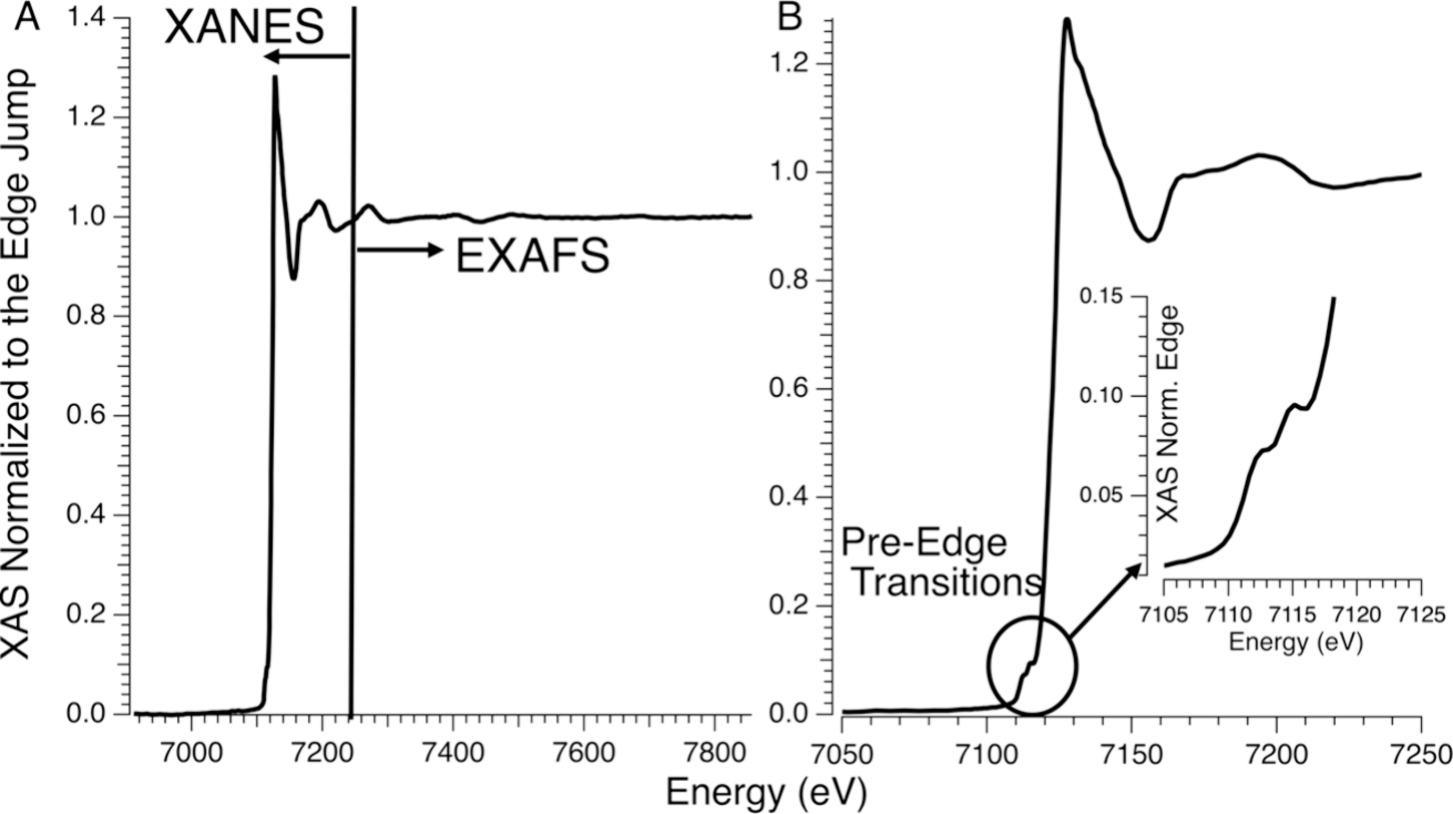
(a) Fe K-edge X-ray absorption spectrum of an Fe-porphyrin complex depicting the XANES vs EXAFS regions of the spectrum. (b) XANES region of the Fe K-edge X-ray absorption spectrum of an Fe-porphyrin complex. The inset depicts the pre-edge features resulting from nominal Fe­(1s → 3d) transitions.

The pre-edge feature, which is approximately 10 eV lower in energy than the edge-position, results from a dipole forbidden metal 1s → 3d transition that gains intensity through a quadrupole mechanism, and is therefore weak.^239,240^ However, the pre-edge feature can gain significant intensity by the mixing of a minor degree of metal 4p character into the 3d-set. Thus, the pre-edge feature can gain intensity as one transitions from a centrosymmetric (6-coordinate or 4-coordinate square-planar geometry) to a noncentrosymmetric geometry or if a short ligand is coordinated to the metal center. Both conditions will be met in the case of metal oxo complexes. For example, if one considers the calculated pre-edge intensity (PBE0/def2-tzvp­(-f)) of the Fe­(IV)–oxo species [Fe­(TMC-14)­(O)­(MeCN)]^2+^ as a function of Fe–O bond length one observes a dramatic increase in the calculated intensity of the Fe­(1s → 3d) transition as the Fe–O bond becomes shorter (Figure 18). Upon contraction of the Fe−O bond, Fe­(4p) character increases in the Fe­(3d) dominated acceptor states, which is a direct result of an increase in O­(2p)-Fe­(3d/4p) covalency. Thus, the intensity of the pre-edge feature can be used as a diagnostic criterion for the formation of a metal–oxo species.

**Figure 18:**
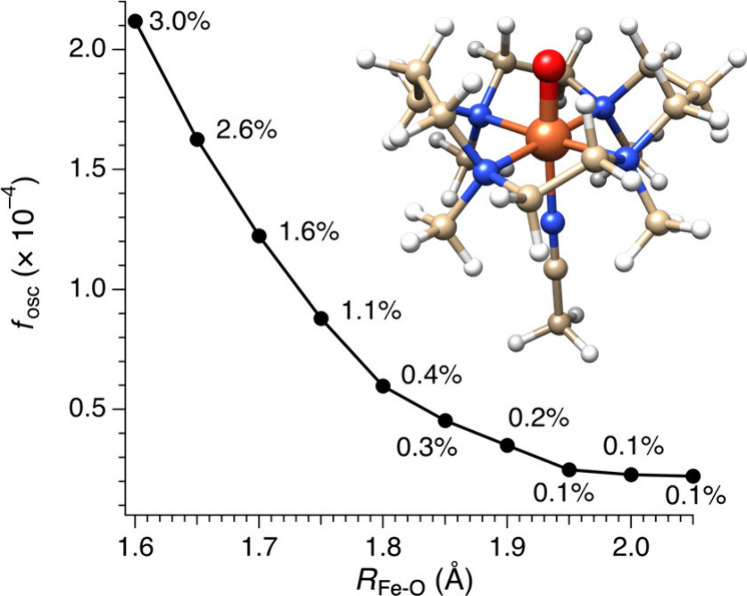
Calculated combined oscillator strengths vs Fe–O distance for [Fe^IV^(TMC-14)­(O)­(MeCN)]^2+^ (*S* = 1 spin-state; PBE0/def2-tzvp­(-f)/zora) for the nominal Fe­(1s→ 3d) transitions. Next to each calculated point is the %Fe­(4p) contribution to the acceptor MOs of predominantly Fe­(3d) character (Löwdin analysis) for the given Fe–O bond length. The inset depicts a ball-and-stick model of [Fe^IV^(TMC-14)­(O)­(MeCN)]^2+^.

The edge position is also often used as a criterion for the formation of a high-valent metal–oxo species. The edge is the sharp rise in absorption coefficient that results from the promotion of the metal 1s core electron into high energy bound and continuum states. As the metal oxidation state increases the core–electrons become more deeply buried, and thus one would expect to observe a blue-shift in the position of the edge as the charge on the metal-center increases–a change of +2 to 3 eV is expected for a change in the metal oxidation state of +1 (e.g., Fe^3+^ to Fe^4+^).^239,241–243^ Figure 19 depicts the XANES region of the Fe K-edge spectra for two formally Fe­(V)–oxo complexes, two [Fe­(TAML)­(O)]^−^ complexes with different degrees of ligand protonation, and the Fe­(III) starting material [Fe­(TAML)]^−^. As can be seen, upon oxidation of the Fe­(III) starting material there is shift of >3 eV in the edge positions indicating an increase in oxidation state of the iron center. One can also note changes to the pre-edge intensities. The square-planar complex [Fe­(TAML)^−^] displays a rather weak pre-edge feature while those obtained for the [Fe­(TAML)­(O)]^−^ species are significantly more intense. It was noted that the pre-edge features for [Fe­(TAML)­(O)]^−^ complexes differ depending upon the addition of a strong Brønsted acid, which protonates the carbonyl oxygen atoms of the TAML ligand and causes a change in Fe–O bond length of 0.04 Å. This increase in Fe–O bond length results in decreased mixing of Fe­(4p) character into the Fe­(3d) dominated MOs, thus leading to a decrease in intensity of the pre-edge transition in the protonated complex.

**Figure 19:**
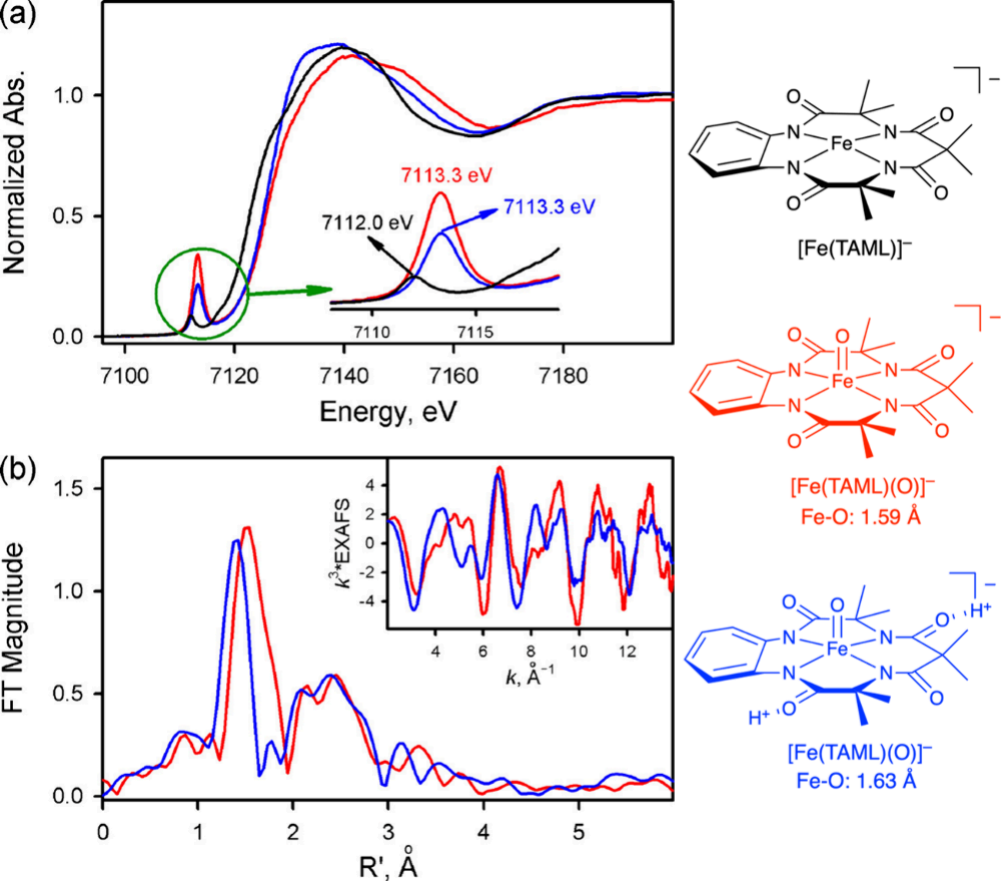
(a) XANES region of the Fe K-edge spectra for [Fe­(TAML)]^−^ (black) and [Fe­(TAML)­O]^−^ (red and blue). (b) EXAFS region of the Fe K-edge spectra for [Fe­(TAML)­O]^−^. Reproduced with permission from ref 244. Copyright 2020 ACS. The chemical drawings for [Fe­(TAML)]^−^ (black) and the corresponding [Fe­(TAML)­O]^−^ complexes (red and blue) are depicted to the right.

We note that extreme care must be taken in assignments of oxidation states on the basis of edge positions alone. First, an exact assignment of the edge position can be difficult leading to some ambiguity in its position. More important are two additional factors that are fundamental in what the edge position is truly reporting on. As stated, the edge position is a reporter on the overall charge of the metal-center and therefore is only roughly correlated to the formal metal oxidation state. Also, the edge position can be viewed as a so-called continuum resonance, which results from a short-lived excitation of the core-level electron into higher-energy states above the continuum level. The energies of these states are inversely proportional to the metal–ligand distance and are thus an *indirect* reporter of metal-oxidation state; shorter bond lengths are observed in complexes with higher oxidation states resulting in blue-shifted edge positions.^245,246^ Therefore, the edge position is not simply related to the oxidation state of the metal center. This can be illustrated nicely by examining the edge position of the FeX_3_ series (X = F, Cl, Br, I), which all contain iron in the formal 3+ oxidation state. Despite identical formal oxidation states there is an ∼5 eV shift^239^ in edge-position across the series owing to both decreasing Fe–X covalency and bond lengths as one progresses from X = I to F.

Analysis of the EXAFS region can yield structural information concerning metal–ligand bond lengths.^247–249^ The metal K-edge EXAFS phenomenon results from the backscattering of an outgoing metal 1s photoelectron by surrounding atomic potentials.^250^ In the case of an isolated atom (e.g., an atom in the gas phase) the outgoing spherically symmetric photoelectron wave does not interact with a near-by atom and thus as the energy increases there is a monotonic decay in the absorption coefficient (Figure 20a). However, in the case of near-by neighboring atoms the photoelectron is inelastically backscattered resulting in (de)­constructive interference of the outgoing wave (Figure 20b), which results in an oscillatory behavior in the absorption coefficient. This can be modeled using the single-scatterer approximation,^251^ wherein it is assumed that the outgoing wave only interacts with one neighboring atom before being deflected back; multiple scattering effects can complicate this and need to be accounted for in cases involving ridged ring systems (e.g., porphyrins, imidazoles, etc.) and linear ligands (e.g., CN^–^, CO, etc.). By assuming that the EXAFS region results from the summed total of all of the individual single-scattering interactions one can obtain information concerning the number and distances of the ligands about the metal center. Analysis of the EXAFS region involves the removal of the free-atom potential and other baseline effects and conversion from energy into *k*-space in units of Å^–1^ (Figure 20c). To amplify the oscillation at high energy the EXAFS are often weighted by *k* ^*n*^, typically *k* ^*3*^, which gives a good balance of amplifying the signal at high *k* without overamplifying the noise. An alternative view of the EXAFS region is obtained by examining the spectrum in radial space through the application of a Fourier transform (Figure 20d).

**Figure 20:**
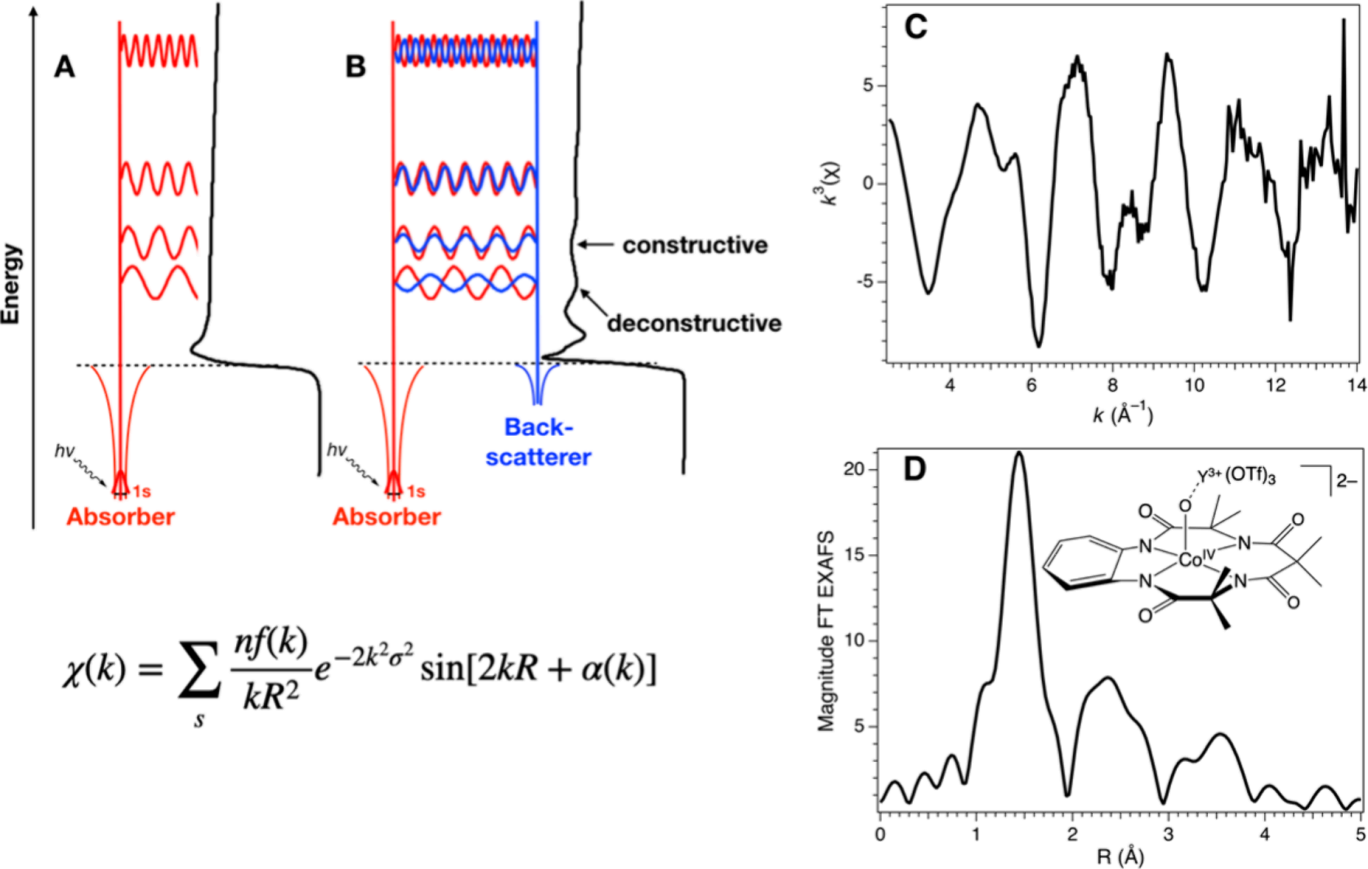
(A) Case of an isolated atom, such as a noble gas, where the propagation of a spherically symmetric photoelectron wave following ejection of a 1s electron from its nuclear potential will not interact with a nearest neighbor. This results in a smooth absorption coefficient with increasing energy. (B) When there is a nearby atom the outgoing spherically symmetric photoelectron wave will inelastically interact with the surrounding nuclear potential(s) and be backscattered toward the absorbing atom. This backscattered wave will interfere with the outgoing wave leading to constructive or deconstructive interference which causes the absorption coefficient to obtain a periodic oscillatory structure. (C) The *k* ^3^-weighted EXAFS data obtained for [Co^IV^(TAML)­(O)­(Y­(OTf)_3_)]^2–^. The EXAFS can be solved by modeling the data with 4 Co–N scatterers at 1.84 Å, 1 Co–O scatterer at 1.68 Å, 1 outersphere Co–Y scattering interaction at 3.98 Å and several shells containing Co–C scattering interactions from the TAML ligand.^252^ Note the increasing noise at higher values of *k* that becomes amplified with the *k* ^3^ weighting. (D) Magnitude FT *k* ^3^-weighted EXAFS data for [Co^IV^(TAML)­(O)­(Y­(OTf)_3_)]^2–^ showing the radial distribution of the scatterers about the cobalt center. The inset depicts the structure of [Co^IV^(TAML)­(O)­(Y­(OTf)_3_)]^2–^. The equation on the bottom left is the EXAFS single-scattering equation where χ­(*k*) is the EXAFS amplitude as a function of *k*, *n* is the number of scatterers in a given shell *s*, *f*(*k*) is an absorber–scatterer dependent amplitude function, σ^2^ is a structural disorder factor (typically the Debye–Waller factor) that accounts for thermal and static disorder, *R* is the average absorber-scatterer distance, and α­(*k*) is an absorber-scatterer dependent amplitude function.

A few factors must be kept in mind during the analysis of the EXAFS region.^251^ First, the spatial resolution of any two ligands with similar atomic masses (Z) is limited by the quality of the data at high *k*; two shells with differences in distances less than Δ*R* < π/(2Δ*k*) cannot be resolved from one another. This places a practical limit on the resolution of two shells to ∼0.11 Å for high-quality EXAFS data (usable data to *k* ∼ 16 Å^–1^). When considering the limited time these high-valent metal–oxo species can be exposed to the X-ray beam, which results in photoreduction of the metal–oxo compound, the resolution is more typically limited to ∼0.13–0.15 Å (usable data to *k* ∼ 12–14 Å^–1^). This is in addition to the fact that the reported bond lengths from an EXAFS measurement should be taken as having a ± 0.01–0.02 Å associated error. These factors are typically not an issue for the resolution of the M–O bond from other inner-sphere scatterers in metal–oxo complexes, owing to the typically short M–O bond lengths (<1.75 Å) relative to the other inner-sphere N/O scatterers, but does become a factor in M–OH compounds. For example, in the [Fe­(TAML)­(O)]^−^ complexes outlined above, the Fe­(V)=O scatterers at 1.59 and 1.63 Å are well resolved from the Fe–N^amide^ scatterers at ∼1.9 Å (Figure 19b). However, in the case of a recently characterized high-valent Co bis−hydroxo complex [Co­(PCAPD)­(OH)_2_]^2–^ (PCAPD = bis-[2-(1*H*-pyrrol-2-carboxamido)]-*o*-phenylenediamine), the two terminal hydroxo ligands could not be distinguished from the four ligand-derived nitrogen ligands.^253^

Lastly, one needs to be cognizant of the fact that if mixtures of species are present in the XAS sample with comparable concentrations, then an analysis of the EXAFS region will yield the averaged coordination environment about the absorber atom. This is especially important when dealing with these highly reactive metal–oxo intermediate species where it is possible that the resulting model would then be that of neither the intermediate nor the product. As the metal-based product is often a M–OH compound, the resulting EXAFS-derived structural model of such a mixture could lead to erroneously long metal–oxo bond lengths or the proposal of a completely invalid structure about the metal-center. We note that although it is mathematically possible to deconvolve mixtures of species in the EXAFS model, extreme care needs to be taken in the interpretation of such models as the increase in the number of fit parameters introduced from such a deconvolution strategy may make the resulting model statistically dubious. Furthermore, such an approach requires some *a priori* knowledge regarding sample composition, typically from other spectroscopic methods. However, it is highly unusual for one to subject complex mixtures of species to an EXAFS analysis; the vast majority of samples are assumed to be of one dominant product such that any additional species present in the sample do not contribute to the EXAFS, either as a whole or in the region of interest. In the end, it is extremely important that one ensures that the proposed structure from modeling EXAFS data is both chemically reasonable and consistent with all of the other available spectroscopic and possibly computational data.

In 2015 Green and co-workers published structural information on Cpd I from CPO and Cyt P450, derived from EXAFS measurements.^254^ It was discovered that the Fe­(IV)–O bond length in CPO Cpd I is shorter (1.661 Å) than that of Cyt P450 Cpd I (1.670 Å), which is directly correlated with the elongated bond length of the *trans* Fe–S bond (Figure 21). It is noted that the Fe­(IV)–O bond lengths obtained are in good agreement with those obtained for synthetic nonheme Fe­(IV)–O systems, determined both crystallographically (*vide infra*) and by EXAFS. The short Fe–O bond lengths observed for Cpd I can be contrasted with the Fe–O bond length found in CPO and Cyt P450 Cpd II, which are 1.82 Å (CPO Cpd II) and 1.84 Å (Cyt P450 Cpd II), demonstrating these are Fe–OH moieties in Cpd II, *not* Fe–oxo moieties.^255^ It was reasoned that the change in electron donation to the Fe=O moiety from the *trans* cysteinate sulfur moiety accounts for the differences in reactivity. The increased electron donation in Cyt P450 Cpd I destabilizes the Fe=O unit in Cyt P450 Cpd I vs CPO Cpd I, which in turn increases the driving force (lowers the reaction barrier) for C–H bond activation in Cyt P450 Cpd I relative to CPO Cpd I.

**Figure 21:**
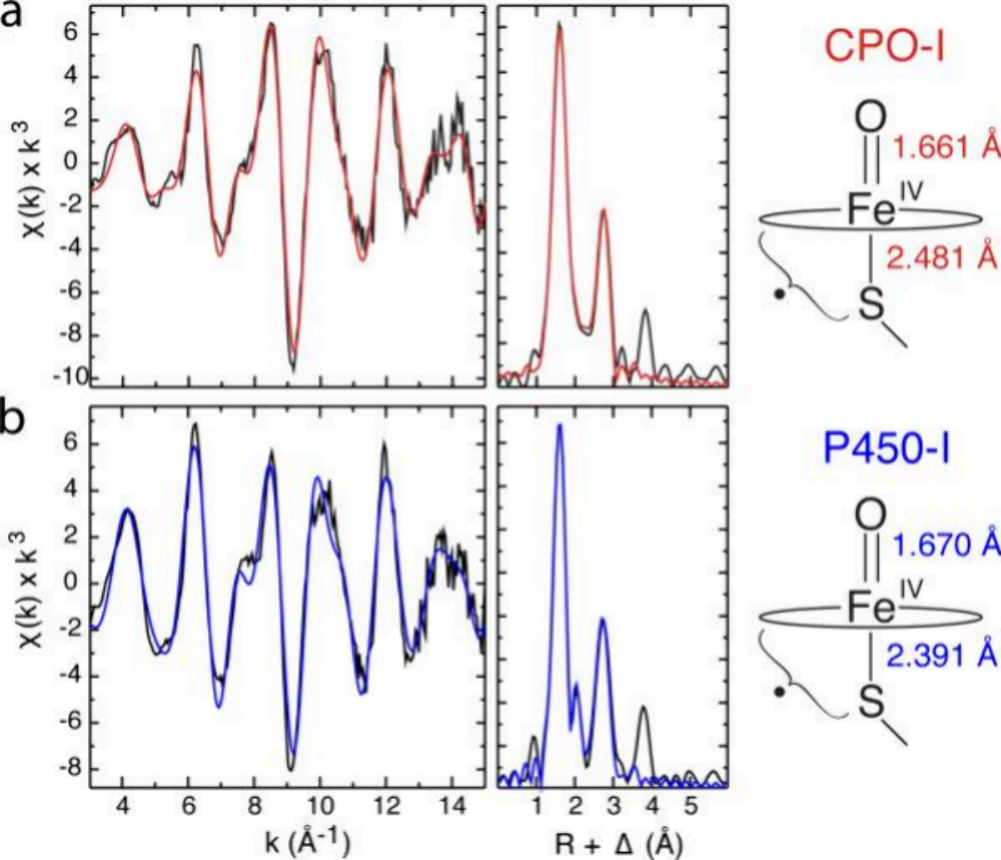
(a) Unfiltered *k* ^3^-weighted EXAFS (left) and magnitude FT *k* ^*3*^ *-* weighted EXAFS (right) obtained for CPO Cpd I (CPO-I). The experimental data are shown in black and the best fit to the data is depicted in red. The chemical figure to the right depicts the Fe–O and Fe–S bond lengths resulting from the best fit to the data. (b) Unfiltered *k* ^3^-weighted EXAFS (left) and magnitude FT *k* ^3^ *-* weighted EXAFS (right) obtained for Cyt P450 Cpd I (P450-I). The experimental data are shown in black and the best fit to the data is depicted in blue. The chemical figure to the right depicts the Fe–O and Fe–S bond lengths resulting from the best fit to the data. Reproduced with permission from ref 254. Copyright 2015 Nature.

###### 1.3.2.4.2 X-ray Emission Spectroscopies

In recent years, with the advent of improvements in synchrotron sources and end-station components, X-ray emission spectroscopies have increasingly become more common.^235,256,257^ Although these are powerful techniques for the elucidation of the geometric and electronic structures of transition metal centers, they have not been widely employed toward understanding high-valent metal–oxo chemistry of the mid to late transition metals. This is because of both experimental hurdles (i.e., photodecomposition, data collection time, poor signal for dilute samples, etc.) and the paucity of currently available beamline end-stations.

XES techniques can be broadly divided into nonresonant and resonant emission spectroscopies. For a first-row transition metal center, nonresonant X-ray emission results from the initial generation of a core M­(1s) hole from the ejection of an electron from the atomic potential by an X-ray whose energy is far above the 1s electron binding energy. Following ejection of the core M­(1s) electron, the M­(1s) core-hole is annihilated through several different decay channels (see Figure 16). The highest probability decay channel results from a M­(2p) electron decaying into the 1s hole yielding two lines, the *K*α_1_ and *K*α_2_ lines, which are split by ∼5–20 eV (depending on the metal-center) owing to metal 2p spin–orbit coupling (SOC). These lines are fairly insensitive to chemical environment, making them ill-suited as reporters for electronic structure in nonresonant studies, but are of greater utility in resonant emission spectroscopies (*vide infra*). Higher in energy are the *K*β transitions, the lowest in energy being the *K*β main-line transitions (the *K*β_1,3_ and *K*β′ transitions). The *K*β main-line transitions, which result from decay of a 3p electron into the 1s core hole, are approximately an order of magnitude less intense than the *K*α transitions. Splitting of the *K*β main-line transitions into the higher-energy *K*β_1,3_ and lower energy *K*β′ transitions occurs owing to 3p-3d exchange; further splitting of the *K*β_1,3_ transition is not observed owing to relatively small metal 3p SOC. Unlike the *K*α lines, the *K*β main-line transitions are highly sensitive to metal spin-state, oxidation state and metal–ligand covalency. Significantly less intense than the *K*β main-line transitions are the so-called valence-to-core (VtC) transitions, which are divided into the higher intensity *K*β_2,5_ transitions (resulting from decay of p-dominated states) and the lower intensity *K*β′′ transitions (resulting from decay of s-dominated states). The VtC region is information rich and can yield a host of information concerning ligand identity.

Resonant X-ray emission spectroscopies, often referred to as resonant inelastic X-ray scattering (RIXS) experiments, involve scanning the incident X-ray energy through the absorption spectrum while measuring the decay channels using a high-resolution crystal array XES spectrometer.^235^ These data therefore differ from fluorescence-detected XAS techniques (i.e., total or partial yield fluorescence; TFY/PFY) owing to the much narrower bandwidth of the detected XES signal. Most resonant XES experiments utilize *K*α detection owing to its greater intensity relative to the *K*β lines.^258–260^ One of the most useful resonant XES experiments is high-resolution fluorescence detected (HRFD) XAS.^261–263^ XANES spectra obtained by transitional XAS techniques are typically broadened owing to the short 1s core-hole lifetime. In a HRFD-XANES experiment the influence of the 1s core-hole lifetime is no longer an issue, resulting in a significantly narrower line-shape for the lower-energy bound transitions found in the XANES (see Figure 22). This is a powerful technique for the elucidation of electronic structure when coupled with time-dependent DFT calculations of the pre-edge region. When applied to the EXAFS region one obtains a HRFD-EXAFS spectrum. HRFD-EXAFS can yield higher metal-selectivity through the elimination of the Z+1 fluorescence background at higher *k* ^264^ (owing to the narrow emission bandwidth measured) and the elimination of beamline metal components^263^ that can lead to artifacts in dilute samples (owing to the use of a multicrystal emission spectrometer).

**Figure 22:**
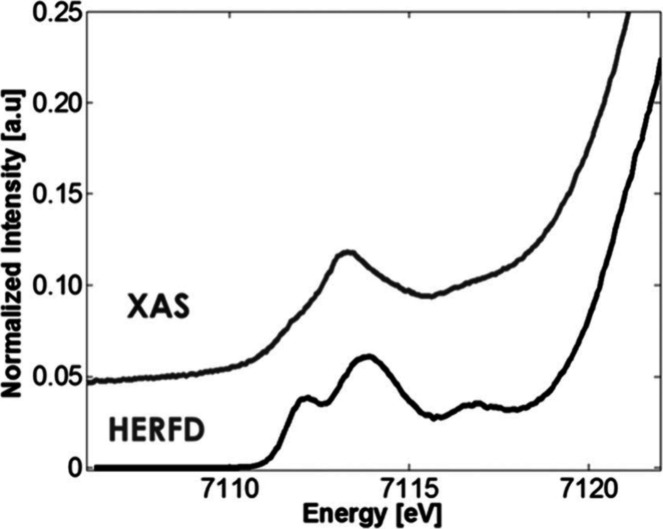
Pre-edge region of the iron K-edge XANES of the μ-dioxo-bridged diiron­(IV) complex **3** from Scheme 7 obtained by PFY- and HRFD-XAS. Reproduced with permission from reference 265. Copyright 2017 American Chemical Society.

HRFD-XAS and *K*β XES were utilized in attempting to elucidate the structure of Intermediate Q of sMMO. As discussed above (see section 1.3.2.2), it has been proposed that methane hydroxylation is effected by a high-valent diiron­(IV) intermediate known as Intermediate Q (MMOH_Q_, Figure 4). The exact nature of Intermediate Q has been of significant debate with two different structures proposed, an open and a closed form. The open form possesses a bridging μ-oxo ligand between the two Fe­(IV) centers and a terminal Fe­(IV)–oxo site. In contrast, the closed form possesses two Fe­(IV) centers bridged by two μ-oxo ligands forming a so-called “diamond core” structure. In addition to the spectroscopic results outlined above, the closed form had significant support based on early PFY EXAFS studies that identified an unusually short Fe······Fe vector (2.46 Å) in Intermediate Q. However, such a short Fe······Fe distance has never been observed experimentally or reproduced computationally; instead, Fe······Fe distances between 2.6–2.8 Å are typically observed in diamond-core structures. To address this ambiguity DeBeer, in collaboration with Lipscomb and Que, has reassessed the nature of Intermediate Q using XES techniques.

Based on the intensity of the *K*β′ transition and splitting between the *K*β′ and *K*β_1,3_ lines (Δ*K*β) it was demonstrated that Intermediate Q possesses two localized *S* = 2 Fe­(IV) centers that are antiferromagnetically coupled forming the *S* = 0 cluster (see Figure 23).^266^ A comparison of the HRFD-XANES spectrum of Intermediate Q with synthetic model compounds mimicking the open and closed forms of Intermediate Q (see Scheme 7) shows good agreement only between the open form model and Intermediate Q, not the closed form (see Figure 24a,b).^265^ The high resolution of the HRFD-XANES allows for the assignment of the individual transitions in the pre-edge feature, which is well reproduced by electronic structure calculations that employ an open core structure. Lastly, the HRFD-EXAFS data obtained for Intermediate Q lack the previously observed short Fe······Fe vector, and instead, show a longer Fe······Fe vector at 3.4 Å (see Figure 24c).^50^ The origin of the 2.46 Å Fe······Fe vector from the earlier PFY EXAFS spectrum of intermediate Q was attributed to “contaminate fluorescence” of iron–metal found in the beamline components. In fact, the PFY data could be nicely replicated by adding a small contribution of the spectrum of iron foil to the HRFD-EXAFS data (see Figure 24d).

**Figure 23:**
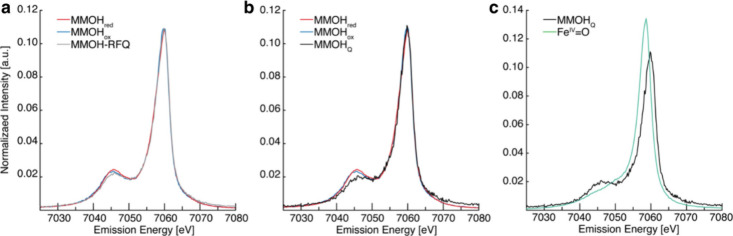
(a) *K*β main-line spectra of reduced and oxidized sMMO (MMOH_red_ and MMOH_ox_, see Figure 4) and the rapid freeze quench isolated oxidized sMMO-RFQ. (b) Spectrum of Intermediate Q generated by subtracting the components of MMOH_red_ and MMOH_ox_ from MMOH-RFQ. (c) Spectrum of Intermediate Q (MMOH_Q_) compared to a structurally characterized *S* = 1 Fe­(IV)=O synthetic complex [Fe­(2PyN2Q)­(O)]^2+^. Reproduced with permission from ref 266 under Creative Commons License CC BY. Copyright 2022 The Authors, Springer Nature.

**Scheme 7:**
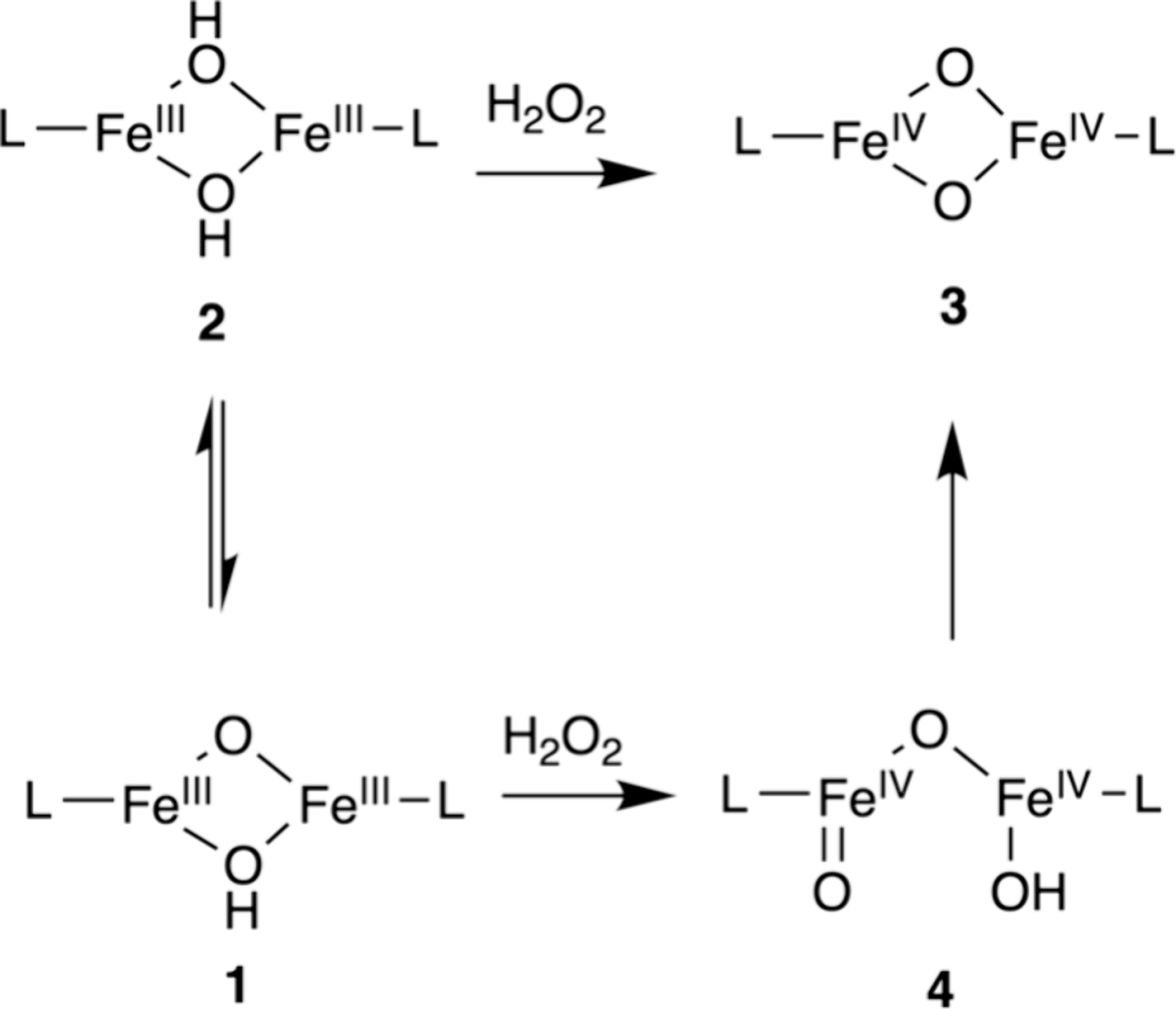
Synthetic Models of Different Core-Structures^52,193^ Used in the HRFD-XANES Studies to Aid in the Structural Elucidation of Intermediate Q (see Figure 23; L = Tris­[3,5-dimethyl-4-methoxypyridyl-2-methyl]­amine)

**Figure 24:**
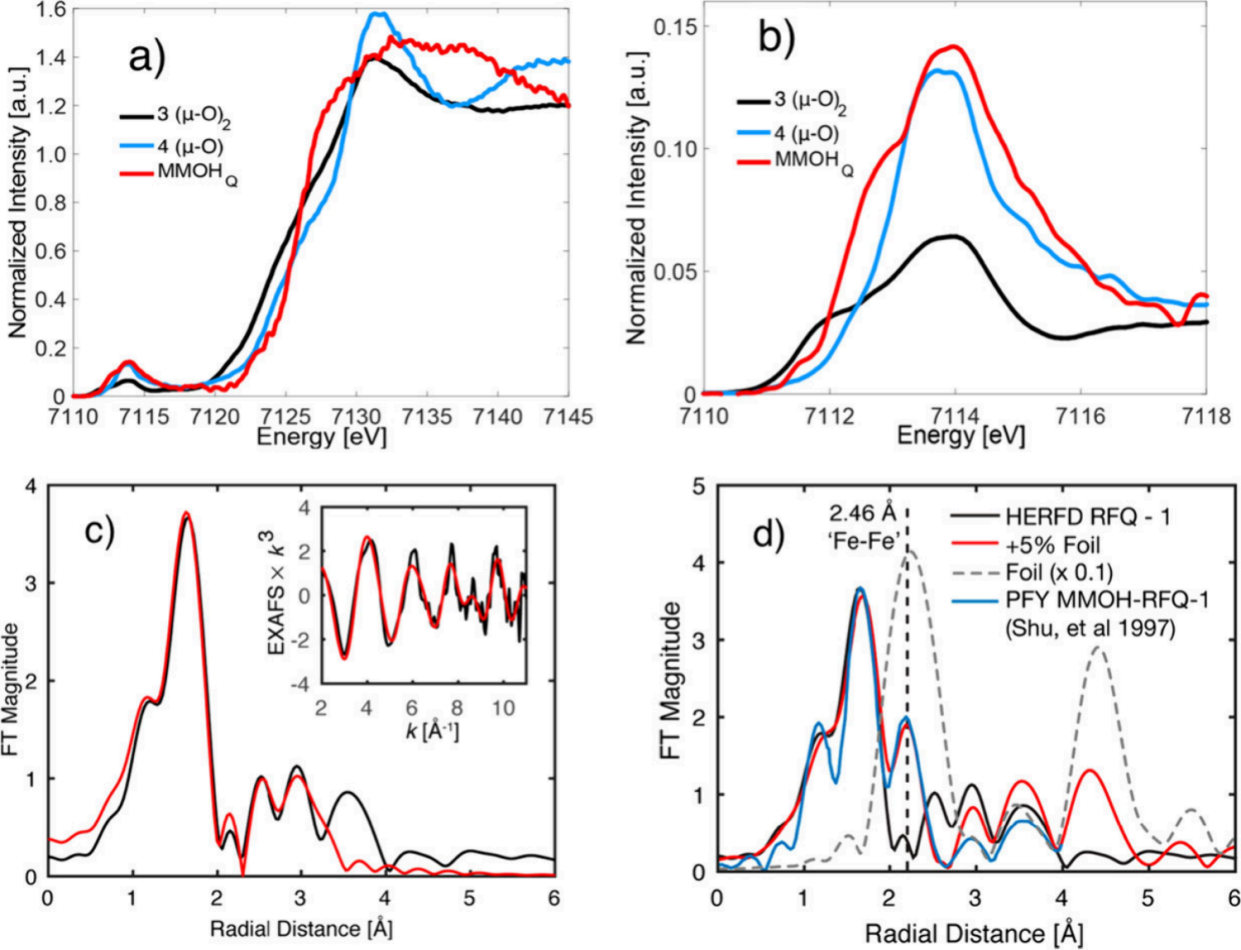
(a) HRFD-XANES of Intermediate Q (MMOH_Q_, red) and the closed (black) and open (blue) diiron­(IV) model compounds (see Scheme 7). (b) Pre-edge region of the HRFD-XANES spectra of Intermediate Q (red) and the closed (black) and open (blue) diiron­(IV) model compounds. (c) Magnitude FT *k* ^3^-weighted HRFD-EXAFS data of rapid freeze quench isolated sMMO (MMOH-RFQ), which contains Intermediate Q and reduced sMMO (MMOH_red_; in a ∼1:1 ratio). The inset depicts the unfiltered *k* ^3^-weighted HRFD-EXAFS data of MMOH-RFQ. The experimental data are given in black while the best fit to the data is shown in red. (d) Magnitude FT *k* ^3^-weighted EXAFS of HRFD-detected MMOH-RFQ (black), iron-foil (dashed gray), PFY detected MMOH-RFQ (blue) and HRFD-detected MMOH-RFQ convolved with a 5% iron foil “contaminant” (red). Reproduced with permission from refs 265 (a,b) and^50^ (c,d). Copyright 2017 and 2018 American Chemical Society.

In addition to measuring data at one emission energy, one can scan both the excitation and emission energies resulting in a 2D spectrum denoted as the RIXS plane (Figure 25).^267^ It is typical that the incident photon energy is plotted along the *x*-axis and the so-called energy-transfer is plotted along the *y*-axis; the energy transfer is the difference in energy between the excitation and emission energies. RIXS planes can be collected using any emission lines, but the collection of data at the *K*α (1s2p RIXS) or *K*β (1s3p RIXS) main lines is most typical. RIXS provides a wealth of information.^268–271^ First, it should be pointed out that RIXS is inherently a 2D spectroscopy and therefore transitions not observed in typical 1D experiments become resolvable in these 2D experiments. Furthermore, by taking different “cuts” along the RIXS plane different information can be obtained. The above HRFD-XAS experiments are obtained using constant emission energy cuts corresponding to the *K*α/β main lines. Constant excitation energy cuts yield L-edge (1s2p RIXS) and M-edge (1s3d RIXS) XAS-like spectra.^272,273^ This ability to produce spectra at atmospheric pressures that can normally only be obtained under UHV conditions makes these RIXS studies of potential use in obtaining insight into the electronic structures of quasi-stable intermediates.^274^ Section 2.4.2.2 provides an application of 1s2p RIXS in understanding the mechanism of hydrogen atom transfer by nonheme Fe­(IV)–oxo centers.

**Figure 25:**
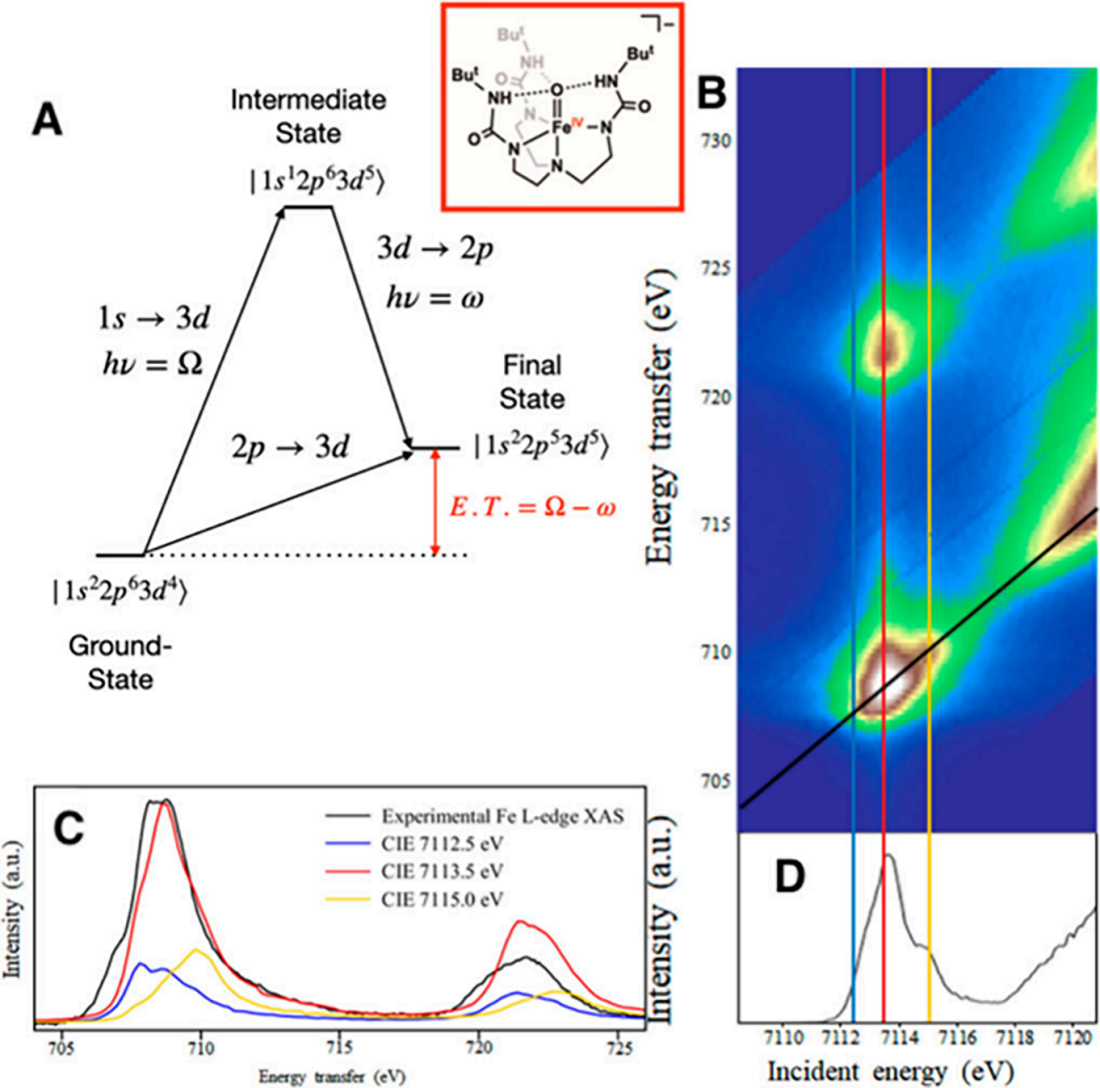
(A) Physical processes taking place during the 1s2p RIXS experiment for an Fe­(IV) complex. (B) 1s2p RIXS plane collected for [Fe^IV^(H_3_buea)­(O)]^−^. The black line on the diagonal depicts the constant emission energy slice resulting in a HRFD-XAS spectrum. The horizontal lines depict constant excitation energy slices resulting in L-edge XAS-like spectra. (C) L-edge XAS-like spectra resulting from different constant excitation energy slices probing different features in the pre-edge transition (blue, red, and yellow spectra) overlaid with the L-edge XAS spectrum (black) of [Fe^IV^(H_3_buea)­(O)]^−^. (D) HRFD-XANES spectrum of [Fe^IV^(H_3_buea)­(O)]^−^. The structure of [Fe^IV^(H_3_buea)­(O)]^−^ is depicted in the red box. (B,C) reproduced from ref 275. Copyright 2023 American Chemical Society.

##### 1.3.2.5 Mössbauer Spectroscopy

Mössbauer spectroscopy is a powerful tool for trapping and characterizing high-valent metal–oxo intermediates in enzymatic systems.^276,277^ This technique provides insights into iron-containing compounds, in particular their oxidation states, spin states, and electronic environments by analyzing isomer shifts (δ) and quadrupole splittings (Δ*E*_Q_).^276,278,279^ In particular, Mössbauer spectroscopy has been used to detect Fe­(IV)–oxo intermediates in iron enzymes due to their unusually low isomer shifts (see Table 2). For example, Krebs, Bollinger and co-workers applied Mössbauer spectroscopy to the nonheme iron enzymes TauD, CytC3 and SyrB2 to identify the critical Fe­(IV)–oxo intermediates, which show distinct, low isomer shifts (∼0.0–0.3 mm/s). Rittle and Green reported the Mössbauer parameters for CPO Cpd I, δ = 0.13 mm/s and Δ*E*_Q_ = 0.96 mm/s, and for CYP119 Cpd I, δ = 0.11 mm/s and Δ*E*_Q_ = 0.90 mm/s.^15^ Guo and co-workers discovered the first Fe­(IV)–oxo intermediate in a nonheme iron enzyme that has an *S* = 1 ground state, using Mössbauer spectroscopy. This enzyme, OvoA from *Methyloversatilis thermotolerans*, contains a nonheme iron center that is coordinated by three histidines in a plane. A Cys residue and a His residue bind as substrates in a monodentate fashion, leaving one vacant coordination site for O_2_ binding and activation for OvoA. The Fe­(IV)–oxo intermediate of OvoA displays an isomer shift of 0.16 mm/s and a quadrupole splitting of 1.35 mm/s.^280^

**Table 2:** Comparison of the Spectroscopic Properties of Fe­(IV)–oxo Intermediates in Metalloenzymes

type of enzyme	Fe(IV)–oxo intermediate in enzyme	λ_max_ (nm) [ε^d^ (M^–1^ cm^–1^)]	r(Fe=O) (Å)	ν(Fe=O) (cm^–1^)	δ (mm/s)	Δ*E*_Q_ (mm/s)	ref
nonheme	***J*** of TauD	318(1550)	1.62	821	0.31	–0.88	^24,281,282^
	***J*** of P4H	320 (1500)			0.30	–0.82	^108^
	TyrH				0.25	–1.27	^283^
	PheH				0.28	1.26	^284^
	IPNS				0.27	–0.44	^285^
	CytC3-Cl	318			0.30	–1.09	^98^
	CytC3-Br				0.22	–0.70	^286^
	SyrB2-Cl	318			0.31	–1.06	^199^
	SyrB2-Br				0.25	0.77	^197,199^
	Int490 *S* = 1				0.16	1.38	^280^
	Q of sMMO	330 (7500)	1.78 (Fe-μ-O)^c^	690	0.17	0.53	^49^
		430 (7500)					

heme	CPO Cpd I	367 (49)^a^	1.66		0.15	1.02	^218,254^
		545 (7),^b^ 610 (6.5), 688 (11.7)					
	CPO Cpd II		1.82	565			^190,255^
	Cyt P450 Cpd I	370 (∼50)^a^	1.67		0.11	0.90	^15,17,254^
		610 (∼40), 690 (40)					
	Cyt P450 Cpd II	370, 426^a^	1.84		0.10	2.05	^287^
		532, 565^b^					
	bC*c*P Cpd I	409^a^			0.09	1.21	^222^
		658					
	bCcP Cpd II				0.05	1.67	^222^
	HRP Cpd I	402 (80)^a^	1.60	790	0.08	1.25	^188,288,289^
		580 (5),^b^ 640 (5)					
	HRP Cpd II		1.80		0.03	1.61	^290^
	Cyt *c*′_β_ Cpd I	400 (93)^a^			0.08	1.30	^223^
		586 (5),^b^ 645 (15)					
	Cyt *c*′_β_ Cpd II				0.09	1.59	^223^
	catalase Cpd I	399^a^			0.12	1.09	^291,292^
		540,^b^ 624					

a Soret band.

b Q bands.

c A detailed discussion of Intermediate Q bond distances is provided in the previous section;

d ε for heme intermediates is in [mM^–1^ cm^–1^]

The Mössbauer properties of sMMO Intermediate Q have been studied by Que, Lipscomb and co-workers, and suggest an exchange-coupled high-valent Fe­(IV)­Fe­(IV) dimer. The Fe­(IV) oxidation state assignment is based on a significant decrease in isomer shift from δ = 0.50 mm/s in Fe­(III)­Fe­(III) sMMO to δ = 0.17 mm/s in Intermediate Q. This lower isomer shift aligns with values observed for well-characterized Fe­(IV)–oxo complexes in other nonheme iron enzymes and model complexes, confirming the presence of two Fe­(IV) centers in Intermediate Q.^49^

## 2 Fundamental Concepts: Electronic Structure and the Oxo Wall

### 2.1 Electronic Properties of Metal–Oxo Intermediates

Metal–oxo complexes (compounds where a metal is bonded to oxygen) are so common that scientists created special names to describe them. Back in 1903, Koppel and Goldmann^293^ suggested calling the VO^2+^ ion “vanadyl,” which was similar to how the UO_2_^2+^ ion was already called “uranyl”. Consequently, the FeO^2+^ ion is called “ferryl”. Here, the objective was to replace older names like “hypovanadate,” which were seen as confusing. The newer and more accurate way to describe these compounds is to call them “metal–oxo complexes,” where the oxo (O^2–^) group is seen as a ligand (something that binds to a metal). However, mixing the old and new naming styles has caused some confusing names to stick around, like “oxoferryl”, used for compounds with the FeO^2+^ group. Compared to V–, Mn–, and Fe–oxo complexes, high-valent metal–O species of groups 9–11 metals are exceedingly difficult to isolate and stabilize for detailed investigations, and have not been amenable to characterization by crystallographic techniques.^3^ Also, their electronic structure is more dubious due to bond inversion (see below). Due to the absence of crystallographic data, the geometric structures of late transition metal−oxo complexes must be derived solely through rigorous spectroscopic analysis coupled to computational studies. For copper, the corresponding, formally Cu­(III)–oxo intermediate has remained completely elusive.

The instability of groups 9–11 metal−oxo complexes, which was designated as the “oxo wall” by Ballhausen and Gray, can be rationalized from simple ligand-field theory arguments (see Figure 26).^294^

**Figure 26:**
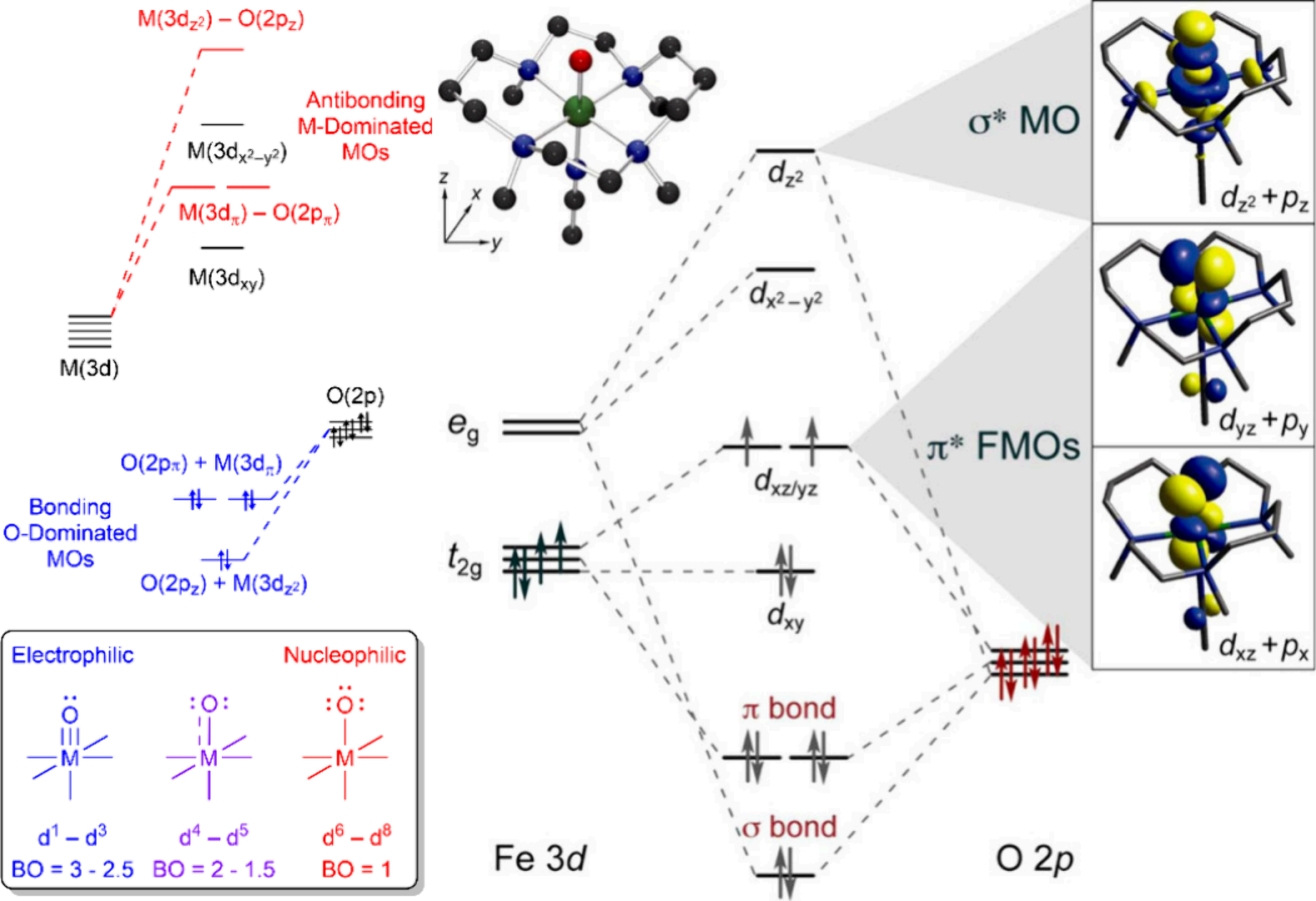
Left: Simplified ligand-field diagram of metal–oxo compounds in *C*_4*v*_ symmetry, highlighting the metal–O orbital interactions. Right: Molecular orbital diagram and contour plots illustrating the metal–O σ- and π-bonds, calculated for the *S* = 1 complex [Fe^IV^(TMC)­(O)­(CH_3_CN)]^2+^. Reproduced with permission from ref 22. Copyright 2013 American Chemical Society.

### 2.2 Definition of the Oxo Wall

Ballhausen and Gray applied MO theory to analyze the electronic structure of the vanadyl ion. The bonding interactions between a metal and an oxo ligand in tetragonal (*C*_4*v*_) symmetry yield three high-lying metal d-orbitals with significant M–O antibonding character (see Figure 26). Traversing from the left to the right of the transition metal block, the M­(ndπ)–O­(2pπ) π-antibonding orbitals become occupied, reducing, and ultimately eliminating multiple bonding character between the metal and the O^2–^ ligand. In this way, an electrophilic M–O multiple bonded species transforms into a M–O single bonded species that is both basic and unstable with respect to nucleophilic attack and oligomerization. In systems with tetragonal (*C*_4*v*_) symmetry, the retention of a metal–oxo double bond is only possible when the metal center possesses no more than four d-electrons. Achieving the low d-electron count necessary for sustaining metal–oxo multiple bonding becomes increasingly challenging for late transition metals (groups 9–11). This systematic breakdown of M=O multiple bonding in late transition metals is referred to as the “oxo wall,” a term introduced by Ballhausen and Gray.^294^ Despite occasional reports of “oxo wall violations” in the literature, such cases do not truly contradict the original oxo wall concept. Notably, the oxo wall applies specifically to metal–oxo species in tetragonal symmetry; systems lacking this geometry fall outside the scope of this concept.

### 2.3 Bond Inversion in Late Transition Metal–Oxo Intermediates

For groups 9–11 first-row transition metals, there is also the possibility of a bonding scheme or ligand field inversion.^295–297^ This results from (a) the increase in the effective nuclear charge of a transition metal as one traverses from early to late transition metals, or (b) an increase in the metal’s oxidation state; both are at play in groups 9–11 high-valent metal−oxo compounds. Under these circumstances, the metal-dominated frontier orbitals plummet in energy, and the high-lying antibonding M–O orbitals gain significant oxygen character. Although this does not necessarily alter the net M–O bond order, it changes the fundamental nature of the M–O moiety, thus generating a more electrophilic metal–oxyl (M–O^•–^) instead of a metal–oxo (M–O^2–^) species.^3,4,298–301^ Whereas in the case of V, Mn, and Fe, the corresponding intermediates are true metal–oxo complexes, the M–O unit transitions more and more toward metal–oxyl character from Co to Ni and finally Cu (Figure 27). In the extreme of ligand-field inversion, it is possible that significant metal–oxene (O atom) character is acquired. For Cu, the corresponding Cu­(III)–oxo complexes are generally considered as Cu­(II)–oxyl (or possibly Cu­(I)–oxene); we note these species have only been studied computationally thus far. Importantly, how this increase in metal–oxyl character affects different reactivities, especially C–H bond activation and OAT, is not known.

**Figure 27:**
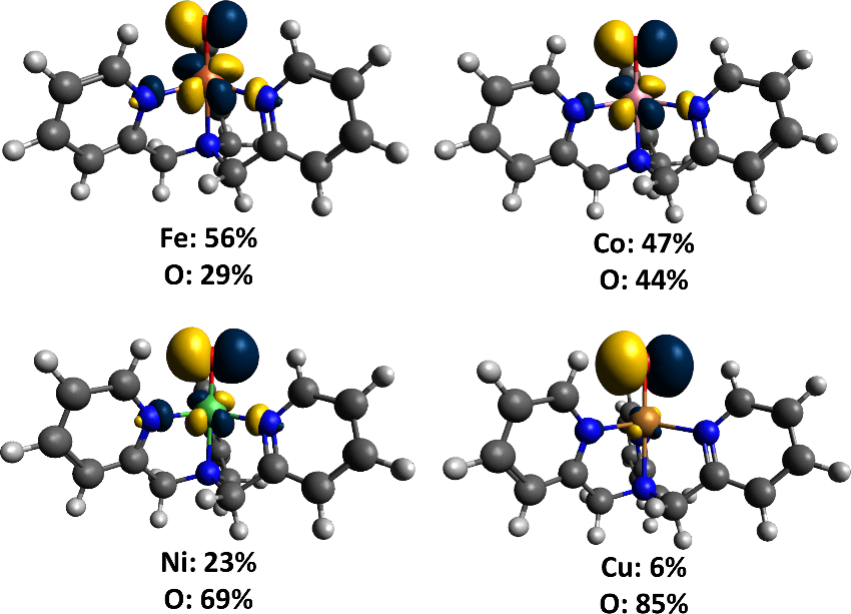
Plot and orbital charge contributions of the metal–O π-antibonding MOs for [M^IV^(TPA)­(O)]^2+^ (M = Fe, Co, Ni) and [Cu^III^(TPA)­(O)]^+^ (TPA = tris­(pyridylmethyl)­amine), demonstrating ligand–field inversion of the metal–O π-bonds when traversing from Fe to Cu.

### 2.4 General Reactivity Principles for Metal−Oxo Complexes

#### 2.4.1 Thermodynamic Driving Force for Hydrogen Atom Abstraction (HAA)

Hydrogen Atom Abstraction (HAA) is a fundamental reaction in biology where enzymes remove hydrogen atoms from molecules to start important chemical transformations. This process is carried out by special enzymes, like heme and nonheme iron enzymes, which employ high-valent iron­(IV)–oxo complexes to activate C–H as well as O–H and N–H bonds. Beyond metal–oxo intermediates, HAA reactions are also observed with many organic radicals, such as tyrosine and tryptophan radicals,^302^ peroxyl radicals,^303^ and radical SAM,^304^ in both biological and chemical contexts. HAAs are proton-coupled electron transfer (PCET) reactions in which a proton and an electron are removed from the substrate and transferred to the metal–oxo unit. In general, PCET reactions can be concerted (the proton and electron move at the same time), asynchronous (where one particle moves earlier than the other), or even stepwise, in two steps with a defined intermediate (ET/PT or PT/ET). Since it is *a priori* not clear how PCET from the substrate to the metal–oxo unit is orchestrated, we will generally refer to these reactions as HAA. Note that a Hydrogen Atom Transfer (HAT) reaction is a special case of a PCET where the proton and electron are transferred to the same location in the acceptor complex. This is observed in radical HAA reactions, R-XH + Y^•^ → R-X^•^ + Y–H, which are true HAT processes that have been studied in much detail by organic chemists.^305,306^

Mayer and co-workers analyzed the thermodynamic driving force for HAA and concluded that the ability of a metal–oxo species to abstract a hydrogen atom from toluene (the substrate originally studied) correlates with the bond-dissociation free energy (BDFE) of the M–OH bond formed after HAA.^307–309^ In this model, the reaction becomes more favorable (exergonic) when the newly formed M–OH bond is stronger, and this thermodynamic driving force largely determines reactivity regardless of the spin state of the metal–oxo species. The majority of BDFEs are calculated from known p*K*_a_ values and standard redox potentials (*E*°), using a thermochemical framework first developed by Bordwell for organic molecules^310^ and later extended by Parker and Wayner^311^ and by Tilset.^312^ The BDFE of the formed O–H bond is proportional to the reduction potential (*E*_1/2_) of the metal^*n*+^–oxo species, the basicity of the reduced metal^(*n*–1)+^–oxo species, expressed as the p*K*_a_ of the conjugate acid, and a constant C_g_, according to the Bordwell equation:^308,313^

$${\mathrm{BDFE}}_{O-H}=23.06E_{1/2}+1.37pK_{a}+C_{g}$$

This equation is often used to analyze the reactivity of related metal–oxo species, for example in Cyt P450s.^314^ According to this equation, the *E*_1/2_ and p*K*_a_ can be used to tune the reactivity of metal–oxo intermediates.

Experimentally, however, these properties are challenging to obtain for reactive and thus unstable metal–oxo complexes. Green and co-workers analyzed HAA by Cyt P450s and found that the p*K*_a_ of protonated Cpd II (the Fe­(IV)–OH complex) of CYP158 is 11.9, assessed by changing the pH of the solution from 13.9 to 9 and determining the obtained Fe­(IV)–oxo/Fe­(IV)–OH ratio.^287^ EXAFS data suggest there is a slight increase of the Fe–O bond length (by 0.15 Å at pH 9) compared to higher pH, which confirms the protonation of the Fe­(IV)–oxo complex.^287^ A few years later, Green and co-workers also assessed the redox potential of Cpd I of CYP158 using a redox titration with [Ir­(IV)­Cl_6_]^2–^.^316^ Through a combined experimental and theoretical approach, they determined the bond dissociation free energy (BDFE) of the MO–H bond of Cpd II. They reported a reduction potential of 1.22 V (vs NHE, at pH 7) for Cpd I, with a corresponding O–H bond strength in Cpd II of 95 kcal/mol, obtained from a thermodynamic “square scheme” (see Figure 28, right for an example). For Cpd II, the reduction potential was determined to be 0.99 V (vs NHE, at pH 7), with the O–H bond strength in the ferric−hydroxo complex determined to be 90 kcal/mol. For one nonheme Fe­(IV)–oxo complex, its Fe­(III)­O–H BDFE has been reliably assessed as well.^217^ This unique complex, [Fe^IV^(H_3_buea)­(O)]^–^, has been characterized by Borovik and co-workers and found to have an Fe­(III)­O–H BDFE of 87 kcal/mol (see Figure 28). Unfortunately, very little has been reported about its HAA reactivity, which may be significantly attenuated by the bulky groups surrounding the Fe­(IV)–oxo unit. Very recently, the Barnet group reported the most stable Fe­(IV)–oxo complex to date,^317^ which has a O–H BDFE of 91 kcal/mol. The BDFE of this complex is substantially larger than the BDFEs for the C–H bonds in different hydrocarbon substrates. Accordingly, the lack of substantial reactivity of this intermediate with 1,4-cyclohexadiene (CHD) and 9,10-dihydroanthracene (DHA) as substrates (with weak C–H bonds) can be ascribed to steric hindrance. Substrate access is one of the key factors for HAA reactions, which is well established in the literature, as shown, for example, by the Que group for their highly sterically hindered complex [Fe^IV^(TMG_3_tren)­(O)]^2+^ and a less sterically encumbered derivative.^318^ These examples confirm that the strength of the MO–H bond is not the sole factor determining HAA reactivity.

**Figure 28:**
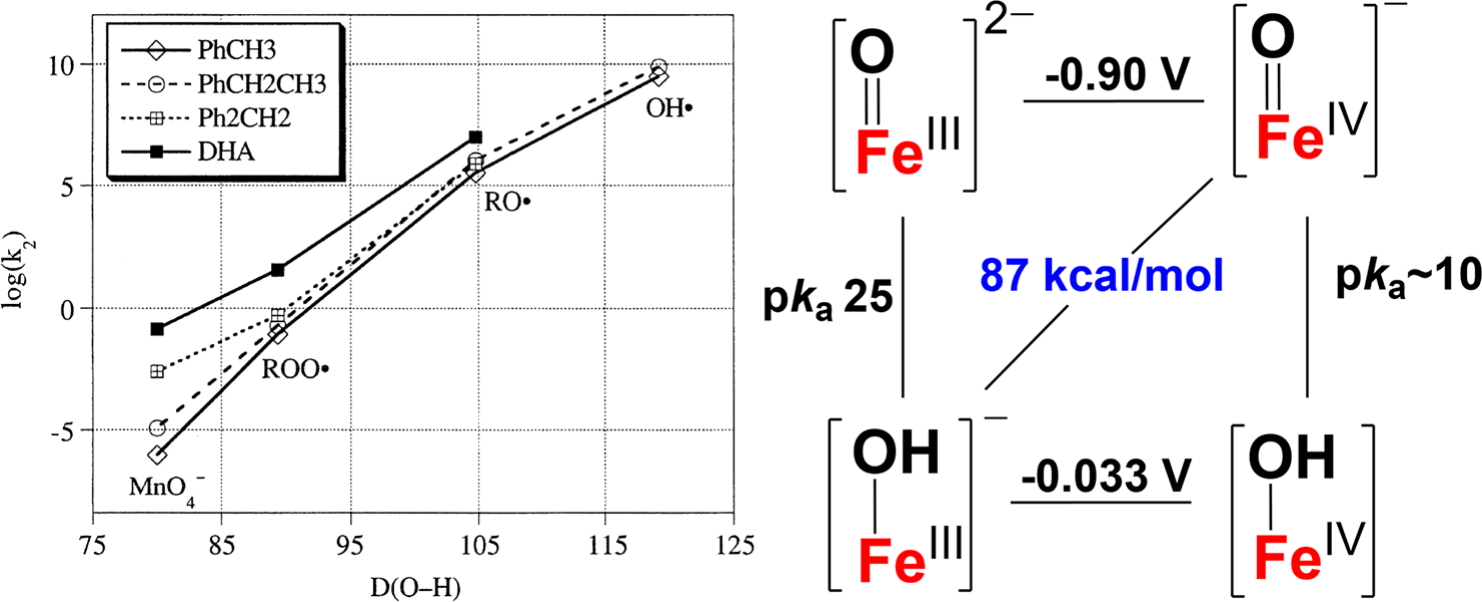
Left: Rate constants for HAA by HO^•^, RO^•^, tBuOO^•^, and MnO_4_^–^ vs the strength of the O–H bond formed for toluene, ethylbenzene, diphenylmethane, and dihydroanthracene. Reproduced with permission from ref 307. Copyright 1998 American Chemical Society. Right: Square scheme of the thermodynamic cycle for [Fe^IV^(H_3_buea)­(O)]^−^. Adapted from ref 315. Copyright 2013 American Chemical Society.

#### 2.4.2 The Kinetics of HAA

Besides the thermodynamics of C–H bond activation by metal–oxo complexes described above, the factors that govern the kinetics of the HAA reactions have been widely investigated. Other important factors that affect the kinetics of these reactions include the way the substrate approaches the metal–oxo center and the spin states involved, which affect the overall activation energy required for HAA, and the synchronicity of the PCET process. These factors are briefly discussed in the following.

##### 2.4.2.1 HAA σ vs π Reaction Channels

Solomon and co-workers introduced the concept of σ vs π reactivity channels to explain how Fe­(IV)–oxo complexes in the relevant *S* = 1 and 2 spin states perform HAA.^319,320^ In this model, reactivity depends on which molecular orbital of the Fe­(IV)–oxo unit the substrate interacts with (see Figure 29). For the C–H bond activation process, the first step is to abstract a hydrogen atom from the substrate to form the Fe­(III)­O–H complex and the substrate radical, followed by the radical rebound step (see below) to create the corresponding hydroxylated product. The energetic ordering of the d-orbitals, d_*xy*_ < d_*xz*,*yz*_(Fe–O π*) < d_*x*2–*y*2_ < d_*z*2_(Fe–O σ*) in *C*_4*v*_ symmetry, is a consequence of the strong σ and π donation from the oxo ligand to the Fe­(IV) center. The single-electron transfer in the HAA reaction on the *S* = 2 surface yields a roughly linear transition state and involves a σ attack of the substrate FMO on the d_z2_(σ*) orbital. This σ channel is characterized by limited steric interactions between the substrate and the Fe=O core. This often yields a lower steric contribution to the energy barrier for HAA at the transition state. In contrast, in the *S* = 1 state, the iron center is surrounded by a stronger ligand field from the coligand, causing a larger energy splitting of the d-orbitals and a shift of d_*z*2_(σ*) to higher energy. In this case, reactivity on the *S* = 1 surface is preferred via a π attack of the substrate FMO on the d_*xz*,*yz*_(π*) orbitals, which requires a side-on approach of the substrate on the Fe=O core (see Figure 29). This often results in large steric interactions between the substrate and the equatorial chelating coligand(s) and can result in a larger barrier at the transition state compared to the *S* = 2 surface.^321–323^

**Figure 29:**
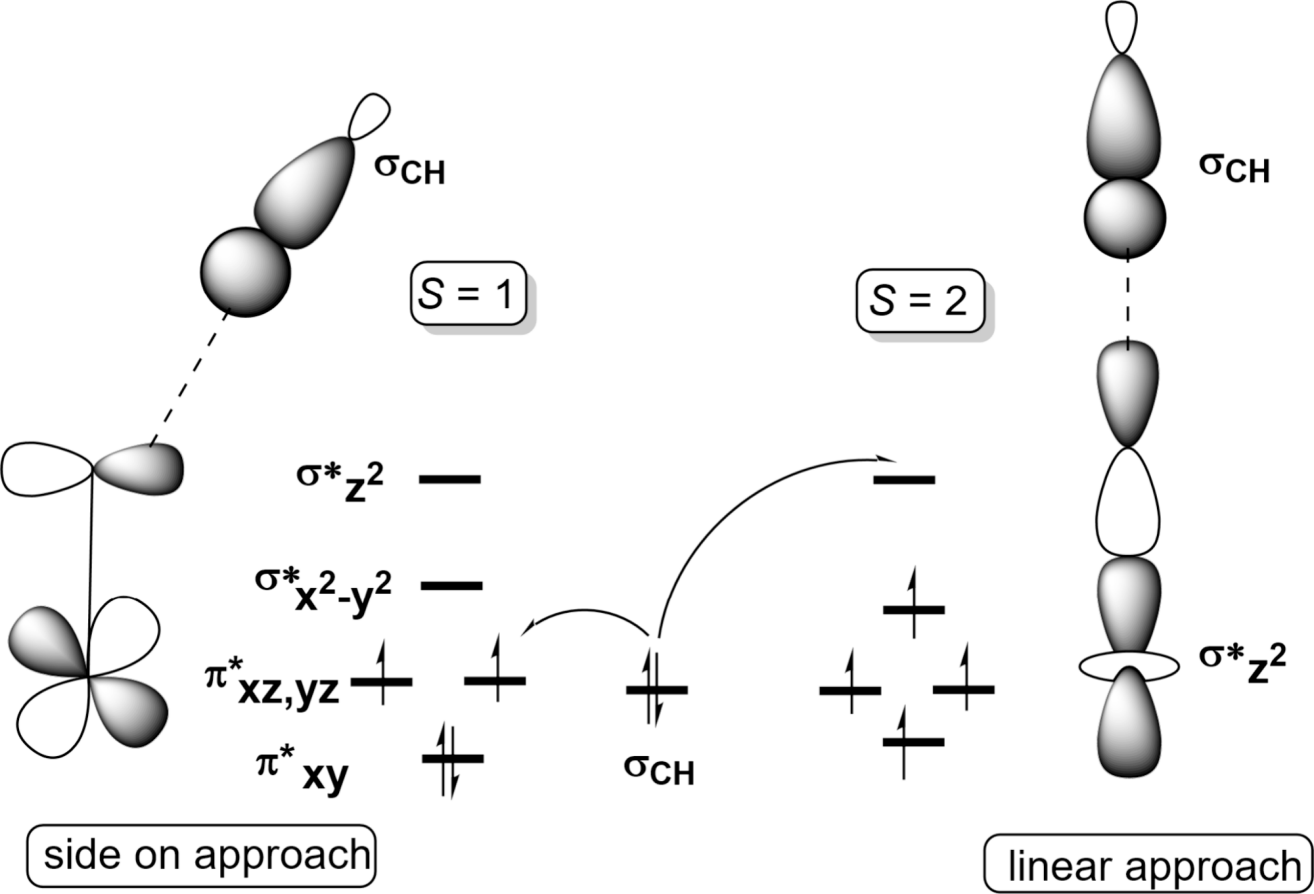
Illustration of the concept of π (left) vs σ (right) reactivity channels for the HAA by Fe­(IV)–oxo complexes.

##### 2.4.2.2 Two-State Reactivity

The two-state reactivity (TSR) concept, first proposed by Shaik, is based on the consistent observation from computational chemistry that the *S* = 2 state of a Fe­(IV)–oxo complex has a lower barrier for HAA than the *S* = 1 state of the same complex.^324–327^ So for complexes that have an *S* = 1 (triplet) ground state, their reactivity for HAA can therefore be greatly enhanced if the Fe­(IV)–oxo complex can access the *S* = 2 (quintet) state along the HAA reaction coordinate. This, in turn, depends on how these energy surfaces intersect, and how well the complex can undergo the required intersystem crossing. The TSR model therefore applies when the triplet and quintet energy surfaces cross, as shown in Figure 30, leading to reduced reaction barriers and facilitating efficient HAA for *S* = 1 Fe­(IV)–oxo complexes that have this property. Experimentally, however, this is difficult to prove, because this would require comparing the substrate oxidation rate of the same complex in the two different spin states, to first demonstrate the underlying assumption that the *S* = 2 state is, in fact, more reactive (has faster reaction rates). Recently, Que and co-workers have reported a Fe­(IV)–oxo intermediate, which shows a temperature-dependent spin transition from *S* = 2 (233 K) to *S* = 1 (150 K) (see below).^328^ However, since it is very difficult to run reactions at 150 K, this direct comparison is still challenging. Nevertheless, these results provide a path forward for how such a comparison could potentially be made in the future.

**Figure 30:**
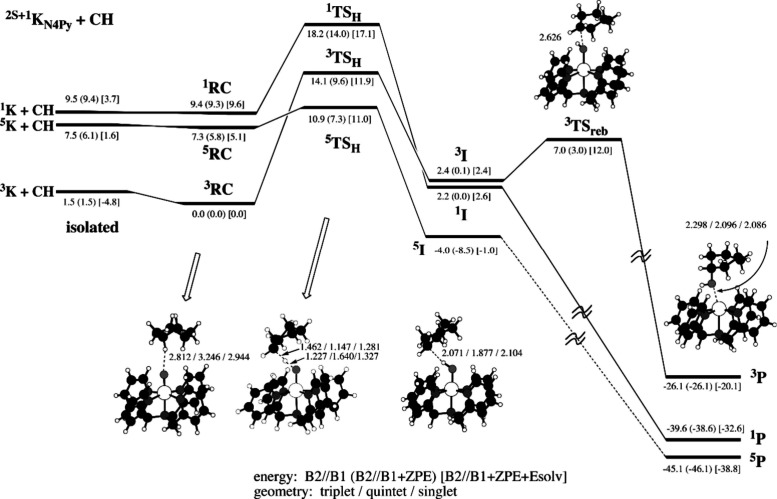
Energy profile for the reaction of ^2S+1^ *K*_N4Py_ (*S* = 0, 1, 2), the [Fe­(N4Py)­(O)]^2+^ intermediate, with cyclohexane (CH). Relative energies are indicated in the order: B2//B1 (B2//B1 + ZPE) [B2//B1 + ZPE + *E*_solv_]. Key bond lengths (in Å) are noted for the species in different spin states, in the order: triplet/quintet/singlet. Reproduced with permission from ref 324. Copyright 2006 American Chemical Society.

Despite extensive experimental and theoretical attention to the TSR concept, a direct experimental measurement of the triplet-to-quintet excitation energy in Fe­(IV)–oxo complexes had long remained elusive. In a recent study, DeBeer, Neese and co-workers reported that combining MCD spectroscopy with 2p3d RIXS allowed for experimental access to the low-energy spin-forbidden triplet-to-quintet transition of Fe­(IV)–oxo complexes, using the pentadentate ligand frameworks N2Py2Q, N2Py2P*, and N4Py.^329^ The lower the energy of the ^5^A_1_ state, the easier it is to cross from the ground state triplet surface to the quintet surface. One would therefore expect a correlation between the energies of the ^5^A_1_ state and the second-order rate constants for HAA reactions. For the first time, MCD identified a weak, narrow optical band in the 5,000–10,000 cm^–1^ energy range, identified with this transition. In addition, 2p3d RIXS allows for the assignment of electronic transitions below 10,000 cm^–1^, and this method was used as well to assign the triplet-to-quintet ligand field transitions in the three complexes. The energy of this transition increases from 0.45 eV (N2P2Q) < 0.86 eV (N2Py2P*) < 0.94 eV (N4Py), which is in good agreement with the calculated values of 0.31 eV (N2Py2Q) < 0.91 eV (N2Py2P*) < 0.95 eV­(N4Py). With the experimental transition energies in hand, it became possible to directly correlate the triplet–quintet energy gaps with observed rate constants. These results indicate a correlation between the previously reported^330,331^ second-order rate constants for cumene oxidation by the three complexes and their triplet-quintet energy gaps.

Recent work by Solomon and co-workers further investigated the two-state reactivity for HAA by Fe­(IV)=O complexes.^332^ In this study, the *S* = 1 Fe­(IV)–oxo complex [Fe­(TMC)­(O)­(CH_3_CN)]^2+^ was probed using Fe 1s2p RIXS and Fe L-edge XAS. Analysis of the spectroscopic data within a ligand-field multiplet model demonstrated that the ground-state wave function of [Fe­(TMC)­(O)­(CH_3_CN)]^2+^ is comprised of an admixture of *S* = 1 and *S* = 2 states (∼13% *S* = 2 state). Deconvolution of the constant energy cuts of the Fe 1s2p RIXS data yielded features resulting from promotion of an electron into the 3d manifold derived from these two different states (Figure 31; green transitions *S* = 1 states, red transitions *S* = 2 states). Owing to the fact that the Fe­(1s) hole has negligible interaction with the 3d orbitals, the deconvoluted pre-edge energies correlate with the energetics of electron transfer into the 3d-manifold following HAA. Thus, these energies provide an experimental handle on the HAA reaction energies promoted by [Fe­(TMC)­(O)­(CH_3_CN)]^2+^. The low-energy *S* = 1 transition corresponds to excitation into the Fe­(3d_*xz*_) orbital, which would be the acceptor orbital for a substrate being oxidized in a π-type fashion along the complex’s equatorial plane. In contrast, the low-energy *S* = 2 transition corresponds to excitation into the Fe­(3d_z2_) orbital, which would be the acceptor orbital for a substrate being oxidized in an axial σ-type fashion. What the data show is that an *S* = 2 pathway is predicted to be low in energy and nearly isoenergetic (Δ*E* = +0.2 eV) with an *S* = 1 pathway (see Figure 31). Thus, both are energetically accessible reaction channels. Furthermore, the reaction coordinate is different if the reaction is taking place along the *S* = 1 or *S* = 2 surface.

**Figure 31:**
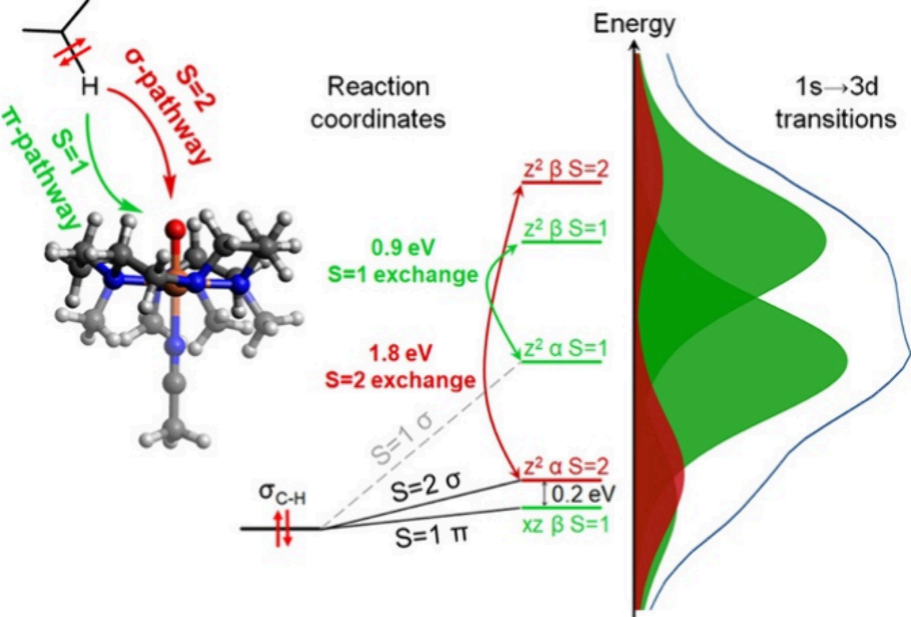
HRFD-XANES resolved Fe­(1s → 3d) transitions for [Fe­(TMC)­(O)­(CH_3_CN)]^2+^, which reflect the energies of HAA along the oxo axial or equatorial vectors. The sterically preferred axial σ-pathway on the *S* = 2 surface (acceptor orbital d_*z*_^2^ α, red) is only 0.2 eV above the equatorial π-pathway (acceptor orbital d_*xz*_ β, green) on the *S* = 1 surface, meaning both *S* = 2 and *S* = 1 surfaces are thermally accessible reaction pathways. Reproduced with permission from ref 332. Copyright 2024 American Chemical Society.

##### 2.4.2.3 Transition-State Asynchronicity

An expansive and growing literature on the HAA mechanism of transition metal–oxo complexes has uncovered a multifaceted complexity of the transition state landscape, and identified how the observed rates for C–H bond oxidation relate to the underlying thermodynamic driving forces of the process.^309^ It is well established that C–H bond activation generally follows the Bell–Evans–Polanyi (BEP) principle, with reaction rates increasing as the C–H bond strength decreases for a given metal–oxo complex.^309^ In a true HAT event, both the proton and electron from an X–H bond are transferred in a single step to the same acceptor (e.g., reactivity of phenols and radical species, R^•^, forming PhO^•^ and RH as the products; see Figure 32). On the other hand, in a PCET process, the proton and electron end up on different atomic sites or ligands in a metal–oxo intermediate. As frequently observed in transition metal–oxo mediated oxidation reactions, the electron may go to the metal center, while the H^+^ migrates to a ligand or the oxo group of the complex. HAT and PCET can occur through a concerted mechanism where both the electron and proton are transferred simultaneously, or they can proceed in an asynchronous manner where the reactions have more/less proton and electron transfer character in the transition state (see Figure 32). In the most extreme case, the reactions could proceed in a stepwise manner, with either ET or PT occurring first, followed by the respective other process.

**Figure 32:**
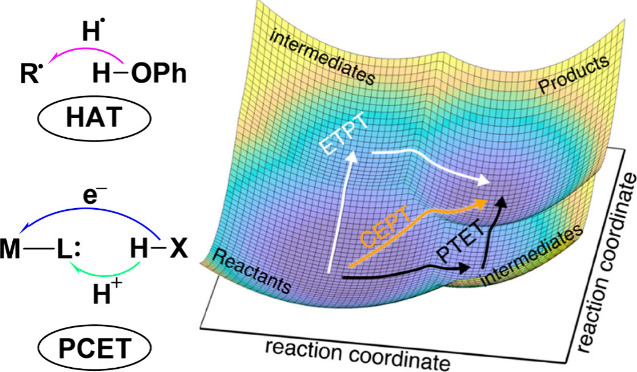
Left: The generic representations of HAT (the proton (H^+^) is transferred along with its electron (e^–^)) and PCET (where the proton and the electron are transferred to different locations in the acceptor). Right: Illustration of the three main mechanisms for PCET, each with a distinct transition state. (ET = electron transfer, PT = proton transfer, CEPT = concerted electron and proton transfer). Reproduced with permission from ref 333. Copyright 2021 American Chemical Society.

This idea constitutes the basis for the transition state asynchonicity model by Srnec and co-workers.^334^ In fact, in many cases, HAT/PCET is not a perfectly concerted process, meaning the proton and electron do not transfer simultaneously or to the same degree in the transition state. Notably, this asynchronicity can significantly influence the activation barrier and reaction pathway of an HAA reaction (see Figure 32). For example, a reaction may proceed via an initial electron transfer followed by a proton transfer, or vice versa, depending on the nature of the substrate and the iron–oxo species. This asynchronicity can stabilize or destabilize the transition state and thus modulate the HAA rate (see Figure 33). Srnec and co-workers recently introduced a third thermodynamic component in HAA reactions—frustration,^335^ which arises from the mismatch between the oxidant and the substrate in their relative abilities to accept a proton and an electron (p*K*_a_ and reduction potential). Although this frustration-based model has not yet gained wide adoption in the field for explaining reactivity trends, Srnec’s earlier concept of asynchronicity has started influencing the field, and experimental verification of this concept is starting to emerge. For example, Borovik and co-workers examined the HAA mechanism by their [Mn^IV^(H_3_buea)­(O)]^−^ complex, when reacted with C–H bonds. They evaluated multiple mechanistic possibilities to reconcile the experimental kinetic and thermodynamic observations. The absence of a linear relationship between log­(*k*_2_) (with *k*_2_ being the second-order rate constant for C–H bond activation for a given substrate) and the substrates’ C–H BDFE excluded synchronous PCET as a feasible pathway. Similarly, a purely electron-transfer-limited mechanism was ruled out, because of the observed correlation of log­(*k*_2_) with substrate p*K*_a_, which implicates proton transfer in the rate-determining step. Based on these findings, the authors proposed a basic, asynchronous PCET mechanism, where proton transfer predominates the transition state for C–H bond activation.^336^

**Figure 33:**
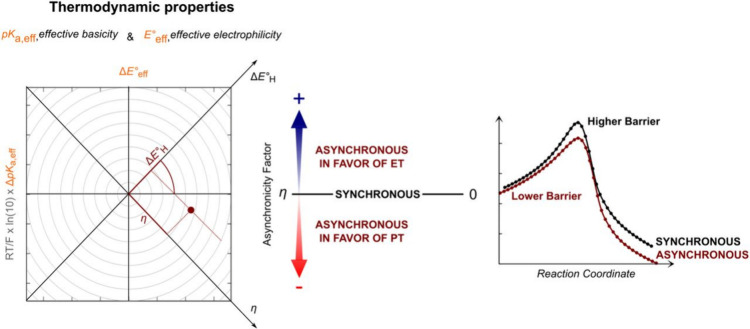
Left: Differences in effective redox potential and p*K*_a_ between the oxidant and the substrate determine the factor η that quantifies the propensity for asynchronicity in a PCET reaction, as shown in the middle panel. Right: η further correlates with the height and width of the reaction barrier. Reproduced with permission from ref 334. Copyright 2018 National Academy of Science.

#### 2.4.3 Rebound Mechanism

The stepwise events of HAA and radical recombination in the rebound mechanism represent a general strategy exploited by nature to perform controllable radical reactions. Groves first introduced the radical rebound mechanism in 1976,^337^ and since then, it has become widely accepted as a central mechanistic paradigm in enzymatic C–H functionalization reactions and in the action of biomimetic small-molecule catalysts (see Figure 34). In both enzymatic systems and synthetic models, the mechanism typically begins with the abstraction of a hydrogen atom from the substrate (R–H) by a high-valent metal–oxo species (M^n+^–O), resulting in the formation of a substrate-centered radical and a reduced iron hydroxide species, [M^(n‑1)+^–OH ^•^R]. This transient, ″caged″ radical pair then proceeds along a complex potential energy surface, giving rise to multiple possible reaction pathways. One of the primary outcomes is the recombination of the substrate radical with the hydroxyl ligand—commonly referred to as ″oxygen rebound″—to yield the hydroxylated product (R–OH). The defining hallmark of the radical rebound mechanism is the formation of the substrate radical intermediate, which arises from the initial HAA step.^338^ Radical rebound is not only about adding hydroxyl groups, it can also involve adding chlorine, azide, or nitrite. In Fe­(II)/α-KG halogenase enzymes, for example, a chloroferryl intermediate (Cl–Fe^IV^=O) first takes a hydrogen atom from the substrate, creating a carbon radical and a chlorohydroxoferric intermediate (Cl–Fe^III^–OH)). The radical then quickly recombines with the iron-bound chloride to produce a chlorinated product.^199^ Bollinger and co-workers also found that Fe­(II)/α-KG halogenases could catalyze C–H azidation and nitration upon replacing chloride with azide or nitrite.^339^

The properties and behavior of the incipient substrate radical (i.e., lifetime and conformational degrees of freedom) and the physical and chemical characteristics of the radical rebound step (i.e., rate constant) are of crucial importance for understanding the reaction outcomes (especially selectivity) when high-valent metal–oxo complexes react with C–H bonds in complex organic molecules (see Figure 34). However, the transient nature of the substrate radical and the large rate of the radical rebound step has generally precluded direct mechanistic studies with common kinetic and spectroscopic methods. In this regard, mechanistically diagnostic substrates, which form radicals that change stereochemistry or structure with a defined rate after HAA, offer a powerful tool to study the intermediate radical and the rebound step. The first radical rearrangement studies to probe biologically relevant C–H hydroxylation used norcarane as a mechanistically diagnostic substrate (a “radical clock”) and manganese porphyrins as the catalyst.^340^ The application of norcarane to a variety of Cyt P450s gave radical rebound rates in the range between 10^10^ and 10^11^ s^–1^, corresponding to radical lifetimes in the picosecond regime.^341^ Similar radical-clock studies have also been performed on a variety of nonheme iron-containing enzymes, which showed large variations in substrate radical lifetimes. For instance, radical lifetimes for diiron-containing sMMO,^342–344^ Toluene 4-Monooxygenase (T4moH)^345,346^ and Alkane Hydroxylase AlkB^347–349^ were determined to be 20 ps, 263 ps, and 1 ns, respectively. A very long radical lifetime (11 ns) was observed for monooxygenation reactions catalyzed by NDO, which belongs to the family of Rieske dioxygenases.^70^

**Figure 34:**
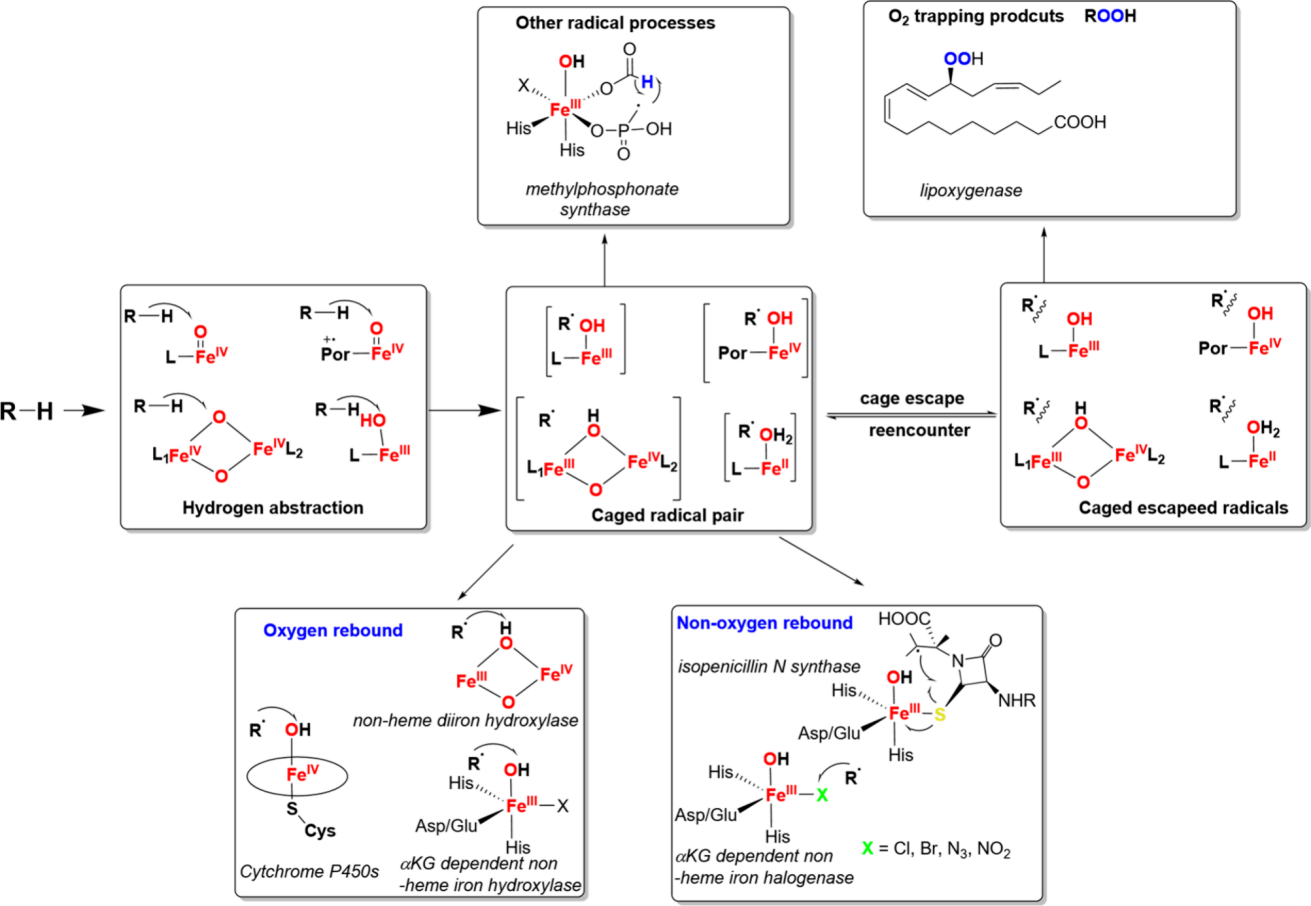
Generation of the substrate radical via HAA by a high-valent metal–oxo intermediate and the various reaction outcomes depending on the behavior of the radical. Adapted with permission from ref 338. Copyright 2017 Springer.

Goldberg and co-workers recently investigated a series of nonheme [Fe^III^(BNPAPh_2_O)­(OH)­(X)] (X = Cl^–^, Br^–^) complexes to probe radical rebound selectivity with trityl-type carbon radicals.^350^ When reacted with tertiary carbon radicals, these Fe^III^(OH)­(X) complexes undergo selective OH^•^ rebound, yielding alcohols exclusively. Surprisingly, halogen rebound was not observed, even though DFT calculations suggested that the barrier for halogen transfer may be lower. This selectivity is attributed to the endergonic nature of the halogenation pathway, which creates a nonproductive equilibrium favoring reactants. In contrast, reactions with secondary carbon radicals favor X^•^ transfer, since the halogenated product is thermodynamically more stable than the reactant, making halogen rebound favorable.^351^ The study was further extended to Fe^III^(OH)­(X) complexes with X = N_3_^–^, NCS^–^, NCO^–^, and F^–^ ligands using various trityl radical substrates. These results demonstrate that rebound selectivity can also vary with the nature of both the radical and the ligand X.^352,353^

## 3 Synthetic Terminal Iron–Oxo Complexes (Emphasizing Results from the Last 5 Years)

Over the last two decades, there has been a significant surge in research on dioxygen-activating nonheme iron enzymes and their corresponding model complexes. This review aims to highlight recent developments, particularly over the last five years, in the field of biomimetic analogs of high-valent iron–oxo intermediates. Detailed reviews are available that summarize the earlier results.^159,354–362^ Biomimetic, quasi-stable nonheme iron–oxo complexes, which emulate the reactive intermediates found in enzymatic systems, have only been synthesized and thoroughly studied since the early 2000s. Today, over a hundred examples have been reported, but only ∼10% of these Fe­(IV)–oxo species have been structurally characterized via X-ray crystallography (see Figure 35 for examples). These structural insights have provided a strong foundation for understanding the geometric and electronic properties of the critical Fe­(IV)–oxo intermediates in corresponding nonheme iron enzymes.

**Figure 35:**
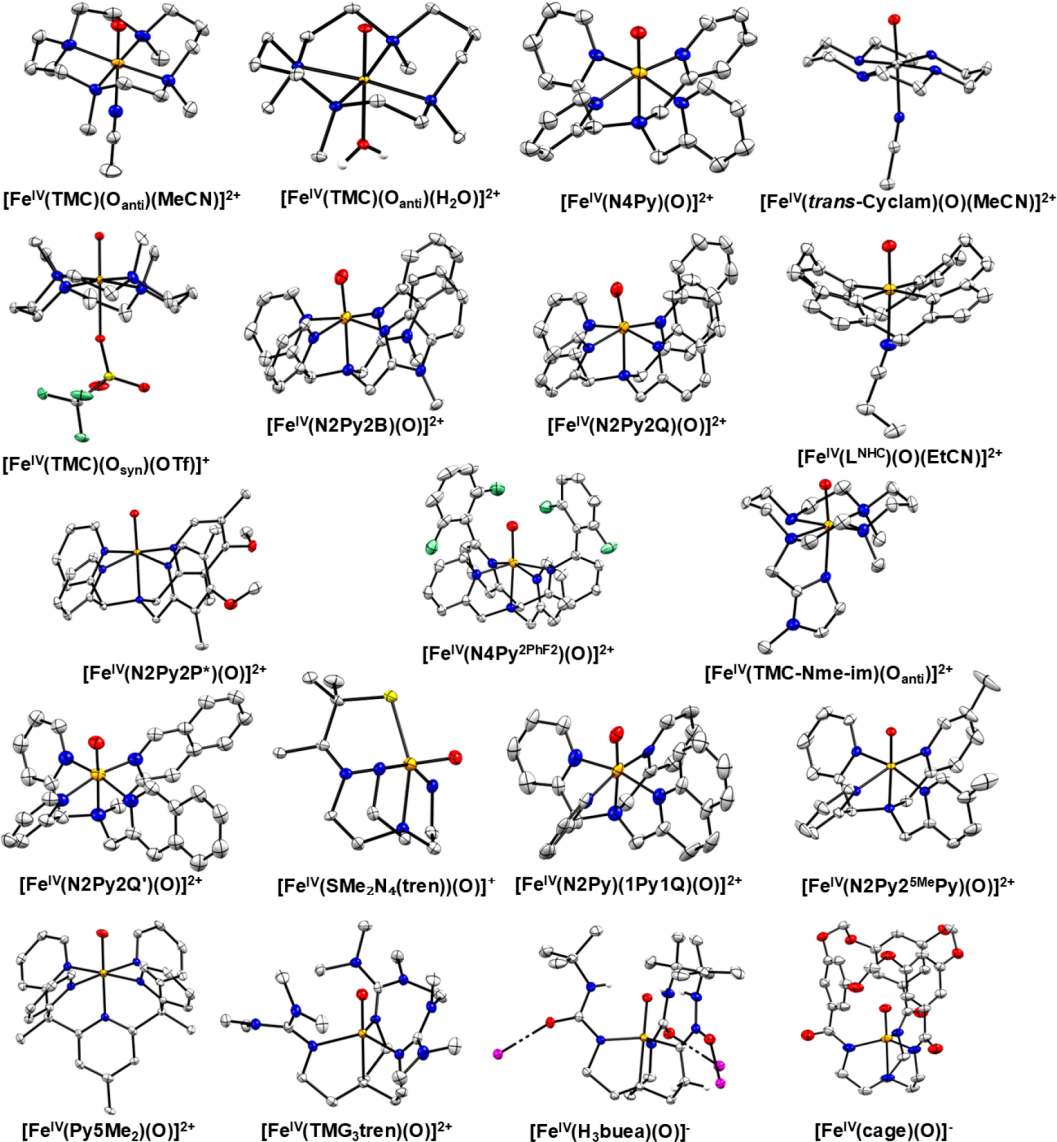
A collection of crystal structures of synthetic Fe­(IV)–oxo complexes obtained by X-ray crystallography.

In 2000, Borovik and co-workers reported the first nonheme Fe­(III)–oxo complex, supported by the tripodal ligand tris­[(*N*′-*tert*-butylureaylato)-*N*-ethyl]­aminato, H_3_buea^3−^, using dioxygen.^363^ In the same year, Wieghardt and co-workers first generated a mononuclear nonheme Fe­(IV)–oxo complex by the ozonolysis (using ozone as an O atom transfer agent) of [Fe^III^(cyclam-CH_2_CO_2_)­(CF_3_SO_3_)]^+^ (cyclam-CH_2_CO_2_^−^= 1,4,8,11-tetraazacyclotetradecane-1-acetate) in a solvent mixture of acetone/water at −80 °C.^364^ The metastable intermediate was shown to be a low-spin (*S* = 1) iron­(IV)–oxo species based on Mössbauer data (δ = 0.1 mm s^–1^ and Δ*E*_Q_ = 1.39 mm s^–1^). The instability of the compound prevented additional spectroscopic studies. However, the first observation of an Fe­(IV)–oxo compound likely occurred in 1998 when R. Ho from the Que laboratory tried the reaction of [Fe^II^(TPA)­(MeCN)_2_]^2+^ (TPA = tris­(2-pyridylmethyl)­amine) with a stoichiometric amount of peracid in MeCN at −40 °C. This reaction generated a transient absorbance band at 720 nm albeit with low intensity (ε ≈ 200 M^–1^ cm^–1^), assuming complete conversion of the precursor complex to the new species.^358^ At the time, the nature of the intermediate was unclear, and it was not suspected to be an Fe­(IV)–oxo species, as the expectation was that such a complex would display a strong oxo-to-iron­(IV) charge transfer band in the visible range.^358^ The first crystal structure of an Fe­(IV)–oxo complex, [Fe^IV^(TMC)­(O)­(MeCN)]^2+^, was later reported by Que and Nam using the N-tetramethylated cyclam ligand (1,4,8,11-tetramethyl-1,4,8,11-tetraazacyclotetradecane, TMC) in 2003.^205^ Coincidentally, that same year saw the trapping and characterization of the first enzymatic Fe­(IV)–oxo intermediate in TauD (Intermediate *J*, see above).^24^ The crystal structure of the TMC complex revealed an Fe–O bond length of 1.646(3) Å, supported by the macrocyclic TMC ligand, with all four methyl groups oriented on one side of the macrocycle, opposite to the oxo group (called “O_anti_” form, see Figure 35). In 2007, the Collins group reported the first Fe­(V)–oxo intermediate in a tetraamido (TAML^4–^) ligand framework.^365^

Following these groundbreaking discoveries, iron–oxo chemistry rapidly gained momentum. Back-to-back contributions from Que, Nam, Borovik, Costas, Comba, Ray, Goldberg, and scientists worldwide significantly expanded this field. Their efforts led to the generation of over 100 new Fe­(IV)–oxo complexes, including some with an *S* = 2 spin state, reminiscent of enzymatic nonheme iron systems. Several of these complexes have also been structurally characterized. Our discussion examines a few important early examples and mainly focuses on discoveries made in the last five years. Over the past decade, numerous groundbreaking discoveries have been made in the chemistry of Fe­(IV)–oxo complexes. One of the most notable findings was the synthesis of a highly reactive *S* = 2 Fe­(IV)–oxo species supported by a tripodal quinoline ligand framework.^366^ Solomon and co-workers characterized an *S* = 2 Fe­(IV)–oxo species within an iron-containing zeolite that demonstrated the ability to convert methane to methanol.^367^ More recently, Kojima and co-workers reported an *S* = 1 Fe­(IV)–oxo complex capable of catalytically converting methane to methanol in an aqueous medium.^368^ In another significant advancement, Long and co-workers identified an *S* = 2 Fe­(IV)–oxo center within a metal–organic framework (MOF), which catalyzes the oxidation of cyclohexane to cyclohexanol using molecular oxygen.^369^ Most recently, the Barnet group unveiled the most stable Fe­(IV)–oxo species reported to date, confined within a molecular cage.^317^ These discoveries, from tripodal ligands to zeolites, MOFs, and supramolecular cages, underscore the rapid progress and versatility of Fe­(IV)–oxo chemistry in recent years.

Besides these complexes, new pathways have been reported in the literature for generating Fe­(IV)–oxo species, via small-molecule activation reactions.^370^ For example, Chang and co-workers generated an Fe­(IV)–oxo intermediate in the tpa^Mes^ ligand framework using N_2_O as an O atom donor. Very recently, Meier, Lehnert and co-workers reported direct NO reduction to N_2_O at a single iron site via formation of an Fe­(IV)–oxo intermediate, using the H_3_buea ligand framework.^371^ In this case, the high-valent intermediate is generated from an Fe­(II) precursor and NO gas.

### 3.1 Structure and Reactivity of Terminal Fe­(IV)–Oxo Complexes

Figure 35 shows a structural overview of different Fe­(IV)–oxo complexes, which are further discussed in the following. We have divided this section into three major parts, based on both spin-states and the nature of the coligand scaffolds.

#### 3.1.1 Synthetic *S* = 1 Fe­(IV)−Oxo Complexes with Macrocyclic Ligands

In 2020, Nam, Ray and co-workers reported the isolation and spectroscopic characterization of an Fe­(IV)–oxo species, *trans*-[Fe^IV^(cyclam)­(O)­(MeCN)]^2+^, which efficiently catalyzes olefin epoxidation with high stereo- and regioselectivity.^393^ The corresponding Fe­(II) complex is stable in air in acetonitrile, but it reacts with O_2_ in acetone at −20 °C to form the Fe­(IV)–oxo complex as confirmed by X-ray crystallography (see Figure 35). This complex is much more stable compared to the corresponding *cis* isomer.^392^ This complex has two equally intense UV–vis absorption features at 583 and 690 nm, and a Mössbauer quadrupole doublet with δ = 0.05 mm/s and Δ*E*_Q_ = 2.49 mm/s, which is similar to other reported *S* = 1 Fe­(IV)–oxo complexes (Table 3). Using EXAFS, the Fe–O distance was determined to be 1.67 Å and the Fe–O stretching frequency was observed at 842 cm^–1^ (Δ^18^O = −36 cm^–1^) from rRaman. Notably, *trans*-[Fe^IV^(cyclam)­(O)­(MeCN)]^2+^ represents a rare example of an Fe­(IV)–oxo intermediate that selectively favors epoxidation over hydroxylation. However, the previously reported *cis* isomer is more reactive compared to the *trans* isomer for HAA; for instance, the DHA oxidation second-order rate constant of 1.3 M^–1^s^–1^ at −40 °C for the *cis* isomer is much larger than that for the *trans* isomer, which is 4.9 × 10^–2^ M^–1^s^–1^ at 20 °C.

**Table 3:** Spectroscopic Properties of Synthetic *S* = 1 Macrocycle-Based Fe­(IV)–oxo Complexes (See Scheme 7 for Structural Drawings)

complex^a^,^b^	λ_max_ (nm) [ε(M^–1^ cm^–1^)]	r(Fe=O) (Å)	ν(Fe–O) (cm^–1^)	δ (mm/s)	Δ*E*_Q_ (mm/s)	half-life (298 K)	ref
[Fe^IV^(TMC)(O_anti_)(MeCN)]^2+^	824 (400)	1.646	839	0.17	1.24	10 h	^204,205,208,372–375^
[Fe^IV^(TMC)(O_anti_)(H_2_O)]^2+^	820 (250)	1.650	840				^374,376^
[Fe^IV^(TMC)(O_anti_)(OTf)]^+^		1.666		0.20	1.87		^374^
[Fe^IV^(TMC)(O_anti_)(CF_3_CO_2_)]^+^	836 (250)	1.64	854	0.20	1.39	1 h	^208,372,373^

[Fe^IV^(TMC)(O_anti_)(OAc)]^+^	831 (160)	1.65	826	0.19	0.99	3 min	^377^
	997 (140)						

[Fe^IV^(TMC)(O_anti_)(OPr)]^+^	837 (∼160)					3 min	^377^
	1030 (∼140)						

[Fe^IV^(TMC)(O_anti_)(NCO)]^+^	845 (150)	1.67	822	0.16	0.42	30 min	^208,372^
	1010 (130)						

[Fe^IV^(TMC)(O_anti_)(NCS)]^+^	850 (200)	1.65	820	0.18	0.60	30 min	^208,372^
	1010 (170)						

[Fe^IV^(TMC)(O_anti_)(N_3_)]^+^	850 (130)	1.66	814	0.17	0.70	15 min	^208,372^
	1050 (110)						

[Fe^IV^(TMC)(O_anti_)(CN)]^+^	858 (250)	1.66	823	0.15	0.25	1.5 h	^208^

[Fe^IV^(TMC)(O_anti_)(OH)]^+^	830 (100)	1.68	803	0.15	0.16	seconds	^208,376^
	1060 (110)						

[Fe^IV^(TMC-py)(O_anti_)]^2+^	834 (260)	1.667	826	0.18	1.08	7 h	^378,379^
[Fe^IV^(TMC-NMe-im)(O_anti_)]^2+^	814 (290)	1.655	828				^380^
[Fe^IV^(TMC-dma)(O_anti_)]^2+^	810 (270)	1.658	833	0.13	1.1	120 h	^379^

[Fe^IV^(TMC-dmaH^+^)(O_anti_)]^+^	588 (460)	1.67	823	0.13	0.51	1.5 h (273 K)	^379^
	806 (270)						

[Fe^IV^(TMCAc)(O_anti_)]^+^	811 (180)	1.64	831	0.13	0.74	48 h	^377^
	962 (160)						

[Fe^IV^(TMCPr)(O_anti_)]^+^	831 (135)	1.65	822	0.17	0.82	4 h	^377^
	1033 (145)						

[Fe^IV^(TMCS)(O_anti_)]^+^	460 (1300)	1.70		0.19	–0.22	5 min	^208,381,382^
	570 (1100)						

[Fe^IV^(TMC–SO_2_)(O_anti_)]^+^	830 (170)	1.64	831	0.19	1.28	∼hours	^381^
[Fe^IV^(TMC)(O_syn_)(OTf)]^+^	815 (380)	1.625	856	0.16	1.55	2 h	^383,384^
[Fe^IV^(TMC-HOR)(O_syn_)(MeCN)]^+^	820 (320)	1.65	862	0.13	1.25		^385^
[Fe^IV^(Me_2_EBC)(O)(MeCN)]^2+^	800 (270)	1.64	824	0.13	0.60	5 h (273 K)	^386^
[Fe^IV^(13-TMC)(O_syn_)(OTf)]^+^	735 (240)		833	0.12	1.98	30 min (233 K)	^387,388^
[Fe^IV^(13-TMC)(O_syn_)(NCS)]^+^	755 (160)		848	0.07	1.34		^389^
[Fe^IV^(15-TMC)(O_anti_)]^2+^	890					1 min (233 K)	^390^

[Fe^IV^(TMCO)(O_anti_)(OTf)]^+^	585 (200)	1.64		0.21	1.58	1.5 h (223 K)	^391^
	848 (130)						
	992 (125)						

[Fe^IV^(TMCN-d_12_)(O_syn_)]^+^	820 (300)					8 min (273 K)	^391^
[Fe^IV^(cyclam-CH_2_CO_2_)(O_anti_)]^+^	676			0.01	1.37		^364^
*cis*-[Fe^IV^(cyclam)(O)(MeCN)]^2+^	737 (230)	1.66		0.10	1.09	3 min (253 K)	^392^

*trans*-[Fe^IV^(cyclam)(O)(MeCN)]^2+^	583 (200)	1.67	842	0.05	2.49	2 h	^393^
	690 (200)						

*trans*-[Fe^IV^(dithiacyclam)(O)(MeCN)]^2+^	596 (226)		794	0.13	1.21	2.7 h (208 K)	^394^
	815 (549)						

[Fe^IV^(15-cyclam)(O)(L)]^2+^	750 (500)		841			1 h	^390^

[Fe^IV^(TBC)(O_anti_)(MeCN)]^2+^	885 (360)	1.64	842	0.22	0.97	1.5 h	^395^
	945 (190)						

[Fe^IV^(TBC)(O_syn_)(MeCN)]^2+^	865 (380)		852	0.21	1.11	23 min	^396^
[Fe^IV^(TB^F8^C)(O_anti_)(MeCN)]^2+^	885 (400)		842	0.24	0.87	6 min	^396^
[Fe^IV^(TB^F8^C)(O_syn_)(MeCN)]^2+^	862 (400)		855	0.21	1.27	2 min	^396^

[Fe^IV^(TB^F8^C)(O_syn_)(Cl)]^+^	890 (160)		837	0.23	0.97	1.5 min (253 K)	^396^
	1070 (220)						

[Fe^IV^(PyMAC)(O)]^2+^	705 (∼240)			0.03	2.0	2–3 min	^397^

[Fe^IV^(PyNMe_3_)(O)]^2+^	792	1.66	822	0.07	0.98		^398^
(oxo trans to amine)	970						

[Fe^IV^(PyNMe_3_)(O)]^2+^	805 (230)	1.65	829	0.09	0.24		^398^
(oxo trans to pyridine)	990 (320						

[Fe^IV^(LNHC)(O)(RCN)]^2+^	400 (∼200)	1.661		–0.13	3.08	∼5 h	^399^
[Fe^IV^(TAML)(O)]^2–^	435 (2500)	1.69		–0.19	3.95	>2 h (10%)	^400^

a For the TMC complexes, there are two isomers. *Syn* Isomer: the oxo ligand is located on the same side as the four methyl substituents of the TMC macrocycle. *Anti* Isomer: the oxo ligand is located on the opposite face as the four methyl groups of the TMC macrocycle.

b Ligand nomenclature: TMC = 1,4,8,11-tetramethyl-1,4,8,11-tetraazacyclotetradecane; TMCS^–^ = 1-mercaptoethyl-4,8,11-trimethyl-1,4,8,11-tetraaza cyclotetradecane; TMC-py = 1-(2′-pyridylmethyl)-4,8,11-trimethyl-1,4,8,11-tetraazacyclotetradecane; TMCO = 4,8,12-trimethyl-1-oxa-4,8,12-triazacyclotetradecane; 13-TMC = 1,4,7,10-tetramethyl-1,4,7,10-tetraazacyclotridecane; TMCN-*d*_12_ = 1,4,7,11-tetra­(methyl-*d*_3_)-1,4,7,11-tetraazacyclotetradecane; 15-TMC = 1,4,8,12-tetramethyl-1,4,8,12-tetraazacyclopentadecane; Me_2_EBC = 4,11-dimethyl-1,4,8,11-tetraazabicyclo[6.6.2]­hexadecane; TMC-HOR = 2-(4,8,11-trimethyl-1,4,8,11-tetraazacyclotetradecan1-yl)­ethan-1-ol; cyclam = 1,4,8,11-tetraazacyclotetradecane; 15-cyclam = 1,4,8,12-tetramethyl-1,4,8,12-tetraazacyclopentadecane; TBC = 1,4,8,11-tetrabenzyl-1,4,8,11-tetraazacyclotetradecane; TB^F8^C = 1,4,8,11-tetra­(2,6-difluorobenzyl)-1,4,8,11-tetraazacyclotetradecane; PyMAC = 2,7,12-trimethyl-3,7,11,17-tetra-azabicyclo[11.3.1]­heptadeca-1(17),13,15-triene; PyNMe_3_ = 3,6,9-trimethyl-3,6,9-triaza-1­(2,6)-pyridinacyclodecaphane; LNHC = 3,9,14,20-tetraaza-1,6,12,17-tetraazoniapenta-cyclohexacosane-1(23),4,6(26),10,12(25),15,17(24),21-octaene tetrakis-trifluoromethanesulfonate; TAML^4–^ = 5,6-benzo-3,8,11,13-tetraoxo-2,2,9,9-tetramethyl-12,12-diethyl-1,4,7,10-tetraazacyclotridecane; Dithiacyclam = 1,8-dithia-4,11-diazacyclotetradecane

In 2021, Apfel, Ray and co-workers reported the reactivity of a new oxo complex, *trans*-[Fe^IV^(dithiacyclam)­(O)­(MeCN)]^2+^, which features a modified cyclam ligand where two nitrogen atoms in the ring are replaced by sulfur.^394^ This new intermediate has two absorption bands at 596 and 815 nm and displays isomer shift (0.13 mm/s) and quadrupole splitting (1.21 mm/s) values that are similar to those reported for other *S* = 1 Fe­(IV)–oxo complexes (Table 3). The Fe–O stretching frequency of this complex was observed at 793 cm^–1^ by rRaman spectroscopy, which is shifted to lower frequency by 49 cm^–1^ compared to the previously studied *trans*-cyclam complex. The introduction of the equatorial sulfur ligands also affects the reactivity of the intermediate for the HAA reaction. It was found that *trans*-[Fe^IV^(dithiacyclam)­(O)­(MeCN)]^2+^ is at least 3–4 orders of magnitude faster (in terms of the rate constant) than the *trans*-[Fe^IV^(cyclam)­(O)­(MeCN)]^2+^ complex in HAA. The second-order rate constant for DHA oxidation is 0.347 M^–1^s^–1^ at −80 °C for the *trans*-dithiacyclam complex, whereas the corresponding rate constant for the *trans*-cyclam complex is only 4.9 × 10^–2^ M^–1^s^–1^, even at 20 °C.

Que, Swart and co-workers reported on the difference in reactivity between the two isomers of [Fe^IV^(TMC)­(O)­(MeCN)]^2+^, *anti* and *syn*, which differ in the location of the oxo group relative to the methyl groups of the TMC ligand (see Table 3).^384^ The *syn* isomer exhibits HAA rates that are 1.5–3 times faster and OAT rates that are 100–1000 times faster than those of the *anti* isomer. DFT calculations revealed that the significant reactivity difference between the two isomers can be linked back to the 0.02 Å shorter Fe–O bond found for the *syn* isomer, which allows for an increased out-of-plane dislocation of the Fe=O unit, resulting in dissociation of the axial MeCN ligand and a highly reactive five-coordinate Fe­(IV)–oxo intermediate.^384^

Ray and co-workers also reported the reactivity of an Fe­(IV)–oxo complex with a new TMC ligand, where a pendant alcohol unit is attached to one of the nitrogen arms of TMC.^385^ The authors found that the alcohol moiety stays protonated and does not axially coordinate to iron. Mössbauer data for the oxidized intermediate show an isomer shift (δ = 0.13 mm/s) and quadrupole splitting (Δ*E*_Q_ = 0.25 mm/s) that suggest the presence of an *S* = 1 Fe­(IV)–oxo complex. The presence of additional hydrogen bonding from the pendant alcohol group enhances the HAA reactivity compared to the Fe­(IV)–oxo intermediates with the *anti* and *syn* isomers of TMC (see above). However, OAT reactivity is significantly lower compared to the *syn* complex with TMC.^385^

In 2025, Ching and co-workers reported the formation of an Fe­(IV)–oxo complex by the heterolysis of the O–O bond of the corresponding hydroperoxo complex using a TMC ligand framework in the presence of a pendant imidazole ligand.^380^ The new iron–oxo complex was characterized by single-crystal X-ray diffraction (Figure 35). A comparative study between the pyridine- and imidazole-coordinated intermediates provided detailed mechanistic insight into the O–O bond heterolysis reaction for the formation of the Fe­(IV)–oxo intermediate. Switching the trans-ligand from pyridine to *N*-methylimidazole halves the rate of formation of the Fe­(IV)–oxo complex from the Fe­(II)–OOH precursor, indicating that the stronger electron-donating *N*-methylimidazole increases iron’s electron density, lowers its Lewis acidity, and weakens its affinity for the OOH^–^ anion. The stronger the push from the trans ligand, the greater pull required from the added base for deprotonating H_2_O_2_ to form the Fe­(II)−OOH complex. A sufficiently basic coligand then facilitates proton transfer to the distal oxygen of hydroperoxide, leading to heterolytic O–O bond cleavage and formation of the Fe­(IV)–oxo intermediate.^380^

Recently, Que, Swart and co-workers claimed the generation of a highly reactive *S* = 1 Fe­(IV)–oxo complex with a TMC ligand derivative, where all the methyl groups of TMC are replaced with benzyl groups (Scheme 8).^396^ The authors further studied the reactivity differences between corresponding *syn* and *anti* isomers, since both isomers can be generated depending on the choice of the oxidant. Finally, they explored the role of the axial chloride ligand for the reactivity of the complexes. The new intermediate, [Fe^IV^(TB^F8^C)­(O_syn_)­(Cl)]^+^, shows two UV–vis absorption bands at 890 and 1070 nm, which are characteristic features of Fe­(IV)–oxo complexes. This intermediate displays an isomer shift of 0.23 mm/s and a quadrupole splitting of 0.97 mm/s, along with an Fe–O stretching frequency of 837 cm^–1^ (see Table 3). After the initial discovery of [Fe^IV^(TMC)­(O_anti_)­(MeCN)]^2+^ in 2003, this new intermediate is the first TMC derivative capable of reacting with substrates with strong C–H bonds, like cyclohexane. In addition, [Fe^IV^(TB^F8^C)­(O_syn_)­(Cl)]^+^ is over a million-fold more reactive compared to the parent complex for fluorene and thioanisole oxidation.^396^

**Scheme 8:**
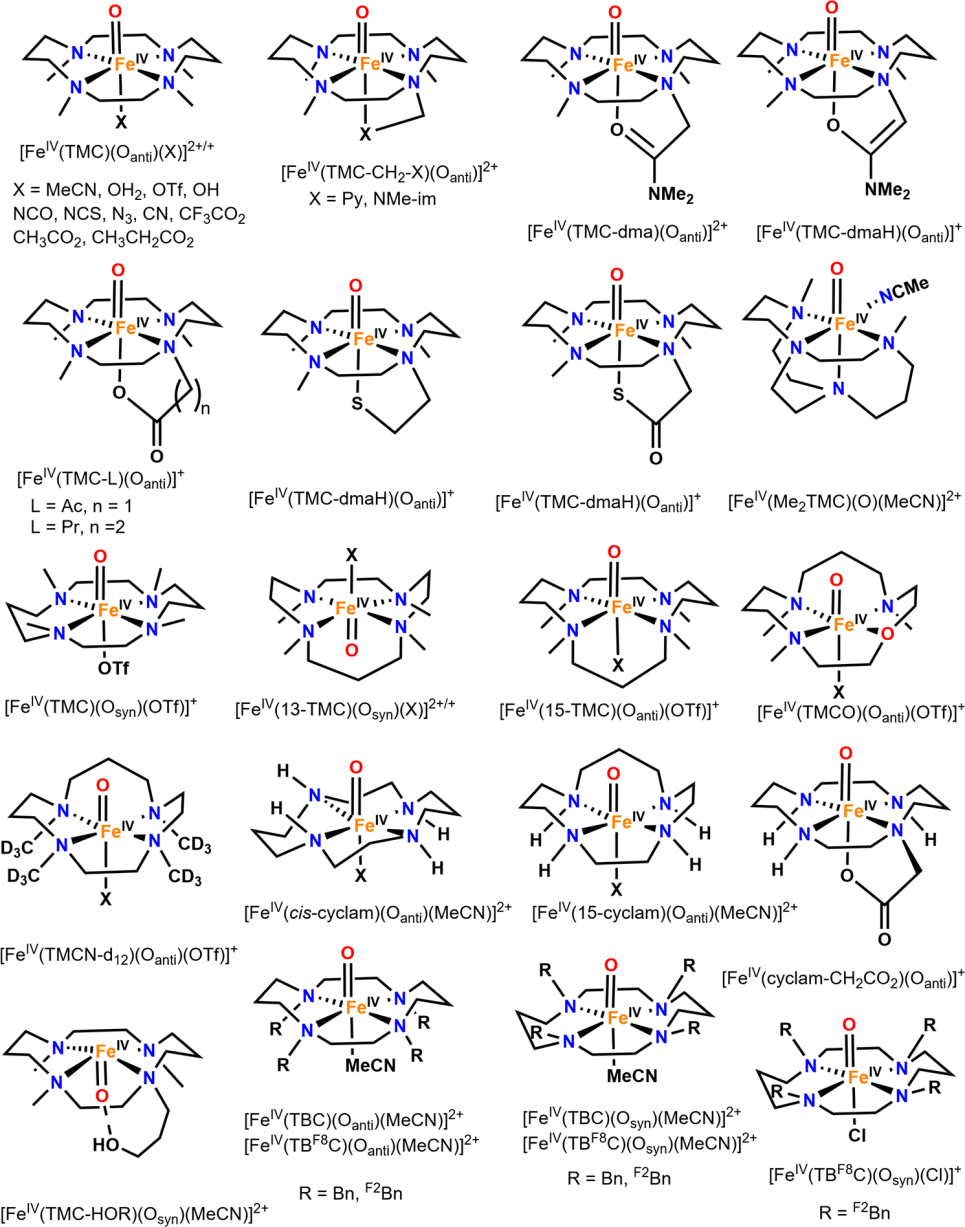
Structures of Fe­(IV)–oxo Complexes Ligated by Cyclam and TMC Ligand Derivatives

#### 3.1.2 Synthetic *S* = 1 Fe­(IV)−Oxo Complexes with Polypodal Ligands

Beyond macrocycles, polypodal ligands have been crucial in identifying the factors that influence the nature of the Fe­(IV)–oxo moiety and mediate its reactivity. The first synthetic Fe­(IV)–oxo intermediate in a polypodal ligand framework was reported using the TPA ligand framework.^431^ While [Fe^IV^(TPA)­(O)­(MeCN)]^2+^ is stable only for a few minutes, addition of one more pyridine to one of the methylene arms of TPA (= ligand N4Py) generates an Fe­(IV)–oxo complex, [Fe^IV^(N4Py)­(O)­(MeCN)]^2+^, with a half-life of 60h at room temperature. The Que group first reported that a room-temperature stable Fe­(IV)–oxo intermediate can oxidize the strong C–H bond of cyclohexane using both the N4Py and Bn-tpen ligand frameworks (Scheme 9). Also, the complex with N4Py was structurally characterized (see Figure 35).^206^ Following this initial report, various groups have reported numerous complexes involving pentadentate and tetradentate ligands (Scheme 9). One of the important discoveries in the TPA ligand framework by Nam and co-workers was the Me_3_NTB derivative, where the three pyridines of the TPA ligand are replaced with benzimidazole (B).^448^ The Nordlander group introduced two benzimidazole units in the N4Py ligand and studied the HAA reactivity of the resulting complex, [Fe^IV^(N2Py2B)­(O)]^2+^.^409^ A few years later, Que and co-workers reported the crystal structures and reactivity of [Fe^IV^(N2Py2B)­(O)]^2+^ and [Fe^IV^(N2Py2Q)­(O)]^2+^ (Q = quinoline).^330^

**Scheme 9:**
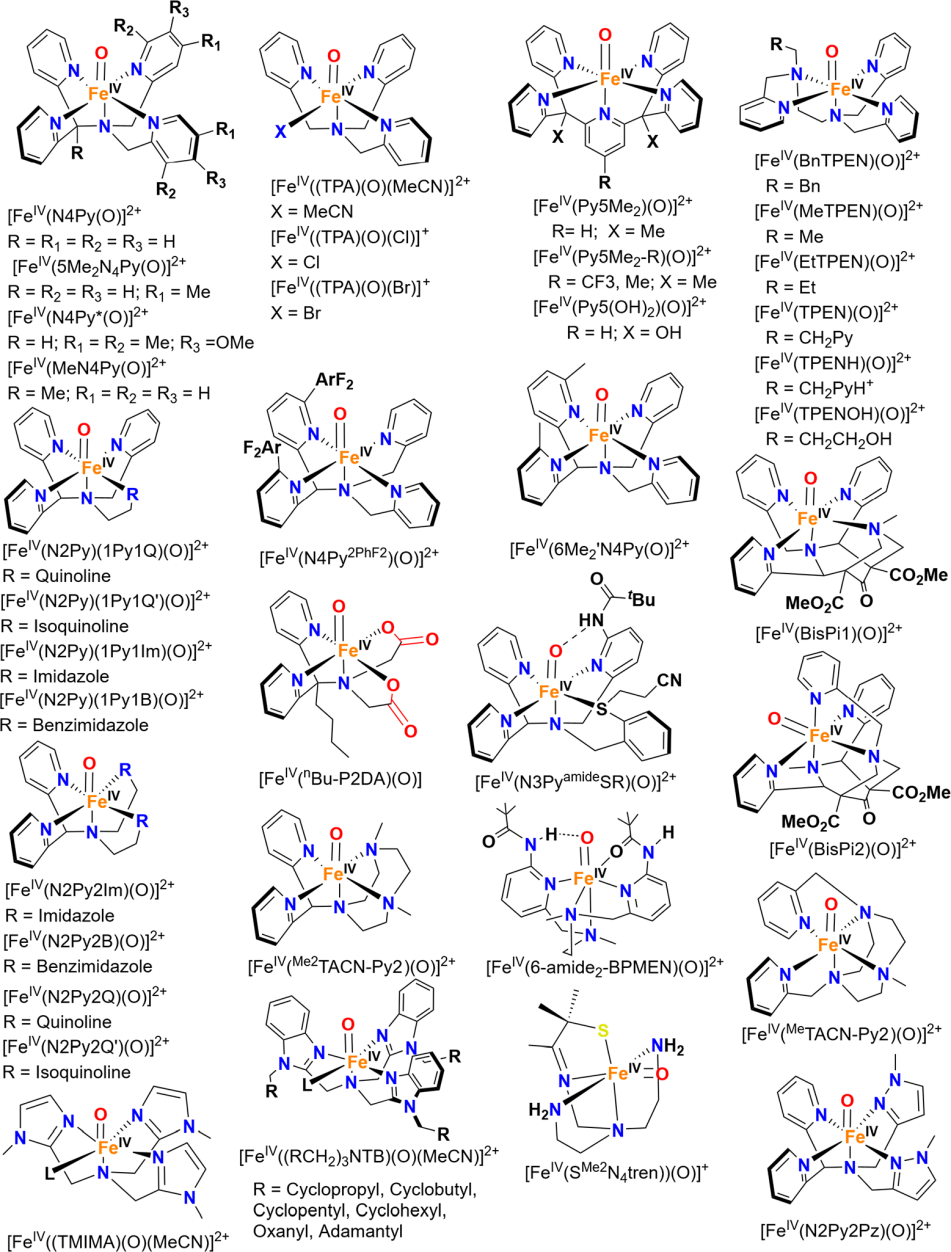
Structures of *S* = 1 Fe­(IV)–oxo Complexes with Polypodalamine-Based Ligands

In 2022, Wendt, Paine, Nordlander and co-workers reported an Fe­(IV)–oxo complex with a new pentadentate ligand from the N4Py family, N2Py­(1Py1Q), with three pyridines and one quinoline unit.^411^ This complex displays an isomer shift of −0.05 mm/s and a quadrupole splitting of 0.66 mm/s, which is similar to other *S* = 1 Fe­(IV)–oxo complexes in this family. The authors concluded that the earlier hypothesis by Que and co-workers,^330^ who proposed that sterically bulkier quinoline donors tilt the Fe=O unit away from a linear N–Fe=O arrangement by 10° in nonheme Fe­(IV)–oxo complexes, which in turn would govern their HAA reactivity, does not hold when a broader range of such intermediates is considered. In earlier comparisons, not all Fe­(IV)–oxo species were included. These newer results show that tilt angle does not reliably predict HAA reactivity of Fe­(IV)–oxo intermediates.

The Nordlander group recently reported a series of iron complexes, based on modified N4Py ligand frameworks, where one and two pyridine donors are replaced with imidazole and isoquinoline groups.^412^ Incorporation of isoquinoline enhances the stability of the resulting Fe­(IV)–oxo complexes relative to the parent N4Py system. In contrast, introducing imidazole donors led to significantly less stable Fe­(IV)–oxo complexes in the series. These new complexes exhibit isomer shifts around 0.03 mm/s, similar to that reported for the parent *S* = 1 complex with the N4Py ligand (see Table 4). The Fe­(IV)–oxo complex with the N2Py2Im ligand framework exhibits the highest reactivity in C–H bond oxidation of cyclohexane compared to the other three new complexes discussed in this work (see Table 4). The authors relate the difference in reactivity between these complexes mainly to the differences in the electronic (donor) properties of the various N-donor groups incorporated in these ligands, which suggests that (a) benzimidazole, quinoline and imidazole are weaker field ligands compared to pyridine, and (b) that weaker field coligands give more reactive Fe­(IV)–oxo complexes.

**Table 4:** Spectroscopic Properties of Synthetic *S* = 1 Polypodalamine-Based Fe­(IV)–Oxo Complexes (See Scheme 9 for Structural Drawings)

complex^a^	λ_max_ (nm) [ε (M^–1^ cm^–1^)]	r(Fe=O) (Å)	ν(Fe–O) (cm^–1^)	δ (mm/s)	Δ*E*_Q_ (mm/s)	half-life (298 K)	ref
[Fe^IV^(N4Py)(O)]^2+^	695 (400)	1.639	841	–0.04	0.93	60 h	^206,207,401^
[Fe^IV^(5Me_2_N4Py)(O)]^2+^	695	1.654				60 h	^402^
[Fe^IV^(N4Py*)(O)]^2+^	692 (430)					50 h (303 K)	^331^
[Fe^IV^(MeN4Py)(O)]^2+^	670		843				^403^
[Fe^IV^(6Me_2_N4Py)(O)]^2+^	775 (200)					30 min	^404^
[Fe^IV^(6Me’_2_N4Py)(O)]^2+^	750 (340)			0.05	0.62	2 h	^404,405^
[Fe^IV^(N4Py^2PhF2^)(O)]^2+^	750 (250)	1.666		0.03	0.54		^406,407^
[Fe^IV^(asN4Py)(O)]^2+^	705 (400)			–0.02	0.83	10 days	^408^
[Fe^IV^(N3PyB)(O)]^2+^	708 (400)			–0.03	1.1	40 h	^409^
[Fe^IV^(N2Py2B)(O)]^2+^	725 (450)	1.656	842	–0.02	1.36	2.5 h	^330,409^
[Fe^IV^(N2Py2Q)(O)]^2+^	770 (380)	1.677	833	0.03	0.56	2.5 h	^330,410^
[Fe^IV^(N2Py(1Py1Q))(O)]^2+^	730 (340)			–0.05	0.66	50 h	^411^
[Fe^IV^(N2Py2Im)(O)]^2+^	721 (274)			–0.03	1.38	1.6 h	^412^
[Fe^IV^(N2Py1Py1Im)(O)]^2+^	706 (379)			–0.03	1.13	16 h	^412^
[Fe^IV^(N2Py1Py1^iso^Q)(O)]^2+^	695 (540)			–0.04	0.87	63 h	^412^
[Fe^IV^(N2Py2^iso^Q)(O)]^2+^	696(540)	1.66		–0.04	0.85	45 h	^412^
[Fe^IV^(N2Py2Pz)(O)]^2+^	750 (250)	1.63		0.04	0.93	2 min	^413^
[Fe^IV^(N3Py^amide^SR)(O)]^2+^	750 (400)			0.04	0.80	meta stable (233 K)	^414^
[Fe^IV^(Py5Me_2_)(O)]^2+^	712 (310)	1.656	821	0.08	0.80	2 h	^402,415^
[Fe^IV^(Py5Me_2_-R)(O)]^2+^							
R = CF_3_ (in H_2_O)	710 (300)		841	0.08	0.80	2 h	^415^
R = Me (in H_2_O)	713 (300)		822	0.08	0.72	2 h	^415^
[Fe^IV^(Py5(OH)_2_)(O)]^2+^	710 (∼500)					1.5 h (208 K)	^416^
[Fe^IV^(BnTPEN)(O)]^2+^	740 (400)	1.67	835	0.01	0.87	6 h	^204,401,417^
	900 (sh)						
[Fe^IV^(NHBnTPEN)(O)]^2+^	740 (190)			0.02	0.83	∼10 min	^418^
	900 (sh)						
[Fe^IV^(MeTPEN)(O)]^2+^	756		832			1.5 h	^419,420^
[Fe^IV^(MeTPPN)(O)]^2+^	742 (300)			0.02	1.20	5 min (223 K)	^419^
[Fe^IV^(TPEN)(O)]^2+^	730 (380)		832	0.01	0.87	30 min	^420,421^
[Fe^IV^(EtTPEN)(O)]^2+^	718		833			1.5 h	^420^
[Fe^IV^(Pro3Py)(O)]^2+^	740 (250)					1 h (273 K)	^422^
[Fe^IV^(TPENaH)(O)]^+^	730 (∼300)		832	0.00	0.90	2 h	^420,423,424^
[Fe^IV^(TPENa)(O)]^+^	730		831			1.5 h	^420,423^
[Fe^IV^(TPENH)(O)]^3+^	712		833			26 h	^420^
[Fe^IV^(TPENOH)(O)]^2+^ (pH 2)	723		832			80 s	^420^
[Fe^IV^(BisPi1)(O)]^2+^	730 (400)	1.64	840	0.02	0.69		^401,425,426^
	916 (sh)						
[Fe^IV^(BisPi2)(O)]^2+^	730 (380)	1.62	825				^401,425^
	896 (sh)						
[Fe^IV^(^Me2^TACN-Py_2_)(O)]^2+^	740 (340)	1.63	839				^401^
	900 (200)						
[Fe^IV^(^Me^TACN-Py_2_)(O)]^2+^	736 (310)	1.63		–0.01	0.93		^427^
Fe^IV^(^Me^TACNS-Py_2_)(O)]^2+^	737 (310)					30 min	^428^
[Fe^IV^(^n^Bu-P2DA)(O)]	770 (220)	1.66		0.04	1.13	20 min (213 K)	^429^
[Fe^IV^(dpaq)(O)]^+^	686 (710)		805			6 h	^430^
	912 (110)						
[Fe^IV^(TPA)(O)(MeCN)]^2+^	724 (300)	1.67	830	0.01	0.92	1 h (283 K)	^431–433^
[Fe^IV^(TPA)(O)(CF_3_CO_2_)]^+^	745 (300)	1.66		0.02	0.92	20 min (283 K)	^432^
[Fe^IV^(TPA)(O)(Cl)]^+^	778 (350)	1.65		0.04	0.95	2 min (283 K)	^432^
[Fe^IV^(TPA)(O)(Br)]^+^	800 (400)	1.66		0.06	0.95	2 min (283 K)	^432^
[Fe^IV^(TPA*)(O)(MeCN)]^2+^	738 (300)			0.01	0.95		^434^
[Fe^IV^(6MeTPA)(O)(MeCN)]^2+^	770 (300)					16 min (283 K)	^435^
[Fe^IV^(QBPA)(O)(MeCN)]^2+^	775 (300)			0.05	0.70	20 min (283 K)	^328,435^
[Fe^IV^(BPMEN)(O)(MeCN)]^2+^	740 (−)						^436^
[Fe^IV^(β-BPMCN)(O)(MeCN)]^2+^	753 (280)	1.66					^437^
[Fe^IV^(α-BQCN)(O)(MeCN)]^2+^	758 (∼120)			0.07	1.02		^438^
	895 (sh)						
[Fe^IV^(β-BQCN)(O)(MeCN)]^2+^	770 (∼180)					1.5 h (273 K)	^438^
	910 (sh)						
[Fe^IV^(BQEN)(O)(MeCN)]^2+^	740 (−)	1.67				30 min (273 K)	^439^
[Fe^IV^(PDP*)(O)(MeCN)]^2+^	730 (350)					2 min	^440^
[Fe^IV^(BisPi3)(O)(MeCN)]^2+^	768 (130)			0.07	0.66	30 min (233 K)	^441,442^
[Fe^IV^(BisPi3)(O)(Cl)]^+^	595					2 min (183 K)	^441,442^
	850						
[Fe^IV^(Pytacn)(O)(X)]^2+^	750 (200)			0.05	0.73	2.4 h (288 K)	^443^
X = MeCN/H_2_O							
[Fe^IV^(Pytacn)(O)(Cl)]^+^	803			0.06	0.89	3.2 h (243 K)	^444^
[Fe^IV^(Pytacn)(O)(Br)]^+^	823			0.07	0.89	2.8 h (243 K)	^444^
[Fe^IV^(^Cl,H^Pytacn)(O)(H_2_O)]^2+^	778 (270)					>90 min	^445^
[Fe^IV^(^CO2Et,H^Pytacn)(O)(H_2_O)]^2+^	770 (300)					>90 min	^445^
[Fe^IV^(^NO2,H^Pytacn)(O)(H_2_O)]^2+^	754 (240)					>90 min	^445^
[Fe^IV^(6-amide_2_-BPMEN)(O)]^2+^	750			0.06	1.19	25 min (273 K)	^446^
[Fe^IV^{(6-amide_2_-BPMEN)-H)}(O)]^2+^	740			–0.05	1.24	5 min	^446^
[Fe^IV^(Bn^CR^TPEN)(O)]^2+^	739						^447^
[Fe^IV^(Me_3_NTB)(O)(MeCN)]^2+^	770 (200)	1.65		0.02	1.62	4 min (233 K)	^448,449^
[Fe^IV^((RCH_2_)_3_NTB)(O)(MeCN)]^2+^	772 (418)			0.04	1.60	2 min (233 K)	^450^
R = cycloproyl							
[Fe^IV^((RCH_2_)_3_NTB)(O)(MeCN)]^2+^	771 (387)			0.04	1.60	43 s (233 K)	^450^
R = cyclobutyl							
[Fe^IV^((RCH_2_)_3_NTB)(O)(MeCN)]^2+^	773 (342)			0.04	1.60	33 s (233 K)	^450^
R = cyclopentyl							
[Fe^IV^((RCH_2_)_3_NTB)(O)(MeCN)]^2+^	765 (310)			0.08	1.53	30 s (233 K)	^450^
R = cyclohexyl							
[Fe^IV^((RCH_2_)_3_NTB)(O)(MeCN)]^2+^	773 (389)			0.05	1.55	36 s (233 K)	^450^
R = oxanyl							
[Fe^IV^((RCH_2_)_3_NTB)(O)(MeCN)]^2+^	767 (387)			0.04	1.60	37 s (233 K)	^450^
R = adamantyl							
[Fe^IV^(^R^PY_4_Cl2BIm)(O)]^2+^	∼800 (>100)						^368^
R = Py							
[Fe^IV^(^R^PY_4_Cl2BIm)(O)]^2+^	∼800 (>100)						^368^
R = mesityl (Mes)							
[Fe^IV^(^R^PY_4_Cl2BIm)(O)]^2+^	∼800 (>100)						^368^
R = anthracenyl (Ant)							
[Fe^IV^(TMIMA)(O)(MeCN)]^2+^	750 (250)			0.07	1.77	4 min (193 K)	^451^

a Ligand nomenclature: TPA = tris­(2-pyridylmethyl)­amine; TMIMA = tris­(2-imidazolemethyl)­amine; Me_3_NTB = tris­(1-methylbenzimidazolyl)­amine); bispidine = 2,4-di­(pyridine-2-yl)-3,7-diazabicyclo[3.3.1]­nonane-1-one (bispidine); MeTACNS-Py_2_ = 1-thia-4,7-bis­(2-pyridylmethyl)-diazacyclononane; PyTACN = 1-(pyridyl-2-methyl)-4,7-dimethyl-1,4,7-triazacyclononane; Tpena = *N,N,N*′-tris­(2-pyridylmethyl)­ethylenediamine-*N*′-acetate; BnTPEN = *N*-benzyl-*N*,*N*′,*N*′-tris­(2-pyridylmethyl)-1,2-diaminoethane; 5Me_2_N4Py = *N*,*N*-bis­((5-methyl-2-pyridinyl)­methyl)-1,1-di­(2-pyridinyl)­methanamine; N­(2Py)­(1Py)­(1Im) = [*N*-(1-methyl-2-imidazolyl)­methyl-*N*-(2-pyridyl)-methyl-*N*-(bis-2-pyridylmethyl)-amine; N2Py2Im = *N*-bis­(1-methyl-2-imidazolyl)­methyl-*N*-(bis-2-pyridylmethyl)­amine; N­(2Py)­(1Py)­(1Q′) = *N*-(isoquinolin-3-ylmethyl)-1,1-di­(pyridin-2-yl)-*N*-(pyridin-2-ylmethyl)­methanamine; N2Py2Q’ = *N*,*N*-bis­(isoquinolin-3-ylmethyl)-1,1-di­(pyridin-2-yl)­methanamine; N­(2Py)­(1Py)­(1B) = *N*-(1-methyl-2-benzimidazolyl)­methyl-*N*-(2-pyridyl)­methyl-*N*-(bis-2-pyridyl methyl)­amine; N2Py2B = *N*-bis­(1-methyl-2-benzimidazolyl)­methyl-*N*-(bis-2-pyridylmethyl)­amine; N2Py2Pz = *N*-bis­(1-methyl-2-pyrazolyl)­methyl-*N*-(bis-2-pyridylmethyl)­amine; 6Me_2_N4Py = bis­(6-methylpyridin-2-yl)-*N*,*N*-bis­((pyridin-2-yl)­methyl)­methanamine; Bn^CR^TPEN = *N*-benzyl-*N*-(5-(4′-benzo-18-crown-6)-2-pyridylmethyl)-*N*′,*N*′-bis­(2-pyridylmethyl)-1,2-diaminoethane; PY5Me_2_-X = 2,6-bis­(1,1-bis­(2-pyridyl)­ethyl)­pyridine; X = CF_3_, H, Me, or NMe_2_; MeTPEN = *N*-methyl-*N*,*N*′,*N*′-tris­(2-pyridylmethyl)­ethane-1,2-diamine; MeTPPN = *N*-methyl-*N*,*N*’,*N*’-tris­(2-pyridylmethyl)­propane-1,3-diamine; MePy2tacn = *N*-methyl-*N*,*N*-bis­(2-picolyl)-1,4,7-triazacyclononane; dpaq^–^ = 2-[bis­(pyridin-2- ylmethyl)]­amino-*N*-quinolin-8-yl-acetamido; bpmen = *N*,*N*′-dimethyl-*N*,*N*′-bis­(2-pyridylmethyl)­ethane-1,2-diamine; BQCN = *N*,*N*′-dimethyl-*N*,*N*′-bis­(8-quinolyl)­cyclohexanediamine; bqen = *N*,*N*′-dimethyl-*N*,*N*′-bis­(8-quinolyl)­ethane-1,2-diamine; bpmen = *N*,*N*′-dimethyl-*N*,*N*′-bis­(2-pyridylmethyl)­ethane-1,2-diamine; PDP* = bis­(3,5-dimethyl-4-methoxypyridyl-2-methyl)-(*R*,*R*)-2,2′-bipyrrolidine; ^Me,H^Pytacn = 1-(2′-pyridylmethyl)-4,7-dimethyl-1,4,7-triazacyclononane; BPQA = bis­(2-pyridylmethyl)­(2-quinolylmethyl)­amine

In a follow-up study, Que and co-workers investigated a new Fe­(IV)–oxo complex, featuring a pyrazole-based ligand derivative of the N4Py ligand (N2Py2Pz), designed to modulate the electrophilicity of the complex by variation of the ligand field.^413^ The new Fe­(IV)–oxo complex displays a UV–vis absorption band around 750 nm, and an isomer shift (δ = 0.04 mm/s) and quadrupole splitting (Δ*E*_Q_ = 0.93 mm/s) similar to other reported *S* = 1 Fe­(IV)–oxo complexes. Since pyrazole is less basic compared to other heterocycles studied so far in the N4Py ligand family, the authors observed a dramatic enhancement in HAA reactivity, with the pyrazole-ligated complex being approximately 5000 times more reactive than the parent N4Py complex and 40,000 times more reactive than the isoquinoline analogue for cyclohexane oxidation at 298 K in acetonitrile (see Figure 36). The study emphasizes the dual importance of substrate accessibility and electrophilicity in controlling reactivity. Among the series, the most reactive complex also exhibits the highest electron-transfer rate. Importantly, the reactivity of all complexes strongly correlates with their Fe­(IV)/Fe­(III) reduction potentials (*E*_1/2_). DFT-calculated energy differences between the Fe­(IV) and Fe­(III) complexes lie in the range from −92 to −100 kcal/mol along this series (see Figure 37). These results reinforce the idea that tuning electronic properties through ligand design is a key strategy for enhancing the oxidative power of high-valent Fe­(IV)–oxo intermediates.

**Figure 36:**
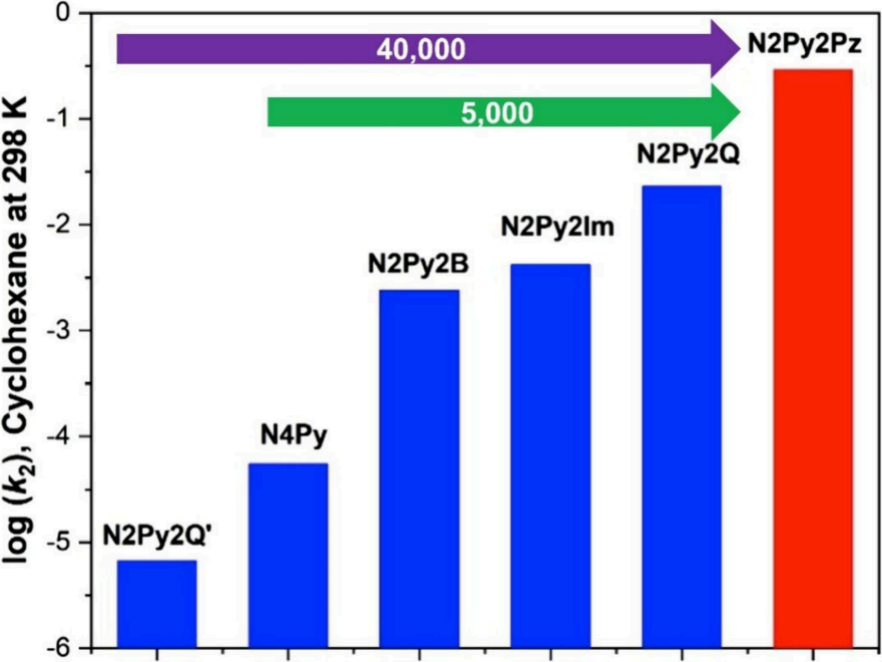
Bar graph comparing log­(*k*_2_) values (with *k*_2_ being second-order rate constants for cyclohexane oxidation) at 298 K by various [Fe^IV^(L)­(O)]^2+^ complexes, with ligands L being derivatives of the N4Py ligand, using different *N*-heterocycles in the ligand framework (see Table 4 for ligand definitions). Reproduced with permission from ref 413 under Creative Commons License CC BY-NC-ND. Copyright 2025 The Authors, National Academy of Sciences.

**Figure 37:**
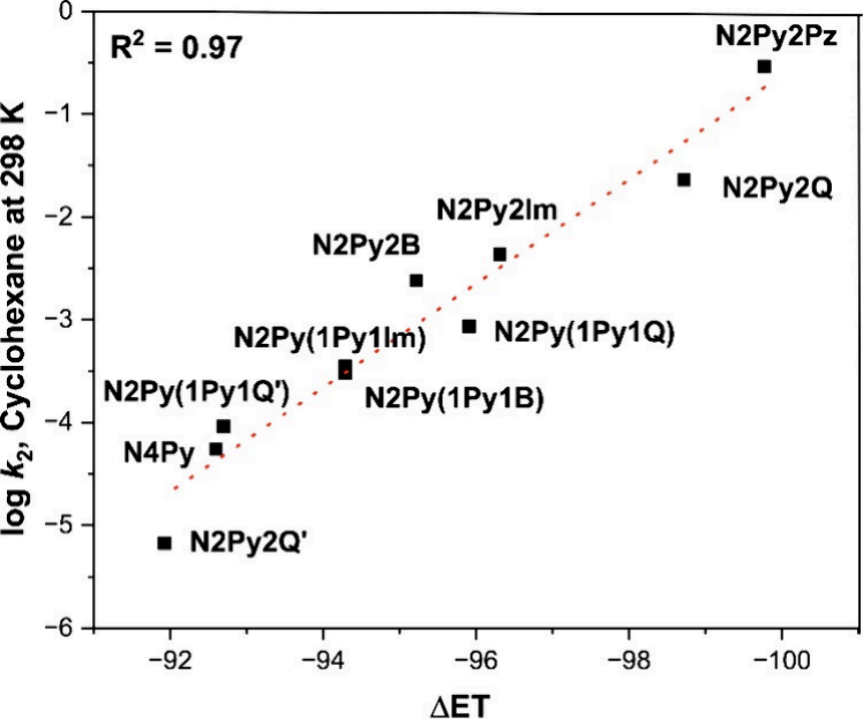
Plot of log­(*k*_2_) (with *k*_2_ being second-order rate constants) for cyclohexane oxidation vs DFT-calculated energy differences between the Fe­(IV) and Fe­(III) states of the indicated complexes, ΔET (B97-D2/TZ2P, COSMO, ZORA; kcal mol^–1^). Reproduced with permission from ref 413 under Creative Commons License CC BY-NC-ND. Copyright 2025 The Authors, National Academy of Sciences.

In 2023, Dantignana et al. reported the reactivity of two Fe­(IV)–oxo isomers that differ in the relative position of the oxo group with respect to the pyridine unit in the [Fe­(^R^PyNMe_3_)­(O)]^2+^ complex (see Scheme 9). These intermediates were generated from their iron­(II) precursors using NBu_4_IO_4_.^169^ The authors prepared a series of ligands using different substituents on pyridine based on their previously studied Fe­(IV)–oxo intermediate with ligand PyNMe_3_. The authors have previously found that this ligand framework generates two Fe­(IV)–oxo isomers, which are well characterized by rRaman and Mössbauer spectroscopy, and EXAFS.^398^ From both spectroscopic and reactivity studies, it was found that when the pyridine unit is trans to the oxo ligand, the isomer is thermodynamically more stable. In this recent work on a series of electronically tuned Fe­(IV)–oxo intermediates, kinetic and computational studies uncovered a previously unreported pathway for interconversion between geometric Fe­(IV)–oxo isomers. These results provide further insight into the “ferryl flip” postulated in several nonheme iron-dependent oxygenases.^167,168^ Interestingly, variations in the electronic nature of the R substituent on the pyridine ring have little effect on reaction rates. On the other hand, the position of the oxo ligand in the coordination sphere of the Fe­(IV) center in the isomers governs their relative HAT reactivity, essentially overriding changes in ligand donor properties.

In 2024, Paine and co-workers reported new Fe­(IV)–oxo complexes in the BPMEN ligand framework, with the intent of elucidating the role of SCS amide NH groups in governing the HAA reactivity of these complexes (see Scheme 9).^446^ The 6-amide_2_-BPMEN ligand stabilizes an *S* = 1 Fe­(IV)–oxo intermediate, [Fe^IV^(6-amide_2_-BPMEN)­(O)]^2+^, with a half-life of 25 min at 273 K, and which shows an absorption band around 750 nm similar to other reported Fe­(IV)–oxo complexes (see Table 4). The addition of one equiv of 2,6-lutidine to this species led to the generation of a new complex, which has an absorption band at 740 nm due to the deprotonation of the coordinated amide NH proton. This transformation is reflected by a change in isomer shift from −0.05 mm/s to 0.06 mm/s. The authors claim that this new oxidant shows the highest alcohol/ketone selectivity in cyclohexane oxidation among Fe­(IV)–oxo complexes of this kind, as well as 3°/2° selectivity in adamantane oxidation, which had not been previously reported for any synthetic nonheme Fe­(IV)–oxo complex.^446^ A theoretical analysis further demonstrates that a hydrogen bond between the −NH moiety of the noncoordinating amide and the Fe=O core significantly modulates the electronic structure of the complex: by redistributing electron density within the equatorial plane, this interaction reduces the energy gap between the triplet and quintet spin states, facilitating rapid access to the reactive quintet surface (see TSR model above). As a result, this intermediate exhibits enhanced reactivity by lowering the energetic barrier for HAA.

In 2024, Kumar, Sastri, deVisser and co-workers reported a new Fe­(IV)–oxo complex that explores the influence of sulfur coordination on the properties of these reactive intermediates. For this purpose, they modified the previously studied ligand ^Me^TACN–Py_2_, replacing one equatorial nitrogen donor with sulfur.^428^ The resulting Fe­(IV)–oxo intermediate exhibits an absorption band at 737 nm and has a half-life of 30 min at 298 K, comparable to that of the parent complex, [Fe^IV^(^Me^TACN-Py2)­(O)]^2+^, reported by Company et al. (see Table 4).^427^ Both experimental and DFT studies revealed that the sulfur donor in the equatorial plane shifts the reduction potential of the intermediate more positively (480 mV), enhances electron transfer processes, and facilitates an unusual hydride transfer for the HAA reaction. A lower KIE value of 4.7 for xanthene oxidation compared to the value of 23.7 for [Fe^IV^(^Me^TACN-Py2)­(O)]^2+^ rules out a PT/ET-type mechanism for the oxidation of this substrate. The authors conclude that a different mechanism is operating for the HAA reaction with their sulfur-containing compound, favoring hydride transfer over traditional HAA reactivity.^428^

Costas, Stefeno, Olivo and co-workers demonstrated how substrate recognition in the SCS enhances C–H bond oxidation in a quantitative way by a biomimetic nonheme Fe­(IV)–oxo model complex.^447^ Both [Fe^IV^(BnTPEN)­(O)]^2+^ and its crown ether variant [Fe^IV^(Bn^CR^TPEN)­(O)]^2+^ share the same primary coordination sphere, but only the latter selectively binds the substrate (protonated long-chain primary amines) via a crown ether receptor. The protonated amine binds to the crown ether due to electrostatic interactions, placing the tail of the long hydrocarbon chain in proximity to the iron–oxo center (Figure 38). This supramolecular recognition turns the following C–H activation reaction into an intramolecular process, giving rise to Michaelis–Menten kinetics, higher turnover rates, and remarkable site selectivity for distal C–H bonds. Additionally, substrate binding improves the efficiency of hydroxyl rebound by positioning the reactive substrate radical closer to the formed Fe­(III)–OH center. Overall, these findings underscore the power of substrate positioning via molecular recognition to modulate and enhance the reactivity of nonheme Fe­(IV)–oxo catalysts.^447^

**Figure 38:**
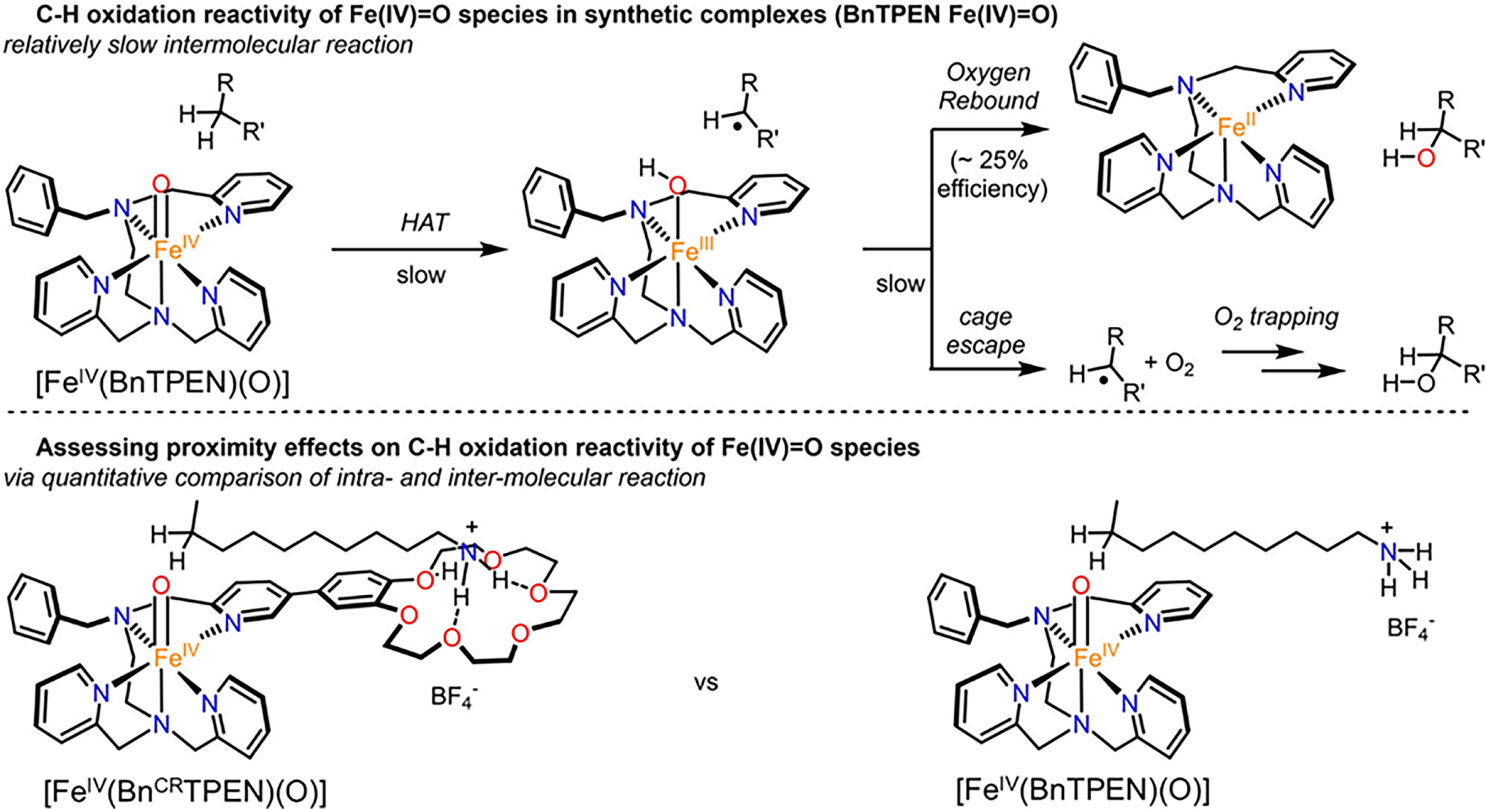
Top: C–H oxidation by the synthetic nonheme Fe­(IV)–oxo model complex [Fe^IV^(BnTPEN)­(O)]^2+^. Bottom: Targeted analysis of proximity effects on the reactivity of a related nonheme Fe­(IV)–oxo species. Reproduced with permission from ref 447 under Creative Commons License CC BY. Copyright 2024 The Authors, Wiley.

Kovacs and co-workers recently reported the geometric and electronic structures of O_2_-derived, aliphatic thiolate-ligated Fe complexes, including Fe–peroxo, Fe–hydroxo, and high-valent Fe­(IV)–oxo species.^452^ The authors have previously shown that their thiolate-ligated complex [Fe^III^(S^Me2^N_4_(tren))­(THF)]^2+^ reacts with superoxide to form a transient ferric superoxo intermediate, [Fe^III^(S^Me2^N_4_(tren))­(O_2_)]^+^, which can only be trapped below −125 °C.^453^ This intermediate can also be generated using O_2_ directly at −130 °C in MeOH/EtOH (1:1) solvent mixture. By warming the solution temperature, the authors found a new intermediate that forms within 75 s at −73 °C in their stopped-flow experiments. This intermediate is EPR silent in both perpendicular and parallel mode, indicating it is a dimeric species, [{(S^Me2^N_4_(tren))­Fe^III^}_2_(μ-O_2_^2–^)]^2+^. While this peroxo species is stable for weeks at −80 °C, it transforms into a new intermediate upon warming to −40 °C, identified as the complex [Fe^III^(S^Me2^N_4_(tren))­(OH)]^+^. The oxidation state assignment of this complex was confirmed by EPR spectroscopy, showing a low-spin (*S* = 1/2) ferric complex. This Fe­(III)–peroxo to Fe­(III)–OH transformation has a KIE effect of 4 when the reaction is carried out in CD_3_OD, indicating the presence of a short-lived Fe­(IV)–oxo intermediate, which can cleave the strong C–H bond of MeOH (BDFE = 96 kcal/mol). Further crystallization of the oxygenated species allowed for the structural characterization of a *cis*-thiolate-coordinated Fe­(IV)–oxo intermediate (the thiolate is *cis* to the Fe=O unit), [Fe^IV^(S^Me2^N_4_tren))­(O)]^+^. Electronic structure calculations support an *S* = 1 ground state for this Fe­(IV)–oxo complex, which exhibits an unusually high Fe–O stretching frequency of 918 cm^–1^. This is consistent with its exceptionally short Fe–O bond length (∼1.603 Å).^452^ Upon warming, OAT from the Fe=O unit to the thiolate ligand is observed, leading to formation of a sulfenic acid group that is coordinated to the iron center.

Que and co-workers investigated a series of N-substituted Me_3_NTB ligands, systematically varying cycloalkylmethyl groups at the 1-position of the benzimidazole (see Scheme 9).^450^ The authors synthesized *S* = 1 Fe­(IV)–oxo complexes with these ligands that exhibit the highest C–H bond oxidation reactivity reported for nonheme iron complexes so far that are supported by tripodal ligands. This series revealed that although the complexes have comparable spectroscopic (isomer shift: 0.03–0.08 mm/s) and electronic properties (absorption band: 765–773 nm) across the series (see Table 4), their rates for substrate oxidation differ by up to an order of magnitude due to the SCS effects of the cycloalkyl substituents. The second-order rate constant (*k*_2_) of [Fe^IV^((RCH_2_)_3_NTB)­(O)­(MeCN)]^2+^ (where R = cyclohexyl) for cyclohexane oxidation is 1.75 M^–1^s^–1^ at −40 °C, while the previously reported^449^ complex [Fe^IV^(Me_3_NTB)­(O)­(MeCN)]^2+^ has a rate constant for the same reaction of only 0.24 M^–1^s^–1^. Low-temperature NMR studies revealed that all of these intermediates have an *S* = 1 spin state at −40 °C, by comparing chemical shifts of benzimidazole protons with data from previous NMR studies on [Fe^IV^(Me_3_NTB)­(O)­(MeCN)]^2+^. Interestingly, these reactivity trends correlate with the ring strain enthalpies of the cycloalkyl substituents, underscoring the critical role of SCS sterics in tuning high-valent iron–oxo reactivity.

Another report of a highly reactive Fe­(IV)–oxo intermediate by Que, Swart and co-workers used the TMIMA ligand (see Scheme 9).^451^ The authors show that this intermediate is highly unstable, with a *t*_1/2_ of only 4 min at −80 °C in acetone/MeCN (8:2) solvent, compared to other complexes in the TPA ligand family that are much more stable under these conditions (see Table 4). The Fe­(IV)–oxo intermediate exhibits a weak UV–vis absorption band at 750 nm (εM = 250 M^–1^ cm^–1^). Mössbauer spectroscopy revealed that this species has an *S* = 1 spin state, with an isomer shift of 0.07 mm/s and a quadrupole splitting of 1.77 mm/s, which are similar to those of other reported *S* = 1 Fe­(IV)–oxo complexes (see Table 4). This compound is the most reactive Fe­(IV)–oxo intermediate in the TPA ligand family prepared to this date, showing an almost 1 order of magnitude higher second-order rate constant for cyclohexane oxidation than [Fe^IV^(TQA)­(O)­(MeCN)]^2+^ and [Fe^IV^(Me_3_NTB)­(O)­(MeCN)]^2+^ (see Table 5). The authors propose that substrate access is a dominating factor for the increased reactivity of the TMIMA over the TQA complex and not differences in electrophilicity, due to the presence of three small imidazole over the three large quinoline units, respectively. The TMIMA Fe­(IV)–oxo intermediate shows a C–H/D KIE of 50 for toluene oxidation, indicating that C–H bond cleavage is the rate-determining step in toluene oxidation by this intermediate.

**Table 5:** Reactivity and Spin State of Fe­(IV)–Oxo Complexes

complex	T (K)	half-life *t*_1/2_	S at 4K	*k*_2_ (PhSMe) (M^–1^s^–1^)	*k*_2_ (DHA) (M^–1^s^–1^)	*k*_2_(toluene) (M^–1^s^–1^)	*k*_2_ (cyclohexane) (M^–1^s^–1^)	ref
[Fe^IV^(TMC)(O_anti_)(MeCN)]^2+^	298	10 h	1	1.0 × 10^–2^	2.3 × 10^–1^			^205,384^
[Fe^IV^(TMC)(O_syn_)(MeCN)]^2+^	298	2 h	1	8.2				^384^
[Fe^IV^(TBC)(O_anti_)(MeCN)]^2+^	298	35 min	1	4				^395^
[Fe^IV^(TBC)(O_syn_)(MeCN)]^2+^	298	23 min	1	3.0 × 10^2^				^396^
[Fe^IV^(TB^F8^C)(O_anti_)(MeCN)]^2+^	298	6 min	1	5.0 × 10^1^	6 (233 K)		1.0 × 10^–2^	^396^
[Fe^IV^(TB^F8^C)(O_syn_)(MeCN)]^2+^	298	2 min	1	1.2 × 10^4^	1.2 × 10^1^ (233 K)		1.5 × 10^–2^	^396^
[Fe^IV^(TB^F8^C)(O_syn_)(Cl)]^+^	253	1.5 min	1	4.9 × 10^2^	3.5 × 10^1^ (193 K)		2.0 × 10^–1^	^396^
[Fe^IV^(Me_2_EBC)(O_syn_)(MeCN)]^2+^	273	5 h	1	4.1 × 10^–1^	7.4			^386^
*cis*-[Fe^IV^(cyclam)(O)(MeCN)]^2+^	253	3 min	1		1.3			^393^
*trans*-[Fe^IV^(cyclam)(O)(MeCN)]^2+^	298	2 h	1		4.9 × 10^–2^			^392^
[Fe^IV^(TMCO)(O_anti_)(OTf)]^+^	223	1.5 h	1			5 × 10^–2^ (233 K)	2 × 10^–2^ (233 K)	^391^
[Fe^IV^(13-TMC)(O_syn_)(OTf)]^+^	233	30 min	1		5.7			^387^
[Fe^IV^(TMCN-d_12_)(O_syn_)]^+^	273	8 min	1	4.0 × 10^–3^ (233 K)				^391^
[Fe^IV^(TAML)(O)]^2–^	298	>2 h	1			1.4 × 10^–1^	2.3 × 10^–2^	^454,455^
[Fe^IV^(N4Py)(O)]^2+^	298	60 h	1	1.24	9.85	6.3 × 10^–4^	5.5 × 10^–5^	^206^
[Fe^IV^(BnTPEN)(O)]^2+^	298	6 h	1		100	8.9 × 10^–3^	3.9 × 10^–4^	^206,401^
[Fe^IV^(6Me_2_N4Py)(O)]^2+^	298	30 min				1.9 × 10^–2^	2.5 × 10^–2^	^405^
[Fe^IV^(6Me’_2_N4Py)(O)]^2+^	298	2 h		5.0 × 10^–4^	1.42	1 × 10^–3^	1 × 10^–3^	^456^
[Fe^IV^(N2Py2B)(O)]^2+^	298	2.5 h	1			1.2 × 10^–2^	2.9 × 10^–3^	^409^
[Fe^IV^(N2Py)(1Py1B)(O)]^2+^	298	40 h	1			1.3 × 10^–3^	3 × 10^–4^	^409^
[Fe^IV^(N2Py2Q)(O)]^2+^	298	2.5 h	1			1.2 × 10^–2^	2.9 × 10^–2^	^330^
Fe^IV^(N2Py)(1Py1Q)(O)]^2+^	298	50 h	1	2.3 × 10^–2^		7.4 × 10^–3^	8.6 × 10^–4^	^411^
Fe^IV^(N2Py2Im)(O)]^2+^	298	1.67 h	1			4.7 × 10^–3^	4.33 × 10^–3^	^412^
Fe^IV^(N2Py2Q’)(O)]^2+^	298	63 h	1			4.5 × 10^–4^	6.7 × 10^–6^	^412^
[Fe^IV^(N2Py)(1Py1Im)(O)]^2+^	298	16 h	1			5.78 × 10^–4^	3.53 × 10^–4^	^412^
[Fe^IV^(N2Py)(1Py1Q′)(O)]^2+^	298	43 h	1			3.3 × 10^–4^	9.14 × 10^–5^	^412^
[Fe^IV^(N2Py2Pz)(O)]^2+^	298	2 min	1			1.5 × 10^–1^	2.9 × 10^–1^	^413^
[Fe^IV^(N3Py^amide^SR)(O)]^2+^	233	meta stable	1	4.3 (233 K)				^414^
Fe^IV^(Py5(OH)_2_)(O)]^2+^	208	1.5 h	1		4.3 × 10^–4^ (233 K)			^416^
[Fe^IV^(BisPi1)(O)(Cl)]^+^	193	3.5 s	1				7.6 × 10^2^	^457,458^
[Fe^IV^((^Me2^TACN-Py_2_)(O)]^2+^	298		1	4.0 × 10^–3^ (263 K)	7.4			^401^
[Fe^IV^(BisPi1)(O)]^2+^	298			2.4 × 10^–2^ (263 K)	1.1			^401^
[Fe^IV^(BisPi2)(O)]^2+^	298			2.4 (263 K)	40			^401^
[Fe^IV^((^Me^TACN-Py_2_)(O)]^2+^	298	60 min	1	2.87 × 10^–2^				^428^
[Fe^IV^((^Me^TACNS-Py_2_)(O)]^2+^	298	30 min	1	2.40 × 10^–1^				^428^
[Fe^IV^((dpaq)(O)]^2+^			1			3.9 × 10^–3^		^430^
[Fe^IV^((PDP*)(O)]^2+^	298	2 min	1	274			9.9 × 10^–2^	^440^
[Fe^IV^((Pytacn)(O)(H_2_O)]^2+^	288	2.4 h	1		5.7 (258 K)		4.0 × 10^–4^ (298 K)	^443^
[Fe^IV^((Pytacn)(O)(Cl)]^+^	243	3.2 h	1		2.2 × 10^–3^			^444^
[Fe^IV^((Pytacn)(O)(Br)]^+^	243	2.8 h	1		1.9 × 10^–3^			
[Fe^IV^(TPA)(O)(MeCN)]^2+^	273	460 min	1			6.0 × 10^–3^	2.3 × 10^–4^	^328^
[Fe^IV^(BPQA)(O)(MeCN)]^2+^	273	40 min	1			6.56 × 10^–2^	4.2 × 10^–3^	^328^
[Fe^IV^(TMIMA)(O)(MeCN)]^2+^	193	4 min	1			8.0	1.56	^451^
[Fe^IV^(Me_3_NTB)(O)(MeCN)]^2+^	233	2 min	1			4.7 × 10^–1^	2.4 × 10^–1^	^448^
[Fe^IV^((RCH_2_)_3_NTB)(O)(MeCN)]^2+^ R = *c*-propyl	233	43 s	1			9.2 × 10^–1^	5.3 × 10^–1^	^450^
[Fe^IV^((RCH_2_)_3_NTB)(O)(MeCN)]^2+^ R = *c*-butyl	233	38 s	1			1.10	6.2 × 10^–1^	^450^

[Fe^IV^((RCH_2_)_3_NTB)(O)(MeCN)]^2+^	233	33 s	1			1.64	1.08	^450^
R = *c*-pentyl								

[Fe^IV^((RCH_2_)_3_NTB)(O)(MeCN)]^2+^	233	30 s	1			3.11	1.75	^450^
R = *c*-hexyl								

[Fe^IV^((RCH_2_)_3_NTB)(O)(MeCN)]^2+^	233	36 s	1			2.58	1.56	^450^
R = oxanyl								

[Fe^IV^((RCH_2_)_3_NTB)(O)(MeCN)]^2+^	233	37 s	1			1.96	1.22	^450^
R = adamantyl								

[Fe^IV^(TMG_3_tren)(O)(MeCN)]^2+^	243	4.3 h	2		9.0 × 10^–2^			^318^
[Fe^IV^(TMG_2_dien)(O)(MeCN)]^2+^	223	30 min	2		5.7 × 10^1^			^318^
[Fe^IV^(TQA)(O)(MeCN)]^2+^	233	15 min	2		2.0 × 10^2^ (193 K)	6.0 × 10^–1^	3.7 × 10^–1^	^366^
[Fe^IV^(TQA)(O)(Cl)]^+^	233	5 min	2			7.0 × 10^–2^		^459^
[Fe^IV^(TQA)(O)(Br)]^+^	233	5 min	2			5.0 × 10^–2^		^459^
[Fe^IV^(BQPA)(O)(MeCN)]^2+^	233	2 min	2			4.0 × 10^–1^	1.1 × 10^–2^	^328^
[Fe^IV^(^t^Bu_3_TACN)(O)]^2+^	203	20 min	2		1.6	4.3 × 10^–1^		^460^

In 2023, Kojima and co-workers reported catalytic methane oxidation using nonheme Fe­(IV)–oxo complexes in an aqueous medium.^368^ These complexes feature an N-heterocyclic carbene as the axial ligand and four pyridine arms with hydrophobic moieties (mesityl and anthracenyl) in the equatorial plane (see Figure 39). The authors found that the presence of hydrophobic moieties allows for the capture of gaseous substrates and release of the hydrophilic products into the solution (see Figure 39). This catch-and-release strategy provides higher selectivity of alcohol over acid products, the latter being generated by overoxidation. Gaseous alkanes, methane, ethane, propane, and *n*-butane, were used as substrates for C–H hydroxylation in this study. Kinetic studies of CH_4_ oxidation were conducted in H_2_O:CH_3_CN (95:5, v/v) at 323 K in the presence of a large excess of Na_2_S_2_O_8_, yielding a rate constant of (2.8 ± 0.1) × 10^–6^ s^−1^ for the anthracenyl-substituted complex. KIE studies using CD_4_ under the same reaction conditions revealed a significant KIE of 37, suggesting C–H bond cleavage as the rate-determining step of the reaction.

**Figure 39:**
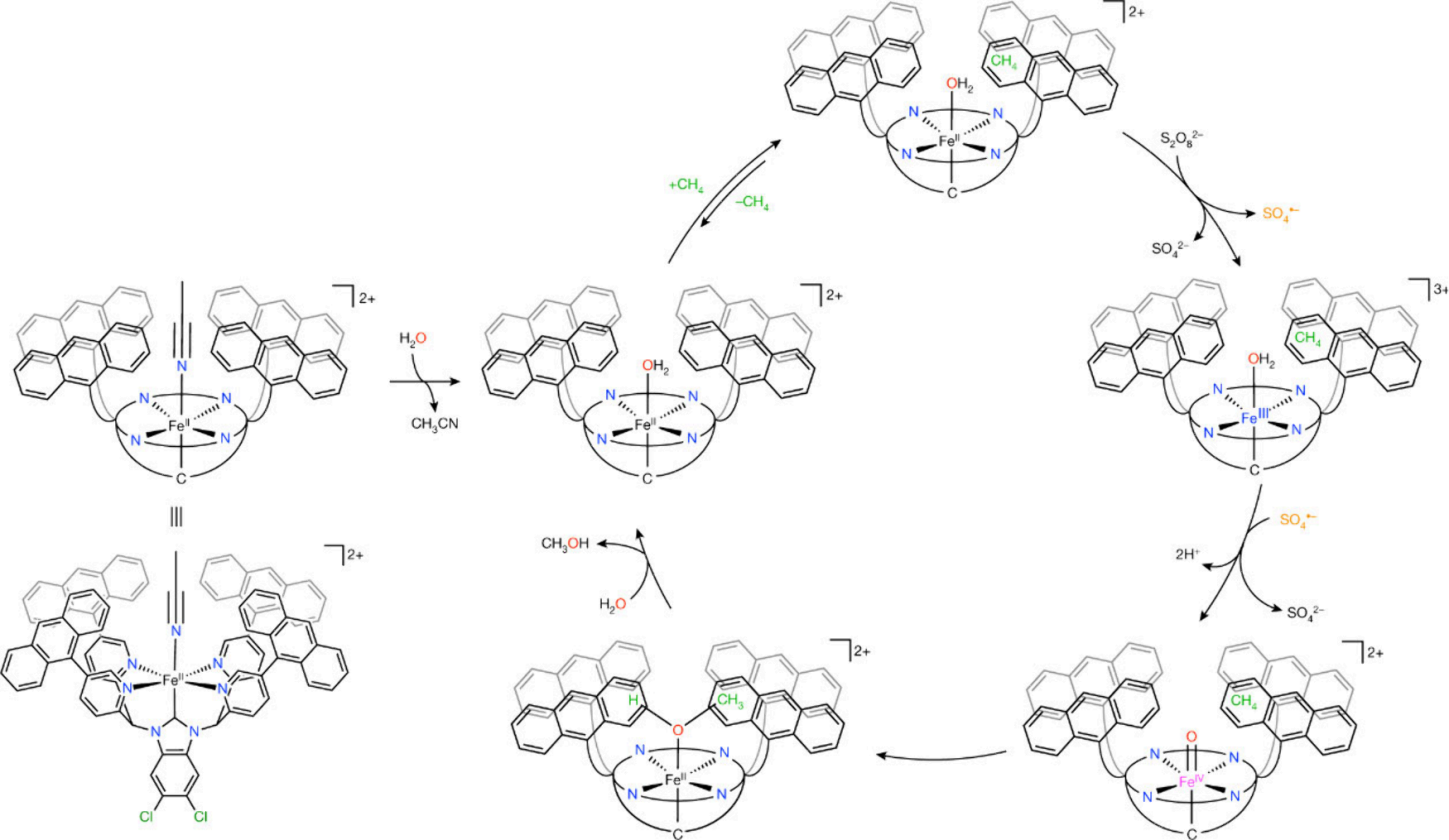
Proposed mechanism for the catch-and-release oxidation of CH_4_ by [Fe^IV^(^R^PY_4_Cl2BIm)­(O)]^2+^ (where R = anthracenyl (Ant)). Reproduced with permission from ref 368. Copyright 2023 Nature.

#### 3.1.3 Synthetic *S* = 2 Fe­(IV)–Oxo Complexes

All of the Fe­(IV)–oxo complexes discussed above exhibit an *S* = 1 ground state.^360^ In contrast, all high-valent Fe­(IV)–oxo intermediates in nonheme iron enzymes (other than OvoA) characterized to date possess an *S* = 2 ground state. Most of the corresponding model complexes with an *S* = 2 spin state have initially been reported in trigonal bipyramidal (TBP) geometries, typically supported by tripodal ligands such as TMG_3_tren,^209^ H_3_buea^3–^,^217^ and (tpa^Ph^)^3–^.^463^ An exception is the *S* = 2 Fe­(IV)–oxo penta-aquo complex characterized by Bakac and co-workers, which was formed from the reaction of [Fe^2+^(H_2_O)_6_]^2+^ with ozone (O_3_).^461^ But due to the very short lifetime of this complex, its reactivity was not studied. In search of more reactive *S* = 2 Fe­(IV)–oxo species, Que and co-workers investigated derivatives of the TPA ligand, where all of the pyridine groups are replaced by weak-field quinoline groups (ligand TQA). Three weak-field equatorial ligands in a six-coordinate Fe­(IV)–oxo complex reduce the energy splitting of the d-orbitals enough to favor the *S* = 2 ground state.^360,366^

In 2020, Hendrich, Borovik and co-workers reported a new variant of the H_3_buea^3–^ ligand called poat^3–^,^465^ which helps understand how hydrogen bonding in the SCS affects the properties of Fe­(IV)–oxo complexes. The corresponding complex [Fe^IV^(poat)­(O)]^−^ (see Scheme 10) was characterized extensively with different spectroscopic methods. EXAFS revealed an Fe–O bond length of about 1.65 Å for this species. Furthermore, NRVS measured the Fe–O vibrational frequency at 843 cm^–1^, and Mössbauer spectroscopy gave an isomer shift of 0.06 mm/s and a quadrupole splitting of 0.39 mm/s for this complex, which are very similar to those observed for the *S* = 2 complex [Fe^IV^(TMG_3_tren)­(O)]^2+^.^209^ Interestingly, the poat^3–^ complex shows a near-IR absorption band at 925 nm (ε ≈ 170 M^–1^ cm^–1^), which is shifted by the addition of group 2 metal ions. The blue shift is more pronounced with smaller metal ions, suggesting they act as Lewis acids and interact with the Fe=O unit.

**Scheme 10:**
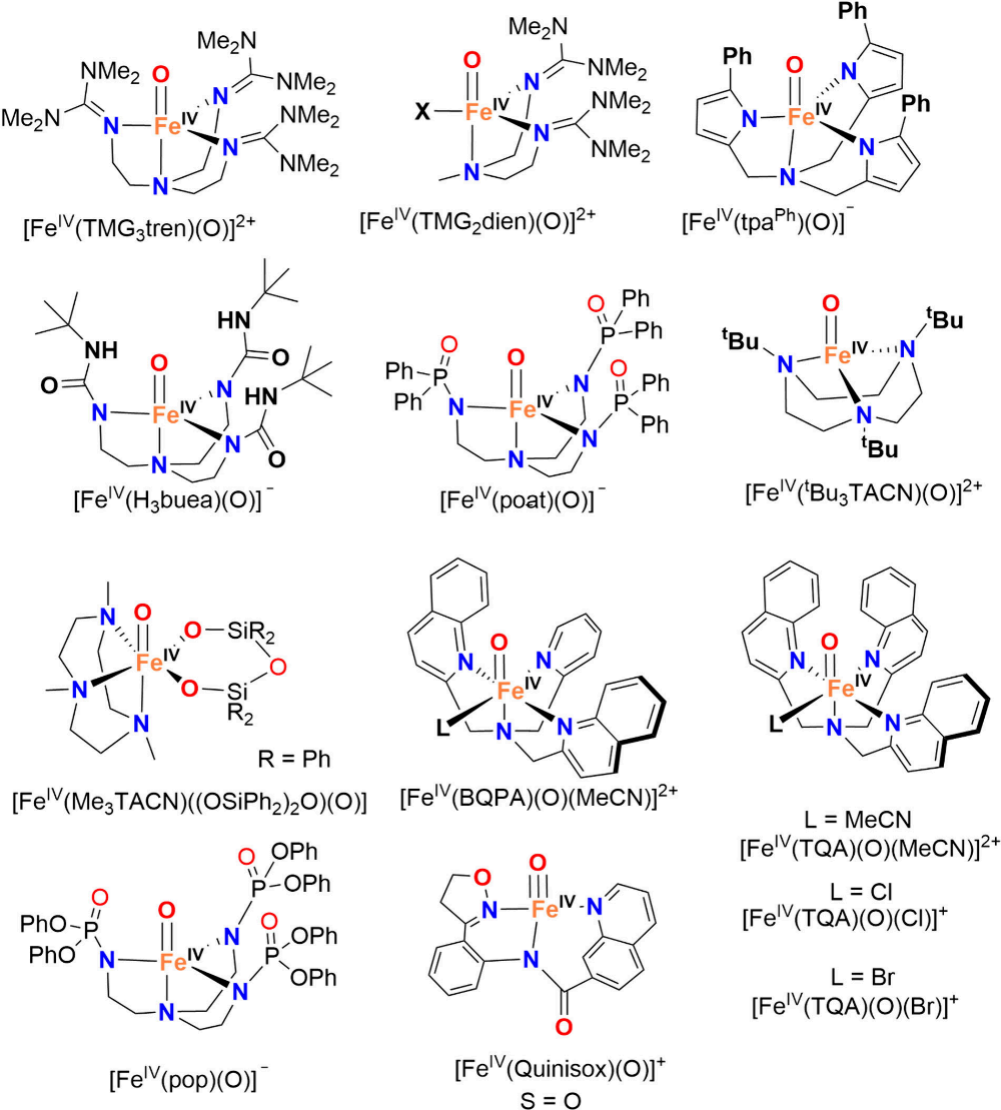
Structures of *S* = 2 and *S* = 0 Fe­(IV)–oxo Complexes

The Ray group reported an *S* = 2 Fe­(IV)–oxo complex in a pseudotetrahedral (PTD) geometry using the ^t^Bu_3_TACN coligand framework (Scheme 10).^460^ A lack of chemical shift in the fluorine NMR and the observation of an identical chromophore in both coordinating and noncoordinating solvents confirmed that neither triflate nor solvent molecules are bound to the iron center. This evidence supports the formulation of this species as a four-coordinate, pseudotetrahedral Fe­(IV)–oxo complex. The intermediate shows a Mössbauer isomer shift of 0.11 mm/s and a quadrupole splitting of 0.96 mm/s. HAA reactivity of this intermediate toward C–H bonds is quite interesting, as this complex exhibits almost similar reactivity toward moderately strong C–H bonds (substrates like toluene or ethylbenzene) compared to the most reactive Fe­(IV)–oxo complexes with TQA or Me_3_NTB coligand frameworks (see above). On the other hand, this complex is almost 100 times less reactive for the oxidation of DHA. To explain these observations, the authors propose an asynchronous PCET mechanism for C–H bond activation (see section 2.4.2.3) by this complex that prevails for substrates with weak C–H bonds like DHA, xanthene, indene, and fluorene, as the reactivity of the intermediate decreases linearly with increasing ionization energy (IE) of the substrate (so ET advances faster than PT).^460^ It was also found from KIE studies that the complex has a very large KIE of 53 for substates with strong C–H bonds like ethylbenzene. On the other hand, the KIE is only 1.2 for DHA as the substrate, which further establishes that two different mechanisms are operating for the HAA reaction by this complex, depending on the strength of the C–H bond that is activated.

Recently, Goldberg and co-workers reported a reactive *S* = 2 Fe­(IV)–oxo complex that is formed from the breaking of the O–O bond in [Fe_2_^III^(μ-O_2_)­(Me_3_TACN)_2_((OSiPh_2_)_2_O)_2_] upon light exposure in frozen solution.^467^ Mössbauer spectroscopy confirmed that this intermediate, [Fe^IV^(Me_3_TACN)­((OSiPh_2_)_2_O)­(O)], has an *S* = 2 ground state, showing an isomer shift of 0.22 mm/s and a quadrupole splitting of −0.23 mm/s (see Table 6). This is the first *S* = 2 Fe­(IV)–oxo complex that was derived directly from dioxygen. DFT calculations predict a high Fe­(III)­O–H BDFE of approximately 98 kcal/mol for this complex, indicating very strong HAA reactivity. Although experimental reactivity studies could not be performed, because the complex remains stable only within a frozen matrix, this calculated BDFE value strongly suggests that this Fe­(IV)–oxo intermediate would be a strong oxidant toward C–H bonds.^467^

**Table 6:** Spectroscopic Properties of Synthetic *S* = 2 Fe­(IV)–oxo Complexes (see Scheme 10 for Structural Drawings)

complex^a^	λ_max_ (nm) [ε (M^–1^ cm^–1^)]	r(Fe=O) (Å)	ν(Fe–O) (cm^–1^)	δ (mm/s)	Δ*E*_Q_ (mm/s)	half-life (298 K)	ref
[Fe^IV^(H_2_O)_5_(O)]^2+^	320 (∼500)			0.38	–0.33	7 s (298 K)	^461^

[Fe^IV^(TMG_3_tren)(O)]^2+^	400 (9800)	1.661	843	0.09	–0.29	4.3 h (243 K)	^209,462^
	825(260)						
	865(250)						

[Fe^IV^(H_3_buea)(O)]^−^	440 (3100)	1.680	799	0.02	0.43	2.2 h (298 K)	^217^
	550 (1900)						
	808(280)						

[Fe^IV^(tpa^Ph^)(O)]^−^	400 (−)	1.62	850	0.09	0.51	1 h (233 K)	^463^
	∼900(−)						

[Fe^IV^(TMG_2_dien)(O)(MeCN)]^2+^	380 (8200)	1.65	807	0.08	0.58	30 min (223 K)	^318^
	805 (270)						

[Fe^IV^(TMG_2_dien)(O)(N_3_)]^+^	412 (9700)		833	0.12	–0.30	24 min (233 K)	^318^
	827 (290)						

[Fe^IV^(TMG_2_dien)(O)(Cl)]^+^	385 (7800)	1.65	827	0.22	0.96	5 min (233 K)	^318^
	803 (290)						

[Fe^IV^(TQA)(O)(MeCN)]^2+^	650 (300)		838	0.24	–1.05	15 min (233 K)	^366^
	900 (75)						

[Fe^IV^(BQPA)(O)(MeCN)]^2+^	850 (200)			0.10	0.66	2 min (233 K)	^328^

[Fe^IV^(TQA)(O)(Cl)]^+^	625 (460)		827	0.22	0.96	5 min (233 K)	^459^
	875 (110)						

[Fe^IV^(TQA)(O)(Br)]^+^	625 (460)		828	0.21	0.94	5 min (233 K)	^459^
	875 (110)						

[Fe^IV^(^t^Bu_3_TACN)(O)]^2+^	356 (7500)	1.66	802	0.11	0.96	20 min (203 K)	^460^
[Fe^IV^(Quinisox)(O)]^+^ (*S* = 0)			965.5 (IRPD)				^464^
[Fe^IV^(poat)(O)]^−^	925 (170)	1.65	843 (NRVS)	0.06	0.39		^465^
[Fe^IV^(pop)(O)]^−^	900						^466^
[Fe^IV^(Me_3_TACN)((OSiPh_2_)_2_O)(O)]			818	0.22	–0.23		^467^
Zeolite Fe^IV^(O) α-O	∼600	1.63	855 (NRVS)				^367^
Long Fe^IV^(O) in MOF			831	0.26	0.57		^369^
Barnet Fe^IV^(O) in cage	875 (61)	1.64	859			21 h (343 K)	^317^

a Ligand nomenclature: TMG_3_tren = tris­[2-(*N*-tetramethylguanidyl)­ethyl]­amine; H_3_buea^3–^ = tris­(*tert*-butylureaylethylene)­aminato; tpa^Ph 3–^ = tris­(5-phenylpyrrol-2-ylmethyl)­amine; TMG_2_dien = 2′,2′-(2,2′-(methylazanediyl)­bis­(ethane-1,2-diyl))­bis­(1,1,3,3-tetramethylguanidine); TQA = tris­(2-quinolylmethyl)­amine; BQPA = 1-(pyridine-2-yl)-*N,N*-bis­(quinoline-2-ylmethyl)­methanamine; ^t^Bu_3_TACN = 1,4,7-tri*tert*-butyl-1,4,7-triazacyclononane; Quinisox^–^ = *N*-(2-(2-isoxazoline-3-yl)­phenyl)­quinoline-8-carboxamide; poat^3–^ = *N*,*N*′,*N*′-(nitrilotris­(ethane-2,1-diyl))­tris­(*P*,*P*-diphenylphosphinic amide); pop^3–^ = hexaphenyl (nitrilotris­(ethane-2,1-diyl))­tris­(phosphoramidate); Me_3_TACN = 1,4,7-trimethyl-1,4,7-triazacyclononane.

Que and co-workers systematically replaced pyridine with quinoline groups in the TPA ligand framework, resulting in hybrid pyridine/quinoline-type ligands.^328^ Their work revealed that the BQPA ligand (containing two quinoline and one pyridine unit) supports an Fe­(IV)–oxo complex that exhibits two distinct spin states. NMR and Mössbauer spectroscopy showed that the complex possesses an *S* = 2 ground state at 233 K, and an *S* = 1 ground state between 150 and 4 K (see Table 6). Although the exact temperature at which the spin crossover occurs could not be determined, this study marks an important experimental demonstration of dual spin-state behavior and supports the concept of two-state reactivity (see section 2.4.2.2). Furthermore, the reactivity of all Fe­(IV)–oxo complexes in the series was also correlated with the DFT-derived reduction potentials of their Fe­(IV)–oxo/Fe­(III)–OH couples. Replacing pyridine groups with the weaker-field quinoline groups was found to favor more positive reduction potentials, which enhances HAA reactivity.

Most synthetic Fe­(IV)–oxo complexes are generated using iodosylbenzene (PhIO) or other chemical oxidants. In contrast, nonheme iron enzymes in nature utilize molecular oxygen (O_2_) to produce *S* = 2 Fe­(IV)–oxo intermediates. Despite significant efforts, replicating this enzyme-like O_2_ activation in synthetic model systems to generate *S* = 2 Fe­(IV)–oxo species has remained a major challenge. Only Goldberg and co-workers have generated a corresponding *S* = 2 Fe­(IV)–oxo complex using dioxygen in a frozen matrix via photochemistry (see above).^467^ In 2023, the Long group reported a breakthrough in this regard: a metal–organic framework (MOF) featuring iron­(II) centers with a local coordination environment resembling that of α-KG-dependent nonheme iron dioxygenases (see Figure 40). Remarkably, this MOF reacts with O_2_ at low temperatures to generate *S* = 2 Fe­(IV)–oxo intermediates capable of catalyzing the conversion of ethane to ethanol at 100 K. This Fe­(IV)–oxo intermediate displays an isomer shift of 0.26 mm/s and a quadrupole splitting of 0.57 mm/s, which is similar to corresponding values previously reported for other *S* = 2 Fe­(IV)–oxo complexes.^369^ Very recently, Austin, Groves, Long, and co-workers explored the reactivity of this intermediate with different radical clock substrates, including norcarane, yielding radical lifetimes in the low-nanosecond regime, consistent with a diffusion-limited rebound mechanism.^468^

**Figure 40:**
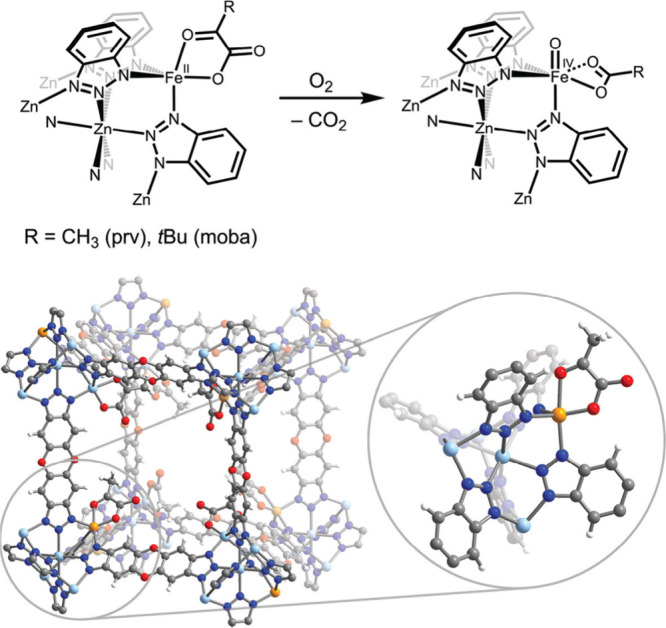
Top: Illustration of the local coordination environment of the iron­(II) sites in FeZn_4_(prv)_4_(btdd)_3_ (R = CH_3_) and FeZn_4_(moba)_4_(btdd)_3_ (R = ^t^Bu) and observed reactivity with O_2_ at low temperatures to form an *S* = 2 Fe­(IV)–oxo species coordinated by acetate or pivalate formed through the decarboxylation of pyruvate (prv) or 3,3-dimethyl-2-oxobutanoate (moba), respectively. Bottom: Illustration of a cubic pore within FeZn_4_(prv)_4_(btdd)_3_ derived using single-crystal X-ray crystallography. Adapted from ref 369. Copyright 2023 AAAS.

A recent study by the Barnett group reported the generation of an *S* = 2 Fe­(IV)–oxo complex encapsulated within an organic cage (see Scheme 11), which is currently the most thermally stable Fe­(IV)–oxo species known.^317^ Remarkably, this complex exhibits a half-life of 21 h in MeCN at 70 °C. The authors claim that this exceptional stability is due to the significant steric hindrance provided by the cage, which effectively suppresses intermolecular reactions with typical oxygen atom acceptors and hydrocarbons possessing weak C–H bonds. Nonetheless, the complex undergoes PCET with 2,4,6-tri-*tert*-butylphenol (TTBP) readily at room temperature, despite the steric bulk of both species. Given the lack of reactivity with substrates such as CHD or DHA, and the steric constraints of both the iron complex and TTBP, a concerted PCET mechanism appears unlikely. Instead, kinetic and spectroscopic analysis supports a stepwise mechanism involving initial proton transfer followed by electron transfer (PT/ET) for TTBP oxidation by the Fe­(IV)–oxo intermediate.

**Scheme 11:**
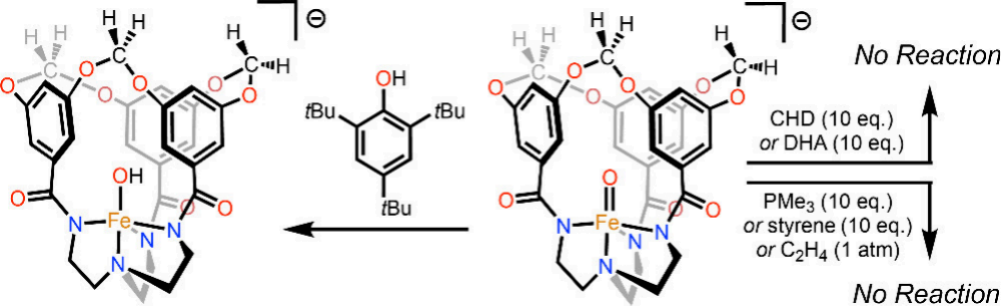
Reactions of the Caged Fe­(IV)–oxo Complex with TTBP (left) and Other Potential HAA or OAT Substrates (right)^a^

a Reproduced with permission from ref 317. Copyright 2025 American Chemical Society.

#### 3.1.4 A Unique *S* = 0 Fe­(IV)–Oxo Complex

Andris and co-workers applied iterative computer-aided ligand design employing DFT calculations to identify potential candidates for singlet Fe­(IV)–oxo complexes.^464^ They then synthesized the most suitable candidates and characterized them by gas-phase IR/vis ion spectroscopy and DFT calculations. The authors reported a novel pseudotetrahedral Fe­(IV)–oxo complex supported by a tridentate ligand monoanion, referred to as Quinisox^–^ (Scheme 10). For the gas phase studies the complex was generated from the precursor ion, [Fe^III^(Quinisox)­(NO_3_)]^+^, by collision-induced dissociation of NO_2_ with xenon, which generates [Fe^IV^(Quinisox)­(O)]^+^. This compound was characterized by IR photodissociation spectroscopy to have an Fe–O stretching frequency of 960.5 cm^–1^. When ^18^O-labeled nitrate was used, the band was observed to down-shift by 36 cm^–1^. The observed Fe–O stretching frequency is much higher than the values typically reported for Fe­(IV)–oxo and Fe­(V)–oxo intermediates, which range between 800–885 cm^–1^ (see Table 6). In fact, the ∼100 cm^–1^ shift to higher frequency in the Fe–O vibration puts it very close to the Fe–N stretching frequency for the Fe≡N triple bond reported in singlet Fe­(IV)−nitrido complexes.^469^ This is the highest Fe–O stretching frequency recorded to date for an Fe­(IV)–oxo complex, consistent with the formation of an *S* = 0 ground state species, allowing for an effective Fe≡O triple bond.

### 3.2 Structure and Reactivity of Terminal Fe­(V)–Oxo Complexes

High-valent iron–oxo species are powerful and reactive molecules that help carry out many important oxidation reactions in nature. Whereas α-KG-dependent enzymes are proposed to function via Fe­(IV)–oxo reactive intermediates, Rieske oxygenases have been proposed to engage transient, nonobservable Fe­(V)–oxo intermediates in the *cis*-dihydroxylation of aromatic compounds and in aliphatic C–H oxidation (see section 1.2 for a detailed discussion).^57,60,67^ As synthetic Fe­(V)–oxo complexes remain exceedingly rare, primarily due to their intrinsic reactivity and fleeting nature, no crystallographic data are currently available, and spectroscopically well-characterized examples are limited. Nonetheless, advances in biomimetic chemistry have led to the development of catalyst systems capable of generating high-valent Fe­(V)–oxo species, often from Fe­(III)–OOH precursors via heterolytic O–O bond cleavage.^470^

Collins and co-workers spent many years exploring the coordination chemistry of a group of tetraamido macrocyclic ligands, known as the TAML^4–^ ligand family, for their ability to support high-valent metal–oxo and related intermediates (Scheme 12).^471,472^ Their work resulted in the successful synthesis and detailed characterization of a series of [Fe^V^(TAML)­(O)]^−^ complexes, representing the first well-defined examples of Fe­(V)–oxo intermediates. The [Fe^V^(TAML)­(O)]^−^ complex can be synthesized in nearly quantitative yield by reacting its iron­(III) precursor with *m*-CPBA.^365^ The resulting intermediate displays a mass-to-charge ratio (*m*/*z*) signal of 442.2 in mass spectrometry (MS), along with an isotopic pattern consistent with the molecular formula [Fe^V^(TAML)­(O)]^−^. Spectroscopically, the complex exhibits two UV–vis absorption bands at 445 nm (ε = 5400 M^–1^ cm^–1^) and 630 nm (ε = 4200 M^–1^ cm^–1^). Its EPR spectrum is rhombic, with g-values of 1.99, 1.97, and 1.74, indicative of an *S* = 1/2 spin state (see Figure 41). The iron­(V) oxidation state was further confirmed by Mössbauer spectroscopy, which revealed a quadrupole doublet with an isomer shift of −0.42 mm/s and an unusually large quadrupole splitting of 4.25 mm/s (see Figure 41). These Mössbauer parameters are clearly distinct from those of all mononuclear Fe­(IV)–oxo complexes discussed in this review, with [Fe^V^(O)­(TAML)]^−^ exhibiting the most negative isomer shift and the largest quadrupole splitting.^473^

**Scheme 12:**
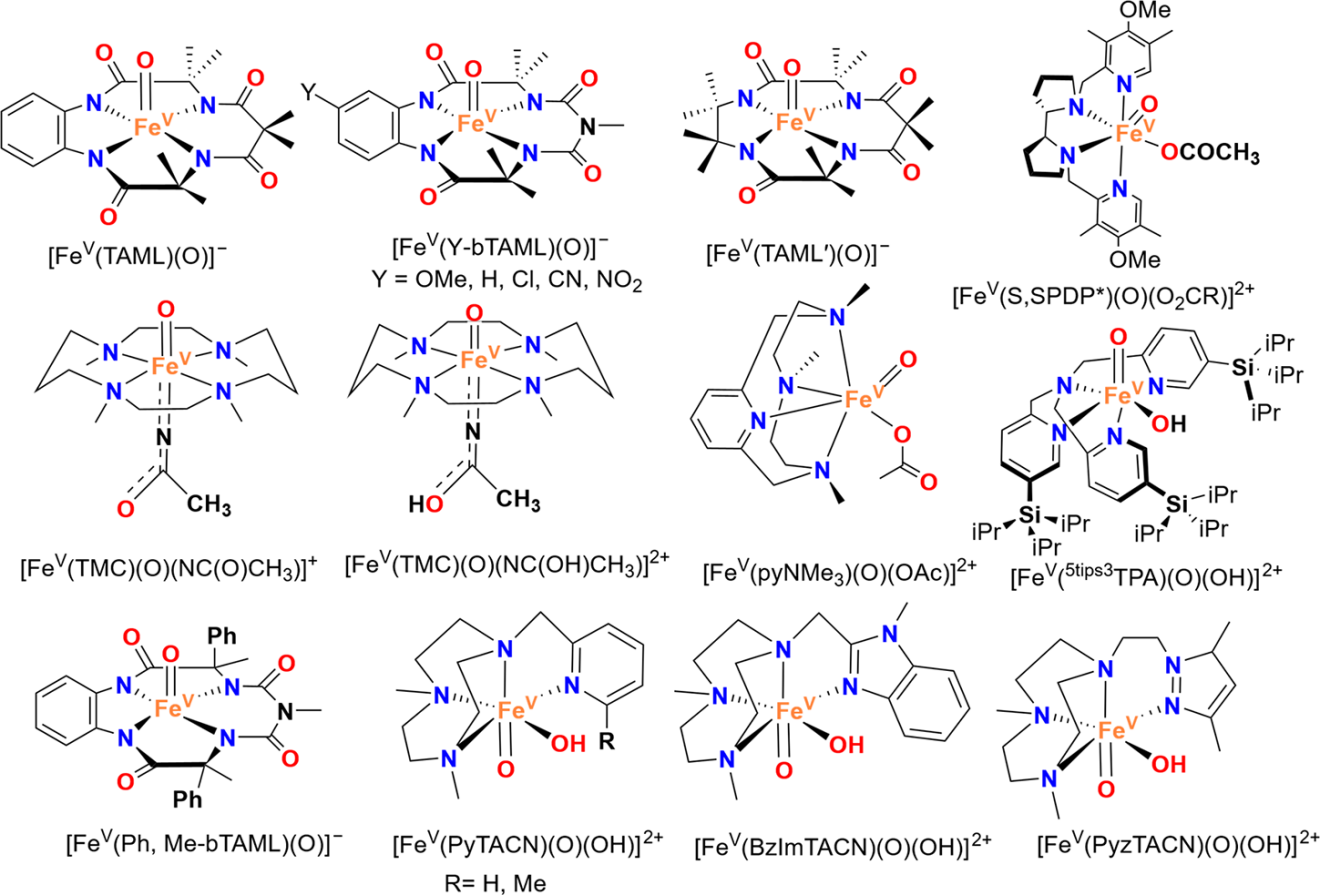
Structures of Fe­(V)–oxo Complexes

**Figure 41:**
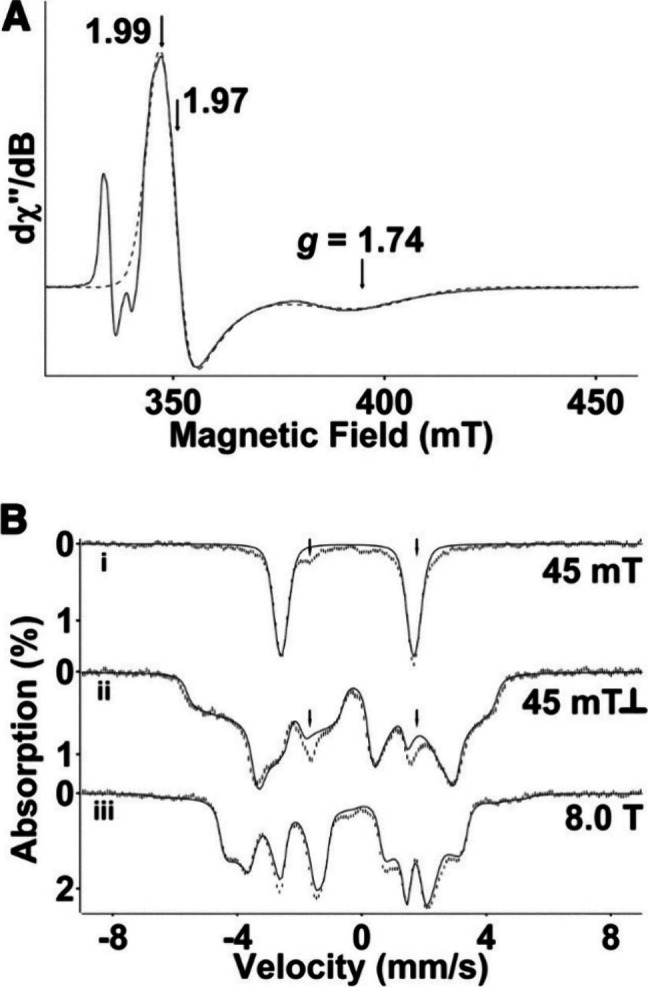
(A) X-band EPR spectrum of about 2 mM ^57^Fe-enriched [Fe^V^(TAML)­(O)]^−^ in *n*-butyronitrile. Conditions: *T* = 28 K, 9.66 GHz microwave frequency, 0.02 mW microwave power, 1 mT modulation. The dashed line is a spectral simulation for the majority species. (B) Mössbauer spectra of about 2 mM ^57^Fe-enriched [Fe^V^(TAML)­(O)]^−^ in *n*-butyronitrile recorded at 140 K (i) and 4.2 K (ii and (iii) in magnetic fields as indicated; incident γ beam perpendicular (ii) and parallel (i and (iii) to the applied magnetic field. Reproduced with permission from ref 365. Copyright 2007 AAAS.

The neutral ligand TMC has also been reported to support the formation of Fe­(V)–oxo complexes as shown by Que, Munk and co-workers.^474^ This intermediate is formed by one-electron oxidation of [Fe^IV^(TMC)­(O)­(MeCN)]^2+^ with *tert*-butyl hydroperoxide (^*t*^BuOOH) in the presence of a strong base. The evidence for the formation of an Fe­(V) intermediate comes from the characteristic EPR signal for *S* = 1/2 species with resonances at g = 2.05, 2.01, and 1.97 (see Table 7) and a Mössbauer isomer shift of 0.10(4) mm/s, more negative than the δ values (0.15–0.20 mm/s) typically observed for analogous Fe­(IV)–oxo TMC complexes. The Fe­(V) intermediate is proposed to form by the attack of ^t^BuOO^–^ on the carbon atom of the bound MeCN, followed by O–O bond cleavage. The resulting Fe­(V)–oxo species was therefore assigned an axial acetylimido ligand, giving the formula [Fe^V^(TMC)­(O)­(NC­(O)­CH_3_)]^+^ for the intermediate, further supported by DFT calculations (see Scheme 12). The presence of the strongly basic axial acetylimido ligand was confirmed by the ∼50 cm^–1^ lower (789 cm^–1^) Fe–O stretching frequency in the Fe­(V)–oxo intermediate, compared to a value of 839 cm^–1^ observed for the Fe­(IV)–oxo precursor. This is likely due to the Fe–N bond gaining partial Fe=N character, which would weaken the Fe­(V)–oxo bond.

**Table 7:** Spectroscopic Properties of Synthetic Fe­(V)–oxo Complexes (See Scheme 12 for Structural Drawings)

complex^a^	g values	δ(Δ*E*_Q_) (mm/s)	r(Fe=O) (Å)	ν(Fe–O) (cm^–1^)	ref
[Fe^V^(TAML)(O)]^−^	1.99, 1.97, 1.74	–0.42 (−4.25)	1.58		^365^
[Fe^V^(bTAML)(O)]^−^	1.98, 1.94. 1.73	−0.44 (–4.27)	1.59	862 (Δ^18^O-36)	^454,455^
[Fe^V^(TAML′)(O)]^−^	2.02, 1.98, 1.85		1.63	886 (Δ^18^O-37)	^244^
[Fe^V^(TAML′)(O)]^−^ + 2H^+^	2.02, 1.98, 1.85		1.59	890 (Δ^18^O-37)	^244^
[Fe^V^(TMC)(O)(NC(O)Me)]^+^	2.05, 2.01, 1.97	+0.10 (−0.2)		798 (Δ^18^O-35)	^474^
[Fe^V^(TPA*)(O)(OOC(O)R)]^2+^	2.07, 2.01, 1.96				^493^
[Fe^V^(^5tips3^TPA)(O)(OH)]^2+^				827 (IRPD)	^494^
[Fe^V^(S,SPDP*)(O)(O_2_CR)]^2+^	2.07, 2.01, 1.96				^493^
[Fe^V^(PyNMe_3_)(O)(O_2_CR)]^2+^	2.07, 2.01, 1.95	0.06 (1.0)		815 (Δ^18^O-32)	^470^
[Fe^IV^(Me_3_TACN)(O)(Cl-acac^+•^)]^2+^	1.97, 1.93, 1.91				^495^
[Fe^V^(Ph, Me-bTAML)(O)]^−^	2.00, 1.98, and 1.83	0.58 (4.11)			^485^

a Ligand nomenclature: bTAML^4–^ = 3,3,6,9,9-pentamethyl-3,4,8,9-tetrahydro-1*H*-benzo­[*i*]­[1,3,5,8,11]­pentaazacyclotridecine-2,5,7,10­(6*H*,11*H*)-tetraone; TAML′ ^4–^ = 2,2,5,5,6,6,9,9,12,12-decamethyl-1,4,7,10-tetraazacyclotridecane-3,8,11,13-tetraone; PDP* = bis­(3,5-dimethyl-4-methoxypyridyl-2-methyl)-(*S*,*S*)-2,2′-bipyrrolidine; TPA* = tris­(3,5-dimethyl-4-methoxypyridyl-2-methyl)­amine.

In 2015, Munk, Que, Costas and co-workers reported the reaction of [Fe^II^(PyNMe_3_)­(OTf)_2_] with peracetic acid (AcOOH), which led to a very reactive intermediate formulated as [Fe^V^(PyNMe_3_)­(O)­(OAc)]^2+^ (Scheme 12).^470^ This species is capable of hydroxylating cyclohexane with a second-order rate constant of 2.8(1) M^–1^ s^–1^ at −40 °C. Compared to other nonheme iron oxidants, this intermediate exhibits the highest rate of cyclohexane oxidation. Despite its high oxidative reactivity, this intermediate has been characterized by a variety of spectroscopic methods. The proposed Fe­(V)–oxo species displays a strong absorption band at 490 nm (ε ≈ 4500). In the EPR spectrum, it gives rise to two *S* = 1/2 species: (a) a minor component (∼5% of the total Fe) with g = 2.20, 2.19, 1.99, which can reasonably be attributed to a low-spin Fe­(III) center (likely the initial [Fe^III^(PyNMe_3_)­(OOAc)]^2+^ adduct); and (b) a major component (∼40% of the total Fe) with g = 2.07, 2.01, 1.95 (see Table 7) that is associated with the Fe^V^(O)­(OAc) intermediate. These g values are similar to those reported previously by Talsi and co-workers for the Fe­(V)–oxo intermediates derived from the TPA* and PDP* coligand complexes (see Scheme 12) with peracids. The EPR spectrum of a ^57^Fe-enriched sample of the Fe^V^(O)­(OAc) intermediate further confirmed the presence of an iron-derived *S* = 1/2 species due to splitting of the *g* = 2.01 signal into a doublet, arising from the hyperfine coupling with the ^57^Fe nucleus (*I* = 1/2). Mössbauer spectroscopy showed an isomer shift between −0.06 and −0.08 mm/s for this species, which is lower than the +0.09 mm/s value observed for the Fe­(IV)–oxo counterpart. In 2018, Fan and co-workers reported detailed spectroscopic and DFT studies on the Fe^V^(O)­(OAc) intermediate.^475^ Based on DFT calculations, the species is proposed to have predominant Fe^V^(O)­(OAc) character, but the ground state was proposed to also have contributions from the Fe^IV^(O)­(^•^OAc) and Fe^III^(OOAc) electronic states, in a so-called “catalytic troika”. This concept was previously invoked in the carboxylic-acid-assisted mechanism of O–O bond breaking of acylperoxoiron­(III) for Fe­(TPA) and Fe­(TPA*) catalysts. The distance between the O atom of the Fe=O unit and the carbonyl O atom of the OAc group in the Fe^V^(O)­(OAc) intermediate is calculated to be 2.04 Å. Decreasing the O–O bond distance below 2.04 Å leads to the acylperoxoiron­(III) isomer, while stretching the O–O bond beyond 2.04 Å evolves the system toward the Fe^IV^(O)­(^•^OC­(O)­R) isomer, with complete O–O bond homolysis.^475^

In contrast to the computational results by Fan and co-workers, a computational study by Ye and co-workers supports an active species that is best described as having predominant [Fe^IV^(O···^•^OC­(O)­CH_3_)]^2+^ character, where the *S*_Fe_ = 1 iron­(IV) center is antiferromagnetically coupled to an O–O^•^ radical.^476^ In this electronic configuration, the remaining approximately half σ-bond in the O–O group allows the species to have two electron-accepting orbitals (α-σ*­(O–O) and β-Fe­(d_xz_)) that can simultaneously engage the doubly occupied donor orbitals of the substrate (σ­(C–H) or π­(C–C)), thereby enabling both C–H and C=C bond oxidation. In any case, these findings highlight how minor changes in ligand framework or reaction conditions can lead to different iron–oxo oxidants with distinct reactivities.

Sen Gupta and co-workers reported a series of Fe­(V)–oxo studies over the years using the bTAML^4–^ ligand framework.^477^ This ligand differs from the prototype first-generation TAML^4–^ activator by the replacement of the −CMe_2_ unit in the six-membered macrocyclic subring with an −NMe group (see Scheme 12). The corresponding intermediate, [Fe^V^(bTAML)­(O)]^−^, is sufficiently stable and can be generated quantitatively, and its reactivity was studied at room temperature. The EPR spectrum of this intermediate exhibits a rhombic *S* = 1/2 signal with g values of 1.983, 1.935, and 1.726 (see Table 7). Spin quantification indicated essentially complete conversion of the starting Fe­(III) complex to the corresponding Fe­(V)–oxo intermediate. The Möss-bauer spectrum of this intermediate shows a doublet with an isomer shift of δ = −0.44 mm/s and a quadrupole splitting of Δ*E*_Q_ = 4.27 mm/s.^454^ Sen Gupta and co-workers also reported the first Fe­(V)–oxo intermediate generated in H_2_O.^478^ Interestingly, the reactivity of the Fe­(V)–oxo complex toward the oxidation of toluene can be tuned by varying the amount of water in the H_2_O/CH_3_CN solvent mixture. The authors observed that when the reaction of this intermediate with toluene is carried out in a 70:30 H_2_O/CH_3_CN mixture, the rate of toluene oxidation increases about sixty-fold. They claimed that the enhanced reactivity of this intermediate in the presence of water is due to a stabilization of the transition state during the HAA step from the substrate’s C–H bond. Recently, Sen Gupta and co-workers reported a series of Fe­(V)–oxo complexes with the ligands Y-bTAML^4–^ (Y = H, F, Cl, CN and NO_2_; see Scheme 12). This new series of intermediates was characterized using UV–vis absorption and EPR spectroscopy, and mass spectrometry. The EPR spectra are remarkably similar across all complexes, and the observed *g* values are consistent with those previously reported for the bTAML^4–^ intermediate. The dramatic differences observed in the UV–vis spectra between these species were proposed to originate from significant changes in the electronic structure, resulting from perturbations of the equatorial ligand. Sen Gupta and co-workers also explored the electrochemical^479^ and photochemical generation^480^ of their Fe­(V)–oxo intermediates and studied their reactivity. Recently, they explored the use of dioxygen and H_2_O_2_ for generating their Fe­(V)–oxo intermediates instead of using more traditional oxidants like *m*-CPBA or NaOCl,^481^ and they also explored the solvent-free hydroxylation of C–H bonds by their Fe­(V)–oxo intermediates.^482^

In 2020, Kim, Fukuzumi, Nam and co-workers investigated the effect of the protonation state of [Fe^V^(TAML′)­(O)]^−^ (see Scheme 12) on the OAT and oxidation (ET) reactions catalyzed by this intermediate.^244^ Protonation of the Fe­(V)–oxo complex dramatically enhances the rates of OAT and ET. For example, in the presence of 200 mM HOTf, the rate of oxidation of *p*-CN-thioanisole increases by approximately 10^7^-fold compared to the parent complex. The authors also found that the one-electron reduction potential of the intermediate is shifted positively with the addition of acid, which explains the increase in reactivity. Based on the spectroscopic characterization by rRaman and EPR spectroscopy, EXAFS, and DFT calculations, protons are bound to the ligand backbone and not the Fe=O unit. The EXAFS data showed a slightly shorter Fe–O bond distance of 1.59 Å for the protonated species compared to 1.63 Å for the parent complex, in agreement with this proposal. In addition, the Fe–O stretching frequency (determined by rRaman) of the protonated species was found to be shifted by ∼4 cm^–1^ to higher energy. The authors propose that under their experimental conditions, the TAML′^4–^ ligand is in fact doubly protonated at two of the carbonyl groups of the ligand, whereas the Fe=O unit is not protonated.^244^

In 2023, Nordlander, Costas and co-workers reported a series of Fe^V^(O)­(OH) complexes with different monosubstituted TACN-based ligands.^483^ Based on their findings, the authors claimed that the steric bulk of the ligands influences the relative reactivities of the two oxo/hydroxo tautomers (see Scheme 12) of the high-valent oxidant in C–H bond hydroxylation. These catalysts efficiently hydroxylate alkane C–H bonds using hydrogen peroxide. The observed large H/D kinetic isotope effects for C–H bond hydroxylation, high normalized tertiary/secondary (C3/C2) selectivity in adamantane oxidation, and substantial stereoretention in the oxidation of *cis*-1,2-dimethylcyclohexane were all taken as evidence that these reactions proceed via a metal-based oxidant.

In 2023, Sen Gupta and co-workers reported the HAA reactivity of the Fe­(IV)–oxo coligand cation radical and its Fe­(V)–oxo valence tautomer using bTAML^4–^ type ligand frameworks.^484^ From Mössbauer spectroscopy, an isomer shift of 0.13 mm/s and a quadrupole splitting of 3.36 mm/s were observed for Fe­(IV)­(Y-bTAML^•+^)­(O) with Y = OMe, which is in stark contrast to the isomer shift (δ = −0.44 mm/s) of the Fe­(V)–oxo intermediate with bTAML^4–^ (see Scheme 12).^454^ The isomer shift of this new intermediate is very close to that previously reported for Fe­(IV)–oxo complexes with the bTAML^4–^ ligand framework. EPR spectroscopy showed g values of 1.99, 1.94, and 1.74 for the new intermediate, which correspond to an *S* = 1/2 species. Based on these results, Sen Gupta and co-workers claimed the presence of an Fe­(IV)–oxo intermediate with an overall spin *S* = 1/2, which would then be described as an Fe­(IV)­(bTAML^•+^)­(O) intermediate where the coligand carries one unpaired electron, and the spins are antiferromagnetically coupled. Sengupta and co-workers found that the OAT reactivity toward olefins by the Fe­(IV)­(Y-bTAML^•+^)­(O) intermediate with Y = OMe is almost similar to the reactivity of the complexes with Y = Cl or CN, which suggests that OAT depends only on the reduction potential of the iron–oxo complexes. In contrast, for the HAA reaction, p*K*_a_ also plays a role along with the reduction potential (see section 2.4).

Recently, an Fe­(V)–oxo complex supported by another modified bTAML^4–^ ligand was synthesized and characterized by Sen Gupta and co-workers.^485^ Here, a phenyl group replaces a methyl substituent on either side of the biuret moiety (see Scheme 12). In earlier studies, it was found that the formation of the Fe­(V)–oxo intermediate using bTAML^4–^ in CH_3_CN upon oxidation with *m*-CPBA or NaOCl proceeds via a μ-oxo-diiron­(IV) dimeric intermediate,^454,478,479,486–488^ a thermodynamic sink that limits catalytic turnover. This reaction was prevented in the new study by the addition of a phenyl ring to the ligand framework. This new modified ligand system efficiently generates an Fe­(V)–oxo intermediate entirely in water. Remarkably, the reactivity of this species in water is several hundred times greater than in CH_3_CN as the solvent. Furthermore, this is the first demonstration that such a [Fe­(Ph, Me-bTAML)] framework can utilize H_2_O_2_ in pure water to selectively oxidize a wide range of substrates bearing tertiary C–H bonds (including natural products) and C=C bonds. From EPR and Mössbauer spectroscopy, it was found that this new intermediate displays an isomer shift of −0.58 mm/s, a quadrupole splitting of 4.11 mm/s, and g values of 2.00, 1.98, and 1.83, which are similar to those previously reported for Fe­(V)–oxo intermediates in the bTAML^4–^ ligand family (see Table 7).^489^

In 2024, Luis, Costas, Company and co-workers reported the intramolecular C–H oxidation by a series of Fe­(V)–oxo-carboxylato intermediates.^490^ Theoretical and spectroscopic investigations indicated that the reaction of the ferrous precursor complexes with peracids generates a family of Fe­(V)­(O)­(carboxylate) species, [Fe^V^(PyNMe_3_)­(O)­(OCOR)]^2+^ (R = CH_2_CH_2_CH­(CH_3_)_2_, (CH_2_)_7_CH_3_, CH_2_C­(CH_3_)_3_, CH_2_CH­(CH_3_)_2_), each bearing a carboxylate ligand of distinct substitution. These intermediates were characterized by UV–vis, EPR and Mössbauer spectroscopy, and cold spray ionization mass spectrometry (CSI-MS). From the Mössbauer data, these intermediates have isomer shifts of −0.06 mm/s and quadrupole splittings of 1.00 mm/s. EPR spectroscopy further supports an *S* = 1/2 ground state for these complexes, showing rhombic EPR spectra with g = 2.07, 2.01, 1.95, which is similar to those of previously reported Fe­(V)–oxo complexes (see Table 7). Remarkably, the self-decay pathway of these intermediates proceeds via intramolecular γ-C–H oxidation of the carboxylates’ alkyl chains, yielding γ-lactones in a stereoretentive, site-selective fashion (see Scheme 13). The authors also found that these intermediates can undergo intermolecular HAT and OAT reactions during the lactonization process. The use of the OAT substrate, 1-octene, resulted in the generation of an epoxide product; interestingly, the formation of lactone was stopped as soon as the olefin was added. On the other hand, the presence of an external substrate with a strong C–H bond, like cyclohexane, resulted in intramolecular oxidation of the tertiary C–H bond in the γ-position of the carboxylate rather than oxidation of the external substrate. Only a minor amount of cyclohexane oxidation was obtained, depending on the strength of the internal C–H bond.

**Scheme 13:**
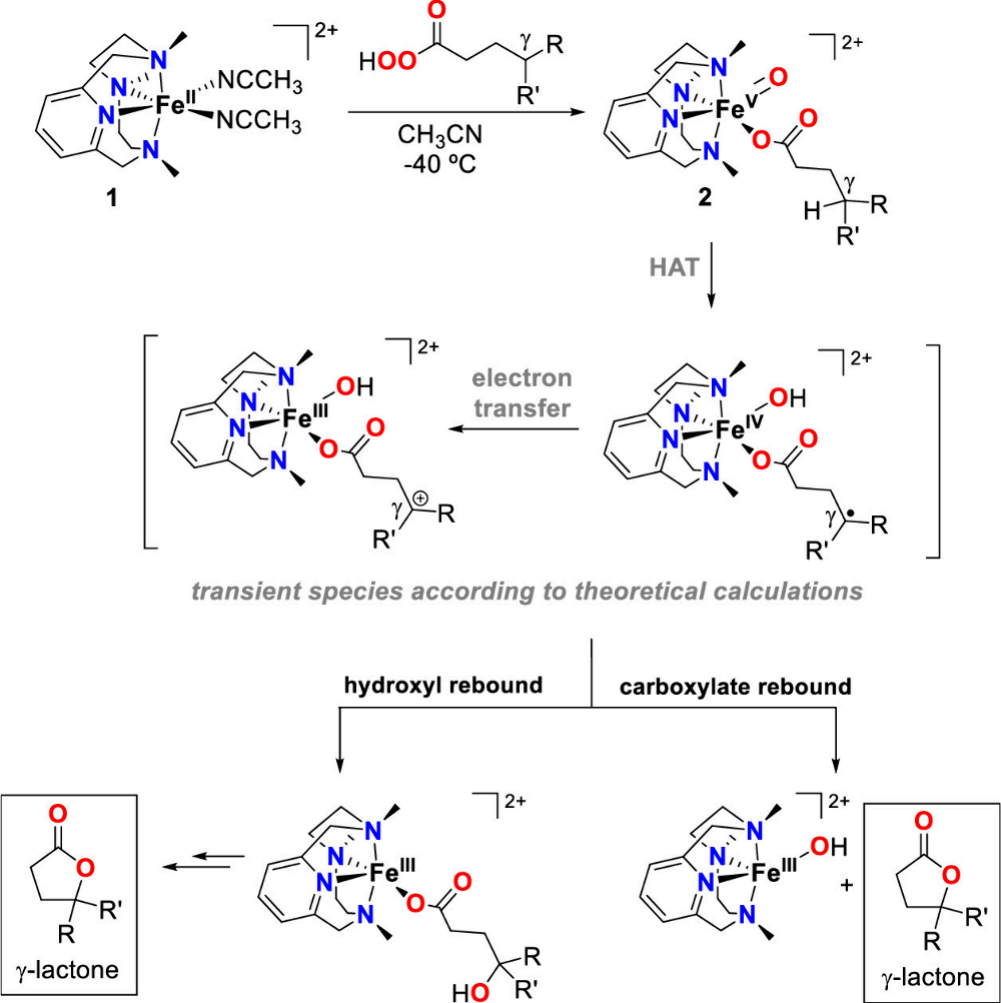
Proposed Mechanism for the Lactonization Reaction Carried out by Fe­(V)–Oxo-carboxylato Intermediates^a^

a Reproduced with permission from ref 490. Copyright 2024 American Chemical Society.

In 2024, Costas and co-workers explored *syn*-dihydroxylation reactions of a broad range of naphthalene derivatives using previously reported [Fe^V^(^5tips3^TPA)­(O)­(OH)]^2+^ (tips = triisopropyl-silyl) complexes (Scheme 12), which mimics NOD activity.^491^ In this study, the authors also modified one of the pyridine arms in the tripodal ligand framework to systematically investigate the role of the steric factor for reactivity. The removal of a tips group from one of the pyridines resulted in a decrease in yield. In addition, the replacement of one pyridine with a 6-methylpicoline provided a lower yield and lower chemoselectivity. The authors further added an electron-donating NMe_2_ group to one of the tips-pyridine units, which also resulted in a poorer catalyst. This suggests that reducing electrophilicity undermines the catalyst’s arene dihydroxylation activity. Based on these results, the authors concluded that [Fe­(^5‑tips3^TPA)] is the most effective catalyst in this series, and they then used this complex to evaluate its substrate scope. The paper further claims that the high electrophilicity of a *cis*-Fe^V^(O)­(OH) intermediate generated using H_2_O_2_ enables overcoming the kinetic inertia imposed by aromaticity and may explain the unusually high *syn*-dihydroxylation chemoselectivity of the [Fe^V^(^5tips3^TPA)­(O)­(OH)]^2+^ complexes among other known naphthalene-oxidizing reagents. It was further proposed that once the first oxidation occurs, a second olefinic site is activated and undergoes smooth dihydroxylation, yielding tetrahydroxylated products in moderate to good yields. The catalyst [Fe^V^(^5tips3^TPA)­(O)­(OH)]^2+^ performs its reactivity on a fairly broad substrate scope, affording access to a variety of densely functionalized products.

Recently, Kovacs and co-workers reported the generation of two formally Fe­(V)–oxo intermediates, [Fe^V^(S_2_^Me2^N_3_(Pr,Pr)­(O)]^+^ and [Fe^V^(S_2_^Me2^N_3_(Et,Pr)­(O)]^+^.^492^ They investigated how OAT to the *cis*-thiolate (with respect to the Fe=O unit) sulfur of the coligand occurs in these complexes using low-temperature kinetic and spectroscopic measurements and computational studies. Reaction of pyridine N-oxide (PNO) with [Fe^III^(S_2_^Me2^N_3_(Pr,Pr))]^+^ leads to the formation of a stable PNO adduct, which was characterized by single crystal X-ray crystallography. Here, PNO does not transfer its oxo atom to the thiolate sulfur, but binds reversibly to the Fe­(III) center, as confirmed by variable-temperature UV–vis absorption spectroscopy. On the other hand, application of PhIO led to the formation of the singly oxygenated ferric sulfenate compound, [Fe^III^(η^2^-S^Me2^O)­(S^Me2^)­N_3_(Pr,Pr)]^+^, at ambient temperatures. The authors further monitored this reaction at −80 °C, using different O-atom donors like PhIO, pentafluoro-iodosyl benzene (PFIB), iodoxybenzene (PhIO_2_) and isopropyl 2-iodoxybenzoate (IBX-ester) to trap the high-valent intermediate that precedes sulfenate formation. Ultimately, the intermediate was trapped using cryo-annealing (i.e, warming to −80 °C, followed by freezing in liquid N_2_). Low-temperature Mössbauer and EPR spectroscopy showed that a new metastable intermediate forms when the initially formed O-atom donor adduct decays, and before sulfur oxidation to the sulfenate final product occurs. This intermediate shows a negative isomer shift of −0.11 mm/s, a quadrupole splitting of 1.96 mm/s, and g-values of 2.22, 2.15, and 1.93, all consistent with a formal Fe­(V) oxidation state. On the other hand, DFT-optimized structures of this intermediate are consistent with an *S* = 1 Fe­(IV)–oxo center that is antiferromagnetically coupled to a radical delocalized over the two *cis*-thiolate groups of the coligand. Over time, this intermediate then mediates sulfur oxygenation.^492^

## 4 Recent Developments in Terminal Cobalt–Oxo Complexes

### 4.1 Structures of Cobalt–Oxo Complexes

Cobalt­(IV)–oxo complexes have been suggested as intermediates for a variety of catalytic processes. Their high d-electron count (d^5^) results in weaker, potentially more reactive cobalt–oxygen bonds compared to their iron counterparts. These weak bonds make Co­(IV)–oxo, or possibly Co­(III)–oxyl, intermediates potentially useful catalysts for a number of reactions, including water oxidation,^496,497^ C–H activation,^498^ and olefin epoxidation^499^ as well as stoichiometric O_2_ reduction.^500^ However, the same properties that could improve the catalytic abilities of formally Co­(IV)–oxo complexes also make them difficult to isolate, meaning that only a few spectroscopically well-characterized high-valent Co­(IV)–oxo complexes exist. Co­(III)–oxyl character could lead to instability, uncontrolled radical reactivity, and potentially Fenton-like chemistry. Some early complexes with the potential to form Co­(IV)–oxo species were discussed in a 2013 review,^300^ and many of the spectroscopically well-characterized Co­(IV)–oxo intermediates were covered in a 2020 review by one of us.^3^ Here we discuss both recently introduced high-valent cobalt–oxo complexes and new insights into known complexes (see Scheme 14 and Table 8 for an overview). Importantly, it should be noted that while this review largely treats Co­(IV)–oxo and Co­(III)–oxyl species as distinct valence tautomers, it is also possible that the ground state of a given complex corresponds to a linear combination of those two electronic states. Recent theoretical work has suggested some ground state mixing between Co­(IV)–oxo and Co­(III)–oxyl states, with the degree of oxo versus oxyl character likely playing a role in the reactivity of these short-lived intermediates.^501–503^

**Scheme 14:**
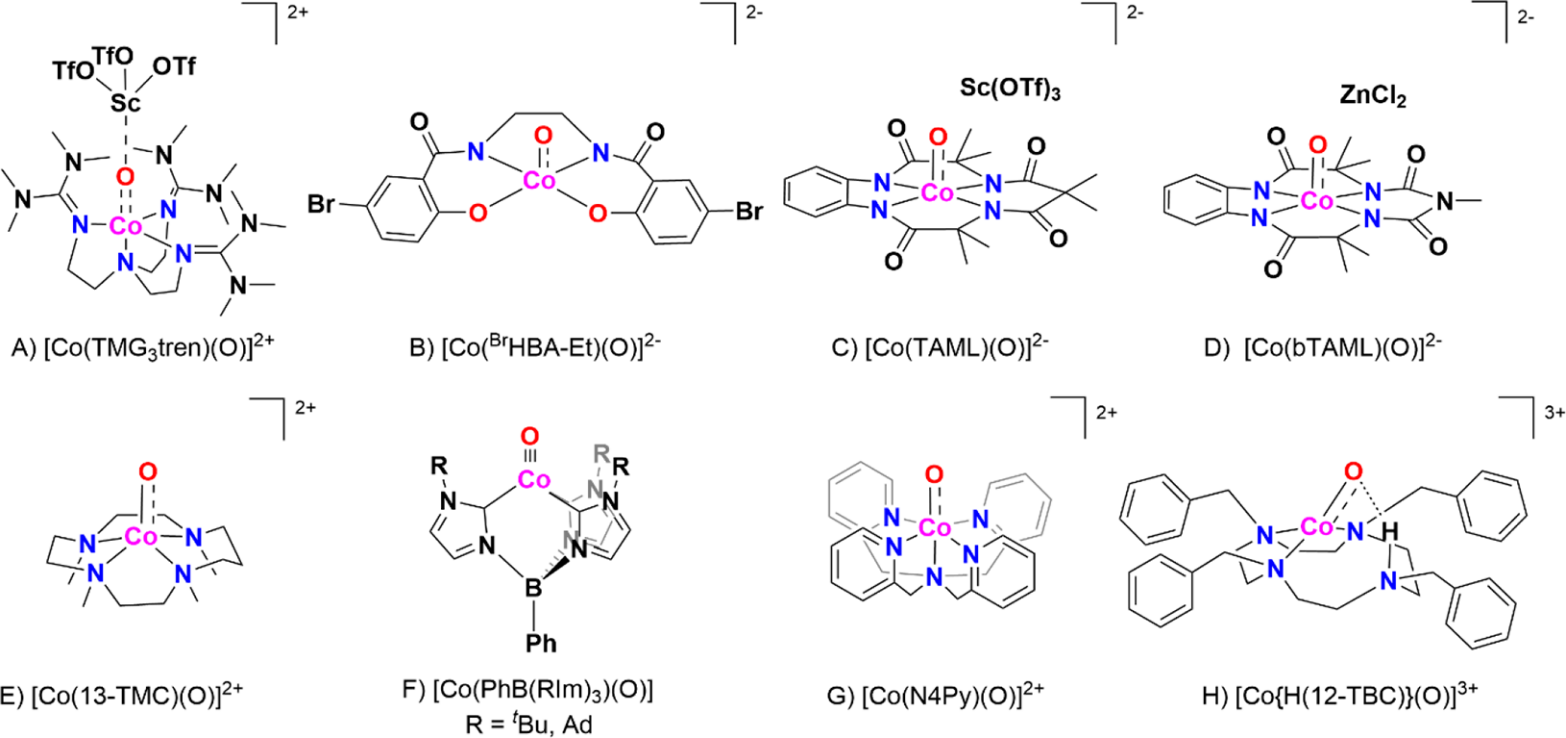
Proposed Structures of High-Valent Cobalt–Oxo Complexes^a^

a Ligand nomenclature: ^Br^HBA-Et^4–^ = *N,N*′-(ethane-1,2-diyl)­bis­(5-bromo-2-hydroxybenzamide; PhB­(*^t^* BuIm)_3_^–^ = tris­(imidazole-2-ylidene)­borate, Ad = adamantane; 12-TBC = 1,4,7,10-tetrabenzyl-1,4,7,10-tetraazacyclodecane.

**Table 8:** Selected Spectroscopic Properties of High-Valent Cobalt–Oxo Complexes (see Scheme 14 for Structural Drawings)

complex	total spin (*S*)	λ_max_ (nm) [ε (M^–1^ cm^–1^)]	r(Co–O) (Å)	ν(Co–O) (^18^O) (cm^–1^)	*g*_1_	*g*_2_	*g*_3_	ref
[Co^IV^(TMG_3_tren)(O)Sc(OTf)_3_]^2+^	3/2	489 (495), 810 (100)	1.85		5.80	5.80	2.58	^504,506^
[Co^III^(TAML^3–•^)(OH-LA)]^−^	1/2^a^	600 (7200)	2.3		2.1095	2.0268	2.6011	^252^

[Co^IV^(H-TAML^3–^)(O-LA)]^−^	1/2	353, 700, 950	1.68 (LA = Y)		1.9998	1.9840	2.0090	^252^
			1.72 (LA = Sc)					

[Co^IV^(13-TMC)(O)]^2+^	3/2	625	1.72	770 (736)	6.5	6.5	2.02	^513^
[Co^III^(PhB(*^t^* BuIm)_3_)(O)]	0		1.682(6)	815 (782)				^519^
[Co^III^(PhB(AdIm)_3_)(O)]	0		1.655(3)	807 (775)				^526^
[Co^IV^(N4Py)(O)]^2+^	1/2			659 (648) and 637 (623)				^514^
[Co^IV^{H(12-TBC)}(O)]^3+^	3/2	720	1.71	782 (745)	5.9	3.4	2.00	^515^

a *S* = 1 Co­(III) center antiferromagnetically coupled to *S* = 1/2 ligand radical.

### 4.2 Spectroscopic Properties of Cobalt–Oxo Complexes

Early strides toward the isolation and characterization of terminal cobalt–oxo intermediates were taken in 2011 by Ray and co-workers, who reported the synthesis of the complex [Co^IV^(TMG_3_tren)­(O)­Sc­(OTf)_3_]^2+^ with enforced TBP symmetry (see Scheme 14).^504^ In addition to the steric stabilization imparted by the TMG_3_tren ligand, the Co­(IV)–oxo group in this compound is stabilized by the association of a Lewis acidic (LA) Sc­(OTf)_3_ group. LA groups like Sc­(OTf)_3_ can bind either to the oxo group or the coligand, but in this case a structural assignment involving a [Co­(IV)–O–Sc­(III)] core was made on the basis of XAS, EXAFS, EPR spectroscopy, and DFT calculations. The complex was additionally characterized by UV–vis absorption spectroscopy and ESI-MS. EPR spectroscopy and DFT calculations both support the formation of an *S* = 3/2 Co­(IV) center, and the Co–O bond length was found at 1.85 Å based on EXAFS data. The long Co–O bond length is attributed to strong binding of the Sc­(III) ion, which largely eliminates any potential Co–O multiple bonding character. However, the Ray group’s Co­(IV)–oxo assignment was challenged in 2012 when Borovik and co-workers reported a Co­(III)−hydroxo species stabilized by a Ca­(II) ion, [Co^III^(MST)­(OH)-(Ca⊂15-crown-5)]^+^ (MST^3–^ = *N*,*N*′,*N*″-[2,2′,2″-nitrilotris­(ethane-2,1-diyl)]­tris­(2,4,6-trimethylbenzenesulfonamido)), which has similar spectroscopic characteristics to [Co^IV^(TMG_3_tren)­(O)­Sc­(OTf)_3_]^2+^.^505^ In particular, the Co–O bond length in the [Co­(III)–OH–Ca­(II)] core based on X-ray crystallography is 1.854 Å, nearly identical to that of the TMG_3_tren complex. Thus, a new proposal for a [Co­(III)–OH–Sc­(III)] structural assignment was made. While the *S* = 3/2 EPR signal in the 2011 study does not correspond to a Co­(III) complex, it has been argued that this signal could instead arise from the presence of some [Co^II^(TMG_3_tren)­(OTf)]­(OTf) precursor remaining in solution. After several years in which no definitive structural assignment could be made, a 2021 study by Ray, Nam and co-workers provided more evidence for the [Co­(IV)–O–Sc­(III)] formulation.^506^ In this study, [Co^II^(TMG_3_tren)­(OTf)]­(OTf) was shown to activate dioxygen in the presence of an H-atom donor and Sc­(OTf)_3_, forming [Co^IV^(TMG_3_tren)­(O)­Sc­(OTf)_3_]^2+^ in >90% yield via a Co­(II)-alkylperoxide intermediate. The EPR spectrum of this complex shows an *S* = 3/2 rhombic signal with *g*_eff,⊥_ = 5.80 and *g*_eff,∥_ = 2.58, which is notably distinct from the *g*_eff,⊥_ ≈ 4.3 and *g*_eff,∥_ ≈ 2.09 signals of all *S* = 3/2 [Co^II^(TMG_3_tren)]^2+^ compounds. This rhombic signal accounts for 94% of the total cobalt-spin in the solution, making the presence of any Co­(III) species unlikely and lending credence to the [Co­(IV)–O–Sc­(III)] structural assignment. It should, however, be noted that both these EPR parameters and the characteristic UV–vis features for the oxo complex differ significantly from the values provided in the original 2011 report,^504^ casting some questions on the ultimate assignment of this complex as a true Co­(IV)–oxo species. From a reactivity standpoint, both [Co^IV^(TMG_3_tren)­(O)­Sc­(OTf)_3_]^2+^ and [Co^III^(MST)­(OH)-(Ca⊂15-crown-5)]^+^ show reactivity toward HAT, but only the TMG_3_tren complex shows reactivity as an OAT agent or one-electron oxidant.

Another early attempt at characterization of a high-valent cobalt intermediate comes from Tilley and co-workers in 2014. They reported the cleavage of dioxygen by a Co­(II) complex to form a potential Co­(IV)–oxo intermediate, [Co^IV^(^Br^HBA-Et)­(O)]^2–^, which can activate C–H bonds (see Scheme 14).^507^ The complex was characterized by UV–vis spectroscopy and ESI-MS. EPR data show the formation of an η^1^−superoxo intermediate which may undergo O–O bond cleavage to form the proposed Co­(IV)–oxo species. ESI-MS and reactivity data, including a relatively low KIE for C–H activation, suggest that the proposed Co­(IV)–oxo compound is the active catalytic species in HAA reactions. Interestingly, C–H bond activation by this complex seems to trend with the p*K*_*a*_ value of the substrate, not the C–H BDFE.

In 2014, Ray, Nam and co-workers reported the stabilization of a putative Co­(IV)–oxo complex, [Co^IV^(TAML^4–^)­(O)]^2–^, which utilizes the TAML^4–^ tetraamido macrocyclic ligand framework (see above) and is further stabilized by a Lewis acid (LA = Sc­(III), Ce­(III), Y­(III), Zn­(II); see Scheme 14).^508^ This complex was characterized by UV–vis and EPR spectroscopy, CSI-MS, XAS, EXAFS, and DFT calculations. Initial DFT studies suggested that the complex tautomerizes from a [Co^III^(TAML^4–^)­(O^•^)]^2–^ core to a [Co^IV^(TAML^4–^)­(O)­(LA)]^+^ core upon LA coordination. EPR spectroscopy and XAS support the existence of an *S* = 1/2 Co­(IV)–oxo center in the LA bound complex. The Co–O bond length was measured at 1.67 Å using EXAFS. Kinetic studies at 298 K showed that the complex is an active participant in HAA from weak C–H bonds and an OAT reagent. Subsequent computational studies by Zhao, Nam, Wang and co-workers provided further evidence that an LA ion is likely necessary for tautomerization from a Co­(III)–oxyl to a Co­(IV)–oxo in this system.^509^ However, a precise assignment of the oxidation state of the Co–oxo unit in this complex with the TAML^4–^ coligand was found to be difficult. As Collins and co-workers have previously shown,^471,510^ TAML^4–^ is redox noninnocent and [Co^IV^(TAML^4–^)] complexes are often better described as [Co^III^(TAML^3–•^)]. This complication comes in addition to the near ubiquitous uncertainty brought about by the potential for Co­(IV)–oxo/Co­(III)–oxyl valence tautomerism^503^ and brings a definitive [Co^IV^(TAML^4–^)­(O)]^2–^ assignment for the complex into question. Based on this uncertainty, as well as EPR inconsistencies in the 2014 study, a more thorough spectroscopic investigation was undertaken by Malik et al. and reported in 2024.^252^ This deeper exploration led to the realization that the 2014 study had conflated two distinct “[Co^IV^(TAML^4–^)­(O)]^2–^” species which exist in a temperature-dependent equilibrium. At high temperatures (253 K), the complex is blue in color and is best described as a Co­(III)−hydroxo species with an LA attached to the hydroxo group and a solvent ligand bound trans to the hydroxo ligand of the cobalt center, [(Sol)­Co^III^(TAML^3–•^)­(OH-LA)]^−^ (see Scheme 15a). The authors note that the four-coordinate square-planar structure could not be definitively ruled out as the identity of the high temperature oxidized [Co­(TAML)] complex. At low temperatures (193 K), the complex is green in color and exists as a mixture of two resonance forms: [Co^IV^(H-TAML^3–^)­(O-LA)]^−^ and [Co^III^(H-TAML^2–•^)­(O-LA)]^−^ (Scheme 15b). Only the blue species was observed in the original 2014 report. Both forms of the complex underwent thorough characterization using a range of spectroscopic techniques, including UV–vis, rRaman, MCD, and regular and pulse EPR spectroscopy, CSI-MS, XAS, EXAFS, and DFT calculations. The two forms of the complex were both assigned an *S* = 1/2 spin state, with the unusual spin state of the high temperature Co­(III) complex arising from antiferromagnetic coupling of the *S* = 1 Co­(III) center to the *S* = 1/2 ligand radical. While no Co–O stretch could be identified in the rRaman spectrum of either form of the complex, EXAFS data suggest a Co–O bond length of ∼2.3 Å in the high temperature complex which contracts significantly to 1.68 Å (when LA = Y­(OTf)_3_) or 1.72 Å (when LA = Sc­(OTf)_3_) in the low temperature form, suggesting that the lower temperature complex has greater Co–O double bond character. Kinetic studies of the complexes at 233 K (where both forms exist in equilibrium) suggest that the high temperature species is significantly more reactive for both OAT reactions and HAA involving the activation of weak C–H bonds.

**Scheme 15:**
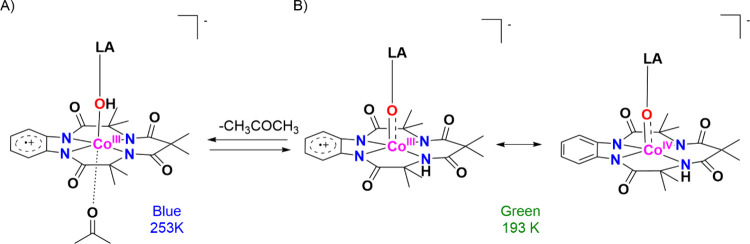
Different Forms of Cobalt–Oxo Complexes with the TAML^4–^ Co-ligand^a^

a (A) Blue, high temperature (253 K) species. (B) Green, low temperature (193 K) species.

It should also be noted that “[Co^IV^(TAML^4–^)­(O)]^2–^” variants have not only shown reactivity in purely chemical oxidations but have also been proposed as intermediates in electrochemical oxidation processes. Sen Gupta and co-workers postulated Co­(III)–oxyl/Co­(IV)–oxo and Co­(IV)–oxyl/Co­(V)–oxo intermediates in the electrochemical oxidation of water by biuret-modified [Co^III^(bTAML^4–^)]^−^ (see Scheme 14) in the presence of the LA ZnCl_2_, based on evidence from UV–vis spectroscopy, high resolution mass spectrometry (HR-MS), and cyclic voltammetry studies.^511^ Additionally, Kim et al. proposed a [Co^III^(TAML^4–^)­(O^•^)]^2–^ intermediate (which can be described as a formal Co^IV^–oxo) in the electrochemical epoxidation of cyclic and acyclic olefins by [Co^III^(TAML^4–^)]^−^ based on *operando* EPR spectroscopy and voltammetry-electrospray ionization mass spectrometry (VESI-MS).^512^ Notably, this reaction proceeds without the addition of a Lewis acidic metal ion.

Unlike many of its predecessors, [Co^IV^(13-TMC)­(O)]^2+^ (see Scheme 14), reported in 2017 by Wang et al, was synthesized without the need for a LA group to stabilize the Co–O moiety.^513^ In fact, [Co^IV^(13-TMC)­(O)]^2+^ can be synthesized by both photochemical and purely chemical oxidation of its Co­(II) precursor. Improved complex purity and stability was observed when synthesized in the presence of an acid, suggesting that ligand protonation may play a role in stabilizing the Co­(IV)–O center. Once stable complex formation was achieved, [Co^IV^(13-TMC)­(O)]^2+^ was characterized via UV–vis, rRaman and EPR spectroscopy, CSI-MS, XAS, EXAFS, and DFT calculations. The cobalt center was assigned as an *S* = 3/2 Co­(IV)–oxo based on EPR and XAS data. An isotope-sensitive Co–O stretch of 770 cm^–1^ was observed via rRaman (see Figure 42), and EXAFS gave a Co–O bond length of 1.72 Å. Notably, the Fe­(IV)–oxo analogue of this complex has both a higher energy M–O stretching frequency and a shorter M–O bond length, suggesting that [Co^IV^(13-TMC)­(O)]^2+^ may have a lower M–O bond order than its iron counterpart, in agreement with the oxo wall concept (see section 2.2). Based on theoretical calculations, the complex does still possess limited multiple bonding character, which was attributed to the existence of a particularly low energy d_*x*2–*y*2_ orbital on the Co center. This lowered d_x2‑y2_ orbital energy results in an electron configuration of [d_*xy*_]^2^[d_*xz*_,d_*yz*_]^2^[d_*x*2–*y*2_]^1^[d_*z*2_]^0^, where the Co–(13-TMC) σ-antibonding d_*x*2–*y*2_ orbital is populated with one electron, instead of doubly occupying the Co–O π-antibonding d_*xz*_ or d_*yz*_ orbital. In terms of reactivity, [Co^IV^(13-TMC)­(O)]^2+^ is a competent oxidant in OAT reactions, C–H bond activation reactions, olefin epoxidation reactions, and the production of terminal Fe­(IV)–oxo complexes.

**Figure 42:**
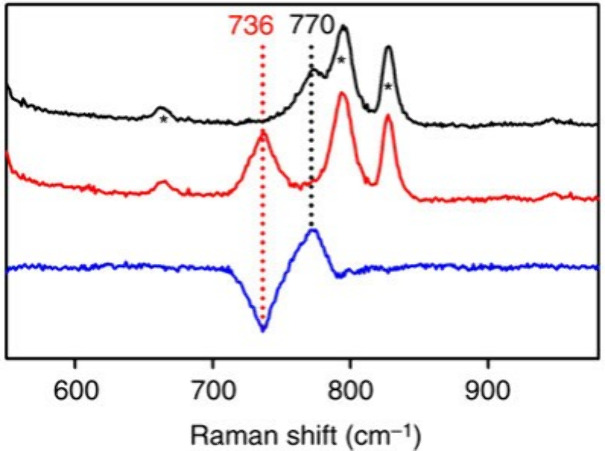
Low-temperature rRaman spectra of [Co^IV^(13-TMC)­(^16^O)]^2+^ (black) and [Co^IV^(13-TMC)­(^18^O)]^2+^ (red) when excited at 413.1 nm in acetone at −40 °C. The blue line shows the difference between the two spectra and solvent-derived peaks are marked with an asterisk. Reproduced with permission from ref 513 under Creative Commons License CC BY. Copyright 2017 The Authors, Springer Nature.

Srnec, Costas, Roithová and co-workers reported the gas phase generation of [Co^IV^(N4Py)­(O)]^2+^ (see Scheme 14), which shows activity in C–H bond activation.^514^ The complex was generated by the electrochemical oxidation and subsequent electrospray ionization of a Co­(II)-chlorate precursor such that a ClO_2_^•^ radical is eliminated by collisional activation, and the resulting oxo species was characterized by MS and helium-tagging infrared and visible photodissociation spectroscopy (IRPD and visPD). IRPD in conjunction with ^18^O labeling studies identified weak Co–O stretching modes at 659 cm^–1^ and 637 cm^–1^. Both modes are coupled with a ligand bending mode involving the nitrogen *trans* to the oxo group and indicate a weak Co–O bond. Spectroscopic data and theoretical calculations were used to assign a spin state of *S* = 1/2 and an electronic configuration of [d_*xy*_]^2^[d_*xz*_/O­(p_*x*_),d_*yz*_/O­(p_*y*_)]^3^[d_z2_]^0^[d_x2–y2_]^0^ to the intermediate with bond inversion of the d_*xz*_–O­(p_*x*_) and d_*yz*_–O­(p_*y*_) interactions such that the putative Co­(IV)–oxo group is best described as a Co­(III)–oxyl center. The complex activates relatively strong C–H bonds in the gas phase, including those of cyclohexane. Data for the corresponding gas phase Co­(III)–oxo species suggest a stronger Co–O bond and no reactivity toward C–H activation.

Most recently, in 2021, Shearer, Lehnert, Nam and co-workers reported "[Co^IV^(12-TBC)­(O)]^2+^" (see Scheme 14), which has a similar ligand environment to the previously characterized complex [Co^IV^(13-TMC)­(O)]^2+^.^515^ Like [Co^IV^(13-TMC)­(O)]^2+^, [Co^IV^{H­(12-TBC)}­(O)]^3+^ was generated in the presence of an acid, which likely protonates one of the ligand amino groups, as indicated in the formula. This complex was characterized by UV–vis, rRaman, EPR and MCD spectroscopy, CSI-MS, XAS, EXAFS, and DFT calculations. EXAFS gave a Co–O bond length of 1.71 Å (see Figure 43), rRaman data show the Co–O stretch at 782 cm^–1^, and EPR and MCD (see Figure 41) data are consistent with a spin state of *S* = 3/2 for the complex. However, EPR and MCD data also suggest that the complex has a rhombic symmetry, with *E*/*D* ∼ 0.17, which is not consistent with the expected symmetry of a tetragonal metal–oxo complex (where *E*/*D* would be around zero). This inconsistency prompted a more detailed study of the EXAFS and XANES regions of the cobalt K-edge X-ray absorption spectrum. The XANES region displays a weak feature approximately 5 eV higher in energy than the Co­(1s → 3d) transition, which was not found in the XANES of square-pyramidal [Co^IV^(13-TMC)­(O)]^2+^. TD-DFT calculations of the pre-edge region identified this feature as a low-energy “MLCT” from the Co­(1s) into a predominantly oxygen-based orbital with significant Co­(4p) character. This feature becomes significantly lower in energy for a “see-saw” structure, where the protonated amino group is no longer bound to the cobalt center and the oxo ligand hydrogen bonds with the protonated amine (see Scheme 16), relative to the square-pyramidal structure. Calculation of *g*-values and zero-field splitting parameters for this structure were also consistent with EPR and MCD derived values. Additionally, theoretical calculations show differences in the degree of ligand field inversion experienced by the π-bonding d_*xz*_–O­(p_*x*_) and d_*yz*_–O­(p_*y*_) interactions. The singly occupied π* orbital of the d_*xz*_–O­(p_*x*_) bond exhibits a metal-centered character more typical for metal–oxo antibonding molecular orbitals, with 59% Co and 35% O contributions. In contrast, the SOMO, which is the π* orbital of the d_*yz*_–O­(p_*y*_) bond shows an inverted interaction, with 40% Co and 56% O character. This interesting difference in the degree of ligand field inversion is attributed to the presence of the hydrogen bond between the oxo group and the protonated amino group of the ligand, which stabilizes the O­(p_*x*_) orbital and in this way, keeps it lower in energy than the cobalt d_*xz*_ orbital, preventing bond inversion. In terms of reactivity, the complex is active for the activation of weak C–H bonds via HAT, as well as for OAT reactions.

**Figure 43:**
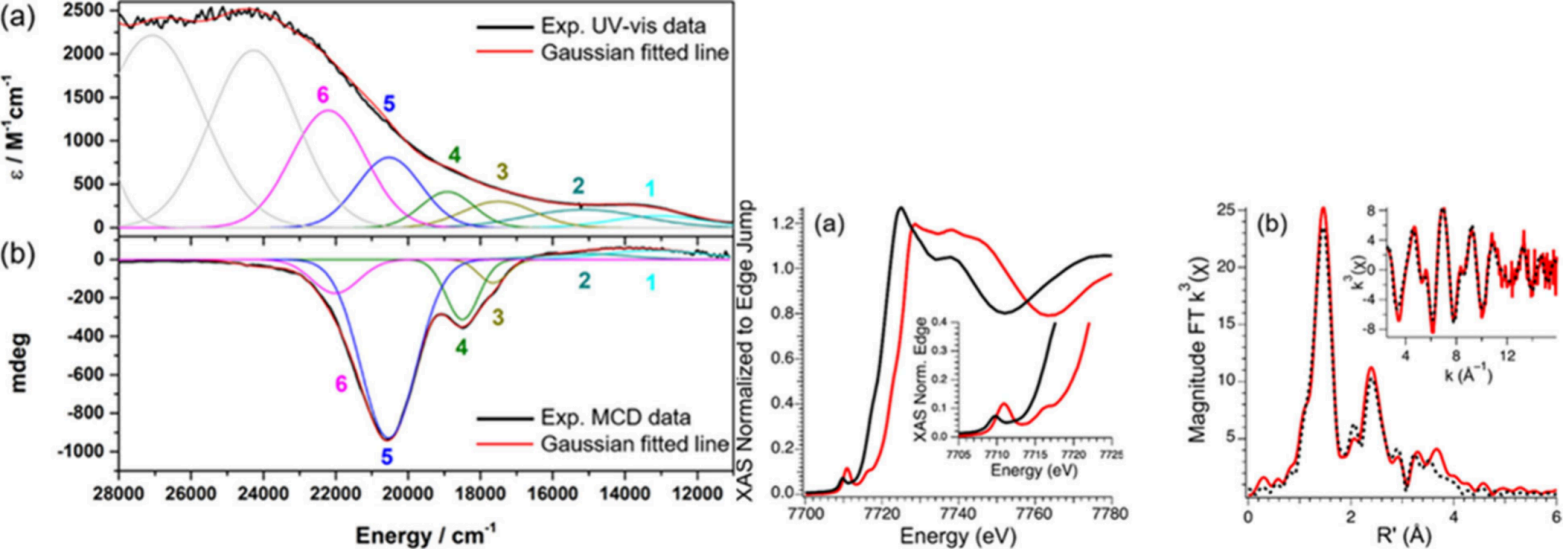
Left: (a) UV–vis absorption spectrum of 1.0 mM [Co^IV^{H­(12-TBC)}­(O)]^3+^ at −40 °C. (b) MCD data of 4.0 mM [Co^IV^{H­(12-TBC)}­(O)]^3+^ at 2 K and 7 T. Right: (a) XANES region of the cobalt K-edge X-ray absorption spectrum of [Co^II^(12-TBC)­(CF_3_SO_3_)_2_] (black) and [Co^IV^{H­(12-TBC)}­(O)]^3+^ (red). (b) EXAFS region of the cobalt K-edge X-ray absorption spectrum of [Co^IV^{H­(12-TBC)}­(O)]^3+^ with experimental data (red) and best fit (black). Reproduced with permission from ref 515. Copyright 2021 American Chemical Society.

**Scheme 16:**
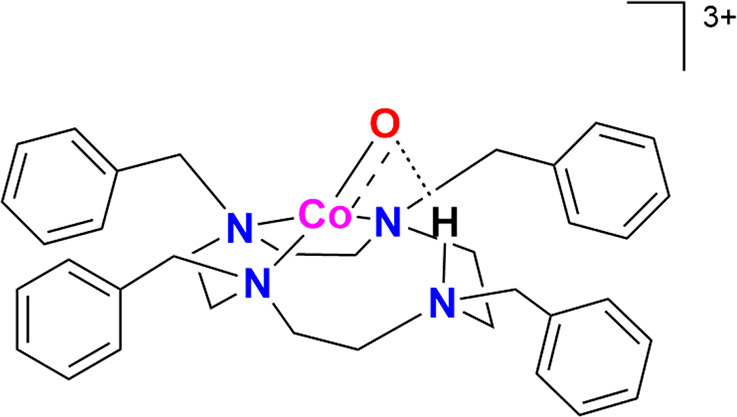
Proposed Structure of the Distorted Complex [Co^IV^{H­(12-TBC)}­(O)]^3+^, Induced by Ligand Protonation

Unless otherwise noted, the high-valent complexes in this section were synthesized via oxidation of a Co­(II) precursor by an iodosylarene, usually PhIO. Some caution must be taken with this method of synthesis, as iodosylarenes are capable of promoting similar reactivity to that expected of metal–oxo complexes, and their decay does not necessarily create the corresponding metal–oxo intermediates.^253^ Illustratively, several Co­(II)– and Co­(III)–iodosylarene adducts with reactivity in line with their cobalt–oxo counterparts have been reported in recent years and are briefly discussed here. Anderson and co-workers reported a series of Co­(II)–iodosylarene and –iodoxyarene complexes, [Co^II^(Tp^Ad,Me^)­(^*s*^PhIO)]^+^, [Co^II^(Tp^*t*Bu^)­(^*s*^PhIO)]^+^ and [Co^II^(Tp^*t*Bu^)­(^*s*^PhIO_2_)]^+^ (Tp^–^ = hydrotris­(pyrazolyl)­borate anion), in 2018, which were characterized by NMR, UV–vis and EPR spectroscopy, and X-ray crystallography (see Figure 44a).^516^ [Co^II^(Tp^Ad,Me^)­(^*s*^PhIO)]^+^ acts only as an OAT agent for phosphines and may form a cobalt–oxo intermediate, while the other two complexes show a broader substrate range for OAT and sluggish HAA reactivity, seeming to react as iodosylarene adducts. In 2020, Fukuzumi, Shearer, Nam and co-workers reported another iodosylarene adduct, [Co^III^(TQA)­(OIPh)­(OH)]^2+^ (see Figure 44b),^517^ characterized by UV–vis, rRaman, EPR and NMR spectroscopy as well as CSI-MS, XAS, EXAFS, and X-ray crystallography. This complex shows a uniquely amphoteric reactivity, acting as an *electrophile* in olefin epoxidation and other OAT reactions, and acting as a *nucleophile* in aldehyde deformylation reactions.

**Figure 44:**
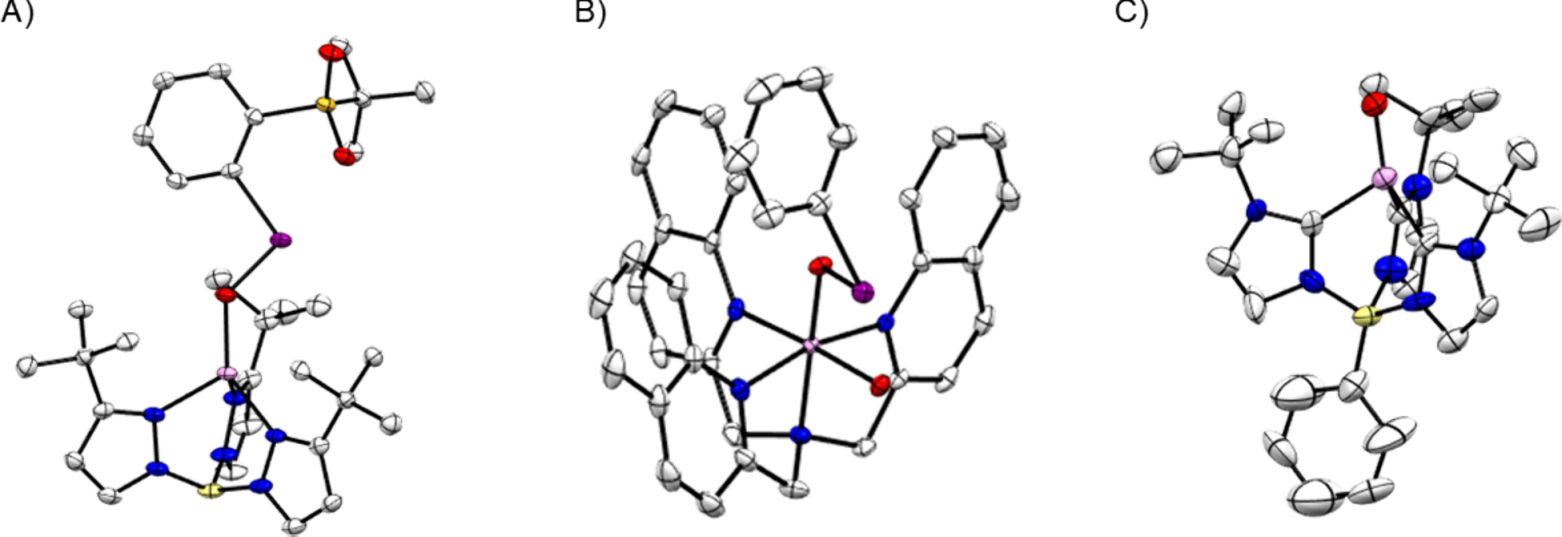
Representative molecular structures of (A) Co­(II)-iodosylbenzene^518^ (B) Co­(III)-iodosylbenzene^517^ and (C) Co­(III)–oxo^519^ complexes. Thermal ellipsoids are set at the 50% probability level and hydrogen atoms are omitted for clarity.

Anderson and co-workers took a different approach to synthesize high-valent cobalt–oxo complexes, taking advantage of strongly donating carbene coligands and an enforced PTD geometry to synthesize [Co^III^(PhB­(*^t^* BuIm)_3_)­(O)] (see Scheme 14 and Figure 44c) via a multistep oxidation and deprotonation process.^519^ Despite having six cobalt d-electrons, this complex is stable enough at temperatures < −35 °C to be isolated as single crystals and characterized via X-ray crystallography, giving a Co–O bond length of 1.682(6) Å. The compound was additionally characterized by ^1^H-, ^13^C-, and ^11^B-NMR, IR and UV–vis spectroscopy, and DFT calculations. In particular, IR spectroscopy shows the Co–O stretch at 815 cm^–1^. The complex shows reactivity in the activation of weak C–H bonds and in OAT. Additionally, Anderson and co-workers synthesized a variant of this complex with a more donating adamantane-substituted ligand, [Co^III^(PhB­(AdIm)_3_)­(O)], reported in 2021.^519^ The Co–O bond length of this complex is 1.655(3) Å by single crystal X-ray crystallography, the Co–O stretch from IR spectroscopy is observed at 807 cm^–1^, and the complex was again further characterized by UV–vis, ^1^H-, ^13^C- and ^11^B-NMR spectroscopy, and cyclic voltammetry. Interestingly, this complex can be further oxidized to form a transient Co­(IV)–oxo intermediate which rapidly (estimated *k*_1_ > 1.3(7) × 10^2^ s^–1^) abstracts an H-atom from a ligand adamantane group to form the Co­(III)–O-C­(Ad) product. This Co­(IV)–oxo intermediate can be observed on the EPR time scale when all ligand adamantane groups are fully deuterated, and was further investigated using DFT calculations. The two Co­(III)–oxo complexes from Anderson’s group remain the only crystallographically characterized high-valent cobalt–oxo compounds to date.

After preliminary results suggested that C–H bond activation by [Co^III^(PhB­(*^t^* BuIm)_3_)­(O)] and [Co^III^(PhB­(AdIm)_3_)­(O)] trends with p*K*_a_ rather than with C–H bond strength (BDFE), as would generally be expected for C–H bond activation reactions of this type, a series of mechanistic studies were performed by Anderson and co-workers on these complexes. Computational and kinetic evidence from these studies suggests that these Co­(III) complexes undergo a basicity-controlled asynchronous PCET mechanism for C–H bond activation. As detailed in Figure 45a, conventional discussion of net PCET processes tends to assign either a one-step concerted proton–electron transfer (CPET) mechanism or one of two stepwise mechanisms: proton transfer-electron transfer (PT/ET) or electron transfer-proton transfer (ET/PT). More recently, evidence has emerged that in addition to these mechanisms, a CPET process can have some degree of “asynchronicity”, characterized by a transition state with greater proton transfer (basic asynchronous PCET) or electron transfer (oxidative asynchronous PCET) character (see section 2.4.2.3), and thus, a dependence of reaction rate on substrate acidity or oxidation potential, rather than C–H BDFE, is then expected.^521^ The basic asynchronous PCET mechanism arises from changes to the free energy of the PT transition state due to substrate acidity and/or metal–oxo basicity. Significant electronic coupling between the reactant and PT electronic states (see Figure 45b) results in an anharmonic reactant energy well (see Figure 45c). Increasing the acidity of substrate C–H bonds results in an increase in this anharmonicity, allowing for a more easily accessible transition state, which is still concerted but is dominated by proton transfer character (see Figure 45d). The lower transition state energy results in faster PCET reaction rates. The more asynchronous the mechanism (or the closer the transition state is to a pure PT transition state), the faster the reaction occurs, up to the point at which a true stepwise PT/ET mechanism becomes more thermodynamically favorable. Once a reaction “crosses over” from an asynchronous CPET to a PT/ET mechanism, it slows down due to the presence of an intermediate state. Based on these findings, it can be concluded that HAA by Anderson’s Co­(III)–oxo complexes is dominated by the basicity of the oxo group, whereas for Co­(IV) complexes, a much higher oxidative potential can be expected, with less or no dependence of the HAA reaction rate on the substrate p*K*_a_.

**Figure 45:**
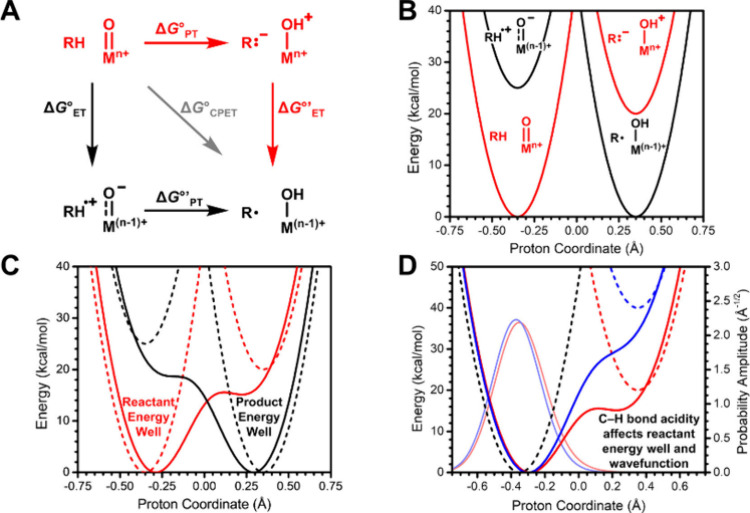
Pathways for net PCET reactions and perturbations induced by C–H bond acidity. (A) Square scheme for net PCET pathways. (B) Energies of each corner of the square scheme as a function of proton position. (C) Coupling between the reactants/PT electronic states (red) and the products/ET electronic states (black). (D) Changes in the energy of the PT electronic state due to substrate C–H bond acidity. Reproduced with permission from ref 520. Copyright 2023 American Chemical Society.

Most recently, several Co­(IV) complexes with bridging oxo groups have been identified and characterized. In 2019, Talipov, Li, Wang and co-workers reported a “diamond core” Co­(III)/Co­(IV) bis­(μ-oxo) species^523^ with supporting tetradentate TPA coligands (see Figure 46b), which they characterized via UV–vis and EPR spectroscopy, XAS, and EXAFS. The complex was further characterized by Que, Wang and co-workers using rRaman and EXAFS in 2023, allowing for the identification of a related (Co^III^)_2_(μ-O)­(μ-OH) intermediate.^525^ Notably, the Co­(III)/Co­(IV) bis­(μ-oxo) complex shows a four- to five-fold increase in reactivity toward C–H bond activation when compared to the analogous Fe­(III)/Fe­(IV) bis­(μ-oxo) complex. Anderson and co-workers reported a Co­(IV)_2_ bis­(μ-oxo) diamond core species,^522^ [(Co^IV^)_2_(HB-(^CF3,Ph^mIm)_3_)_2_(μ-O)_2_]^2+^ (HB-(^CF3,Ph^mIm)_3_ = tris­(4-trifluoromethyl-3-methyl-2-phenylimidazol-5-ylidene)­borate, see Figure 46a). The complex was synthesized from the corresponding Co­(II)–chloride and Co­(III)–superoxo monomers and was characterized by UV–vis, NMR, IR and EPR spectroscopy, XAS, EXAFS, DFT calculations, and X-ray crystallography. This complex is very stable at low temperature and shows no interesting reactivity. Lastly, in 2025 a Co­(IV) dimer with only one bridging oxo group and the supporting coligand *N,N’*-(cyclohexane-1,2-diyl)­bis­(2-hydroxy-2-methylpropanamide) was reported by Moonshiram, Paria and co-workers (Figure 46c), and characterized by UV–vis, rRaman and NMR spectroscopy, XAS, and EXAFS.^524^ This complex shows reactivity in HAA and OAT reactions.

**Figure 46:**
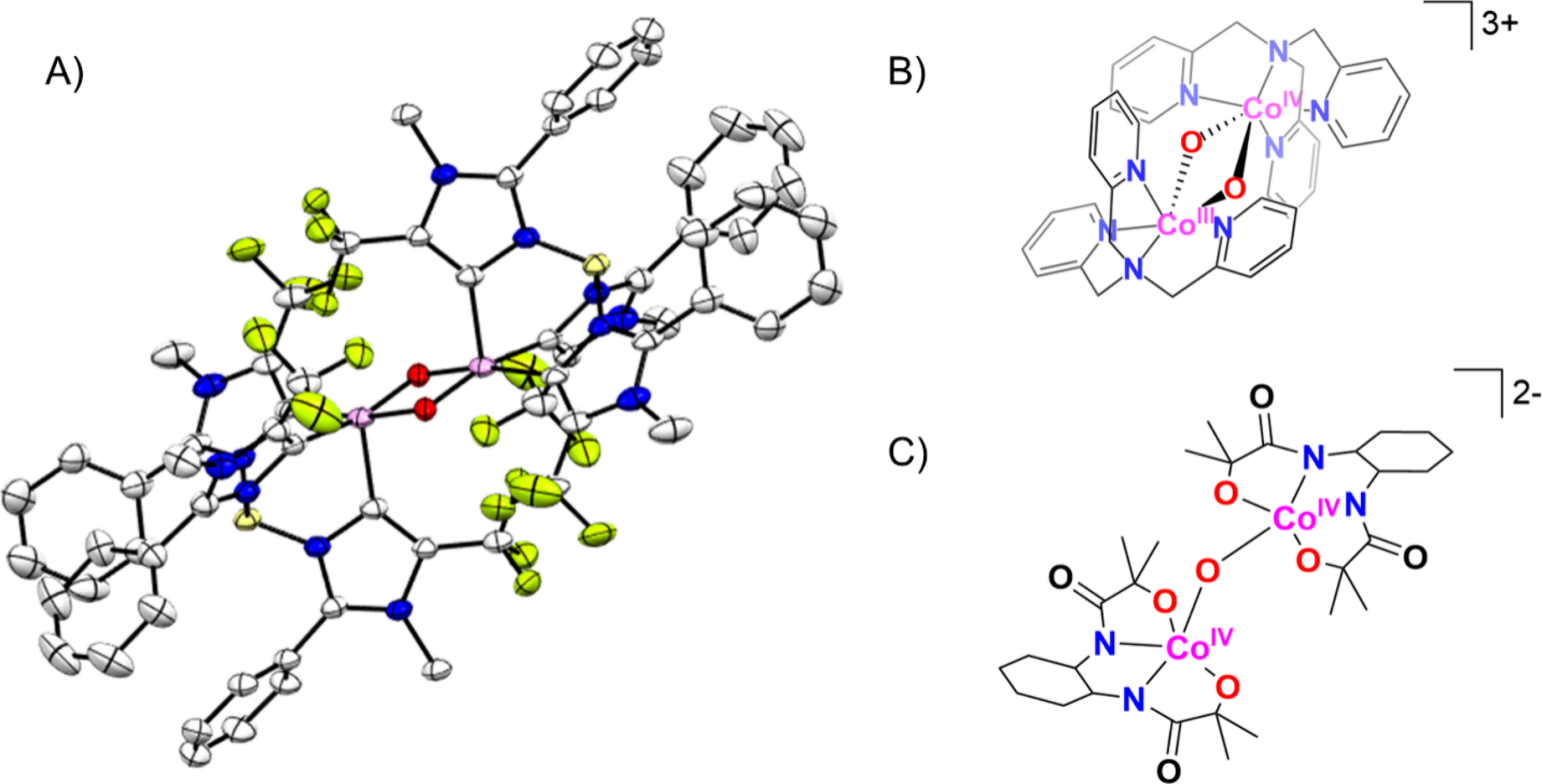
(A) Molecular structure of the Co­(IV)/Co­(IV) bis­(μ-oxo) diamond core dimer.^522^ Hydrogen atoms and outer sphere ions are omitted for clarity. Proposed structures of (B) Co­(III)/Co­(IV) diamond core^523^ and (C) a Co­(IV)/Co­(IV) dimer with a singly bridging oxo ligand.^524^

### 4.3 Reactivity of Cobalt–Oxo Complexes

Because so few of these cobalt–oxo complexes have been characterized, no formal comparisons of reactivity across different ligand frameworks have yet been made to our knowledge. There is yet to be a consensus on the type of substrates that should be used to explore the reactivity of these complexes, and reactions were monitored under varying conditions and temperatures. In an effort to alleviate this gap, we have compiled the reaction rates of a number of cobalt–oxo species across the most common C–H activation substrates in Table 9. However, because many of these complexes are stabilized by LA groups, solvent molecules, or protonation to a hydroxide (rather than being true terminal Co–oxo groups), it must be emphasized that this table constitutes only a rough comparison which may not always reflect the reactivity of true terminal cobalt–oxo complexes.

**Table 9:** Second-Order Rate Constants for HAA Reactions of Co–oxo Complexes

complex	temperature (K)	*k*_2_ (xanthene) (M^–1^s^–1^)	*k*_2_ (DHA) (M^–1^s^–1^)	*k*_2_ (CHD) (M^–1^s^–1^)	*k*_2_ (thioanisole) (M^–1^s^–1^)	ref
[Co^IV^(TMG_3_tren)(O)Sc(OTf_3_)]^2+^	273	7.1 × 10^–1^	2.9 × 10^–1^	7.5 × 10^–2^		^506^

[Co^III^(TAML^3–•^)(OH-LA)]^−^	278	2.3 × 10^–1^	2.1 × 10^–1^	1.2 × 10^–1^	1.5	^252,508^
				1.9 × 10^–1^ (233 K)	2.2 (233 K)	

[Co^IV^(H-TAML^3–^)(O-LA)]^−^	233			4.3 × 10^–3^	9.7 × 10^–2^	^252^
[Co^IV^(13-TMC)(O)]^2+^	233	1.5 × 10^–1^	8.3 × 10^–2^	3.7 × 10^–2^		^513^
[Co^III^(PhB(*^t^* BuIm)_3_)(O)]	298	1.4 × 10^–3^ s^–1^ (*k*_obs_)	5.85 × 10^–2^	2.7 × 10^–3^		^519,527,528^
[Co^IV^{H(12-TBC)}(O)]^3+^	233	3.2 × 10^–2^		2.0 × 10^–2^	1.9 × 10^–2^	^515^
[Co^III^(TQA)(OIPh)(OH)]^2+^	298	3.6 × 10^–2^	4.9 × 10^–2^	4.5 × 10^–2^		^517^

## 5 Synthetic Terminal Nickel–Oxo Complexes

### 5.1 Structures of Nickel–Oxo Complexes

Direct spectroscopic evidence of high-valent Ni–oxo species is hard to come by. While a variety of putative Ni­(III)–oxo and Ni­(IV)–oxo intermediates have been invoked based on characteristic metal–oxo reactivity patterns, in most cases only the decay products of these species have been spectroscopically observed. Here we present several potential Ni–oxo species with varying degrees of successful characterization (see Scheme 17).

**Scheme 17:**
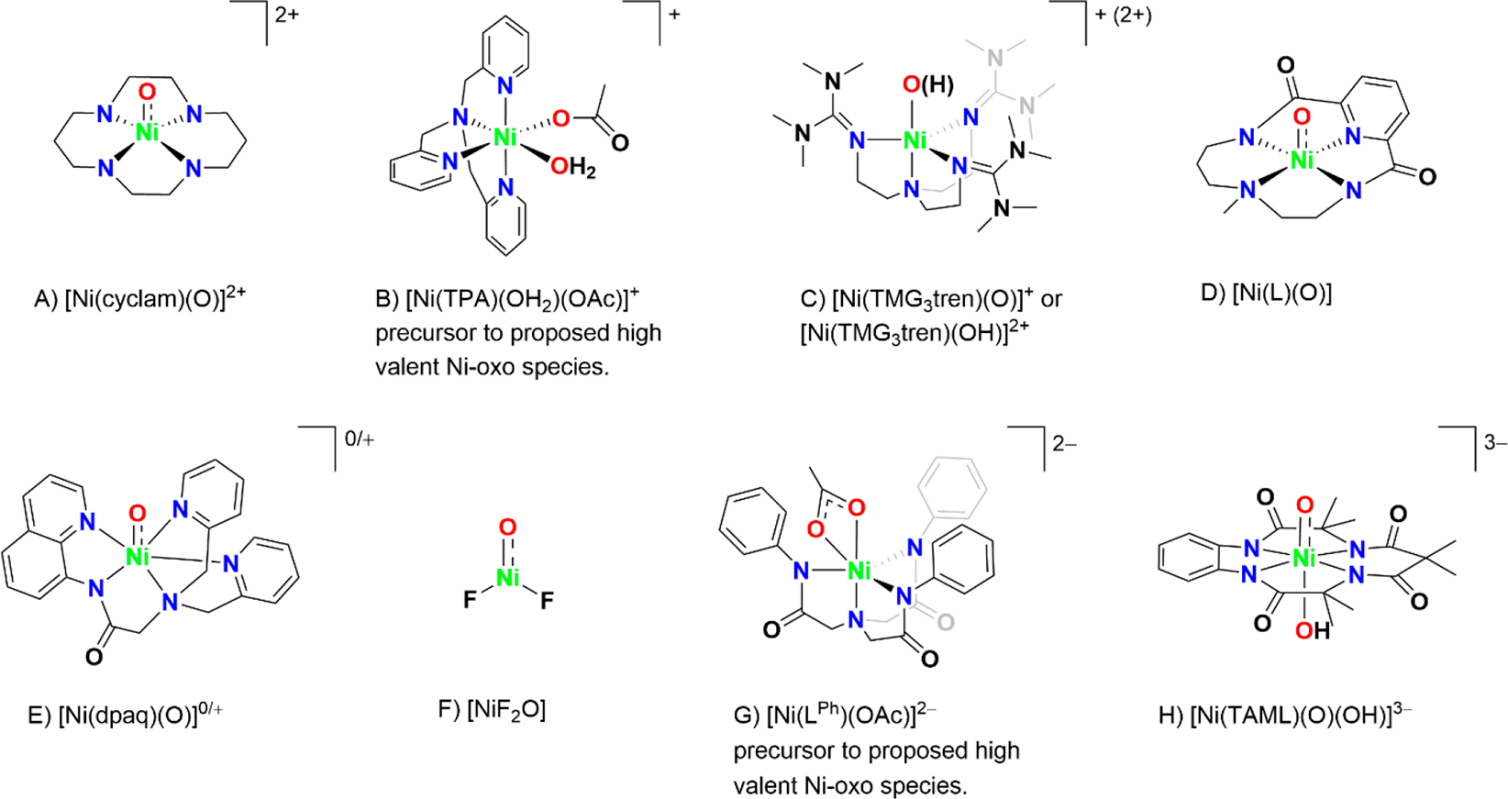
Proposed Structures of High-Valent Nickel–Oxo Complexes^a^

a Ligand nomenclature: L^2–^ = 6-methyl-3,6,9-triaza-1­(2,6)-pyridinacyclodecaphane-2,10-dione; L^Ph^ ^3–^= 2,2′,2′′-nitrilo-tris­(*N*-phenylacetamide).

### 5.2 Spectroscopic Properties of Ni–Oxo Complexes

Early work from Kochi and Burrows in the 1980s established the possibility of a high-valent Ni–oxo intermediate in the epoxidation of alkenes, catalyzed by a Ni­(II) cyclam complex (see Scheme 17).^529,530^ PhIO was confirmed as the oxygen source for the epoxidation process based on ^18^O labeling studies, and a Ni-PhIO adduct or a Ni­(III)–oxyl species were suggested as alternative potential intermediates. However, no intermediate species could be successfully spectroscopically identified, and research involving high-valent Ni–oxo complexes largely stagnated until the early 2000s.

Itoh and co-workers invoked a high-valent nickel–oxo species as a catalytic intermediate in the hydroxylation of alkanes with strong C–H bonds, including cyclohexane, in 2006.^531^ This putative Ni­(III)–oxo species was generated from [Ni^II^(TPA)­(OH_2_)­(OAc)]^+^ (see Scheme 17) using *m*-CPBA, and it could oxidize cyclohexane with TONs of 587 and 69 for the alcohol and ketone products, respectively, giving an alcohol/ketone ratio of 8.5:1. A deuterium KIE of 2.8 was reported when cyclohexane-*d*_*12*_ was used as the substrate. However, no spectroscopic data were provided to support the presence of a high-valent intermediate, and the assignment of the reactive intermediate was simply made on the basis of reactivity trends in the oxidation of cyclohexane by several different M­(TPA) complexes. Trends in catalytic turnover (Mn < Fe < Co < Ni) and alcohol product selectivity (Mn < Fe < Ni < Co) of these complexes matched experimental^532–534^ and theoretical^535^ results for alkane C–H activation by gas phase, high valent [M^III^(O)]^+^ ions. Additionally, Itoh’s group later showed that changes to nickel ligation had some effect on the turnover number and the selectivity of these reactions, supporting the idea that a nickel species is involved in the catalytic mechanism.^536^

While *m*-CPBA has proven to be a useful oxidant in the generation of high-valent metal–oxo complexes, its usage under catalytic conditions should be undertaken with some caution. Recent work has shown that, when activated by a nickel catalyst that enables O–O bond homolysis, *m*-CPBA can enable the oxygenation of unactivated C–H bonds via a free radical chain mechanism (see Scheme 18).^537–539^ Qiu and Hartwig showed that a range of nickel complexes, including both Ni­(TPA) and NiCl_2_, had very similar turnover and selectivity in the oxidation of cyclohexane and adamantane regardless of supporting ligand framework, suggesting that the nickel complex is not the active oxidant in those reactions, at least under multiple turnover conditions.^537^ Further evidence for a mechanism involving free organic radicals with long lifetimes was gathered from the nearly equal fraction of *cis* vs *trans* products in the oxidation of *cis*- or *trans*-1,2-dimethylcyclohexane, which was again consistent across different nickel catalysts. While Itoh’s group later provided some evidence that oxidation by a Ni–oxo species might still occur as a side pathway,^540^ it is clear that the invocation of a high-valent Ni–oxo intermediate when using *m*-CPBA as an oxidant for catalysis does require extensive testing to rule out a free radical pathway.

**Scheme 18:**
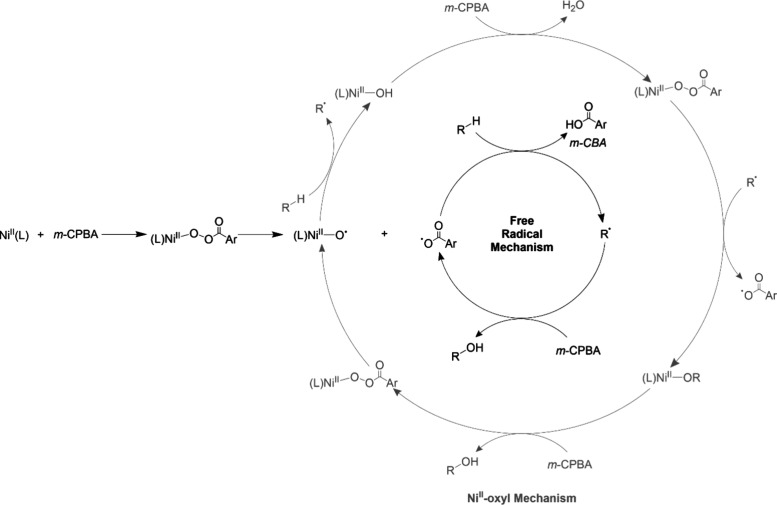
Proposed Catalytic Mechanism of Alkane Oxidation Involving *m*-CPBA and a Nickel Catalyst

Regardless of the actual mechanism of catalytic C–H activation when both *m*-CPBA and a nickel complex are involved, *m*-CPBA has proven a competent oxidant in the generation of high-valent Ni–oxo complexes under noncatalytic conditions. In 2012, the Ray group reported the generation of a potential Ni­(III)–oxyl or hydroxo intermediate, [Ni^III^(TMG_3_tren)­(O)]^+^ or [Ni^III^(TMG_3_tren)­(OH)]^2+^ (see Scheme 17), using *m*-CPBA as the oxidant.^541^ This oxidation occurs via homolysis of the O–O bond in *m*-CPBA, as evidenced by the presence of diagnostic chlorophenyl decay products in the postoxidation solutions and by the formation of a formally Ni­(III) species. Two rhombic S = 1/2 intermediates with half-lives of 1 h at 253 K, presumed to be the Ni­(III)–oxo and Ni­(III)–hydroxo species, appeared in a total of 15% yield upon oxidation of the Ni­(II) precursor complex with one equivalent of *m*-CPBA. They were characterized via UV–vis and EPR spectroscopy, and ESI-MS. By EPR, one species with *g* = 2.05, 2.16, 2.31 accounted for 85% of the Ni­(III) spin, while the other 15% came from a species with *g* = 2.13, 2.17, 2.26. The difference between these species was attributed to the protonation state of the oxo group, but no definitive assignment of which EPR signal belongs to which species could be made. Kinetic studies showed that the oxidized intermediate(s) is/are active for OAT and HAT, and the role of the Ni–oxo/hydroxo group as the active oxidant in these reactions was confirmed by the pseudo-first-order decay of the EPR signals upon substrate addition. In particular, the oxidation of DHA occurs with a second-order rate constant of 1.25 × 10^–3^ M^–1^s^–1^ and shows a deuterium KIE of 3.9 upon use of deuterated substrate.

Company and co-workers reported a high-valent nickel–oxo species supported by a planar bis­(amidate) framework.^542^ Interestingly, the complex [Ni^III^(L)­(O)] (see Scheme 17), likely a formally Ni­(III)–oxyl/Ni­(IV)–oxo intermediate, was formed via heterolysis (rather than homolysis) of the O–O bond in *m*-CPBA. Characterization of the intermediate was carried out via UV–vis, rRaman and EPR spectroscopy, as well as CSI-MS, XANES/EXAFS, and DFT calculations. Two *S* = 1/2 species were identified in 16% yield by EPR spectroscopy, but further experimentation suggested that these species were actually decay products formed by comproportionation between an initially formed Ni­(III)–oxyl/Ni­(IV)–oxo intermediate and its [Ni^II^(L)] precursor, resulting in the formation of [Ni^III^(L)]^+^. EXAFS suggested a Ni–O bond length of 2.12 Å, a bond length indicative of a very weak Ni–O interaction, likely corresponding to a reactive nickel–oxygen intermediate, while DFT calculations predicted a Ni–O bond length of 1.95 Å. The assignment of a weak Ni–O bond was additionally supported by rRaman experiments, where low-energy peaks at 450 and 477 cm^–1^ were assigned as Ni–O and a Ni–(coligand) stretching vibration by DFT, respectively, though no isotopic labeling studies were performed to solidify these assignments. The intermediate is active in OAT, olefin epoxidation, and HAA involving O–H bonds and both activated and unactivated C–H bonds. Reaction with deuterated DHA yielded a KIE of 4. In the catalytic oxidation of cyclohexane, [Ni^III^(L)­(O)] reached a TON of 100 and produced alcohol and ketone products in an essentially 1:1 ratio. An 84% retention of configuration in the oxidation of *cis*-1,2-dimethylcyclohexane and a 3°:2° ratio of 18:1 in the oxidation of adamantane strongly suggests that these oxidation processes occur via a nickel-based oxidant, rather than a free radical mechanism involving *m*-CPBA.

Kim and co-workers claimed the formation of both putative Ni­(IV)–oxo and Ni­(III)–oxo intermediates, which are active for olefin epoxidation in the presence of *m*-CPBA.^543^ The reactivity of these [Ni^III/IV^(dpaq)­(O)]^0/+^ species (see Scheme 17) was studied by epoxide product and Hammett analyses. In addition, peroxyphenylacetic acid was used as a mechanistic probe, and the results suggest that an initial nickel-acylperoxo species undergoes approximately 75% O–O bond heterolysis and 25% O–O bond homolysis to form the respective Ni­(IV)– and Ni­(III)–oxo intermediates, prior to olefin epoxidation. EPR results are consistent with a Ni­(III) species and ESI-MS data indicate the presence of a Ni­(IV) species, but no further characterization of these proposed intermediates is provided.

A nickel oxodifluoride species, NiF_2_O (see Scheme 17), was isolated from the gas phase on a solid neon matrix by Riedel and co-workers.^544^ The complex has an ^18^O sensitive Ni–F stretch at 640.3 cm^–1^ by IR spectroscopy. NiF_2_O was assigned as a Ni­(III)–oxyl species based on theoretical modeling. No Ni–O stretch could be identified experimentally, and computational results suggest that any Ni–O stretching band would be very low in intensity.

McDonald and co-workers reported the formation of a ligand-oxidized Ni­(III)–phenolate intermediate upon the oxidation of [Ni^II^(L^Ph^)­(OAc)]^2–^ with *m*-CPBA (see Scheme 17).^545^ The addition of aliphatic hydrocarbon substrates resulted in the ligand oxidation being suppressed and substrate OAT products being observed instead, suggesting that the process goes through a putative Ni­(IV)–oxo intermediate, which, however, could not be directly observed by spectroscopy at temperatures ≥ 233 K.

To date, the best spectroscopically characterized Ni–oxo species is the complex [Ni^III^(TAML^4–^)­(O^•–^)­(OH)]^3–^, reported by Shearer, Lehnert, Nam and co-workers in 2022 (see Scheme 17).^546^ The formally Ni­(IV)–oxo complex was synthesized by the addition of two equivalents of cerium­(IV) ammonium nitrate (CAN) to a nickel­(II) precursor in the presence of water, or by the addition of PhIO to the same precursor in the presence of trifluoromethanesulfonic acid. It has a half-life of 3 h at 233 K and was characterized in detail using UV–vis, EPR and MCD spectroscopy, CSI-MS, XANES/EXAFS, as well as theoretical calculations. While [Ni^III^(TAML^4–^)­(O^•–^)­(OH)]^3–^ is EPR silent, as would be expected for a formally Ni­(IV) species, the non-oxo Ni­(III) analog, which can be generated either by the addition of one equivalent of CAN to the Ni­(II) precursor or the comproportionation of the Ni­(II) precursor and [Ni^III^(TAML^4–^)­(O^•–^)­(OH)]^3–^, is an *S* = 1/2 species with *g* = 2.20, 2.07, 1.99. For [Ni^III^(TAML^4–^)­(O^•–^)­(OH)]^3–^, MCD results (see Figure 47, right) assign a total spin of *S* = 1 to the intermediate, and the analysis of the VTVH MCD data is consistent with a rhombic complex with *D* = 4.0 cm^–1^, *E*/*D* = 0.24, and *g* = 2.013, 2.070, 2.078, distinct from the nonoxo Ni­(III) species. Analysis of the XANES region of the XAS data (see Figure 47, left) shows a K-edge shift consistent with a nickel oxidation state of +3, but also suggests a six-coordinate, centrosymmetric coordination environment about nickel in [Ni^III^(TAML^4–^)­(O^•–^)­(OH)]^3–^. The data from the EXAFS region suggest two nickel oxygen bonds, one with a bond length of 1.84 Å and the other one with a distance of 2.2 Å (see Figure 47, middle). DFT calculations suggest that the shorter bond belongs to the terminal Ni–oxo moiety, while the more distant oxygen corresponds to a hydroxo ligand, with the extended bond length attributed to potential hydrogen bonding with other species in solution. Further DFT studies revealed a significant degree of bond inversion in the Ni–O π* orbitals. While the d_*yz*_–O­(p_*y*_) orbital interaction is inverted, both the corresponding bonding and antibonding molecular orbitals are fully occupied, so this orbital interaction is net nonbonding. On the other hand, in the case of the d_*xz*_–O­(p_*x*_) orbital interaction, the antibonding molecular orbital is singly occupied and has 82% O­(p_*x*_) and only 9% d_*xz*_ contribution. This significant degree of bond inversion implies a relatively weak Ni–O bond with strong oxyl radical character. As such, the net *S* = 1 spin of the complex results from ferromagnetic coupling of the *S* = 1/2 Ni­(III) center and the *S* = 1/2 oxyl radical ligand. Interestingly, TAML^4–^ appears to behave as a redox innocent ligand in this particular case, which is unexpected. In terms of reactivity, the intermediate is active for OAT, olefin epoxidation, and HAA involving activated C–H bonds, with xanthene oxidation showing a deuterium KIE of 7.0(3).

**Figure 47:**
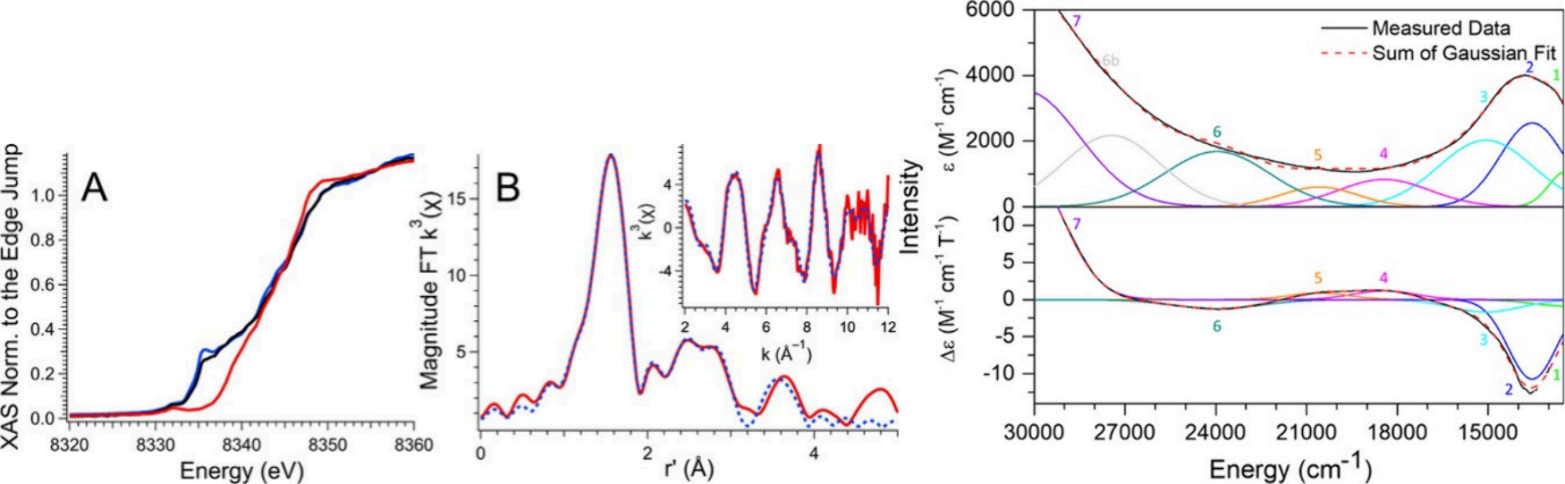
Left: (A) XANES region of the nickel K-edge X-ray absorption spectrum of [Ni^II^(TAML^4–^)]^2–^ (blue), [Ni^III^(TAML^4–^)]^−^ (black), and [Ni^III^(TAML^4–^)­(O^•–^)­(OH)]^3–^ (red). (B) EXAFS region of the nickel K-edge X-ray absorption spectrum of [Ni^III^(TAML^4–^)­(O^•–^)­(OH)]^3–^ with experimental data (red) and best fit (blue). Right: UV–vis data obtained at −40 °C (top) and MCD data obtained at 2 K and 7 T (bottom) for [Ni^III^(TAML^4–^)­(O^•–^)­(OH)]^3–^ and corresponding Gaussian peak fits. Reproduced with permission from ref 546. Copyright 2022, American Chemical Society.

While fully defined Ni­(III)–OH species are largely not addressed in this review, brief mention must be made of very recent work by Hsu and co-workers.^547^ Their crystallographically characterized [Ni­(PS3″)­(OH)]^−^ complex is room temperature stable and exhibits sluggish reactivity toward several common C–H activation substrates at 35 °C, forming a Ni­(II)-aquo product (Figure 48A). The rates of these reactions trend with substrate p*K*_a_, consistent with a basicity-controlled asynchronous PCET mechanism. In addition to crystallographic characterization, the complex underwent UV–vis, XAS, EPR, and NMR spectroscopic studies, and the aquo product was identified via UV–vis, ^1^H- and ^31^P-NMR spectroscopy. Perhaps most interestingly, the authors were able to derive a BDFE of 96.9–100.3 kcal/mol for the newly formed O–H bond in the Ni­(II)-aquo product based on the redox potential and p*K*_a_ of the parent Ni­(III)–OH complex (Figure 48B), adding to the extremely limited existing body of thermodynamic information about high-valent late transition metal complexes, and providing a framework for similar thermodynamic studies on other, related intermediates. Despite the high BDFE of the complex, the authors relate the relatively low reactivity of the intermediate to the proton-dominated asynchronicity of the transition state for HAA. Another factor could be the binding of the LA [K­(18-crown-6)]^+^ to the Ni­(III)–OH unit (see Figure 48A).

**Figure 48:**
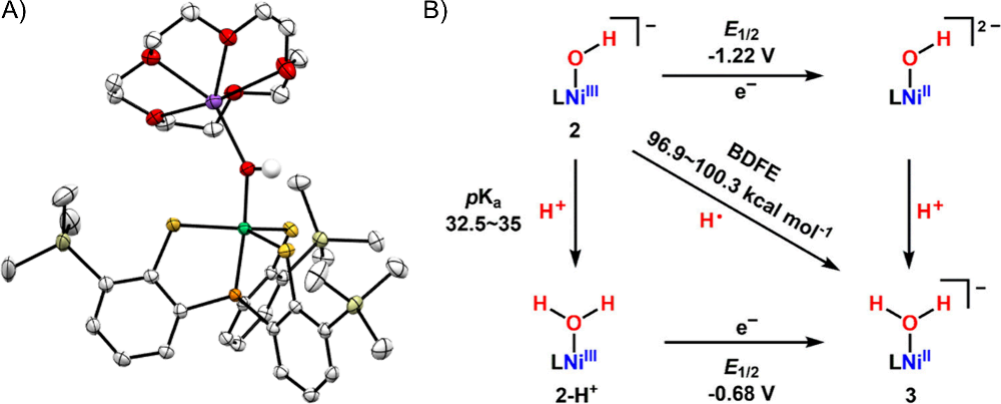
(A) Molecular structure of [Ni­(PS3″)­(OH)]^−^. Thermal ellipsoids are set to the 50% probability level and hydrogen atoms have been omitted for clarity with the exception of the Ni–OH group. (B) Thermodynamic square scheme for [Ni­(PS3″)­(OH)]^−^ with relevant redox potentials, p*K*_a_s and the (Ni–OH)–H BDFE. Adapted from ref 547. Copyright 2025 American Chemical Society.

It should be noted that several high-valent nonoxo Ni complexes have been reported in the literature, but as they fall outside the scope of this review, we point to a few of the relevant reports here.^548–551^

### 5.3 Reactivity of Ni–Oxo Complexes

As discussed in the previous section, the evidence of true Ni–oxo complexes in the literature is limited. Given that there is also debate about whether Ni-based species even play an active catalytic role in C–H activation when oxidants like *m*-CPBA are used, reactivity data for these elusive intermediates is understandably limited. Nonetheless, we have compiled the limited extant data for several common substrates in Table 10.

**Table 10:** Second-Order Rate Constants for HAA and OAT Reactions of Ni–Oxo Complexes (see Scheme 17 for Structural Drawings)^a^

complex	temperature (K)	*k*_2_ (xanthene) (M^–1^s^–1^)	*k*_2_ (DHA) (M^–1^s^–1^)	*k*_2_ (CHD) (M^–1^s^–1^)	*k*_2_ (fluorene) (M^–1^s^–1^)	*k*_2_ (styrene) (M^–1^s^–1^)	*k*_2_ (thioanisole) (M^–1^s^–1^)	ref
[Ni^III^(TMG_3_tren)(O(H))]^+^	243	1.31 × 10^–2^	1.25 × 10^–2^	7.26 × 10^–3^				^541^
[Ni^III^(L)(O)]	243	2.93	2.62	1.69	2.8 × 10^–1^	4.5 × 10^–1^	5.6 × 10^–1^	^542^
[Ni^III^(TAML^4–^)(O^•–^)(OH)]^3–^	233	1.2		1.1	3.2 × 10^–1^	2.5	4.3 × 10^3^	^546^
[Ni^III^(PS3″)(OH)]^−^	308	1.26 × 10^–3^	5.02 × 10^–4^	7.87 × 10^–3^	8.70 × 10^–1^			^547^

a Ligand nomenclature: PS3″^3−^ = tris-(benzenethiolato)­phosphine.

## 6 Challenges for Terminal Copper–Oxo Complexes

[CuO]^+^ species are often invoked as high-valent intermediates in the catalytic cycles of copper-containing monooxygenases and their synthetic analogues, where they facilitate the oxidative activation of strong C–H bonds, including those in polysaccharides and methane.^121,123,125,552–555^ Despite over four decades of foundational work by Itoh,^556,557^ Karlin,^558–562^ Kitajima,^564,565^ Stack,^566,567^ Tolman^568–570^ and others on model systems, and Solomon,^121,571^ Rosenzweig,^123,125,572^ Bissaro,^573^ Sørlie,^574^ Eijsink,^146^ Walton^575,576^ and others on copper–dioxygen chemistry in enzymes, terminal high-valent copper–oxo species have never been observed experimentally. While various copper–dioxygen intermediates are well-documented (see Figure 49), the hypothetical mononuclear [CuO]^+^ species (“copper–oxyl”) remains especially elusive. Most insights into such species derive from computational studies or gas-phase observations, with no confirmed solution-phase detection, highlighting the enduring challenge of capturing this powerful oxidant in an enzyme or model system. Proposals for their involvement in copper-mediated oxidation reactions in solution date back more than two decades.^577–579^ Despite extensive computational studies that have evaluated the [CuO]^+^ unit in the gas phase,^580–586^ in a protein environment,^587,588^ and in complexes in solution,^589–592^ suggesting that such intermediates are highly potent oxidants, direct experimental observation has thus far been limited to the gas phase. Terminal copper–oxo species remain notably elusive due to several intertwined challenges: first, they lie beyond the so-called “oxo wall”, as late transition-metal d-electron configurations tend to destabilize metal–oxo multiple bonding (discussed in section 2.2). Second, even when formed, copper–oxo species are expected to be highly reactive and short-lived, making thorough experimental characterization challenging.

**Figure 49:**
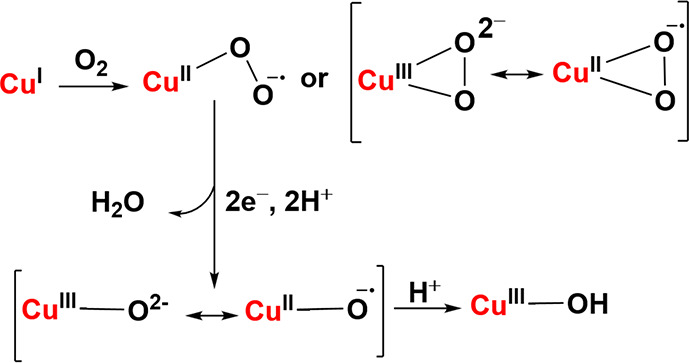
Proposed Cu/O_2_ activation pathways and intermediates. Adapted from ref 569. Copyright 2017 American Chemical Society.

Computational and experimental evidence shows that the bond between copper and oxygen in the [CuO]^+^ ion is much weaker than in the more robust Fe­(IV)–oxo unit. The bond dissociation energy of [CuO]^+^ is found to be only about 31.1 ± 2.8 kcal/mol experimentally (in the gas phase),^593^ and 25 kcal/mol by theoretical studies.^580^

### 6.1 Gas Phase and Solution Studies

In 2011, Schwarz and co-workers successfully generated bare [CuO]^+^ ions in the gas phase and demonstrated their reactivity toward methane.^594^ They also studied [(phen)­CuO]^+^ complexes, revealing the formation of reactive Cu­(III)–oxo species.^583,595^ Roitová and co-workers started their gas-phase syntheses from Cu­(II) chlorate precursors, which allowed for the preparation of [(L)­CuO]^+^ complexes with various ligands (e.g., MeCN, phen, PQ). Among these, [(MeCN)­CuO]^+^ exhibits the highest reactivity, oxidizing water to H_2_O_2_ and hydroxylating ethane. In contrast, [(phen)­CuO]^+^ and [(PQ)­CuO]^+^ participated only in oxygen-exchange reactions. All complexes react similarly with ethylene, undergoing OAT.

Attempts to isolate [CuO]^+^ cores in solution upon reaction of a Cu­(I) precursor with oxo transfer reagents such as pyridinium N-oxides or PhIO remained unsuccessful (with the exception of one ESI-MS data set).^596^ In a related approach, Karlin and co-workers recently treated a series of Cu­(I) complexes with para-substituted *N*,*N*-dimethylaniline N-oxides (DMAO) as O atom donors. The results indicate the transient formation of a highly reactive copper–oxyl species capable of hydroxylating the strong C–H bonds (∼90 kcal/mol) of the *N*,*N*-dimethylaniline product formed after O atom transfer to the Cu­(I) precursors.^597^

### 6.2 Peroxidase Activity

For LPMOs, following the reaction with either dioxygen and electrons or hydrogen peroxide, a controlled Fenton-like mechanism has been proposed. In this mechanistic framework, the species responsible for the activation of strong C–H bonds could involve either hydroxyl radicals and/or copper–oxyl intermediates.^598^ Analogously, the reactivity observed for Cu­(II) complexes in the presence of H_2_O_2_ may proceed through either a “free radical” pathway or a “metal-centered” mechanism. Very recently, Castillo, Simaan and co-workers evaluated three different assays for LPMO-like activity measurements on various substrates (soluble to extended insoluble polysaccharides) using different model complexes. Their mechanistic investigations suggest involvement of a hydroxyl radical in substrate oxidation in all cases.^157^

Cu­(II)–alkylperoxo complexes (Cu^II^–OOR, where R = cumyl or ^t^Bu) have emerged as valuable synthetic models, inspired by the fact that high-valent iron–oxo species (e.g., Fe­(IV)–oxo or Fe­(V)–oxo) can be formed via homolytic or heterolytic O–O bond cleavage of Fe^III^–OOR intermediates.^599^ Accordingly, in these copper systems, Cu^II^–OOR compounds are widely employed as precursors that can potentially generate Cu­(II)–O^•^ species, and they have been utilized to oxidize external substrates.^557,563,600–610^ Most notably, Kitajima and co-workers were the first to accomplish detailed structural and spectroscopic characterization of an LCu^II^–OOR complex using the hydrotris­(pyrazolyl)­borate coligand (L = HB­(3,5-^i^Prpz)_3_^–^).^563^ Very recently, Karlin, Solomon and co-workers have presented experimental data demonstrating that a peroxygenase-type mechanism can operate efficiently in bioinspired model systems. In one such study, the copper­(I) complex [(TMG_3_tren)­Cu]^+^ was reacted with a substoichiometric amount of dry hydrogen peroxide, resulting in the hydroxylation of the TMG_3_tren coligand to form TMG_3_tren–OH (see Figure 50). The addition of hydroxyl radical scavengers completely suppressed ligand hydroxylation, supporting the generation of hydroxyl radicals and indicating participation of a Fenton-like pathway in this reactivity.^611^ A recent investigation by Hendrich, Garcia-Bosch and co-workers with the LH_2_ (= bis­(6-pivalamide-2-pyridylmethyl)-(2-pyridylmethyl)­amine) ligand framework for O_2_ and H_2_O_2_ reactivity with mononuclear copper complexes arrived at a similar conclusion. The complex [(LH_2_)­Cu^I^]^+^ reacts with dioxygen to produce [(LH_2_)­Cu^II^(OOH)]^+^ in the presence of TEMPOH, whereas the reaction with H_2_O_2_ leads to the formation of [(LH_2_)­Cu^II^(OH)]^+^ and the hydroxyl radical, which then reacts with a C–H bond of the substrate.^156^

**Figure 50:**
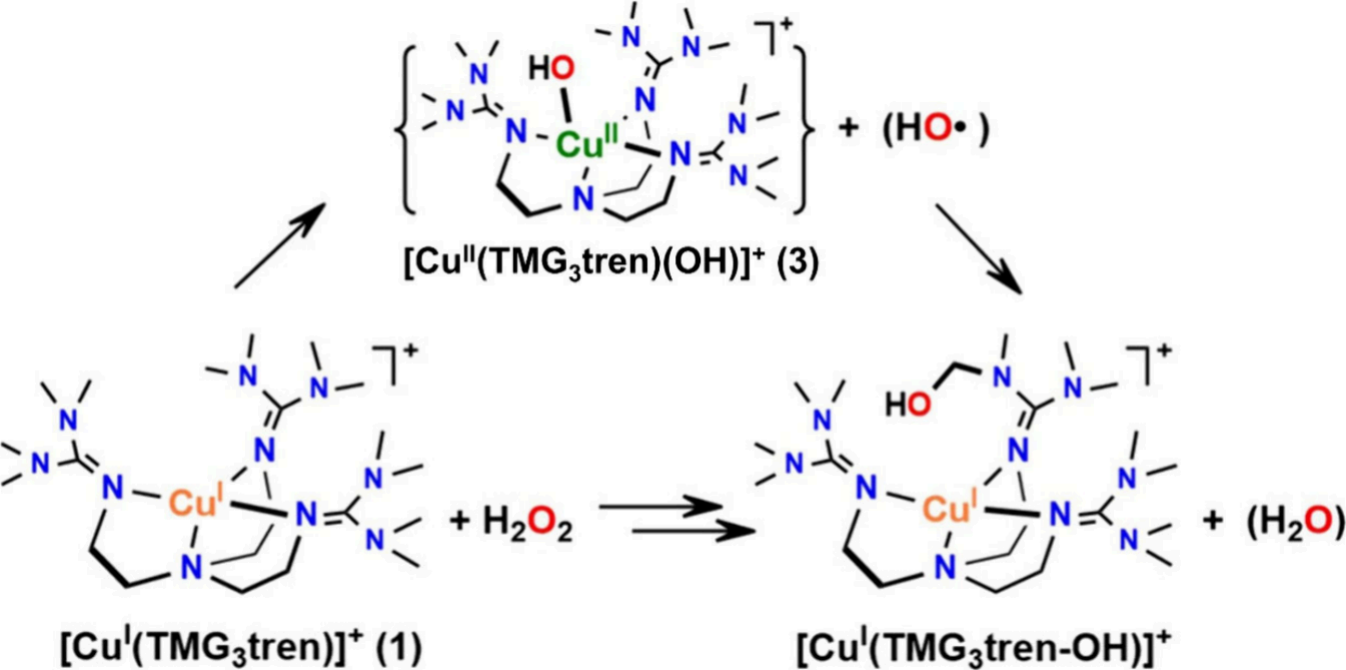
Reaction of the complex [Cu^I^(TMG_3_tren)]^+^ with dry H_2_O_2._ Reproduced with permission from ref 611. Copyright 2023 American Chemical Society.

### 6.3 Reactivity of Cu­(III)–OH

Tolman and co-workers first proposed the potential biological relevance of [Cu­(III)–OH]^2+^ species using the L = *N*,*N*′-bis­(2,6-diisopropylphenyl)-2,6-pyridinedicarboxamide ligand backbone for their studies (see Figure 51).^569^ One-electron oxidation of the tetragonal Cu­(II) complex [Bu_4_N]­[LCu^II^–OH] at −80 °C yielded the reactive intermediate [Cu^III^–OH]^2+^ identified as a Cu­(III) species based on spectroscopic and theoretical analyses. Kinetic studies revealed that this new intermediate can perform HAA reactions with DHA with a large rate constant (*k*_2_ = 1.1 M^–1^s^1^ at −80 °C). EXAFS measurements further support the Cu­(III) assignment, showing a Cu–O bond distance of approximately 1.86 Å, in contrast to the ∼1.95 Å bond length observed for the one-electron-reduced Cu­(II)–OH species. The authors have further determined a BDFE value of 90 ± 3 kcal mol^–1^ for the Cu­(II)–H_2_O species. Interestingly, the intermediate has a high p*K*_a_ of 18.8 ± 1.8 and a low *E*_1/2_ of −0.074 V vs Fc^+^/Fc in THF. This complex also reacts with THF (BDFE = 92 kcal/mol) solvent. Replacing THF with 1,2-difluorobenzene (DFB) solvent increases the stability of the intermediate. The authors further performed reactivity studies with a range of substrates for C–H bond activation.^612^ Finally, Cramer, Tolman and co-workers studied the HAA reaction mechanism for C–H bond activation, and they found that the [Cu^III^–OH]^2+^ intermediate follows a basic asynchronous concerted pathway.^613^

**Figure 51:**
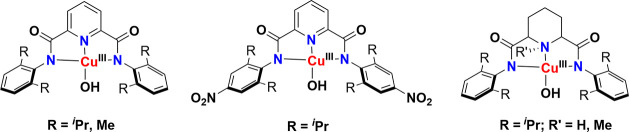
Structures of Cu­(III)–OH complexes studied by Tolman and co-workers.^569,612,614^

### 6.4 Reactivity of Cu–Oxo in Zeolites

Schoonheydt, Sels, Solomon and co-workers studied methane oxidation reactions using copper-containing zeolites, which were believed to have a dinuclear [Cu_2_O]^2+^ active sites, based upon the initial spectroscopic characterization.^615,616^ Interestingly, even after autoreduction of the copper-containing zeolite (mordenite) and the removal of the [Cu_2_O]^2+^ active sites, the zeolite retained its reactivity toward methane. In a recent study, the authors proposed the presence of a mononuclear active site, [CuOH]^+^, using different spectroscopic techniques like rRaman, MCD and EPR spectroscopy, and XAS, and it was concluded that this mononuclear site is also capable of methane oxidation.^617^ However, kinetic experiments comparing the reactivity of the [CuOH]^+^ and [Cu_2_O]^2+^ sites revealed that the binuclear site is significantly more reactive.^617^ DFT studies showed that the enhanced reactivity is due to the stabilization of [Cu_2_OH]^2+^ in the HAA product due to the electron delocalization over two copper centers.

## 7 Conclusions and Future Scope

Despite significant progress in high-valent metal–oxo chemistry, the exploration of late transition metals such as cobalt, nickel, and copper remains in early stages compared to the vast body of literature available for manganese– and iron–oxo intermediates (see Figure 52). Because the number of well characterized late-transition metal–oxo complexes is small, it is too early to draw proper conclusions about their relative reactivity. One of the key limitations is the absence of comparative studies where iron, cobalt, nickel, and copper complexes are stabilized *by the same coligand scaffold* , which is essential for a proper evaluation of reactivity trends. This lack of data hampers our ability to directly correlate reactivity with the choice of metal toward substrates such as C–H bonds. When progressing from Fe to Cu, not only does the reduction potential change, but the electronic structure of the metal–oxo unit may shift from an electrophilic oxidant to a nucleophilic or radical-type species. This observation challenges the prevailing assumption that higher redox potential directly correlates with increased reactivity. Instead, it emphasizes that the electronic structure is a crucial factor in the mechanism of substrate activation, which can vary significantly based on the electronic configuration of the active species. As we transition from high-valent Fe– to Cu–O complexes, the M–O unit transforms from a metal–oxo to a metal–oxyl species. Along the series, the HAA reaction therefore changes as well, from a PCET process, where the electron transfers to the metal and the proton to the oxo group, to a true HAT mechanism, where both the electron and the proton transfer to the oxyl ligand. However, how this affects the ability of metal−oxo complexes to activate C–H bonds, perform O atom transfer, etc., is not clear at this point in time. As research on late transition metal–oxo complexes accelerates, we will without a doubt conquer new grounds in the reactivity landscape of metal−oxo intermediates in the near future.

**Figure 52:**
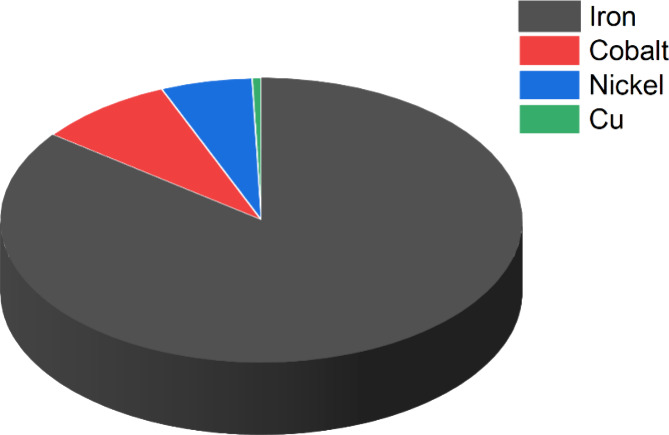
A graphical representation of the number of reports of high-valent metal–oxo intermediates in the literature.

As mentioned above, very few coligands have been used across several different transition metal–oxo complexes, limiting our ability to make direct comparisons of metal–oxo reactivity. One such ligand is TMG_3_tren, which was used across several studies to stabilize high valent Fe–, Co–, and Ni–oxo species. When reacted with dihydroanthracene (DHA), the Fe­(IV)–oxo complex demonstrates substantially greater reactivity than its cobalt and nickel counterparts, with cobalt exhibiting moderate activity and nickel the lowest (see Figure 53). However, there are significant caveats with this comparison: the corresponding Co­(IV)–oxo intermediate could only be obtained with a coordinated Lewis-acid, Sc­(OTf)_3_, which shields the Co–O unit, and in combination with the sterically very encumbering TMG_3_tren coligand, greatly reduces substrate access. In the Ni case, the corresponding intermediate is in the Ni­(III) oxidation state and likely protonated, and given the difference in reduction potential of a formally Ni­(IV) versus Ni­(III) center and the difference in basicity of a formally oxo versus hydroxo ligand, it is not surprising that the Ni­(III)–OH intermediate has a much lower reactivity than the Fe­(IV)– and Co­(IV)–oxo counterparts.

**Figure 53:**
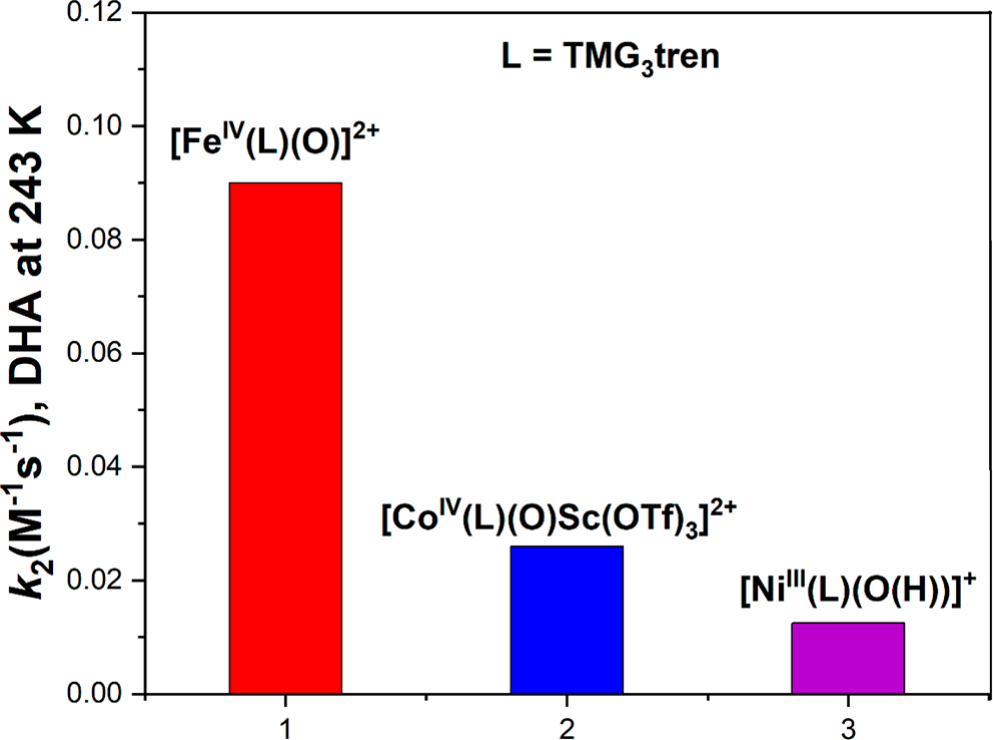
DHA oxidation rate at 243 K for metal–oxo intermediates with the TMG_3_tren coligand.^318,504,541^ For [Co^IV^(TMG_3_tren)­(O)­Sc­(OTf_3_)]^2+^, the original rate was reported at 278 K (see Table 9). A temperature correction was used for this comparison. Note that the Co and Ni complexes are not clean analogues of the Fe complex. The presence of a Lewis-acid adduct for Co and protonation of the oxo ligand for Ni changes the reduction potential and p*K*_a_ for these two high-valent intermediates. Despite these limitations, these are the only HAA reactivity data available in the literature for Fe, Co, and Ni using the same coligand framework.

Another ligand framework, TAML^4–^, was used to generate high-valent oxo species of Fe, Co, and Ni. The reactivity of these species toward both HAA and OAT substrates was subsequently studied. It was found for the thioanisole oxidation rate (see Figure 54) that the corresponding Ni–O intermediate shows higher reactivity than Co and Fe, potentially due to the increased oxyl character of the Ni–O unit. The reactivity of the formally Co­(IV)–O intermediate does not fit well into this comparison, and the reactivity of this species is likely depressed due to LA coordination and possible protonation of the oxo moiety, and reduction of the cobalt center to Co­(III) by the TAML^4–^ coligand. These results again highlight the challenges of making meaningful reactivity comparisons between high-valent Fe–O, Co–O, Ni–O and Cu–O intermediates.

**Figure 54:**
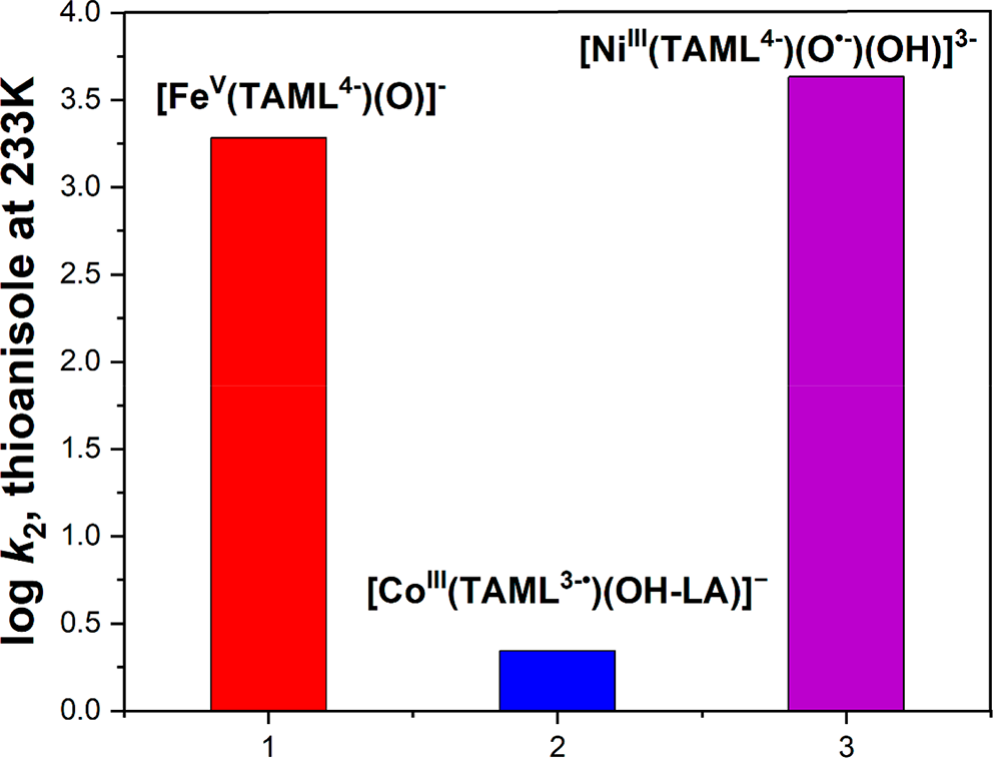
Thioanisole oxidation rate at 233 K for metal–oxo intermediates with the TAML^4–^ co-ligand framework.^252,473,546^ Since the Co and Ni complexes are not clean analogues of the Fe complex, and OAT reactivity depends on the reduction potential of the corresponding intermediate, direct comparison between these data is limited.

So far, only a handful of Co– and Ni–oxo intermediates show reactivity toward strong C–H bonds, like those found in cyclohexane. In contrast, more than 50 Fe­(IV)–oxo complexes are known to react with cyclohexane, some of which can even react with methane or ethane. Despite years of research, we are still in the earliest stages of discovery regarding the properties and reactivities of late transition metal–oxo complexes. Similarly, the full thermodynamic characterization of high-valent metal–oxo complexes to determine their BDFE values, reduction potentials and p*K*_a_s is often lacking. Table 11 lists data for examples where this has been accomplished.

**Table 11:** Thermodynamic Properties of Fe, Co, Ni, and Cu Complexes

complex	p*K*_a_	*E*_1/2_ (V)^a^	O–H BDFE (kcal/mol)	solvent	ref
[Fe^IV^(H_3_buea)(O)]^−^	10	–0.90	87	DMSO	^217^
[Co^III^PhB(^*t*^BuIm)_3_(O)]	26	–0.230	>85	MeCN	^527^
[Ni^III^(PS3″)(OH)]^−^	32–35	–1.22	96.9–100.3	DMSO	^547^
[Cu^III^(L)(OH)]^b^	18.8 ± 1.8	0.074 V	90 ± 3	THF	^612^

a vs Fc^+^/Fc.

b L = *N*,*N*′-bis­(2,6-diisopropylphenyl)-2,6-pyridinedicarboxamide

Recent studies have shown that high-valent Fe­(IV)–oxo species can be stabilized inside larger structures like organic cages and metal–organic frameworks (MOFs).^317,369^ These systems create a controlled environment around the metal–oxo group, making it more stable and easier to study. A similar strategy could also be applied in the future to explore similar high-valent species of other late transition metals like cobalt, nickel, and copper. As illustrated throughout this review, ligand design plays a crucial role in stabilizing reactive, high-valent species. A nice example for this strategy is the recent discovery of an Fe­(VII)–nitrido complex,^618^ highlighting how critical the ligand environment is in supporting such high oxidation states. This finding suggests that with the right ligand design, even more unusual high-valent metal–oxo species could be isolated. It is possible that, even as we write this review, researchers somewhere in the world are working on characterizing species like an Fe­(VI)–oxo complex or oxo/oxyl complexes with the late transition-metals. The field continues to progress rapidly, and innovative ligand platforms help push the boundaries of high-valent transition-metal chemistry.

In summary, this review explores recent advances in nonheme high-valent metal–oxo chemistry, emphasizing the spectroscopic methods used to capture and characterize these transient intermediates, inspired from their enzymatic counterparts. New spectroscopic methods, especially the free electron laser, are making their mark in further allowing scientists to obtain damage-free crystal structures of short-lived intermediates.^619^ Within bioinspired chemistry, the “oxo wall” has served as a guiding concept to rationalize and predict the reactivity of transition-metal–oxo species. While iron–oxo intermediates, especially nonheme Fe­(IV)–oxo complexes, have long dominated the field and have contributed a great deal to better our understanding of the electronic structure and reactivity of corresponding high-valent intermediates in biology, recent efforts have expanded this framework to other transition metals. Going forward, high-valent Co– and Ni–O complexes are of great interest for potential applications in oxidative C–H bond functionalization, and Cu–O model systems are critically needed to better understand monocopper oxygenase chemistry in nature. Experimental techniques such as matrix-isolation, IR and UV–vis spectroscopy at cryogenic temperatures, low-temperature EPR and UV–vis spectroscopy with rapid-mix freeze-quench trapping of intermediates, and confinement of reactive species within a rigid host (such as a MOF or molecular cage), may allow for the further stabilization or trapping of reactive late transition metal–oxo intermediates.

## Abbreviations

α-KG: α-ketoglutarate
AurF: 4-aminobenzoate N-oxygenase
BDFE: bond dissociation free energy
CAN: cerium­(IV) ammonium nitrate
CD: circular dichroism
CHD: 1,4-cyclohexadiene
Cpd 0: compound 0
Cpd I: compound I
Cpd II: compound II
CPET: concerted proton transfer electron transfer
CPO: chloroperoxidase
CSI-MS: cold spray ionization mass spectrometry
Cyt: cytochrome
DHA: 9,10-dihydroanthracene
DFT: density functional theory
ENDOR: electron–nuclear double resonance
EPR: electron paramagnetic resonance
ESI-MS: electrospray ionization-mass spectrometry
ET: electron transfer
EXAFS: extended X-ray absorption fine structure
FMO: frontier molecular orbital
HAA: hydrogen atom abstraction
HAT: hydrogen atom transfer
HRFD-EXAFS: high-energy resolution fluorescence-detected extended X-ray absorption fine structure
His: histidine
HR-MS: high resolution mass spectrometry
HRP: horseradish peroxidase
IPNS: isopenicillin N synthase
IR: infrared
IRPD: infrared photodissociation spectroscopy
KIE: kinetic isotope effect
LPMO: lytic polysaccharide monooxygenase
MCD: magnetic circular dichroism
*m*-CPBA: *meta*-chloroperbenzoic acid
MO: molecular orbital
MS: mass spectrometry
NIR: near infrared
NMR: nuclear magnetic resonance
NRVS: nuclear resonance vibrational spectroscopy
OAT: oxygen atom transfer
OEC: oxygen evolving complex
P4H: prolyl 4-hydroxylase
PCET: proton-coupled electron transfer
PheH: phenylalanine hydroxylase
PhIO: iodosylbenzene
pMMO: particulate methane monooxygenase
PS II: photosystem II
PT: proton transfer
PTD: pseudotetrahedral
QM/MM: quantum mechanics/molecular mechanics
RO: Rieske oxygenase
rRaman: resonance Raman
SAM: S-adenosyl methionine
SCS: second coordination sphere
sMMO: soluble methane monooxygenase
SOC: spin–orbit coupling
TauD: taurine dioxygenase
TBP: trigonal bipyramidal
TD-DFT: time-dependent density functional theory
THF: tetrahydrofuran
TON: turnover number
TSR: two-state reactivity
TTBP: 2,4,6-tri-*tert*-butylphenol
TyrH: tyrosinase hydroxylase
UV–vis: ultraviolet–visible
VDOS: vibrational density of states
VESI-MS: voltammetry electrospray ionization mass spectrometry
visPD: visible photodissociation spectroscopy
VT-MS: variable-temperature mass spectrometry
VTVH: variable-temperature variable-field
XAS: X-ray absorption spectroscopy
XANES: X-ray absorption near edge structure
XES: X-ray emission spectroscopy
